# (3 + 2)-Cycloaddition of bicyclobutanes and thioketones: access to 2-thiabicyclo[2.1.1]hexanes without the use of catalysts or light†

**DOI:** 10.1039/d5sc00125k

**Published:** 2025-04-02

**Authors:** Daniil A. Knyazev, Malini George, Daniel B. Werz

**Affiliations:** a Albert-Ludwigs-Universität Freiburg, Institute of Organic Chemistry Albertstr. 21 79104 Freiburg Germany daniel.werz@chemie.uni-freiburg.de http://www.werzlab.de/

## Abstract

A novel approach to the synthesis of a 2-thiabicyclo[2.1.1]hexane scaffold has been described. This method utilizes two highly reactive species: bicyclo[1.1.0]butanes (BCBs) and thioketones. Their high reactivity enabled the formation of the desired product to occur under ambient conditions, without the need for catalysts, additives or light irradiation. To the best of our knowledge, this is the first rational synthesis of this specific skeleton. A variety of carbonyl-substituted BCBs, with or without a substituent at the other bridgehead, and thioketones were examined.

## Abstract 2

A novel approach to the synthesis of a 2-thiabicyclo[2.1.1]hexane scaffold has been described.

## Graphical abstract

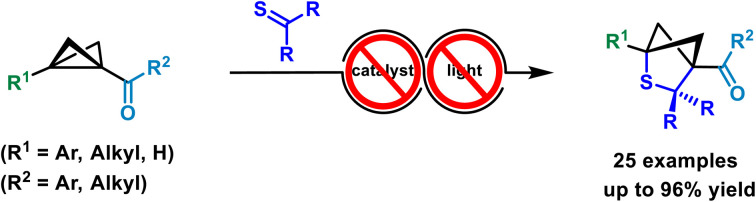

## Introduction

**Scheme 1:**
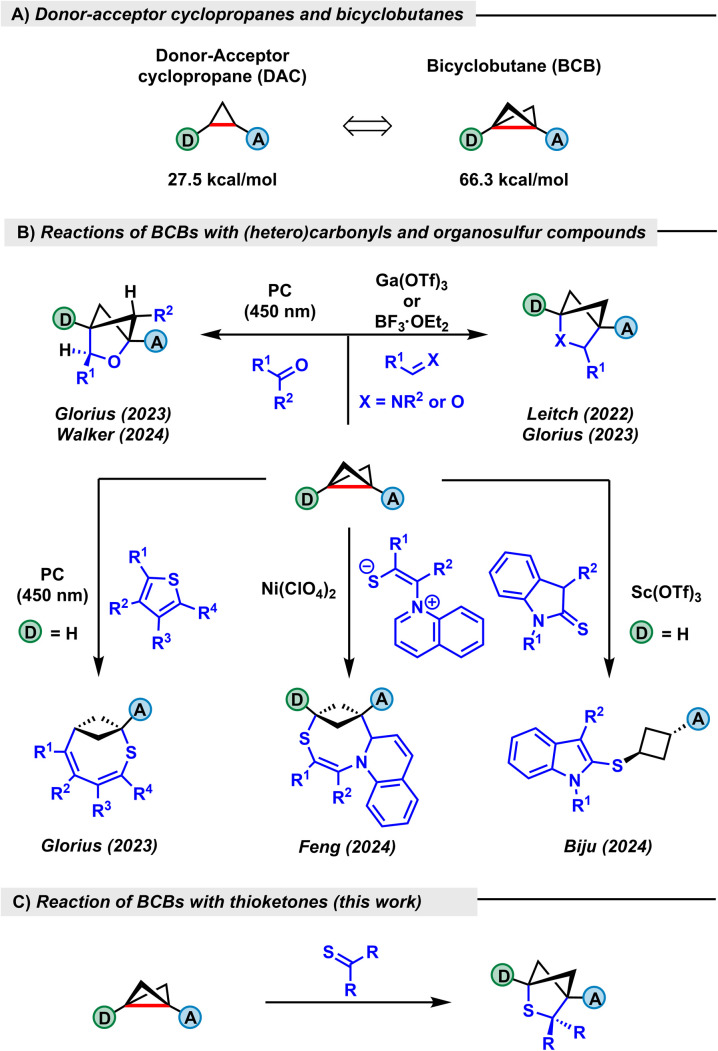
(A) Difference in strain energies between DACs and BCBs. (B) Representative examples of reactions between BCBs and sulfur-containing compounds. (C) Access to 2-thiabicyclo[2.1.1]hexanes (this work).

In recent years, there has been increasing interest in the transformations of bicyclo[1.1.0]butanes (BCBs).^1^ Their cycloaddition products are often bioisosteres of heterocyclic systems being used in pharmaceuticals or agrochemicals.^2^ Synthetically, BCBs with aryl donors and carbonyl acceptors can be regarded as extremely strained variants of well-known donor–acceptor cyclopropanes (DACs).^3^ Whereas the strain energy of a DAC is about 27.5 kcal mol^−1^, it increases in the case of BCBs to 66.3 kcal mol^−1^ because of the additional three-membered ring (Scheme 1A).^4^ This very high Baeyer strain being much more than the sum of two DACs leads to destabilization of the central C–C bond and highly distorted angles. The first BCB was prepared in the 1960s and the reaction with an enamine was established at this time.^5^ However, some years ago BCBs were rediscovered and a renaissance in this chemistry has started,^6^ especially because photochemical conditions have been applied, besides their activation by Lewis acids. Photochemically generated radicals or respective species in their triplet state allow for an easy attack of the highly strained σ bond. Alternatively, BCBs are also able to reach a triplet state when irradiated. BCB chemistry has led to numerous bicyclic versions of well-known carbocycles^7,8^ and heterocycles containing oxygen^9–11^ and nitrogen.^12,13^ Among the variety of reports, we would like to highlight the ones describing (2 + 2)-cycloaddition reactions of BCBs with carbonyls and imines. In 2022, the Leitch group published a pioneering study of the reactivity of BCBs with imines, achieving a 2-azabicyclo[2.1.1]hexane skeleton in the presence of a gallium catalyst.^12^ Later that year, another study published by the Glorius group demonstrated a photochemical approach to 2-oxabicyclo[2.1.1]hexanes from BCBs and benzoylformate esters with reversed regiochemistry of the ketone group addition.^9^ Another report, published by the same group in 2023, showed analogous behaviour of benzaldehydes in the reaction with BCBs catalysed by boron trifluoride etherate,^10^ while reversed photoaddition is also possible, as reported by the Walker group.^8^ The aforementioned examples exhibit BCB reactivity with different classes of unsaturated compounds such as imines, ketones and aldehydes. Nevertheless, it is notable that ketones do not undergo addition *via* the classic Lewis acid promoted pathway. At the same time, there has been much less activity in the field of sulfur-containing ring systems. In 2023, the Glorius group reported two transformations with BCBs under visible-light irradiation.^14^ In the first work, the authors describe an insertion of a BCB into thiophene derivatives (Scheme 1B). The process proceeds *via* photochemical activation of thiophene, followed by dearomative insertion. In the next paper, alkynyl, alkenyl and allylic thioethers were employed. The well-known concept of photoredox-mediated C–S σ-bond cleavage, when applied to BCBs, leads to a series of 1,3-bifunctionalized cyclobutanes. It is noteworthy that both transformations were executed in the presence of photocatalysts.

In 2024, Feng and co-workers demonstrated a Lewis acid catalyzed cycloaddition of a BCB with 3-benzylideneindoline-2-thione derivatives, affording thiabicyclo[4.1.1]octanes.^15^ In another study published by the same group, the authors showed the dual nature of pyridinium 1,4-zwitterionic thiolates, allowing access to either (5 + 3)-cycloaddition products or substituted thiabicyclohexanes, depending on the choice of the catalyst.^16^ Also in 2024, the group of Biju reported the use of thiolactams and thioindolines leading to a formal hetero-ene reaction with bicyclobutanes.^17^ As a result, ring-opened products were obtained. As early as 2019, a study by Malins and co-workers showed the utility of BCBs as a tool for late-stage peptide functionalization, selectively alkylating the free cysteine thiol group of the model substrate.^18^ Higher oxidation states of sulfur were also employed in strain-release reactions, as demonstrated by Anderson and Mykhailiuk in 2022.^19^ In their work, *in situ* generated sulfonyl halide species react with strained hydrocarbons, resulting in 1,3-halosulfonylation products. Recently, the Hari group demonstrated that photochemically generated sulfur-centered radicals add to BCBs and the emerging carbon-centered radical undergoes intramolecular cyclization with the acceptor group attached to the BCB.^20^ These examples demonstrate that complex sulfur-containing molecular scaffolds are easily obtained by BCB chemistry. However, one of the simplest transformations using sulfur-containing compounds has not been investigated so far. As early as 2017, our group demonstrated that thioketones easily insert into DACs using AlCl_3_ as a Lewis acid.^21^ The reaction was even possible without a catalyst when it was conducted in an intramolecular fashion. With the high strain energy of BCBs in mind and the great nucleophilicity of the sulfur in the thiocarbonyl unit, we anticipated that an instantaneous reaction of BCBs with thioketones without using any light, catalyst or any other additive might be possible. This transformation would allow very simple access to the 2-thiabicyclo[2.1.1]hexane skeleton (Scheme 1C), a scaffold that has only been obtained so far as a side product through (oxidative) decomposition reactions of thiiranes.^22^

## Results and discussion

**Scheme 2:**
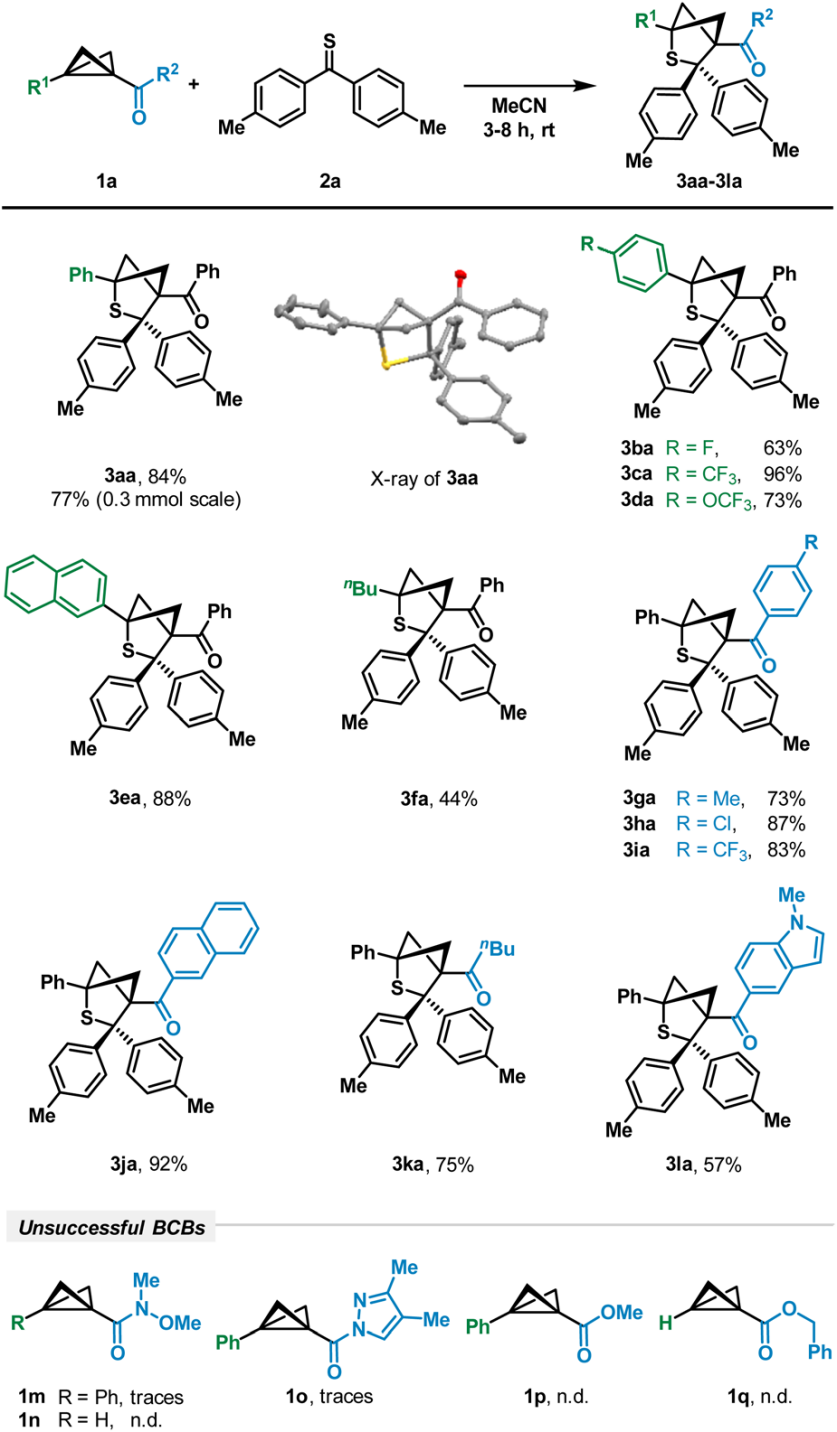
Scope of (3 + 2)-cycloaddition with respect to BCBs. Reaction conditions: 1 (100 μmol), 2 (250 μmol), MeCN (4 mL), rt, and 3–8 h.

To investigate the desired transformation, we chose a variety of BCBs differing in the acceptor moiety. To our delight, the respective ketone-substituted BCB 1a was smoothly converted by reaction with thioketone 2a into the desired bicyclic product 3aa using acetonitrile as the solvent (Scheme 2). The 4,4′-dimethyl-substituted variant 2a of the thiobenzophenone as the model thioketone was chosen because of its increased stability and easier detection by NMR analysis. Attempts to use other solvents such as dichloromethane (CH_2_Cl_2_), toluene or tetrahydrofuran (THF) led to decreased yields of product formation, emphasizing the importance of MeCN as the most polar solvent utilized (see the ESI† for details). An X-ray crystallographic analysis unequivocally proved the regiochemistry of the attack. Various aryl units bearing weak or strong electron-withdrawing substituents such as *p*-fluorophenyl (3ba, 63%), *p*-trifluoromethylphenyl (3ca, 96%) and *p*-trifluoromethoxyphenyl (3da, 73%) furnished the desired products (Scheme 2).

The naphthyl residue was also tolerated (3ea). In addition to aryl groups, a BCB with only a weakly electron-donating aliphatic *n*-butyl group was successfully converted into the corresponding thiabicyclohexane scaffold in a moderate yield of 44% (3fa). These results demonstrate that – similar to DACs – aryl groups are able to increase the reactivity in comparison to aliphatic residues.^23^ Interestingly, the reaction was less sensitive to variations of the ketone acceptor group; these modifications typically had no significant impact. BCBs with aryl residues bearing a weak donor (3ga), a weak acceptor (3ha) and a strong acceptor (3ia) were smoothly converted in yields of 73–87%. An extension of the π-system to naphthyl afforded product 3ja in 92% yield, whereas an *n*-butyl moiety gave target molecule 3ka in 75% yield. The attachment of the indolyl moiety as an example of a heteroaryl was also possible and led to product 3la in a moderate yield of 57%. However, changing the type of acceptor unit to amide-substituted BCBs such as Weinreb amides 1m, 1n or pyrazolyl amide 1o only led to traces of the product under our reaction conditions (Scheme 2). With ester-substituted derivatives such as 1p and 1q, we were not able to detect the products at all.

The presented protocol demonstrated remarkable robustness, enabling the reaction to progress without the need for an inert atmosphere or the use of molecular sieves. To ensure that ambient light does not play a role in the reaction and potentially activates one of the two substrates, the experiments were also carried out in the dark. These studies using BCB 1a and thioketone 2a reproduced the product formation with the same yield.

**Scheme 3:**
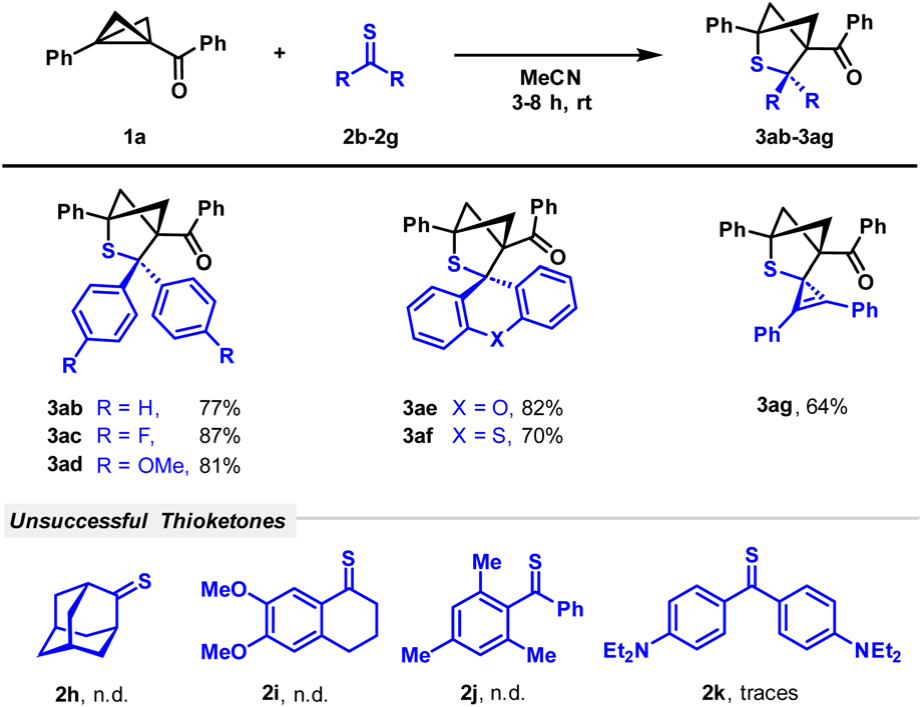
Scope of (3 + 2)-cycloaddition with respect to thioketones. Reaction conditions: 1 (100 μmol), 2 (250 μmol), MeCN (4 mL), rt, and 3–8 h.

After having investigated the scope of BCBs, we turned our attention to a variety of thioketones to be employed in the reaction (Scheme 3). The limited stability of thioketones forced us to narrow our substrate scopes since congeners with two aliphatic groups are not very stable. However, thioketones stabilized with various substituted and unsubstituted aryl groups such as *p*-fluorophenyl as an example of a substituent with a weak electron-withdrawing effect or *p*-methoxyphenyl and xanthone-based molecules as examples of donor residues were employed and demonstrated good to excellent reactivity. The yields of the corresponding products 3ab–3af ranged from 70 to 87%. The insertion of the highly polarized thioketone moiety of a cyclopropenethione into the BCB afforded spiro product 3ag in a yield of 64%. Adamantanethione 2h did not show any conversion and mixed aliphatic/aromatic thioketones showed maximum traces of the product. No product formation was observed while treating the BCB with sterically hindered thioketone 2j. The very electron-rich thioketone 2k bearing two diethylamino residues was also not successfully converted. We explain reduced reactivity by highly electron-donating amino groups, whose positive mesomeric effect reduces positive charge on the carbon of the thiocarbonyl group.

**Scheme 4:**
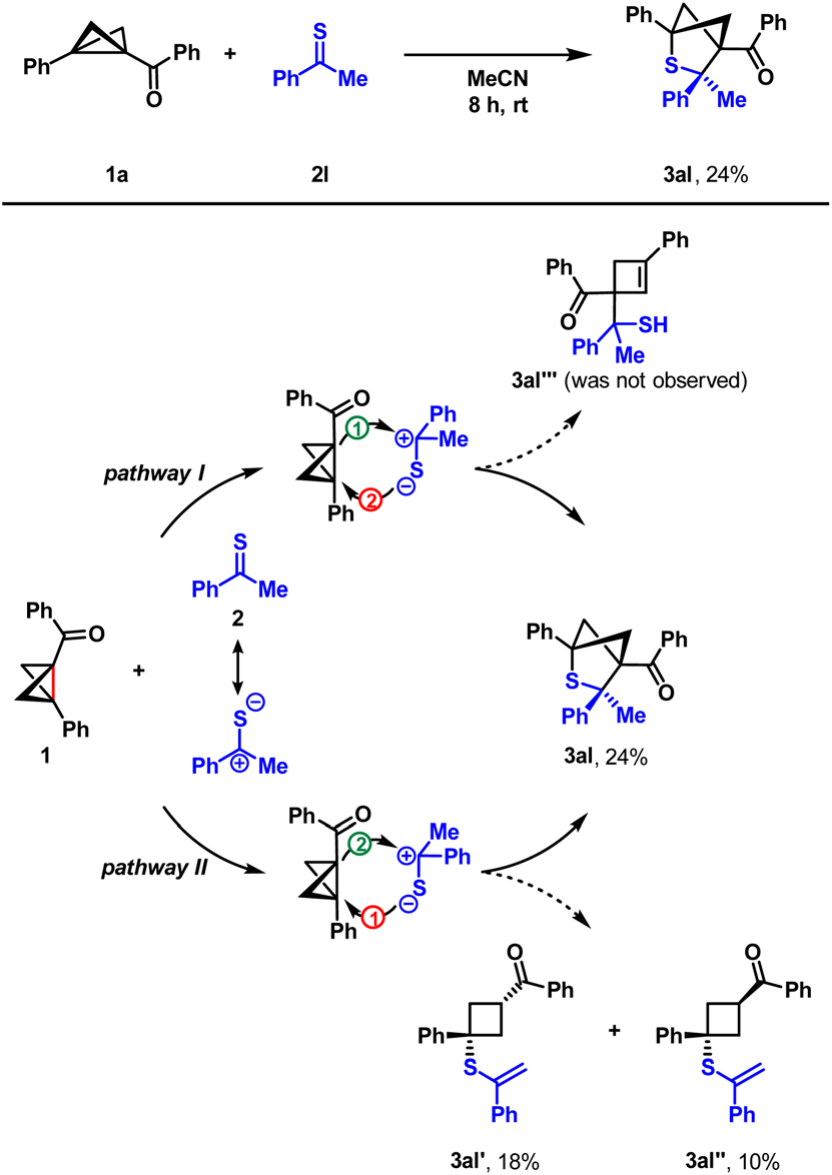
Reaction of BCB 1a and thioacetophenone 2l and a plausible mechanism.

Additionally, we studied the reactivity of thioacetophenone 2l, since the result of its reaction with BCB 1a provides us with an insight into the mechanistical nuances of the process (Scheme 4). In general, two plausible mechanisms for the observed process might be suggested, since both BCBs and thioketones exhibit a dual nature, being reactive as both nucleophiles and electrophiles. In the first (pathway I), the BCB initiates a nucleophilic attack on the thioketone, followed by ring-closure, resulting in the bicyclic product 3. In this scenario, we would expect a detectable amount of a side product **3al′′′**, since its formation was observed in similar Lewis acid promoted processes with aldehydes^10^ and imines.^12^ The second mechanism (pathway II) derives from a sulfur-initiated attack on the BCB with the formation of an intermediate enolate, which further reacts with the thiocarbonyl carbon closing the cycle. In the reaction of BCB 1a and thioacetophenone 2l, along with the expected bicyclic product 3al, formed in decreased yield (24%), we observed the formation of two diastereomeric products **3al′** (18%) and **3al′′** (10%). These products are the result of a nucleophilic addition of the thioketone with further intramolecular proton transfer. The formation of side products **3al′** and **3al′′** leads to a conclusion that the process most probably proceeds through pathway II.

**Scheme 5:**
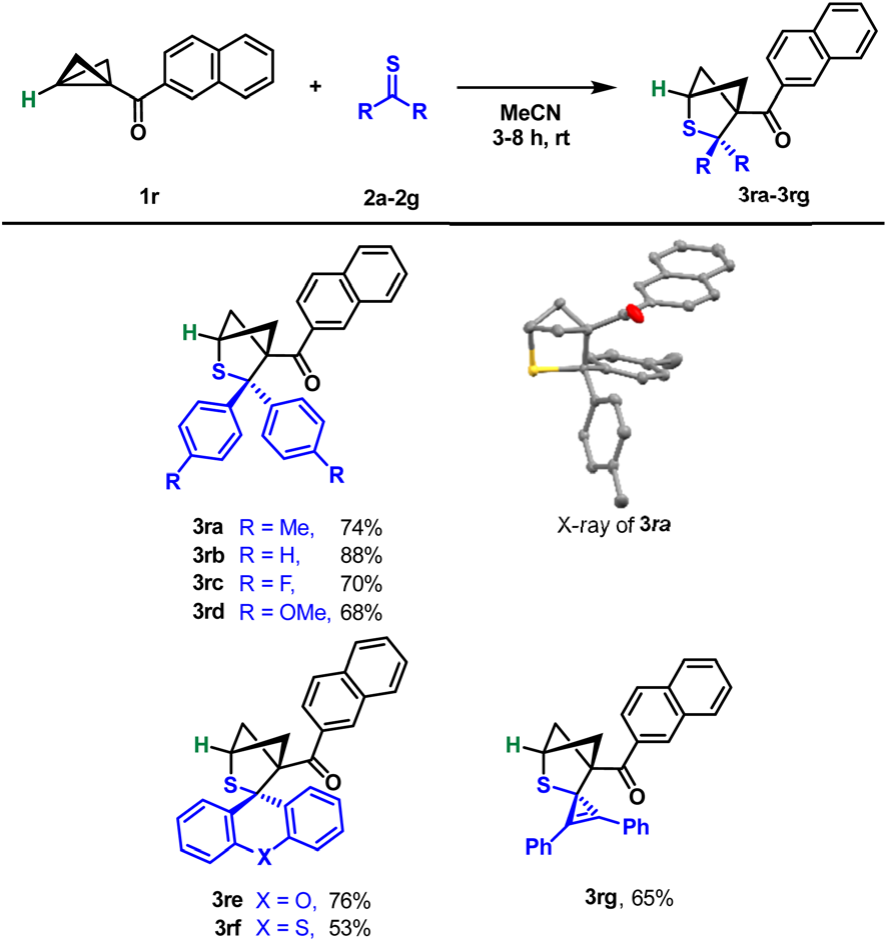
Scope of the (3 + 2)-cycloaddition of monosubstituted BCBs with thioketones. Reaction conditions: 1 (100 μmol), 2 (250 μmol), MeCN (4 mL), rt, and 3–8 h.

As the literature shows,^24^ BCBs unsubstituted on the donor side often react very differently or not at all like their analogues containing aryl or alkyl groups. For this reason, we also tested unsubstituted BCB 1r in the reaction with thioketones under our conditions (Scheme 5). To our surprise, we were able to use exactly the same reaction conditions and obtained the corresponding products 3ra–3rg in moderate to very good yields. The residues on the thioketone, whether electron-rich or rather electron-poor, had hardly any influence. It was again confirmed by an X-ray structure analysis of compound 3ra that the same regiochemistry was also obtained without the donor on the BCB.

**Scheme 6:**
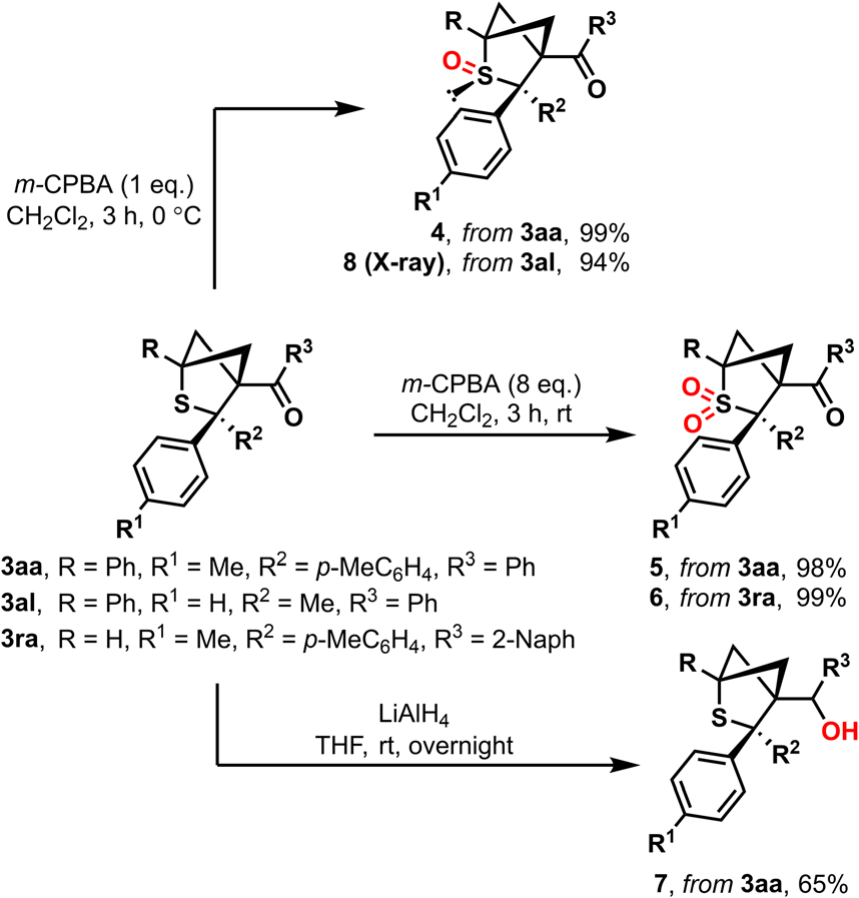
Follow-up chemistry of the parent substrates 3aa, 3al and 3ra.

The cycloaddition products 3aa and 3ra were subjected to follow-up functionalization reactions (Scheme 6). Oxidized products 4 and 5 are easily accessible *via* reaction with *m*-CPBA. The oxidation process can be directed to the desired product by varying the amount of oxidizing agent, resulting in either corresponding sulfoxide 4 or sulfone 5 in quantitative yields. Moreover, corresponding product 6 derived from monosubstituted BCB 1r was easily synthesized according to the same procedure. Monoxidation of unsymmetrical cycloaddition product 3al proceeds with complete diastereoselectivity at the same side as the methyl substituent with the formation of sulfoxide 8; its stereochemistry was confirmed by X-ray analysis. The treatment of the parent compound 3aa with LiAlH_4_ led to the formation of alcohol 7.

## Conclusions

In summary, we have developed a mild and straightforward protocol for the (3 + 2)-cycloaddition of bicyclo[1.1.0]butane ketones with thioketones. As products, 2-thiabicyclo[2.1.1]hexanes are obtained. This bicyclic skeleton has never been synthesized before by rational means. The reaction exhibits remarkable tolerance towards air and moisture and does not necessitate the use of catalysts, light, or any additives. A broad substrate scope of BCBs is tolerated as long as the acceptor moiety is a keto functionality. The transformation works smoothly with aryl and alkyl substituents at the donor side, but even unsubstituted derivatives react in the desired way. The regioselectivity of the cycloaddition was proven by X-ray analyses.

## Data availability

Details on experimental procedures, mechanistic experiments and characterization data of new compounds are available in the ESI† of the manuscript.

## Author contributions

D. K. conceptualized the project, carried out the reaction optimization and investigation of the scope, synthesized the BCBs and the thioketones and conducted follow-up transformations. M. G. synthesized the BCBs. D. B. W. conceptualized the project, provided supervision and acquired funding. The manuscript was written by D. K. with contributions from D.B.W. All authors agreed with the content of the paper.

## Conflicts of interest

There are no conflicts to declare.

## Supplementary Material

SC-016-D5SC00125K-s001

SC-016-D5SC00125K-s002

## References

[cit1] Golfmann M., Walker J. C. L. (2023). Commun. Chem..

[cit2] Denisenko A., Garbuz P., Voloshchuk N. M., Holota Y., Al-Maali G., Borysko P., Mykhailiuk P. K. (2023). Nat. Chem..

[cit3] (a) Donor Acceptor Cyclopropanes in Organic Synthesis, ed. A. T. Biju and P. Banerjee, Wiley, 2024

[cit4] Wiberg K. B. (1986). Angew. Chem., Int. Ed..

[cit5] Cairncross A., Blanchard E. P. (1966). J. Am. Chem. Soc..

[cit6] Lopchuk J. M., Fjelbye K., Kawamata Y., Malins L. R., Pan C.-M., Gianatassio R., Wang J., Prieto L., Bradow J., Brandt T. A., Collins M. R., Elleraas J., Ewanicki J., Farrell W., Fadeyi O. O., Gallego G. M., Mousseau J. J., Oliver R., Sach N. W., Smith J. K., Spangler J. E., Zhu H., Zhu J., Baran P. S. (2017). J. Am. Chem. Soc..

[cit7] Hu S., Pan Y., Ni D., Deng L. (2024). Nat. Commun..

[cit8] Golfmann M., Reinhold M., Steen J. D., Deike M. S., Rodemann B., Golz C., Crespi S., Walker J. C. L. (2024). ACS Catal..

[cit9] Liang Y., Kleinmans R., Daniliuc C. G., Glorius F. (2022). J. Am. Chem. Soc..

[cit10] Liang Y., Paulus F., Daniliuc C. G., Glorius F. (2023). Angew. Chem., Int. Ed..

[cit11] Deswal S., Guin A., Biju A. T. (2024). Angew. Chem., Int. Ed..

[cit12] Dhake K., Woelk K. J., Becica J., Un A., Jenny S. E., Leitch D. C. (2022). Angew. Chem., Int. Ed..

[cit13] Wu F., Wu W.-B., Xiao Y., Li Z., Tang L., He H.-X., Yang X.-C., Wang J.-J., Cai Y., Xu T.-T., Tao J.-H., Wang G., Feng J.-J. (2024). Angew. Chem., Int. Ed..

[cit14] Wang H., Shao H., Das A., Dutta S., Chan H. T., Daniliuc C., Houk K. N., Glorius F. (2023). Science.

[cit15] Wang J.-J., Tang L., Xiao Y., Wu W.-B., Wang G., Feng J.-J. (2024). Angew. Chem., Int. Ed..

[cit16] Xiao Y., Wu F., Tang L., Zhang X., Wei M., Wang G., Feng J.-J. (2024). Angew. Chem., Int. Ed..

[cit17] Guin A., Deswal S., Harariya M. S., Biju A. T. (2024). Chem. Sci..

[cit18] Schwartz B. D., Zhang M. Y., Attard R. H., Gardiner M. G., Malins L. R. (2020). Chem.–Eur. J..

[cit19] Pickford H. D., Ripenko V., McNamee R. E., Holovchuk S., Thompson A. L., Smith R. C., Mykhailiuk P. K., Anderson E. A. (2023). Angew. Chem., Int. Ed..

[cit20] Singha T., Bapat N. A., Mishra S. K., Hari D. P. (2024). Org. Lett..

[cit21] Augustin A. U., Sensse M., Jones P. G., Werz D. B. (2017). Angew. Chem., Int. Ed..

[cit22] Tokitoh N., Choi N., Goto M., Ando W. (1989). J. Org. Chem..

[cit23] Kreft A., Lücht A., Grunenberg J., Jones P. G., Werz D. B. (2019). Angew. Chem., Int. Ed..

[cit24] Zhou J.-L., Xiao Y., He L., Gao X.-Y., Yang X.-C., Wu W.-B., Wang G., Zhang J., Feng J.-J. (2024). J. Am. Chem. Soc..

