# Supplementary material for: (3 + 2)-Cycloaddition of bicyclobutanes and thioketones: access to 2-thiabicyclo[2.1.1]hexanes without the use of catalysts or light
Source: Chem Sci. 2025 Apr 2;16(19):8588–93. doi: 10.1039/d5sc00125k (PMC12046421; doi:10.1039/d5sc00125k)
Supplement: SC-016-D5SC00125K-s001 [file SC-016-D5SC00125K-s001.pdf]

Supplementary Information (SI) for Chemical Science.
This journal is © The Royal Society of Chemistry 2025

(3+2)-Cycloaddition of bicyclobutanes and thioketones: access to 2-thiabicyclo[2.1.1]hexanes without the use of catalysts or light

Daniil A. Knyazev, Malini George and Daniel B. Werz*

Albert-Ludwigs-Universität Freiburg,
Institute of Organic Chemistry,
Albertstr. 21, 79104 Freiburg, Germany

(*corresponding author: daniel.werz@chemie.uni-freiburg.de)

Table of Contents

Table of Contents 2
1. General Experimental 3
2. General Procedures 4
3. Synthesis of Starting Materials 5
4. Optimization of the reaction conditions 13
5. Products from reactions of BCBs with thioketones 14
6. Functionalisation products 34
7. NMR Spectra 39
8. Crystal Structure Determinations 91
References 120

1. General Experimental

All solvents were distilled before use and stored over molecular sieves unless otherwise stated. Air- and moisture-sensitive reactions were carried out in oven-dried or flame-dried glassware, septum-capped under atmospheric pressure of argon. Commercially available compounds were used without further purification unless otherwise stated. For all purifications by column chromatography Silica 60 (40-63 µm pore size) from *Macherey-Nagel* was used.

Proton (^{1}H), carbon (^{13}C) and fluorine (^{19}F) NMR spectra were recorded on a *Bruker* AVIII HD 300, *Bruker* Avance II 400, Bruker DRX 500 or 700 MHz Bruker Avance III Neo 700 instrument using the residual signals from $CHCl_3$, δ = 7.26 ppm and δ = 77.16 ppm as internal reference for ^{1}H and ^{13}C chemical shifts, respectively. Additionally, tetramethylsilane (TMS, δ = 0.00 ppm; 0.03%) was added to NMR samples. The following abbreviations were used for ^{1}H, ^{13}C, and ^{19}F NMR chemical shifts: s = singlet, br.s =broad singlet, d = doublet, t = triplet, q = quartet, m = multiplet. The chemical shift δ is given in ppm. ESI-HRMS and APCI-HRMS was carried out on an Exactive (Thermo Scientific) Orbitrap instrument. GC-QTOF-APCI-HRMS was carried out on an Agilent instrument. IR spectra were recorded on a Spectrum Two FT-IR Spectrometer from *Perkin Elmer* as thin films. Melting points of solid products were recorded on a *Schorpp* MPM-HV2. Gel permeation chromatography was performed on a Japan Analytical Industry LaboACE Recycling Preparative HPLC using a JAIGEL-2H column. Purification by preparative normal phase HPLC was carried out on a Shimadzu Nexera LC Prep System equipped with a LC-20AP pump and a Macherey-Nagel VP 250/21 NUCLEODUR 100-5 silica column. An SPD-M40 photo diode array detector was used for the automated collection of fractions at 254 nm.

2. General Procedures

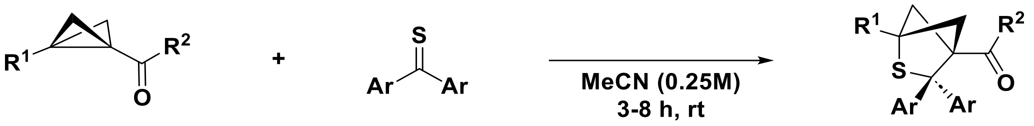

1a-r **2a-k** **3aa-3rg**

General Procedure (GP) for (3+2) Cycloaddition reaction:

In a microwave reaction vial charged with a stir bar, BCB **1a-1r** (1 equiv.) and thioketone **2a-2l** (2.5 equiv.) were added and dissolved in MeCN (4 mL). The vial was allowed to stir for a further 3-8 h at room temperature, then the volatiles were removed *in vacuo*. For purification details see the corresponding compound.

3. Synthesis of Starting Materials

3.1 Synthesis of Bicyclo[1.1.0]butanes (BCBs)

All BCBs **1a–r** were synthesized based on the previously reported procedures,[1–3] and the analytical data of known compounds were in agreement with those reported in the literature.

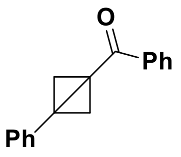

1a

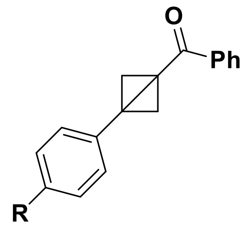

1b-d: R = F, CF_3, OCF_3

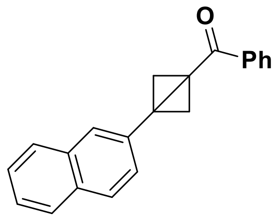

1e

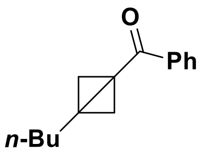

1f

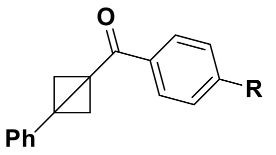

1g,h, i: R = Me, Cl, CF_3

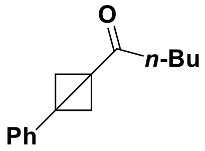

1j

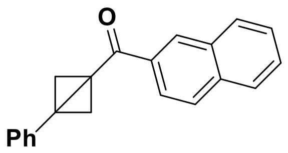

1k

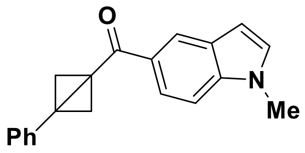

1l

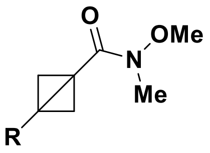

1m, n, R = Ph, H

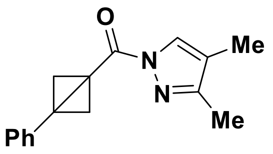

1o

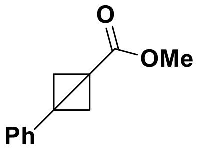

1p

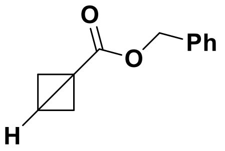

1q

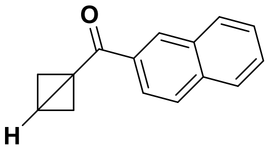

1r

(Note: All compounds were stored in a −20 °C freezer.)

BCBs **1b**, **1c**, **1e**, **1f** and **1l** were synthesized according to the following procedures:

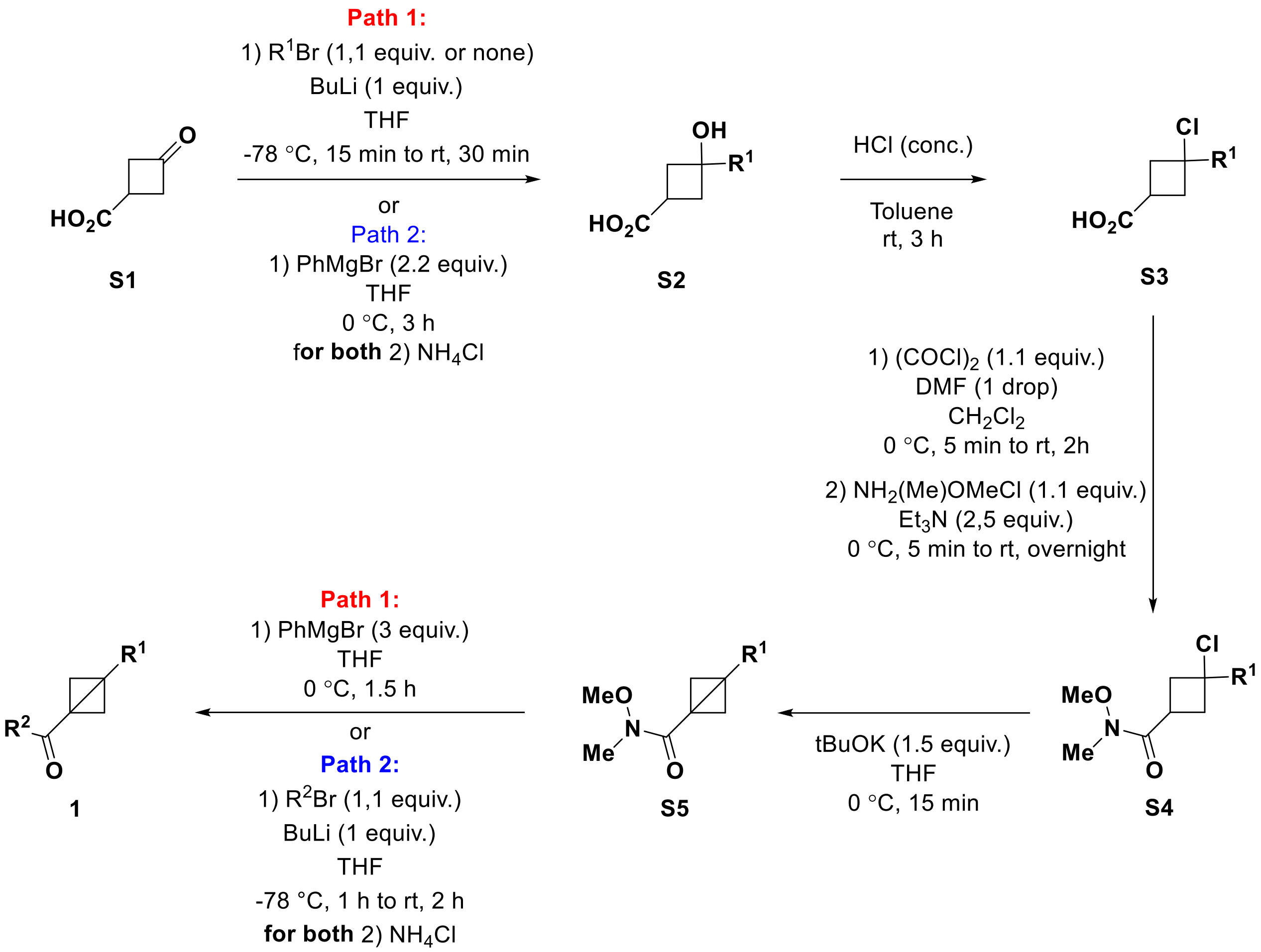

Path 1: An oven-dried round-bottom flask equipped with a stirring bar was backfilled with Ar (3 x) and capped with a septum, then R^1Br (1.1 equiv.) was added and dissolved in dry THF (1 M). Then the reaction mixture was cooled down to -78 °C and BuLi (2.3 M in hexane, 1 equiv.) was added. The reaction mixture was stirred at the same temperature for 1 h. Then, 3-oxocyclobutanecarboxylic acid **S1** (1.00 eq.) dissolved in THF was added (**In case no R^1Br was used:** Only THF was added before BuLi and **S1** was added immediately after.) The reaction mixture was allowed to reach room temperature and stirred for 30 min. After that, NH_4Cl saturated solution was added and the mixture was warmed to room temperature. The reaction was transferred into a separatory funnel and extracted with EtOAc (3x). The organic layers were combined and washed with $NaHCO_3$ 10% solution twice, the organic residue was discarded. The aqueous layers were acidified to pH 5-6 and extracted again with EtOAc (3x). The combined organic fraction was washed with brine, dried over Na_2SO_4, and the solvent was evaporated. The crude product **S2** was used in the next step without additional purification.

Path 2: 3-Oxocyclobutanecarboxylic acid **S1** (1.00 eq.) was added to an oven-dried round-bottom flask flushed with Ar and charged with a stirring bar and dissolved in dry THF (1 M). Then the reaction mixture was cooled down to 0 °C and PhMgBr (2.2 equiv.) was added slowly within 10 min. The reaction mixture was stirring for 3 h at the same temperature and then NH_4Cl saturated solution was added and it was allowed to warm up to room temperature. The reaction was transferred into a separatory funnel and extracted with EtOAc (3x). The organic layers were combined and washed with $NaHCO_3$ 10% solution twice, the organic residue was discarded. The aqueous layers were acidified to pH 5-6 and extracted with EtOAc (3x). The combined organic fraction was washed with brine, dried over Na_2SO_4 and the solvent was evaporated. The crude product **S2** was used in the next step without additional purification.

To the flask containing the crude product **S2** (1 equiv.) Toluene and HCl (conc.) (1:1, 0.15 M) were added. The reaction mixture was stirred for 3 h at room temperature. Then, the reaction mixture was diluted with water and EtOAc and transferred to a separatory funnel and the organic phase was separated. The aqueous layer was extracted additionally with EtOAc twice. The combined organic fraction was washed with brine, dried over Na_2SO_4 and the solvent was evaporated. The crude product **S3** was used in the next step without additional purification**.**

Product **S3** (1 equiv.) was dissolved in CH_2Cl_2 (0.5 M) in a round bottom flask with a stirring bar. Then the reaction mixture was cooled down to 0 °C and DMF (3 drops) and $(COCl)_2$ (1.2 equiv.) were added successively. The reaction was left to stir for 2 h, then the volatiles were evaporated and the residue was redissolved in CH_2Cl_2 (0.5 M) and cooled to 0 °C. *N*-Methoxymethanamine (1.1 equiv.) was then added to the reaction mixture followed by a dropwise addition of Et_3N (2.5 equiv.). The reaction was allowed to stir at room temperature overnight. Next, the reaction mixture was diluted with water and extracted with CH_2Cl_2 (3x). The combined organic fraction was washed with brine, dried over Na_2SO_4 and the solvent was evaporated. The pure product **S4** was purified by FCC (SiO_2, gradient CH_2Cl_2:EtOAc from 0% to 25% EtOAc)**.**

The Weinreb amide **S4** (1.00 eq.) was transferred to an oven-dried flask flushed with Ar, charged with a stirring bar and dissolved in dry THF (0.5 M). Then the reaction mixture was cooled to 0 °C and a solution of KO*t*Bu (1.5 equiv., 0.5 M) was added in one portion , and the reaction mixture was stirred for 15 min. The solution was then diluted with water and warmed to room temperature, transferred into a separatory funnel and extracted with Et_2O (3x). The combined organic fraction was washed with brine, dried over Na_2SO_4

and the solvent was evaporated (a water bath without heating). The crude product **S5** was used in the next step without additional purification.

Path 1: Weinreb amide **S5** (1.00 eq.) was added to an oven-dried flask flushed with Ar and charged with a stirring bar and dissolved in dry THF (1 M). Then the reaction mixture was cooled down to 0 °C and PhMgBr (2.2 equiv.) was added slowly within 10 min. The reaction mixture was stirring for 3 h at the same temperature and then NH_4Cl saturated solution was added and it was allowed to warm up to room temperature. The reaction was transferred into a separatory funnel and extracted with EtOAc (3 x). The organic layers were combined and washed with $NaHCO_3$ 10% solution twice, the organic residue was discarded. The aqueous layers were acidified to pH 5-6 and extracted with EtOAc (3x). The combined organic fraction was washed with brine, dried over Na_2SO_4 and the solvent was evaporated (a water bath without heating). The pure BCB **1** was afforded by FCC.

Path 2: An oven-dried round bottom flask equipped with a stirring bar was backfilled with Ar (3x) and capped with a septum, then R^2Br (1.1 equiv.) was added and dissolved in dry THF (1 M). Then the reaction mixture was cooled down to -78 °C and BuLi (2.3 M in hexane, 1 equiv.) was added. The reaction mixture was left stirring at the same temperature for 1 h. Then Weinreb amide **S5** (1.00 eq.) dissolved in THF was added (**In case no R^2Br was used:** Only THF was added to the flask before BuLi addition and Weinreb amide **S5** (1.00 eq.) dissolved in THF was added right after it.). The reaction mixture was allowed to warm up to room temperature and stir over a period of 30 min. Then NH_4Cl saturated solution was added and it was allowed to warm up to room temperature. The reaction was transferred into a separatory funnel and extracted with EtOAc (3x). The organic layers were combined and washed with $NaHCO_3$ 10% solution twice, the organic residue was discarded. The aqueous layers were acidified to pH 5-6 and extracted with EtOAc (3x). The combined organic fraction was washed with brine, dried over Na_2SO_4 and the solvent was evaporated (a water bath without heating). The pure BCB **1** was afforded by FCC.

(3-(4-Fluorophenyl)bicyclo[1.1.0]butan-1-yl)(phenyl)methanone (1b)

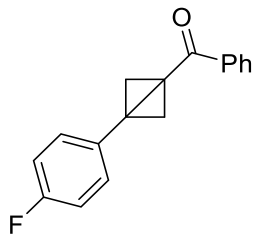

1b

BCB **1b** was synthesized according to the described procedure (Path 1). Column chromatography (SiO_2 (washed with 1% solution of Et_3N in Toluene), gradient Toluene:EtOAc from 0% to 10% EtOAc) afforded the title compound **1b** (0.44 g, 1.72 mmol, 20% from **S1**) as a pale yellow solid.

^{1}H-NMR (700 MHz, Acetone-d$_6$): δ = 7.61 – 7.57 (m, 2H), 7.52 (ddt, J = 8.7, 7.1, 1.3 Hz, 1H), 7.39 (ddt, J = 8.7, 7.5, 1.4 Hz, 2H), 7.25 – 7.21 (m, 2H), 7.03 – 6.98 (m, 2H), 3.16 (t, J = 1.3 Hz, 2H), 1.91 (t, J = 1.3 Hz, 2H).
^{13}C-NMR {^{1}H} (176 MHz, Acetone-d$_6$): δ = 196.4, 162.2 (d, $^1J_{CF}$ = 246.7 Hz), 138.2, 132.0, 128.8 (d, $^4J_{CF}$ = 3.3 Hz), 128.4, 128.1, 127.7 (d, $^3J_{CF}$ = 8.2 Hz), 115.5 (d, $^2J_{CF}$ = 21.8 Hz), 37.9, 30.9, 21.5.
^{19}F-NMR (659 MHz, Acetone-d$_6$): δ = -116.72 (td, J = 8.9, 4.8 Hz)
IR (ATR): $\tilde{\nu}$ (cm^{-1}) = 3050, 2957, 2948, 1624, 1598, 1575, 1526, 1500, 1483, 1447, 1402, 1345, 1224, 1205, 1163, 1136, 1062.
HRMS (APCI+) m/z: $[M+H]^+$ Calcd for $C_{17}H_{14}FO$ 253.1023; Found: 253.1020.
m.p.: 77 - 79 °C.

Phenyl(3-(4-(trifluoromethyl)phenyl)bicyclo[1.1.0]butan-1-yl)methanone (1c)

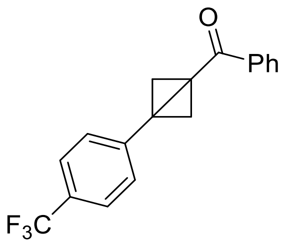

1c

BCB **1c** was synthesized according to the described procedure (Path 1). Column chromatography (SiO_2 (washed with 1% solution of Et_3N in Toluene), gradient

Toluene:EtOAc from 0% to 10% EtOAc) afforded the title compound **1c** (0.47 g, 1.55 mmol, 18% from **S1**) as a pale yellow solid.

^{1}H-NMR (500 MHz, $CDCl_3$): δ = 7.59 – 7.55 (m, 2H), 7.52 – 7.45 (m, 3H), 7.37 (m, 2H), 7.25 – 7.19 (m, 2H), 3.18 (t, *J* = 1.3 Hz, 2H), 1.96 (t, *J* = 1.3 Hz, 2H).

^{13}C-NMR {^{1}H} (126 MHz, $CDCl_3$): δ = 195.9, 137.9, 137.5, 132.3, 129.1 (q, *J* = 32.7 Hz), 128.5, 128.2, 126.3, 125.4 (q, *J* = 3.8 Hz), 124.2 (q, *J* = 271.9 Hz). 77.3, 77.3, 77.1, 76.8, 37.9, 36.7, 31.8.

^{19}F-NMR (471 MHz, $CDCl_3$): δ = –62.53 (s).

IR (ATR): $\tilde{\nu}$ (cm^{-1}) = 3058, 2984, 1633, 1615, 1599, 1577, 1406, 1346, 1321, 1212, 1165, 1113, 1097, 1060.

HRMS (APCI+) m/z: $[M+H]^+$ Calcd for $C_{18}H_{14}OF_3$ 303.0991; Found: 303.0994.

m.p.: 83 - 85 °C.

(3-(Naphthalen-2-yl)bicyclo[1.1.0]butan-1-yl)(phenyl)methanone (1e)

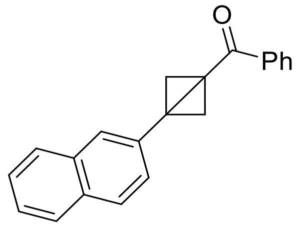

1e

BCB **1e** was synthesized according to the described procedure (Path 1). Column chromatography (SiO_2 (washed with 1% solution of Et_3N in Toluene), gradient Toluene:EtOAc from 0% to 10% EtOAc) afforded the title compound **1e** (0.38 g, 1.36 mmol, 16% from **S1**) as a pale yellow solid.

^{1}H-NMR (400 MHz, $CDCl_3$): δ = 7.81 – 7.63 (m, 4H), 7.58 – 7.50 (m, 2H), 7.48 – 7.37 (m, 3H), 7.35 – 7.27 (m, 2H), 7.20 – 7.13 (m, 1H), 3.28 (s, 2H), 1.98 (s, 2H).

^{13}C-NMR {^{1}H} (101 MHz, $CDCl_3$): δ = 196.3, 138.4, 133.3, 132.6, 131.9, 130.6, 128.4, 128.1, 128.1, 127.7, 127.7, 126.3, 125.9, 125.9, 123.4, 39.0, 38.0, 31.6.

IR (ATR): $\tilde{\nu}$ (cm^{-1}) = 3054, 2952, 1618, 1598, 1575, 1519, 1499, 1447, 1406, 1384, 1364, 1354, 1336, 1316, 1236, 1205, 1128, 1062.

HRMS (ESI+) m/z: $[M+Na]^+$ Calcd for $C_{21}H_{16}ONa$ 307.1093; Found: 307.1096.

m.p.: 116 - 118 °C.

(3-Butylbicyclo[1.1.0]butan-1-yl)(phenyl)methanone (1f)

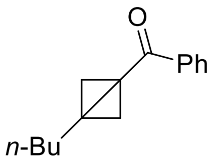

1f

BCB **1f** was synthesized according to the described procedure (Path 1). Column chromatography (SiO_2 (washed with 1% solution of Et_3N in Toluene), gradient Toluene:EtOAc from 0% to 10% EtOAc) afforded the title compound **1f** (0.65 g, 3.03 mmol, 35% from **S1**) as a pale yellow oil.

^{1}H-NMR (500 MHz, $CDCl_3$): δ = 7.92 – 7.76 (m, 2H), 7.54 – 7.48 (m, 1H), 7.45 – 7.38 (m, 2H), 2.50 – 2.43 (m, 2H), 1.78 – 1.69 (m, 2H), 1.52 (t, J = 1.0 Hz, 2H), 1.35 – 1.25 (m, 4H), 0.87 – 0.80 (m, 3H).
^{13}C-NMR {^{1}H} (126 MHz, $CDCl_3$): δ = 198.9, 138.7, 131.9, 128.6, 128.1, 39.8, 37.8, 30.3, 26.9, 22.4, 21.5, 13.9.
IR (ATR): $\tilde{\nu}$ (cm^{-1}) = 2959, 2930, 2872, 2860, 1634, 1598, 1576, 1448, 1404, 1342, 1210, 1173, 1101, 1072, 1002, 982.
HRMS (ESI+) m/z: $[M+H]^+$ Calcd for $C_{15}H_{19}O$ 215.1430; Found: 215.1429.

(1-Methyl-1H-indol-5-yl)(3-phenylbicyclo[1.1.0]butan-1-yl)methanone (1l)

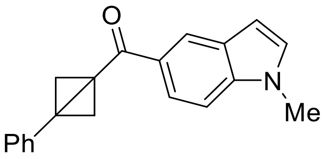

1l

BCB **1l** was synthesized according to the described procedure (Path 2). Column chromatography (SiO_2 (washed with 1% solution of Et_3N in Toluene), gradient from Toluene:CH_2Cl_2 from 10% to 50% CH_2Cl_2) afforded the title compound **1l** (0.43 g, 1.50 mmol, 17% from **S1**) as a pale yellow solid.

^{1}H-NMR (700 MHz, Acetone-d_6): δ = 8.05 (d, J = 1.6 Hz, 1H), 7.52 (dd, J = 8.6, 1.6 Hz, 1H), 7.28 – 7.03 (m, 8H), 6.55 (dd, J = 3.2, 0.8 Hz, 1H), 3.77 (s, 3H), 3.21 (t, J = 1.2 Hz, 2H), 1.90 (t, J = 1.2 Hz, 2H).

^{13}C-NMR {^{1}H} (176 MHz, Acetone-d_6): δ = 194.9, 139.6, 134.8, 131.5, 131.0, 128.9, 128.5, 127.4, 126.9, 123.7, 122.5, 109.7, 103.1, 37.6, 36.1, 33.0, 30.7.

IR (ATR): $\tilde{\nu}$ (cm^{-1}) = 3059, 2937, 1623, 1588, 1516, 1447, 1397, 1342, 1331, 1287, 1245, 1233, 1146, 1134, 1104, 1094, 1083, 1060, 1013, 1001, 986.

HRMS (ESI+) m/z: $[M+H]^+$ Calcd for $C_{20}H_{18}NO$ 288.1383; Found: 288.1379.

m.p.: 144 - 146 °C.

3.2 Synthesis of Thioketones

Thioketones **2a–l** were synthesized based on the previously reported procedures, and the analytical data of known compounds were in agreement with those reported in the literature.[4,5]

4. Optimization of the reaction conditions

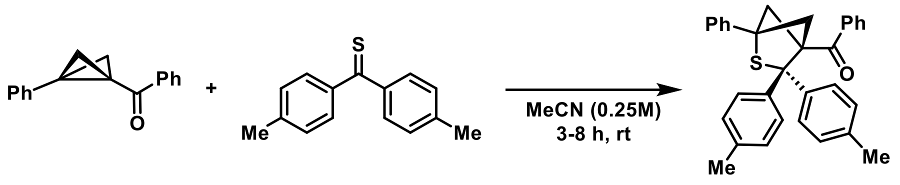

1a 2a 3aa

Entry №	Variation form the standard reaction conditions[a]	NMR yield[b]
1	none	95%
2	THF as a solvent	traces
3	Toluene as a solvent	27%
4	DCM as a solvent	60%
5	DMF as a solvent	traces
6	addition of MS 3 Å	86%
7	MeCN[c]	93%
8	+10 μL H_2O	91%
9	no light access	93%
10	1 h	56%
11	1.5 eq. of **2a**	68%

[a]BCB (100 μmol), thioketone (250 μmol), MeCN (4 mL), rt, 3 h. [b]Mesitylene (0.1 mmol) was used as an internal standard. [c]Ar was bubbled through the solvent for 10 min prior to the reaction.

5. Products from reactions of BCBs with thioketones

Phenyl(1-phenyl-3,3-di-*p*-tolyl-2-thiabicyclo[2.1.1]hexan-4-yl)methanone (3aa)

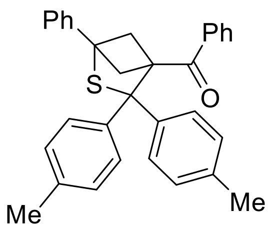

3aa

BCB **1a** (23.4 mg, 100 µmol, 1.0 eq.) and thiobenzophenone **2a** (56.6 mg, 250 µmol, 2.5 eq.) were reacted in MeCN (4.0 mL) for 3 h according to **GP**. The solid residue was washed with 1 mL acetonitrile three times resulting in the title compound **3aa** (38.5 mg, 84 µmol, 84%) as a white solid.

^{1}H-NMR (500 MHz, $CDCl_3$): δ = 7.42 – 7.36 (m, 6H), 7.35 – 7.30 (m, 2H), 7.28 – 7.24 (m, 2H), 7.06 – 7.03 (m, 2H), 7.01 – 6.96 (m, 2H), 6.93 – 6.90 (m, 4H), 3.49 – 3.44 (m, 2H), 2.92 – 2.86 (m, 2H), 2.25 (s, 6H).

^{13}C-NMR {^{1}H} (126 MHz, $CDCl_3$): δ = 202.1, 143.2, 138.1, 138.1, 136.1, 131.8, 129.6, 128.7, 128.5, 128.3, 127.7, 127.6, 126.3, 71.4, 64.6, 59.1, 52.6, 20.9.

IR (ATR): $\tilde{\nu}$ (cm^{-1}) = 3026, 2999, 2951, 2915, 1657, 1596, 1514, 1505, 1445, 1319, 1281, 1219, 1179, 1105, 1023.

HRMS (ESI+) m/z: $[M+H]^+$ Calcd for $C_{32}H_{29}OS$ 461.1934; Found: 461.1931.

m.p.: 217 - 219 °C.

(1-(4-Fluorophenyl)-3,3-di-*p*-tolyl-2-thiabicyclo[2.1.1]hexan-4-yl)(phenyl)methanone (3ba)

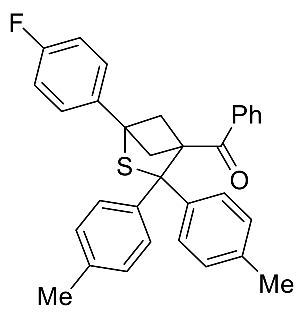

3ba

BCB **1b** (25.2 mg, 100 µmol, 1.0 eq.) and thiobenzophenone **2a** (56.6 mg, 250 µmol, 2.5 eq.) were reacted in MeCN (4.0 mL) for 3 h according to **GP**. The solid residue was washed with 1 mL acetonitrile three times resulting in the title compound **3ba** (30.2 mg, 63 µmol, 63%) as a white solid.

^{1}H-NMR (500 MHz, $CDCl_3$): δ = 7.39 – 7.34 (m, 6H), 7.28 – 7.24 (m, 1H), 7.05 – 6.96 (m, 7H), 6.93 – 6.89 (m, 4H), 3.48 – 3.41 (m, 2H), 2.89 – 2.82 (m, 2H), 2.25 (s, 6H).

^{13}C-NMR {^{1}H} (126 MHz, $CDCl_3$): δ = 201.9, 162.2 (d, J = 246.4 Hz), 143.0, 138.0, 136.2, 131.8, 134.0 (d, J = 3.2 Hz), 129.6, 129.6, 128.7, 128.7, 128.3, 128.3, 128.09 (d, J = 8.1 Hz), 127.6, 115.5 (d, J = 21.6 Hz), 71.6, 64.5, 58.4, 52.6, 20.9.

^{19}F-NMR (282 MHz, $CDCl_3$) δ = -114.15 (m).

IR (ATR): $\tilde{\nu}$ (cm^{-1}) = 3050, 2957, 1624, 1598, 1575, 1526, 1500, 1483, 1447, 1402, 1345, 1224, 1205, 1176, 1163, 1136, 1062.

HRMS (APCI+) m/z: $[M+H]^+$ Calcd for $C_{32}H_{28}FOS$ 479.1839; Found: 479.1837.

m.p.: 206 - 208 °C.

(3,3-Di-*p*-tolyl-1-(4-(trifluoromethyl)phenyl)-2-thiabicyclo[2.1.1]hexan-4-yl)(phenyl)methanone (3ca)

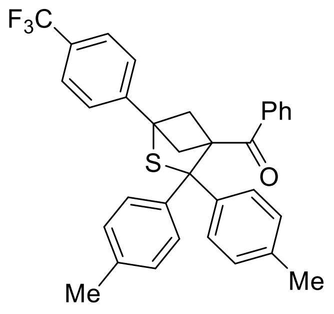

3ca

BCB **1c** (30.2 mg, 100 µmol, 1.0 eq.) and thiobenzophenone **2a** (56.6 mg, 250 µmol, 2.5 eq.) were reacted in MeCN (4.0 mL) for 3 h according to **GP**. The solid residue was washed with 1 mL acetonitrile three times resulting in the title compound **3ca** (50.7 mg, 96 µmol, 96%) as a white solid.

^{1}H-NMR (500 MHz, $CDCl_3$): δ = 7.61 – 7.57 (m, 2H), 7.53 – 7.49 (m, 2H), 7.39 – 7.35 (m, 4H), 7.27 (tt, J = 7.2, 1.4 Hz, 1H), 7.05 – 7.02 (m, 2H), 7.01 – 6.97 (m, 2H), 6.94 – 6.90 (m, 4H), 3.52 – 3.45 (m, 2H), 2.93 – 2.86 (m, 2H), 2.25 (s, 6H).

^{13}C-NMR {^{1}H} (126 MHz, $CDCl_3$): δ = 201.6, 142.9, 142.2, 137.9, 136.3, 131.9, 129.9 (q, *J* = 32.4 Hz), 129.6, 128.7, 128.4, 127.6, 126.8, 125.6 (q, *J* = 3.8 Hz), 124.1 (q, *J* = 272.1 Hz), 71.7, 64.6, 58.3, 52.5, 20.9.

^{19}F-NMR (471 MHz, $CDCl_3$): δ = –62.53 (s).

IR (ATR): $\tilde{\nu}$ (cm^{-1}) = 3019, 2992, 2922, 2867, 1657, 1509, 1408, 1316, 1276, 1218, 1184, 1162, 1129, 1111, 1065, 1017.

HRMS (ESI+) m/z: $[M+Na]^+$ Calcd for $C_{33}H_{27}F_3ONaS$ 551.1627; Found: 551.1624.

m.p.: 178 - 180 °C.

(3,3-Di-*p*-tolyl-1-(4-(trifluoromethoxy)phenyl)-2-thiabicyclo[2.1.1]hexan-4-yl)(phenyl)methanone (3da)

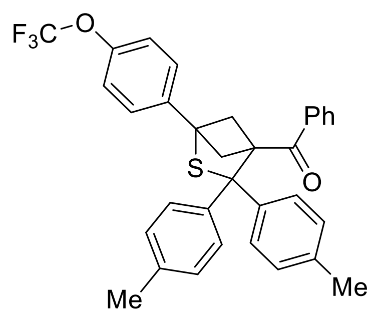

3da

BCB **1d** (31.8 mg, 100 µmol, 1.0 eq.) and thiobenzophenone **2a** (56.6 mg, 250 µmol, 2.5 eq.) were reacted in MeCN (4.0 mL) for 3 h according to **GP**. The solid residue was washed with 1 mL acetonitrile three times resulting in the title compound **3da** (40.0 mg, 73 µmol, 73%) as a white solid.

^{1}H-NMR (500 MHz, $CDCl_3$): δ = 7.44 – 7.40 (m, 2H), 7.39 – 7.35 (m, 4H), 7.29 – 7.25 (m, 1H), 7.19 – 7.16 (m, 2H), 7.03 (dd, *J* = 8.5, 1.5 Hz, 2H), 6.99 (dd, *J* = 8.4, 7.2 Hz, 2H), 6.94 – 6.90 (m, 4H), 3.49 – 3.42 (m, 2H), 2.90 – 2.84 (m, 2H), 2.25 (s, 6H).

^{13}C-NMR {^{1}H} (126 MHz, $CDCl_3$): δ = 201.7, 148.7 (q, *J* = 2.0 Hz), 148.6, 142.9, 138.0, 136.9, 136.3, 131.9, 129.6, 128.7, 128.4, 127.9, 127.6, 121.1, 120.5 (q, *J* = 257.3 Hz), 71.7, 64.5, 58.2, 52.6, 20.9.

^{19}F-NMR (471 MHz, $CDCl_3$): δ = -57.84 (s).

IR (ATR): $\tilde{\nu}$ (cm^{-1}) = 3016, 2995, 2923, 1655, 1595, 1509, 1445, 1252, 1216, 1205, 1160, 1123, 1109, 1017.

HRMS (ESI+) m/z: $[M+Na]^+$ Calcd for $C_{33}H_{27}F_3O_2NaS$ 567.1576; Found: 567.1569.

m.p.: 166 - 168 °C.

(1-(Naphthalen-2-yl)-3,3-di-*p*-tolyl-2-thiabicyclo[2.1.1]hexan-4-yl)(phenyl)methanone (3ea)

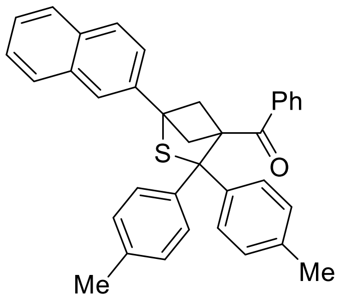

3ea

BCB **1e** (28.4 mg, 100 µmol, 1.0 eq.) and thiobenzophenone **2a** (56.6 mg, 250 µmol, 2.5 eq.) were reacted in MeCN (4.0 mL) for 3 h according to **GP**. The solid residue was washed with 1 mL acetonitrile three times resulting in the title compound **3ea** (45.0 mg, 88 µmol, 88%) as a white solid.

^{1}H-NMR (500 MHz, $CDCl_3$): δ = 7.83 – 7.76 (m, 4H), 7.55 (dd, *J* = 8.4, 1.6 Hz, 1H), 7.49 – 7.43 (m, 2H), 7.43 – 7.39 (m, 4H), 7.27 (tt, *J* = 7.3, 1.6 Hz, 1H), 7.07 (dd, *J* = 8.4, 1.6 Hz, 2H), 7.00 (dd, *J* = 8.4, 7.2 Hz, 2H), 6.95 – 6.91 (m, 4H), 3.59 – 3.54 (m, 2H), 3.00 – 2.94 (m, 2H), 2.26 (s, 6H).

^{13}C-NMR {^{1}H} (126 MHz, $CDCl_3$): δ = 202.0, 143.2, 138.1, 136.2, 135.6, 133.3, 132.8, 131.8, 129.7, 128.7, 128.4, 128.3, 127.9, 127.1, 127.6, 126.4, 126.1, 125.2, 124.2, 71.6, 64.6, 59.4, 52.7, 20.9.

IR (ATR): $\tilde{\nu}$ (cm^{-1}) = 3053, 3020, 2917, 2854, 1655, 1598, 1506, 1443, 1312, 1278, 1239, 1215, 1181, 1133, 1117, 1105.

HRMS (APCI+) m/z: $[M+H]^+$ Calcd for $C_{36}H_{31}OS$ 511.2096; Found: 511.2087.

m.p.: 256 - 258 °C.

(1-Butyl-3,3-di-*p*-tolyl-2-thiabicyclo[2.1.1]hexan-4-yl)(phenyl)methanone (3fa)

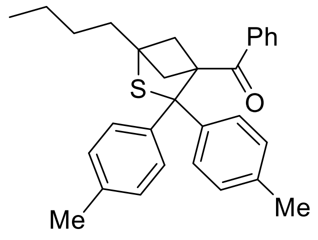

3fa

BCB **1f** (21.4 mg, 100 µmol, 1.0 eq.) and thiobenzophenone **2a** (56.6 mg, 250 µmol, 2.5 eq.) were reacted in MeCN (4.0 mL) for 3 h according to **GP**. Filtration through a pad of silica gel in toluene with a following HPLC (Hexane:EtOAc 20:1, Macherey-Nagel VP 250/21 NUCLEODUR 100-5 silica column) **3fa** (19.4 mg, 44 µmol, 44%) as a yellow oil.

^{1}H-NMR (700 MHz, $CDCl_3$): δ = 7.34 – 7.30 (m, 4H), 7.25 – 7.21 (m, 1H), 7.01 – 6.93 (m, 4H), 6.89 – 6.85 (m, 4H), 3.01 (dd, *J* = 5.7, 2.3 Hz, 2H), 2.49 (dd, *J* = 5.7, 2.3 Hz, 2H), 2.23 (s, 6H), 1.91 – 1.79 (m, 2H), 1.47 – 1.39 (m, 2H), 1.35 (dt, *J* = 7.9, 7.3 Hz, 2H), 0.90 (t, *J* = 7.3 Hz, 3H).

^{13}C-NMR {^{1}H} (176 MHz, $CDCl_3$): δ = 202.6, 143.4, 138.2, 135.9, 131.6, 129.6, 128.6, 128.2, 127.5, 70.2, 64.8, 59.7, 52.0, 33.0, 28.8, 23.0, 20.9, 14.0.

IR (ATR): $\tilde{\nu}$ (cm^{-1}) = 3024, 2997, 2956, 2923, 1659, 1598, 1506, 1401, 1378, 1288, 1184, 1184, 1040, 1021.

HRMS (ESI+) m/z: $[M+O+Na]^+$ Calcd for $C_{30}H_{32}O_2NaS$ 479.2015; Found: 479.2013.

m.p.: 185 - 187 °C.

(1-Phenyl-3,3-di-*p*-tolyl-2-thiabicyclo[2.1.1]hexan-4-yl)(p-tolyl)methanone (3ga)

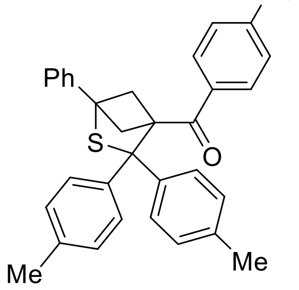

3ga

BCB **1g** (24.8 mg, 100 µmol, 1.0 eq.) and thiobenzophenone **2a** (56.6 mg, 250 µmol, 2.5 eq.) were reacted in MeCN (4.0 mL) for 3 h according to **GP**. The solid residue was washed with 1 mL acetonitrile two times resulting in the title compound **3ga** (35.0 mg, 73 µmol, 73%) as a white solid.

^{1}H-NMR (500 MHz, $CDCl_3$): δ = 7.41 – 7.37 (m, 6H), 7.34 – 7.29 (m, 2H), 7.27 – 7.23 (m, 1H), 6.95 – 6.90 (m, 6H), 6.81 – 6.77 (m, 2H), 3.48 – 3.42 (m, 2H), 2.89 – 2.83 (m, 2H), 2.25 (s, 6H).

^{13}C-NMR {^{1}H} (126 MHz, $CDCl_3$): δ = 201.6, 143.3, 142.7, 138.2, 136.1, 135.6, 129.8, 128.8, 128.5, 128.3, 128.2, 127.7, 126.3, 71.5, 64.5, 59.0, 52.6, 21.5, 20.9.

IR (ATR): $\tilde{\nu}$ (cm^{-1}) = 3024, 2949, 2921, 1651, 1604, 1504, 1445, 1319, 1279, 1218, 1180, 1109, 1029, 1021.

HRMS (ESI+) m/z: $[M+Na]^+$ Calcd for $C_{33}H_{30}ONaS$ 497.1910; Found: 497.1902.

m.p.: 224 - 226 °C.

(4-Chlorophenyl)(1-phenyl-3,3-di-*p*-tolyl-2-thiabicyclo[2.1.1]hexan-4-yl)methanone (3ha)

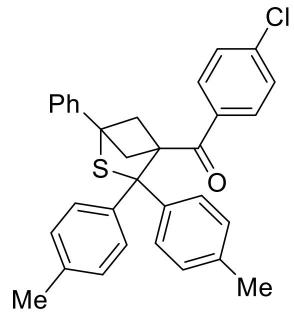

3ha

BCB **1h** (26.9 mg, 100 µmol, 1.0 eq.) and thiobenzophenone **2a** (56.6 mg, 250 µmol, 2.5 eq.) were reacted in MeCN (4.0 mL) for 3 h according to **GP**. The solid residue was washed with 1 mL acetonitrile two times resulting in the title compound **3ha** (43.0 mg, 87 µmol, 87%) as a white solid.

^{1}H-NMR (500 MHz, $CDCl_3$): δ = 7.41 – 7.30 (m, 8H), 7.29 – 7.24 (m, 1H), 6.95 (s, 4H), 6.94 – 6.90 (m, 4H), 3.48 – 3.41 (m, 2H), 2.90 – 2.83 (m, 2H), 2.27 (s, 6H).

^{13}C-NMR {^{1}H} (126 MHz, $CDCl_3$): δ = 200.9, 143.0, 138.4, 138.0, 136.5, 136.4, 130.0, 129.6, 128.6, 128.4, 127.8, 127.8, 126.3, 71.2, 64.6, 59.2, 52.5, 20.9.

IR (ATR): $\tilde{\nu}$ (cm^{-1}) = 3027, 2950, 2921, 2854, 1656, 1587, 1505, 1446, 1321, 1277, 1220, 1185, 1174, 1090, 1013.

HRMS (ESI+) m/z: $[M+H]^+$ Calcd for $C_{32}H_{27}ClNaOS$ 517.1363; Found: 517.1367.
m.p.: 194 - 196 °C.

(1-Phenyl-3,3-di-*p*-tolyl-2-thiabicyclo[2.1.1]hexan-4-yl)(4-(trifluoromethyl)phenyl)methanone (3ia)

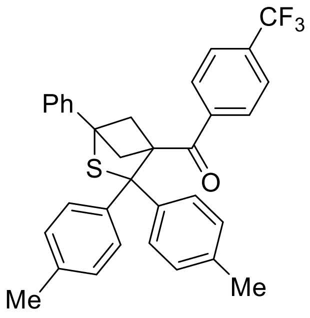

3ia

BCB **1i** (30.2 mg, 100 µmol, 1.0 eq.) and thiobenzophenone **2a** (56.6 mg, 250 µmol, 2.5 eq.) were reacted in MeCN (4.0 mL) for 3 h according to **GP**. The solid residue was washed with 1 mL acetonitrile two times resulting in the title compound **3ia** (44.0 mg, 83 µmol, 83%) as a white solid.

^{1}H-NMR (500 MHz, $CDCl_3$): δ = 7.43 – 7.39 (m, 2H), 7.36 – 7.31 (m, 2H), 7.30 – 7.26 (m, 5H), 7.23 – 7.21 (m, 2H), 7.13 – 7.10 (m, 2H), 6.90 – 6.86 (m, 4H), 3.51 – 3.45 (m, 2H), 2.96 – 2.91 (m, 2H), 2.24 (s, 6H).

^{13}C-NMR {^{1}H} (126 MHz, $CDCl_3$): δ = 201.9, 142.8, 141.4, 137.8, 136.5, 132.9 (q, *J* = 32.6 Hz), 129.5, 128.6, 128.5, 128.3, 127.9, 126.3, 124.5 (q, *J* = 3.8 Hz), 123.6 (q, *J* = 272.6 Hz), 71.0, 64.9, 59.3, 52.2, 20.8.

^{19}F-NMR (471 MHz, $CDCl_3$): δ = -63.13.

IR (ATR): $\tilde{\nu}$ (cm^{-1}) = 3026, 2922, 2853, 1666, 1506, 1405, 1322, 1278, 1170, 1128, 1113, 1065, 1016.

HRMS (ESI+) m/z: $[M+O+Na]^+$ Calcd for $C_{33}H_{27}F_3O_2NaS$ 567.1576; Found: 567.1575.
m.p.: 197 - 199 °C.

Naphthalen-2-yl(1-phenyl-3,3-di-*p*-tolyl-2-thiabicyclo[2.1.1]hexan-4-yl)methanone (3ja)

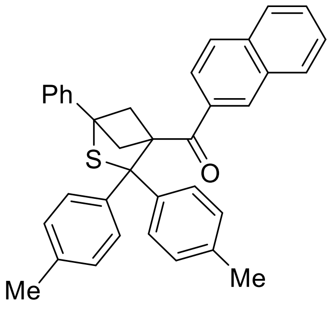

3ja

BCB **1j** (28.4 mg, 100 µmol, 1.0 eq.) and thiobenzophenone **2a** (56.6 mg, 250 µmol, 2.5 eq.) were reacted in MeCN (4.0 mL) for 3 h according to **GP**. The solid residue was washed with 1 mL acetonitrile two times resulting in the title compound **3ja** (47.0 mg, 92 µmol, 92%) as a white solid.

^{1}H-NMR (500 MHz, $CDCl_3$): δ = 7.72 – 7.69 (m, 1H), 7.59 – 7.54 (m, 2H), 7.50 – 7.36 (m, 9H), 7.35 – 7.30 (m, 2H), 7.29 – 7.24 (m, 1H), 7.08 – 7.05 (m, 1H), 6.90 – 6.83 (m, 4H), 3.61 – 3.50 (m, 2H), 2.98 – 2.88 (m, 2H), 2.13 (s, 6H).

^{13}C-NMR {^{1}H} (126 MHz, $CDCl_3$): δ = 201.6, 143.3, 138.2, 136.3, 134.9, 131.9, 131.1, 129.9, 129.8, 128.5, 128.4, 128.2, 127.7, 127.4, 127.4, 126.4, 12.1, 124.5, 71.5, 64.8, 59.2, 52.9, 20.8.

IR (ATR): $\tilde{\nu}$ (cm^{-1}) = 3048, 3029, 2997, 2987, 2919, 2856, 1654, 1626, 1506, 1463, 1448, 1319, 1283, 1216, 1193, 1180, 1134, 1106, 1022.

HRMS (ESI+) m/z: $[M+Na]^+$ Calcd for $C_{36}H_{30}ONaS$ 533.1910; Found: 533.1909.

m.p.: 222 - 224 °C.

1-(1-Phenyl-3,3-di-*p*-tolyl-2-thiabicyclo[2.1.1]hexan-4-yl)pentan-1-one (3ka)

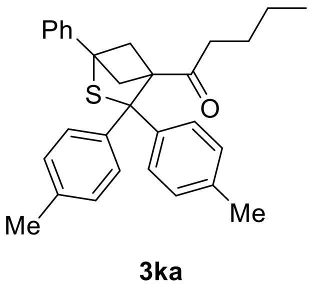

3ka

BCB **1k** (21.4 mg, 100 µmol, 1.0 eq.) and thiobenzophenone **2a** (56.6 mg, 250 µmol, 2.5 eq.) were reacted in MeCN (4.0 mL) for 3 h according to **GP**. Filtration through a pad of silica gel in toluene with a following gel permeation chromatography (JAIGEL-2H, chloroform) afforded the title compound **3ka** (33.0 mg, 75 µmol, 75%) as a yellow oil.

^{1}H-NMR (500 MHz, $CDCl_3$): δ = 7.45 – 7.42 (m, 4H), 7.38 – 7.35 (m, 2H), 7.34 – 7.30 (m, 2H), 7.28 – 7.24 (m, 1H), 7.10 – 7.06 (m, 4H), 3.23 – 3.16 (m, 2H), 2.62 – 2.55 (m, 2H), 2.32 (s, 6H), 1.65 (dd, *J* = 8.0, 7.0 Hz, 2H), 1.34 (tt, *J* = 8.5, 7.0 Hz, 2H), 1.07 – 0.97 (m, 2H), 0.72 (t, *J* = 7.4 Hz, 3H).

^{13}C-NMR {^{1}H} (126 MHz, $CDCl_3$): δ = 211.7, 143.1, 138.1, 136.3, 129.5, 128.5, 127.7, 126.3, 71.0, 64.9, 58.1, 50.6, 40.6, 25.6, 22.1, 21.0, 13.8.

IR (ATR): $\tilde{\nu}$ (cm^{-1}) = 3025, 2956, 2927, 2870, 1738, 1698, 1507, 1446, 1372, 1239, 1176, 1163, 1045, 1022.

HRMS (ESI+) m/z: $[M+Na]^+$ Calcd for $C_{30}H_{32}NaOS$ 463.2066; Found: 463.2073.

(1-Methyl-1H-indol-5-yl)(1-phenyl-3,3-di-*p*-tolyl-2-thiabicyclo[2.1.1]hexan-4-yl)methanone (3la)

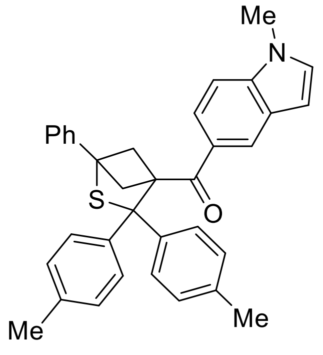

3la

BCB **1l** (28.7 mg, 100 µmol, 1.0 eq.) and thiobenzophenone **2a** (56.6 mg, 250 µmol, 2.5 eq.) were reacted in MeCN (4.0 mL) for 3 h according to **GP**. The solid residue was washed with 1 mL acetonitrile two times resulting in the title compound **3la** (29.0 mg, 57 µmol, 57%) as a white solid.

^{1}H-NMR (500 MHz, $CDCl_3$): δ = 7.69 (dd, *J* = 8.2, 1.1 Hz, 1H), 7.57 – 7.51 (m, 5H), 7.48 (ddd, *J* = 8.2, 4.7, 3.3 Hz, 1H), 7.44 (dd, *J* = 8.7, 1.8 Hz, 1H), 7.39 – 7.37 (m, 2H), 7.11 – 6.99 (m, 7H), 3.68 (t, *J* = 3.4 Hz, 1H), 3.04 – 2.97 (m, 2H), 2.88 – 2.81 (m, 2H).

^{13}C-NMR {^{1}H} (126 MHz, $CDCl_3$): δ = 202.0, 146.4, 134.9, 134.7, 131.9, 131.2, 130.0, 130.0, 128.3, 127.7, 127.4, 127.3, 126.5, 126.1, 124.5, 68.6, 67.8, 50.0, 44.2.

IR (ATR): $\tilde{\nu}$ (cm^{-1}) = 3027, 2995, 2945, 2918, 2870, 1642, 1607, 1513, 1446, 1345, 1323, 1303, 1269, 1248, 1189, 1174, 1150, 1087.

HRMS (ESI+) m/z: $[M+Na]^+$ Calcd for $C_{35}H_{31}ONNaS$ 536.2019; Found: 536.2014.

m.p.: 258 - 260 °C.

Phenyl(1,3,3-triphenyl-2-thiabicyclo[2.1.1]hexan-4-yl)methanone (3ab)

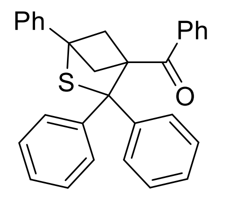

3ab

BCB **1a** (23.4 mg, 100 µmol, 1.0 eq.) and thiobenzophenone **2b** (49.6 mg, 250 µmol, 2.5 eq.) were reacted in MeCN (4.0 mL) for 3 h according to **GP**. The solid residue was washed with 1 mL acetonitrile three times resulting in the title compound **3ab** (33.3 mg, 77 µmol, 77%) as a white solid.

^{1}H-NMR (500 MHz, $CDCl_3$): δ = 7.54 – 7.50 (m, 4H), 7.43 – 7.39 (m, 2H), 7.36 – 7.31 (m, 2H), 7.29 – 7.24 (m, 2H), 7.15 – 7.09 (m, 6H), 7.06 – 6.98 (m, 4H), 3.53 – 3.42 (m, 2H), 2.99 – 2.86 (m, 2H).

^{13}C-NMR {^{1}H} (126 MHz, $CDCl_3$): δ = 201.8, 146.0, 138.0, 137.9, 132.1, 129.8, 128.7, 128.6, 127.8, 127.7, 126.5, 126.3, 71.8, 64.4, 59.3, 52.7.

IR (ATR): $\tilde{\nu}$ (cm^{-1}) = 3078, 3055, 3027, 3005, 1656, 1596, 1576, 1490, 1447, 1440, 1320, 1278, 1219, 1185, 1175, 1109, 1076, 1027.

HRMS (ESI+) m/z: $[M+Na]^+$ Calcd for $C_{30}H_{24}ONaS$ 455.1440; Found: 455.1438.

m.p.: 213 - 215 °C.

(3,3-bis(4-fluorophenyl)-1-phenyl-2-thiabicyclo[2.1.1]hexan-4-yl)(phenyl)methanone (3ac)

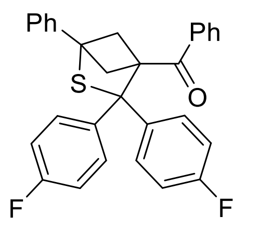

3ac

BCB **1a** (23.4 mg, 100 µmol, 1.0 eq.) and thiobenzophenone **2c** (58.6 mg, 250 µmol, 2.5 eq.) were reacted in MeCN (4.0 mL) for 3 h according to **GP**. The solid residue was washed with 1 mL acetonitrile three times resulting in the title compound **3ac** (41.0 mg, 81 µmol, 81%) as a white solid.

^{1}H-NMR (500 MHz, $CDCl_3$): δ = 7.48 – 7.43 (m, 4H), 7.43 – 7.38 (m, 2H), 7.38 – 7.26 (m, 4H), 7.10 – 7.04 (m, 4H), 6.83 – 6.77 (m, 4H), 3.49 – 3.40 (m, 2H), 3.00 – 2.88 (m, 2H).

^{13}C-NMR {^{1}H} (126 MHz, $CDCl_3$): δ = 201.6, 161.4 (d, *J* = 247.0 Hz), 141.6 (d, *J* = 3.6 Hz), 137.9, 137.6, 132.4, 131.5 (d, *J* = 8.0 Hz), 128.7, 128.6, 128.0, 127.9, 126.3, 114.5 (d, *J* = 21.2 Hz), 70.5, 64.6, 59.6, 52.6.

^{19}F-NMR (471 MHz, $CDCl_3$): δ = -115.91 – -116.00 (m).

IR (ATR): $\tilde{\nu}$ (cm^{-1}) = 3057, 2995, 2970, 1655, 1597, 1578, 150, 1446, 1318, 1275, 1220, 1158, 1107, 1099, 1091, 1013.

HRMS (ESI+) m/z: $[M+H]^+$ Calcd for $C_{30}H_{23}F_2O_4S$ 469.1432; Found: 469.1430.

m.p.: 224 - 226 °C.

(3,3-Bis(4-methoxyphenyl)-1-phenyl-2-thiabicyclo[2.1.1]hexan-4-yl)(phenyl)methanone (3ad)

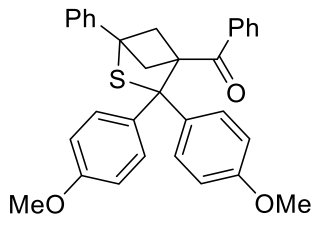

3ad

BCB **1a** (23.4 mg, 100 µmol, 1.0 eq.) and thiobenzophenone **2d** (64.6 mg, 250 µmol, 2.5 eq.) were reacted in MeCN (4.0 mL) for 3 h according to **GP**. The solid residue was washed with 1 mL acetonitrile three times resulting in the title compound **3ad** (40.0 mg, 81 µmol, 81%) as a white solid.

^{1}H-NMR (500 MHz, $CDCl_3$): δ = 7.43 – 7.38 (m, 6H), 7.35 – 7.30 (m, 2H), 7.29 – 7.23 (m, 2H), 7.10 – 7.06 (m, 2H), 7.05 – 7.00 (m, 2H), 6.66 – 6.61 (m, 4H), 3.73 (s, 6H), 3.50 – 3.42 (m, 2H), 2.93 – 2.87 (m, 2H).

^{13}C-NMR {^{1}H} (126 MHz, $CDCl_3$): δ = 202.1, 158.1, 138.4, 138.1, 131.9, 130.9, 128.7, 128.5, 127.7, 127.7, 126.3, 112.9, 71.0, 64.7, 59.2, 55.3, 52.6.

IR (ATR): $\tilde{\nu}$ (cm^{-1}) = 2994, 2952, 2926, 2908, 2830, 1659, 1652, 1603, 1579, 1505, 1462, 1443, 1319, 1302, 1280, 1249, 1218, 1176, 1035.

HRMS (ESI+) m/z: $[M+H]^+$ Calcd for $C_{32}H_{29}O_3S$ 493.1832; Found: 493.1834.

m.p.: 137 - 139 °C.

Phenyl(4-phenyl-3-thiaspiro[bicyclo[2.1.1]hexane-2,9'-xanthen]-1-yl)methanone (3ae)

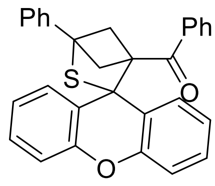

3ae

BCB **1a** (23.4 mg, 100 µmol, 1.0 eq.) and thiobenzophenone **2e** (53.0 mg, 250 µmol, 2.5 eq.) were reacted in MeCN (4.0 mL) for 3 h according to **GP**. The solid residue was washed with 1 mL acetonitrile three times resulting in the title compound **3ae** (31.0 mg, 82 µmol, 82%) as a white solid.

^{1}H-NMR (500 MHz, $CDCl_3$): δ = 8.38 – 8.33 (m, 2H), 7.55 – 7.50 (m, 2H), 7.44 – 7.39 (m, 2H), 7.37 – 7.29 (m, 2H), 7.28 – 7.23 (m, 4H), 7.10 – 7.03 (m, 4H), 6.89 – 6.84 (m, 2H), 3.37 – 3.26 (m, 2H), 2.55 – 2.41 (m, 2H).

^{13}C-NMR {^{1}H} (126 MHz, $CDCl_3$): δ = 197.4, 153.6, 137.8, 136.3, 132.4, 129.9, 129.0, 128.7, 128.5, 128.0, 127.7, 126.6, 125.3, 123.1, 116.5, 65.7, 63.2, 58.1, 50.0.

IR (ATR): $\tilde{\nu}$ (cm^{-1}) = 3064, 3029, 3000, 2924, 1664, 1591, 1465, 1443, 1323, 1302, 1277, 1238, 1217, 1096, 1033.

HRMS (ESI+) m/z: $[M+Na]^+$ Calcd for $C_{30}H_{22}O_2NaS$ 469.1233; Found: 469.1234.

m.p.: 250 - 252 °C.

Phenyl(4-phenyl-3-thiaspiro[bicyclo[2.1.1]hexane-2,9'-thioxanthen]-1-yl)methanone (3af)

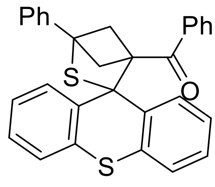

3af

BCB **1a** (23.4 mg, 100 µmol, 1.0 eq.) and thiobenzophenone **2f** (57.1 mg, 250 µmol, 2.5 eq.) were reacted in MeCN (4.0 mL) for 3 h according to **GP**. The solid residue was washed with 1 mL acetonitrile three times resulting in the title compound **3af** (32.5 mg, 70 µmol, 70%) as a white solid.

^{1}H-NMR (500 MHz, $CDCl_3$): δ = 8.77 (d, *J* = 8.1 Hz, 2H), 7.52 – 7.48 (m, 2H), 7.45 – 7.37 (m, 4H), 7.35 – 7.26 (m, 4H), 7.24 – 7.18 (m, 2H), 7.12 – 7.05 (m, 2H), 7.04 – 6.99 (m, 2H), 3.23 – 3.16 (m, 2H), 2.47 – 2.39 (m, 2H).

^{13}C-NMR {^{1}H} (126 MHz, $CDCl_3$): δ = 196.7, 137.9, 136.6, 134.7, 132.4, 132.2, 128.7, 128.5, 127.9, 127.8, 127.7, 127.0, 126.6, 126.2, 71.0, 63.9, 57.1, 51.0.

IR (ATR): $\tilde{\nu}$ (cm^{-1}) = 3061, 3054, 3029, 3001, 2923, 1663, 1593, 1578, 1448, 1433, 1320, 1267, 1216, 1162, 1040.

HRMS (ESI+) m/z: $[M+H]^+$ Calcd for $C_{30}H_{23}OS_2$ 463.1185; Found: 463.1188.

m.p.: 259 - 261 °C.

Phenyl(2',3',4-triphenyl-3-thiaspiro[bicyclo[2.1.1]hexane-2,1'-cyclopropan]-2'-en-1-yl)methanone (3ag)

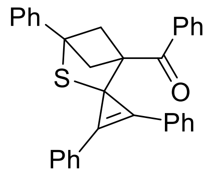

3ag

BCB **1a** (23.4 mg, 100 µmol, 1.0 eq.) and thiobenzophenone **2g** (55.6 mg, 250 µmol, 2.5 eq.) were reacted in MeCN (4.0 mL) for 8 h according to **GP**. Column chromatography (SiO_2, Toluene) afforded the title compound **3ag** (29.4 mg, 64 µmol, 64%) as a white solid.

^{1}H-NMR (500 MHz, $CDCl_3$): δ = 7.78 – 7.68 (m, 6H), 7.52 – 7.48 (m, 2H), 7.45 – 7.20 (m, 10H), 7.06 – 7.01 (m, 2H), 3.56 – 3.42 (m, 2H), 3.02 – 2.92 (m, 2H).

^{13}C-NMR {^{1}H} (126 MHz, $CDCl_3$): δ = 200.9, 139.5, 136.3, 132.7, 129.7, 129.4, 128.8, 128.6, 128.6, 128.0, 128.0, 127.6, 126.4, 120.1, 62.8, 58.3, 53.6, 52.0.

IR (ATR): $\tilde{\nu}$ (cm^{-1}) = 3058, 2923, 2853, 1662, 1597, 1580, 1493, 1446, 1323, 1289, 1223, 1177, 1070, 1025.

HRMS (ESI+) m/z: $[M+H]^+$ Calcd for $C_{32}H_{25}OS$ 457.1621; Found: 457.1619.

m.p.: 106 - 107 °C.

Phenyl(1,3,3-triphenyl-2-thiabicyclo[2.1.1]hexan-4-yl)methanone (3al)

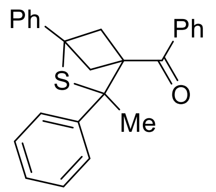

3al

BCB **1a** (150.0 mg, 640 µmol, 1.0 eq.) and thiobenzophenone **2l** (218.0 mg, 1.6 mmol, 2.5 eq.) were reacted in MeCN (20.0 mL) for 8 h according to **GP**. Column chromatography (SiO_2, Pentane:EtOAc 20:1) afforded the title compound **3al** (56.2 mg, 151 µmol, 24%) as a yellowish oil.

^{1}H-NMR (500 MHz, $CDCl_3$): δ = 7.70 – 7.66 (m, 2H), 7.43 – 7.32 (m, 5H), 7.30 – 7.25 (m, 1H), 7.23 – 7.16 (m, 7H), 3.32 (dd, J = 9.6, 8.4 Hz, 1H), 3.00 (dd, J = 9.6, 8.4 Hz, 1H), 2.71 (d, J = 8.4 Hz, 1H), 2.53 (d, J = 8.4 Hz, 1H), 2.19 (s, 3H).

^{13}C-NMR {^{1}H} (126 MHz, $CDCl_3$): δ = δ 201.19, 143.31, 138.45, 137.39, 132.65, 129.01, 128.98, 128.60, 128.04, 128.02, 127.74, 127.24, 126.39, 64.95, 63.54, 57.81, 53.44, 49.80, 28.75.

IR (ATR): $\tilde{\nu}$ (cm^{-1}) = 3083, 3058, 3028, 2991, 2933, 1658, 1597, 1579, 1492, 1446, 1380, 1321, 1278, 1219, 1179, 1094, 1074, 1027, 1009.

HRMS (APCI+) m/z: $[M+H]^+$ Calcd for $C_{25}H_{23}OS$ 371.1464; Found: 371.1466.

Phenyl(3-phenyl-3-((1-phenylvinyl)thio)cyclobutyl)methanone (3al')

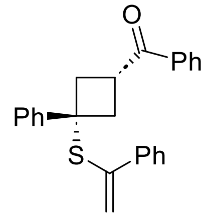

3al'

BCB **1a** (150.0 mg, 640 µmol, 1.0 eq.) and thiobenzophenone **2l** (218.0 mg, 1.6 mmol, 2.5 eq.) were reacted in MeCN (20.0 mL) for 8 h according to **GP**. Column chromatography (SiO_2, Pentane:EtOAc 20:1) afforded the title compound **3al'** (43.0 mg, 116 µmol, 18%) as a yellowish oil.

^{1}H-NMR (700 MHz, $CDCl_3$): δ = 7.83 – 7.80 (m, 2H), 7.55 – 7.52 (m, 1H), 7.44 – 7.41 (m, 2H), 7.37 – 7.33 (m, 4H), 7.32 – 7.28 (m, 2H), 7.23 – 7.19 (m, 4H), 5.45 (d, J = 0.7

Hz, 1H), 5.21 (d, J = 0.7 Hz, 1H), 3.73 (p, J = 8.8 Hz, 1H), 3.03 – 2.97 (m, 2H), 2.94 – 2.88 (m, 2H).

^{13}C-NMR {^{1}H} (176 MHz, $CDCl_3$): δ = 199.51, 144.33, 143.08, 140.53, 135.45, 133.17, 128.72, 128.44, 128.14, 128.13, 128.05, 127.62, 127.08, 126.70, 120.60, 51.17, 39.07, 37.34.

IR (ATR): $\tilde{\nu}$ (cm^{-1}) = 3083, 3057, 3025, 2931, 2855, 1679, 1597, 1580, 1490, 1446, 1347, 1243, 1219, 1178, 1157, 1068, 1049, 1026, 1002.

HRMS (APCI+) m/z: $[M+H]^+$ Calcd for $C_{25}H_{23}OS$ 371.1464; Found: 371.1466.

Phenyl(3-phenyl-3-((1-phenylvinyl)thio)cyclobutyl)methanone (3al'')

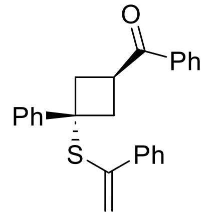

3al''

BCB **1a** (150.0 mg, 640 µmol, 1.0 eq.) and thiobenzophenone **2l** (218.0 mg, 1.6 mmol, 2.5 eq.) were reacted in MeCN (20.0 mL) for 8 h according to **GP**. Column chromatography (SiO_2, Pentane:EtOAc 20:1) afforded the title compound **3al''** (22.6 mg, 61 µmol, 10%) as a yellowish oil.

^{1}H-NMR (500 MHz, $CDCl_3$): δ = 7.88 – 7.84 (m, 2H), 7.58 – 7.53 (m, 1H), 7.51 – 7.47 (m, 2H), 7.47 – 7.43 (m, 2H), 7.29 – 7.26 (m, 3H), 7.23 – 7.18 (m, 2H), 7.18 – 7.15 (m, 2H), 7.14 – 7.09 (m, 1H), 5.58 (s, 1H), 5.18 (s, 1H), 4.37 (tt, J = 9.5, 8.4 Hz, 1H), 2.98 – 2.89 (m, 2H), 2.82 – 2.72 (m, 2H).

^{13}C-NMR {^{1}H} (126 MHz, $CDCl_3$): δ = 200.09, 146.07, 142.84, 140.54, 135.49, 133.27, 128.76, 128.43, 128.30, 128.26, 128.00, 127.38, 126.42, 126.02, 120.14, 52.23, 37.75, 37.72.

IR (ATR): $\tilde{\nu}$ (cm^{-1}) = 3082, 3057, 3026, 2977, 2928, 2851, 1679, 1597, 1580, 1571, 1490, 1446, 1424, 1348, 1276, 1222, 1179, 1158, 1067, 1026, 1002.

HRMS (APCI+) m/z: $[M+H]^+$ Calcd for $C_{25}H_{23}OS$ 371.1464; Found: 371.1469.

(3,3-Di-*p*-tolyl-2-thiabicyclo[2.1.1]hexan-4-yl)(naphthalen-2-yl)methanone (3ra)

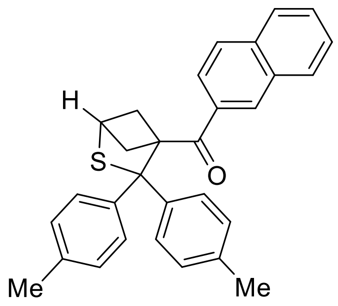

3ra

BCB **1r** (20.8 mg, 100 µmol, 1.0 eq.) and thiobenzophenone **2a** (56.6 mg, 250 µmol, 2.5 eq.) were reacted in MeCN (4.0 mL) for 3 h according to **GP**. Filtration through a pad of silica gel in toluene with a following gel permeation chromatography (JAIGEL-2H, chloroform) afforded the title compound **3ra** (34.5 mg, 74 µmol, 74%) as a white solid.

^{1}H-NMR (700 MHz, $CDCl_3$): δ = 7.73 – 7.69 (m, 1H), 7.56 (dd, J = 8.7, 0.6 Hz, 1H), 7.53 (dd, J = 8.7, 1.8 Hz, 1H), 7.47 (ddd, J = 8.2, 6.7, 1.8 Hz, 1H), 7.41 – 7.33 (m, 6H), 6.98 – 6.96 (m, 1H), 6.86 – 6.81 (m, 4H), 3.68 (t, J = 3.4 Hz, 1H), 3.00 (dd, J = 5.7, 2.3 Hz, 2H), 2.85 (ddd, J = 5.7, 3.4, 2.3 Hz, 2H), 2.11 (s, 6H).

^{13}C-NMR {^{1}H} (176 MHz, $CDCl_3$): δ = 202.31, 143.58, 136.22, 134.87, 131.94, 131.19, 129.91, 129.89, 128.29, 128.21, 127.40, 127.38, 126.11, 124.54, 68.14, 49.86, 44.16, 20.82.

IR (ATR): $\tilde{\nu}$ (cm^{-1}) = 2997, 2946, 2914, 1655, 1628, 1597, 1506, 1466, 1436, 1350, 1283, 1243, 1225, 1198, 1186, 1156, 1113, 1019.

HRMS (APCI+) m/z: $[M+H]^+$ Calcd for $C_{30}H_{27}OS$ 435.1777; Found: 435.1770.

m.p.: 187 - 189 °C.

(3,3-Diphenyl-2-thiabicyclo[2.1.1]hexan-4-yl)(naphthalen-2-yl)methanone (3rb)

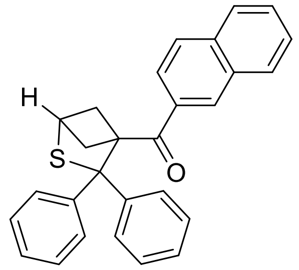

3rb

BCB **1r** (20.8 mg, 100 µmol, 1.0 eq.) and thiobenzophenone **2b** (49.6 mg, 250 µmol, 2.5 eq.) were reacted in MeCN (4.0 mL) for 3 h according to **GP**. Filtration through a pad

of silica gel in toluene with a following gel permeation chromatography (JAIGEL-2H, chloroform) afforded the title compound **3rb** (35.8 mg, 88 µmol, 88%) as a yellowish solid.

^{1}H-NMR (500 MHz, CDCl$_3$): δ = 7.71 – 7.68 (m, 1H), 7.56 – 7.52 (m, 5H), 7.50 – 7.43 (m, 2H), 7.39 – 7.37 (m, 2H), 7.10 – 6.99 (m, 7H), 3.68 (t, *J* = 3.4 Hz, 1H), 3.03 – 2.97 (m, 2H), 2.88 – 2.81 (m, 2H).

^{13}C-NMR {^{1}H} (126 MHz, CDCl$_3$): δ = 202.0, 146.4, 134.9, 134.7, 131.9, 131.2, 130.0, 130.0, 128.3, 127.7, 127.4, 127.3, 126.5, 126.1, 124.5, 68.6, 67.8, 50.0, 44.2.

IR (ATR): $\tilde{\nu}$ (cm^{-1}) = 3057, 3000, 2949, 2923, 2853, 1651, 1626, 1597, 1490, 1463, 1442, 1318, 1286, 1216, 1195, 1179, 1135, 1106, 1084, 1030.

HRMS (APCI+) m/z: [M+H]$^+$ Calcd for $C_{28}H_{23}OS$ 407.1464; Found: 407.1458.

m.p.: 223 - 224 °C.

(3,3-Bis(4-fluorophenyl)-2-thiabicyclo[2.1.1]hexan-4-yl)(naphthalen-2-yl)methanone (3rc)

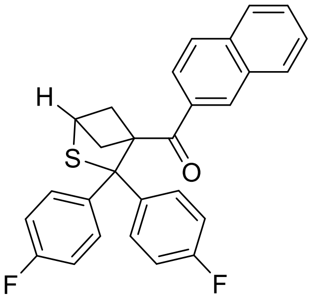

3rc

BCB **1r** (20.8 mg, 100 µmol, 1.0 eq.) and thiobenzophenone **2c** (58.6 mg, 250 µmol, 2.5 eq.) were reacted in MeCN (4.0 mL) for 3 h according to **GP**. Filtration through a pad of silica gel in toluene with a following gel permeation chromatography (JAIGEL-2H, chloroform) afforded the title compound **3rc** (31.0 mg, 70 µmol, 70%) as a brownish oil.

^{1}H-NMR (500 MHz, CDCl$_3$): δ = 7.73 (d, *J* = 8.3, 1H), 7.58 (d, *J* = 8.7 Hz, 1H), 7.55 – 7.37 (m, 8H), 7.17 – 7.12 (m, 1H), 6.81 – 6.67 (m, 4H), 3.71 (t, *J* = 3.4 Hz, 1H), 3.01 – 2.92 (m, 2H), 2.92 – 2.84 (m, 2H).

^{13}C-NMR {^{1}H} (126 MHz, CDCl$_3$): δ = 201.8, 161.3 (d, *J* = 247.4 Hz), 142.0 (d, *J* = 3.3 Hz), 135.0, 134.6, 131.9, 131.6 (d, *J* = 7.7 Hz), 131.1, 129.7, 128.6, 127.6, 127.5, 126.6, 124.2, 114.48 (d, *J* = 21.3 Hz), 68.0, 67.4, 49.9, 44.4.

^{19}F-NMR (471 MHz, CDCl$_3$): δ = -115.99 – -116.08 (m).

IR (ATR): $\tilde{\nu}$ (cm^{-1}) = 3062, 2996, 2954, 2910, 1736, 1651, 1626, 1599, 1504, 1466, 1281, 1227, 1200, 1161, 1114.

HRMS (APCI+) m/z: $[M+H]^+$ Calcd for $C_{28}H_{21}O_4F_2S$ 443.1276; Found: 443.1271.

m.p.: 224 - 225 °C

(3,3-Bis(4-methoxyphenyl)-2-thiabicyclo[2.1.1]hexan-4-yl)(naphthalen-2-yl)methanone (3rd)

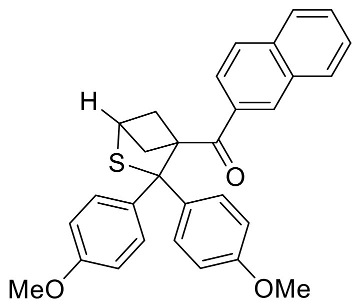

3rd

BCB **1r** (20.8 mg, 100 µmol, 1.0 eq.) and thiobenzophenone **2d** (64.6 mg, 250 µmol, 2.5 eq.) were reacted in MeCN (4.0 mL) for 3 h according to **GP**. Filtration through a pad of silica gel in toluene with a following gel permeation chromatography (JAIGEL-2H, chloroform) afforded the title compound **3rd** (31.6 mg, 68 µmol, 68%) as a white solid.

^{1}H-NMR (500 MHz, $CDCl_3$): δ = 7.71 (d, *J* = 8.3 Hz, 1H), 7.58 (d, *J* = 8.7 Hz, 1H), 7.53 – 7.46 (m, 3H), 7.43 – 7.35 (m, 6H), 7.07 (m, 1H), 6.59 – 6.51 (m, 4H), 3.69 (t, *J* = 3.4 Hz, 1H), 3.56 (s, 6H), 2.99 (dd, *J* = 5.8, 2.3 Hz, 2H), 2.90 – 2.84 (m, 2H).

^{13}C-NMR {^{1}H} (126 MHz, $CDCl_3$): δ = 202.4, 158.0, 138.7, 134.9, 134.8, 132.0, 131.2, 131.1, 129.9, 128.2, 127.4, 127.3, 126.1, 124.5, 112.8, 68.3, 67.7, 55.1, 55.0, 49.8, 44.1.

IR (ATR): $\tilde{\nu}$ (cm^{-1}) = 3000, 2957, 2931, 2908, 2834, 1651, 1626, 1603, 1579, 1507, 1456, 1440, 1290, 1248, 1227, 1184, 1160, 1124, 1115, 1040, 1031.

HRMS (APCI+) m/z: $[M+H]^+$ Calcd for $C_{30}H_{27}O_3S$ 467.1675; Found: 467.1669.

m.p.: 152 - 154 °C.

Naphthalen-2-yl(3-thiaspiro[bicyclo[2.1.1]hexane-2,9'-xanthen]-1-yl)methanone (3re)

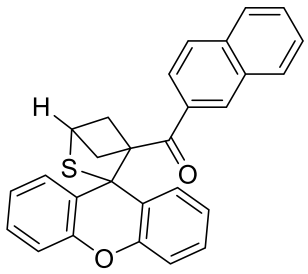

3re

BCB **1r** (20.8 mg, 100 µmol, 1.0 eq.) and thiobenzophenone **2e** (53.1 mg, 250 µmol, 2.5 eq.) were reacted in MeCN (4.0 mL) for 3 h according to **GP**. Filtration through a pad of silica gel in toluene with a following gel permeation chromatography (JAIGEL-2H, chloroform) afforded the title compound **3re** (32.0 mg, 76 µmol, 76%) as a white solid.

^{1}H-NMR (500 MHz, $CDCl_3$): δ = 8.28 (dd, J = 7.8, 1.7 Hz, 2H), 7.73 (d, J = 8.5 Hz, 1H), 7.62 (d, J = 8.5 Hz, 1H), 7.55 – 7.46 (m, 2H), 7.43 – 7.39 (m, 2H), 7.30 – 7.23 (m, 2H), 7.18 (ddd, J = 8.0, 7.3, 1.7 Hz, 2H), 6.98 – 6.94 (m, 1H), 6.75 (dd, J = 8.0, 1.3 Hz, 2H), 3.68 (t, J = 3.2 Hz, 1H), 2.86 – 2.79 (m, 2H), 2.47 – 2.40 (m, 2H).

^{13}C-NMR {^{1}H} (126 MHz, $CDCl_3$): δ = 197.9, 153.7, 135.0, 133.6, 132.0, 130.7, 130.1, 129.8, 129.0, 128.3, 127.6, 127.5, 126.1, 125.8, 124.3, 123.0, 116.4, 69.1, 60.4, 47.0, 42.3.

IR (ATR): $\tilde{\nu}$ (cm^{-1}) = 3064, 3009, 2986, 2924, 2854, 1656, 1624, 1614, 1593, 1570, 1464, 1457, 1444, 1347, 1288, 1278, 1234, 1224, 1197, 1187, 1151, 1113, 1099, 1036.

HRMS (APCI+) m/z: $[M+H]^+$ Calcd for $C_{28}H_{21}O_2S$ 421.1253; Found: 421.1257.

m.p.: 220 - 222 °C.

Naphthalen-2-yl(3-thiaspiro[bicyclo[2.1.1]hexane-2,9'-thioxanthen]-1-yl)methanone (3rf)

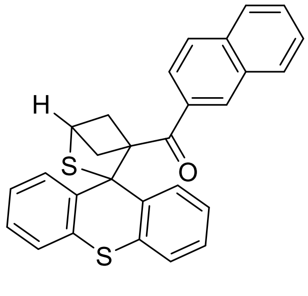

3rf

BCB **1r** (20.8 mg, 100 µmol, 1.0 eq.) and thiobenzophenone **2f** (57.1 mg, 250 µmol, 2.5 eq.) were reacted in MeCN (4.0 mL) for 3 h according to **GP**. The solid residue was washed with 1 mL acetonitrile two times resulting in the title compound **3rf** (23.0 mg, 53 µmol, 53%) as a white solid.

^{1}H-NMR (500 MHz, $CDCl_3$): δ = 8.71 (m, 2H), 7.75 (dd, *J* = 8.2, 1.2 Hz, 1H), 7.67 – 7.63 (m, 1H), 7.58 (dd, *J* = 8.7, 1.8 Hz, 1H), 7.50 (ddd, *J* = 8.1, 6.8, 1.2 Hz, 1H), 7.44 (m, 2H), 7.39 (ddd, *J* = 8.2, 6.8, 1.2 Hz, 1H), 7.33 – 7.28 (m, 1H), 7.26 – 7.22 (m, 2H), 7.07 (br.s, 2H), 6.84 (s, 1H), 3.64 (t, *J* = 3.2 Hz, 1H), 2.76 – 2.67 (m, 2H), 2.37 (br.s, 2H).

^{13}C-NMR {^{1}H} (126 MHz, $CDCl_3$): δ = 197.2, 135.0, 134.0, 132.6, 132.0, 130.2, 129.7, 128.2, 127.7, 127.5, 126.9, 126.1, 126.1, 124.3, 68.1, 67.5, 48.0, 41.6.

IR (ATR): $\tilde{\nu}$ (cm^{-1}) = 3060, 3009, 2988, 1657, 1644, 1633, 1589, 1461, 1451, 1432, 1319, 1281, 1223, 1161, 1151, 1112, 1083, 1073, 1032.

HRMS (ESI+) m/z: $[M+Na]^+$ Calcd for $C_{28}H_{20}ONaS_2$ 459.0848; Found: 459.0850.

m.p.: 191 - 193 °C.

(2',3'-Diphenyl-3-thiaspiro[bicyclo[2.1.1]hexane-2,1'-cyclopropan]-2'-en-1-yl)(naphthalen-2-yl)methanone (3rg)

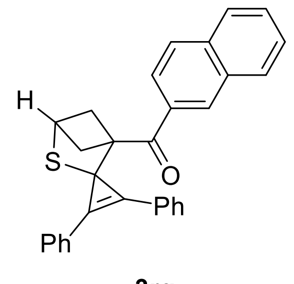

3rg

BCB **1r** (20.8 mg, 100 µmol, 1.0 eq.) and thiobenzophenone **2g** (55.6 mg, 250 µmol, 2.5 eq.) were reacted in MeCN (4.0 mL) for 3 h according to **GP**. Filtration through a pad of silica gel (washed with 1% Et_3N in Toluene) in toluene afforded the title compound **3rg** (27.0 mg, 63 µmol, 63%) as a yellow oil.

^{1}H-NMR (500 MHz, $CDCl_3$): δ = 8.19 (dd, *J* = 1.7, 0.7 Hz, 1H), 7.74 – 7.69 (m, 5H), 7.67 – 7.64 (m, 1H), 7.59 – 7.55 (m, 1H), 7.44 (ddd, *J* = 8.1, 6.8, 1.3 Hz, 1H), 7.42 – 7.32 (m, 6H), 7.32 – 7.26 (m, 2H), 3.69 (t, *J* = 3.8 Hz, 1H), 3.10 – 3.04 (m, 2H), 2.94 – 2.88 (m, 2H).

^{13}C-NMR {^{1}H} (126 MHz, $CDCl_3$): δ = 201.71, 135.26, 133.52, 132.00, 130.88, 129.64, 129.39, 129.32, 128.82, 128.23, 128.15, 128.08, 127.53, 126.31, 124.07, 119.41, 65.66, 50.48, 49.98, 43.08.

IR (ATR): $\tilde{\nu}$ (cm^{-1}) = 2924, 2853, 1770, 1734, 1673, 1599, 1460, 1445, 1241, 1177.

HRMS (ESI+) m/z: $[M+H]^+$ Calcd for $C_{30}H_{23}OS$ 431.1464; Found: 431.1460.

6. Functionalisation products

(2-Oxido-1-phenyl-3,3-di-*p*-tolyl-2-thiabicyclo[2.1.1]hexan-4-yl)(phenyl)methanone (4)

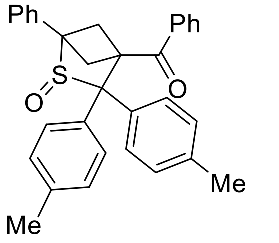

4

Compound **3aa** (20 mg, 43 µmol, 1.0 equiv.) was dissolved in dichloromethane (2 mL). To the stirring solution was added meta-chloroperoxybenzoic acid (*m*-CPBA) (10 mg (75%), 44 µmol, 1.0 equiv). The reaction was allowed to stir for 4 h at 0 °C. To the solution was added more dichloromethane, washed with 2M NaOH solution and extracted using dichloromethane (20 mL x 3). The combined organic layers were dried using Na_2SO_4 and the solvent was evaporated off under reduced pressure. The solid residue was washed with acetonitrile afforded the title compound **4** (20.5 mg, 43 µmol, 99%) as a colorless solid.

^{1}H-NMR (500 MHz, $CDCl_3$): δ = 7.44 – 7.41 (m, 2H), 7.38 – 7.27 (m, 6H), 7.14 – 7.10 (m, 2H), 7.07 – 6.98 (m, 6H), 6.85 – 6.81 (m, 2H), 3.93 (dd, *J* = 9.6, 9.0 Hz, 1H), 3.21 (dd, *J* = 10.3, 9.0 Hz, 1H), 3.14 (d, *J* = 9.6 Hz, 1H), 2.88 (d, *J* = 10.3 Hz, 1H), 2.30 (s, 3H), 2.24 (s, 3H).

^{13}C-NMR {^{1}H} (126 MHz, $CDCl_3$): δ = 13C NMR (176 MHz, CDCl3) δ 200.3, 139.5, 137.5, 137.5, 137.2, 135.1, 134.7, 132.3, 132.2, 130.7, 128.8, 128.7, 128.5, 128.5, 128.2, 127.7, 126.6, 82.6, 68.2, 61.8, 45.7, 40.4, 21.2, 20.9.

IR (ATR): $\tilde{\nu}$ (cm^{-1}) = 3057, 3025, 2919, 2852, 1655, 1597, 1578, 1509, 1445, 1315, 1277, 1222, 1178, 1123, 1072, 1024, 1009, 1000.
HRMS (ESI+) m/z: $[M+Na]^+$ Calcd for $C_{32}H_{28}O_2NaS$ 499.1702; Found: 499.1710.
m.p.: 236 - 238 °C.

(2,2-Dioxido-1-phenyl-3,3-di-*p*-tolyl-2-thiabicyclo[2.1.1]hexan-4-yl)(phenyl)methanone (5)

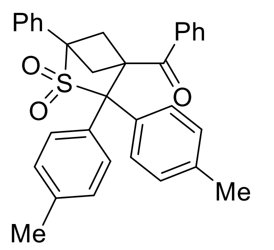

5

Compound **3aa** (20 mg, 43 µmol, 1.0 equiv.) was dissolved in dichloromethane (2 mL). To the stirring solution was added meta-chloroperoxybenzoic acid (*m*-CPBA) (80 mg (75%), 347 µmol, 8.0 equiv). The reaction was allowed to stir for 4 h. To the solution was added more dichloromethane, washed with 2M NaOH solution and extracted using dichloromethane (20 mL x 3). The combined organic layers were dried using Na_2SO_4 and the solvent was evaporated off under reduced pressure. The solid residue was washed with acetonitrile afforded the title compound **5** (21.0 mg, 43 µmol, 98%) as a colorless solid.
^{1}H-NMR (500 MHz, $CDCl_3$): δ = 7.47 – 7.42 (m, 4H), 7.42 – 7.36 (m, 5H), 7.30 – 7.24 (m, 1H), 7.04 – 6.93 (m, 8H), 3.66 – 3.56 (m, 2H), 3.00 – 2.89 (m, 2H), 2.26 (s, 6H).
^{13}C-NMR {^{1}H} (126 MHz, $CDCl_3$): δ = 199.6, 137.8, 137.0, 136.6, 132.1, 130.7, 130.3, 129.2, 128.8, 128.5, 128.4, 127.8, 127.7, 80.6, 70.4, 59.4, 41.3, 21.0.
IR (ATR): $\tilde{\nu}$ (cm^{-1}) = 2920, 2852, 1661, 1596, 1507, 1498, 1466, 1446, 1322, 1302, 1281, 1223, 1194, 1155, 1114, 1034.
HRMS (ESI+) m/z: $[M+Na]^+$ Calcd for $C_{32}H_{28}O_3NaS$ 515.1651; Found: 515.1643.
m.p.: 241 - 243 °C.

(2,2-Dioxido-3,3-di-*p*-tolyl-2-thiabicyclo[2.1.1]hexan-4-yl)(naphthalen-2-yl)methanone (6)

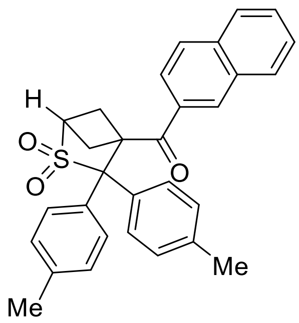

6

Compound **3ra** (20 mg, 43 µmol, 1.0 equiv.) was dissolved in dichloromethane (2 mL). To the stirring solution was added meta-chloroperoxybenzoic acid (*m*-CPBA) (80 mg (75%), 347 µmol, 8.0 equiv). The reaction was allowed to stir for 4 h. To the solution was added more dichloromethane, washed with 2M NaOH solution and extracted using dichloromethane (20 mL x 3). The combined organic layers were dried using Na_2SO_4 and the solvent was evaporated off under reduced pressure. The solid residue was washed with acetonitrile afforded the title compound **6** (21.0 mg, 43 µmol, 98%) as a colorless solid.

^{1}H-NMR (700 MHz, $CDCl_3$): δ = 7.73 – 7.69 (m, 1H), 7.56 (d, *J* = 8.7 Hz, 1H), 7.50 (dt, *J* = 8.2, 4.0 Hz, 1H), 7.48 – 7.39 (m, 7H), 7.04 (dd, *J* = 1.7, 0.9 Hz, 1H), 6.91 – 6.86 (m, 4H), 3.86 (t, *J* = 3.7 Hz, 1H), 3.27 – 3.18 (m, 2H), 2.94 – 2.88 (m, 2H), 2.08 (s, 6H).

^{13}C-NMR {^{1}H} (176 MHz, $CDCl_3$): δ = 199.1, 137.9, 136.4, 134.9, 133.8, 131.8, 130.8, 130.7, 129.8, 128.6, 128.5, 127.8, 127.5, 126.4, 124.1, 78.7, 64.4, 58.3, 38.3, 20.8.

IR (ATR): $\tilde{\nu}$ (cm^{-1}) = 3017, 2854, 2922, 1656, 1627, 1510, 1465, 1325, 1300, 1246, 1232, 1215, 1199, 1169, 1112, 1024.

HRMS (APCI+) m/z: $[M+H-SO_2]^+$ Calcd for $C_{30}H_{27}O$ 403.2056; Found: 403.2064.

m.p.: 99 - 100 °C.

Phenyl(1-phenyl-3,3-di-*p*-tolyl-2-thiabicyclo[2.1.1]hexan-4-yl)methanol (7)

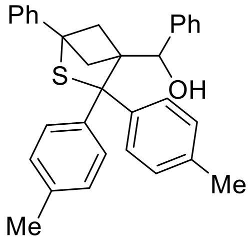

7

Compound **3aa** (20 mg, 43 µmol, 1.0 equiv.) was dissolved in THF (4 mL). To the stirring solution was added $LiAlH_4$ (1.7 mg, 44 µmol, 1.0 equiv) at 0 °C. The reaction was allowed to stir overnight at room temperature. Then saturated solution of NH_4Cl was added and extracted using EtOAc (20 mL x 3). The combined organic layers were dried using Na_2SO_4 and the solvent was evaporated off under reduced pressure. Filtration through a pad of silica gel in toluene with a following gel permeation chromatography (JAIGEL-2H, chloroform) afforded the title compound **7** (13.0 mg, 28 µmol, 65%) as a yellow oil.

^{1}H-NMR (500 MHz, $CDCl_3$): δ = 7.71 (d, *J* = 8.3 Hz, 2H), 7.39 – 7.30 (m, 6H), 7.26 (m, 5H), 7.21 – 7.16 (m, 5H), 5.72 (s, 1H), 2.90 – 2.76 (m, 2H), 2.43 (d, *J* = 8.0 Hz, 1H), 2.38 (s, 3H), 2.36 (s, 3H), 1.80 (d, *J* = 8.0 Hz, 1H).

^{13}C-NMR {^{1}H} (126 MHz, $CDCl_3$): δ = 144.5, 142.9, 142.8, 139.1, 137.3, 135.9, 129.8, 129.2, 128.7, 128.9, 128.5, 128.4, 127.6, 127.4, 126.3, 126.2, 71.1, 69.9, 61.4, 57.6, 51.6, 43.3, 21.0, 21.0.

IR (ATR): $\tilde{\nu}$ (cm^{-1}) = 3564, 3459, 3059, 3027, 2990, 2920, 2869, 1737, 1509, 1495, 1447, 1373, 1241, 1189, 1038, 1022.

HRMS (ESI+) m/z: $[M+Na]^+$ Calcd for $C_{32}H_{30}ONaS$ 485.1910; Found: 485.1908.

(3-Methyl-2-oxido-1,3-diphenyl-2-thiabicyclo[2.1.1]hexan-4-yl)(phenyl)methanone (8)

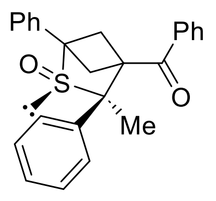

8

Compound **3ra** (20 mg, 54 µmol, 1.0 equiv.) was dissolved in dichloromethane (2 mL). To the stirring solution was added meta-chloroperoxybenzoic acid (*m*-CPBA) (12 mg (75%), 54 µmol, 1.0 equiv). The reaction was allowed to stir for 4 h at 0 °C. To the solution was added more dichloromethane, washed with 2M NaOH solution and extracted using dichloromethane (20 mL x 3). The combined organic layers were dried using Na_2SO_4 and the solvent was evaporated off under reduced pressure. Column chromatography (SiO_2, CH_2Cl_2) afforded the title compound **8** (19.7 mg, 51 µmol, 94%) as a single diastereomer in a form of colorless solid.

^{1}H-NMR (700 MHz, $CDCl_3$): δ = 7.51 – 7.25 (m, 15H), 3.72 (t, J = 9.1 Hz, 1H), 2.87 (d, J = 9.1 Hz, 1H), 2.76 (d, J = 10.5 Hz, 1H), 2.65 (dd, J = 10.5, 9.1 Hz, 1H), 1.79 (s, 3H).

^{13}C-NMR {^{1}H} (176 MHz, $CDCl_3$): δ = 200.63, 141.59, 137.65, 134.92, 133.05, 128.88, 128.86, 128.62, 128.59, 128.47, 128.23, 127.80, 126.60, 73.27, 68.96, 61.02, 44.58, 39.25, 19.00.

IR (ATR): $\tilde{\nu}$ (cm^{-1}) = 3058, 3027, 2989, 2952, 2927, 1654, 1598, 1579, 1498, 1445, 1318, 1282, 1225, 1206, 1176, 1157, 1131, 1075, 1065, 1049, 1028, 878.

HRMS (ESI+) m/z: $[M+H]^+$ Calcd for $C_{25}H_{23}O_2S$ 387.1413; Found: 387.1417.

m.p.: 147 - 149 °C.

7. NMR Spectra

Starting Materials

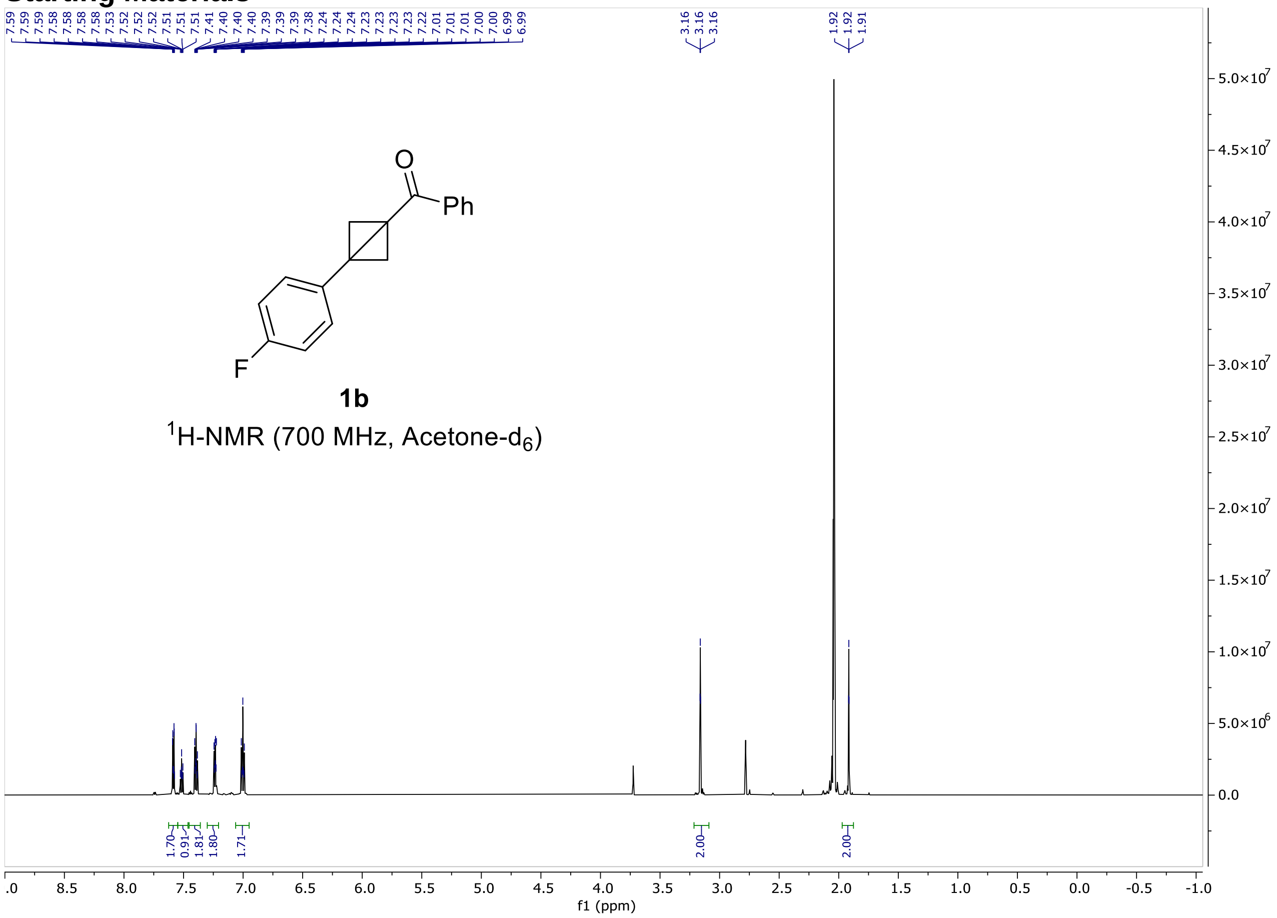

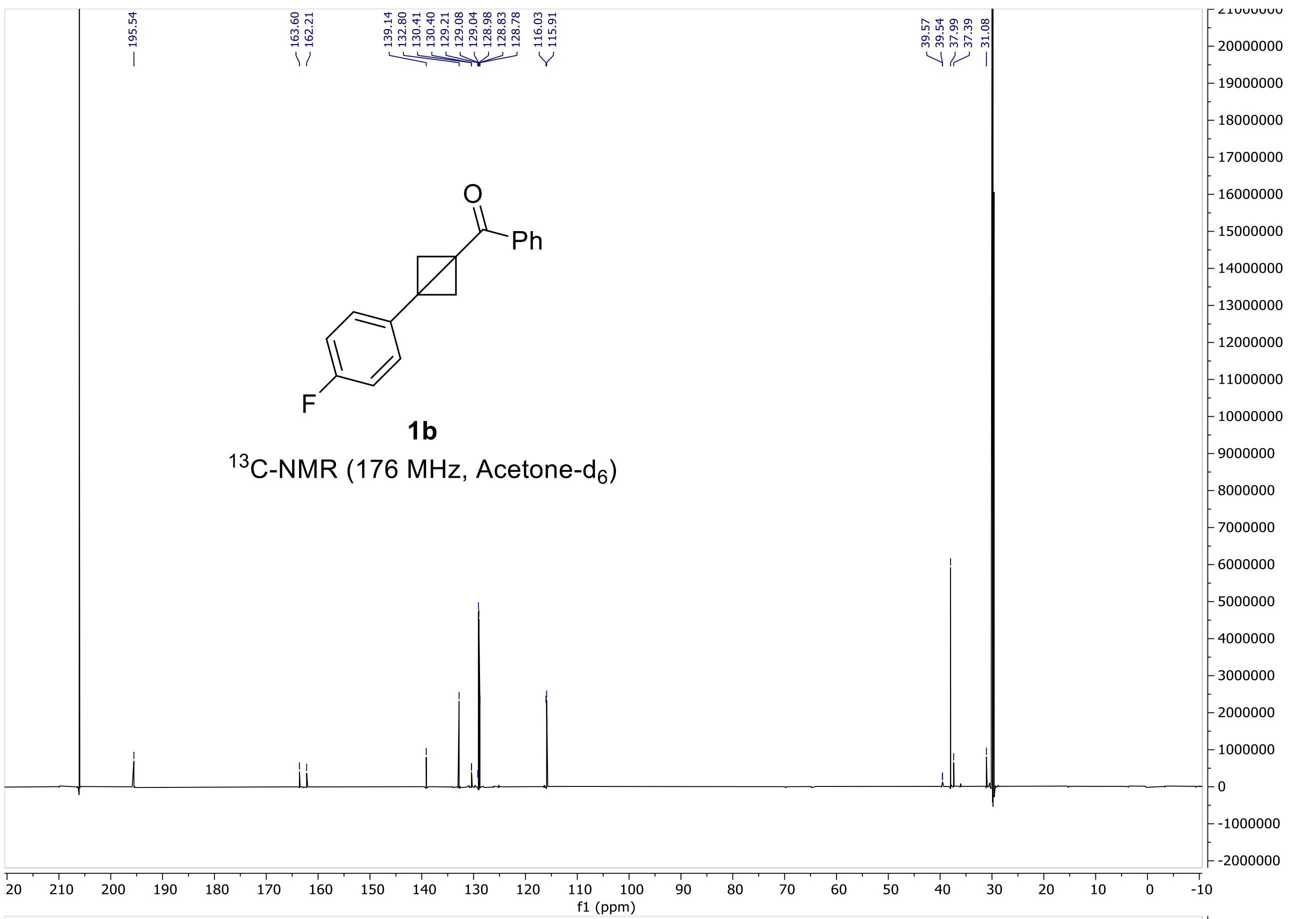
195.54
163.60
162.21
139.14
132.80
130.41
130.40
129.21
129.08
129.04
128.98
128.83
128.78
116.03
115.91
39.57
39.54
37.99
37.39
31.08
O
Ph
F
1b
^{13}C-NMR (176 MHz, Acetone-d_6)
f1 (ppm)

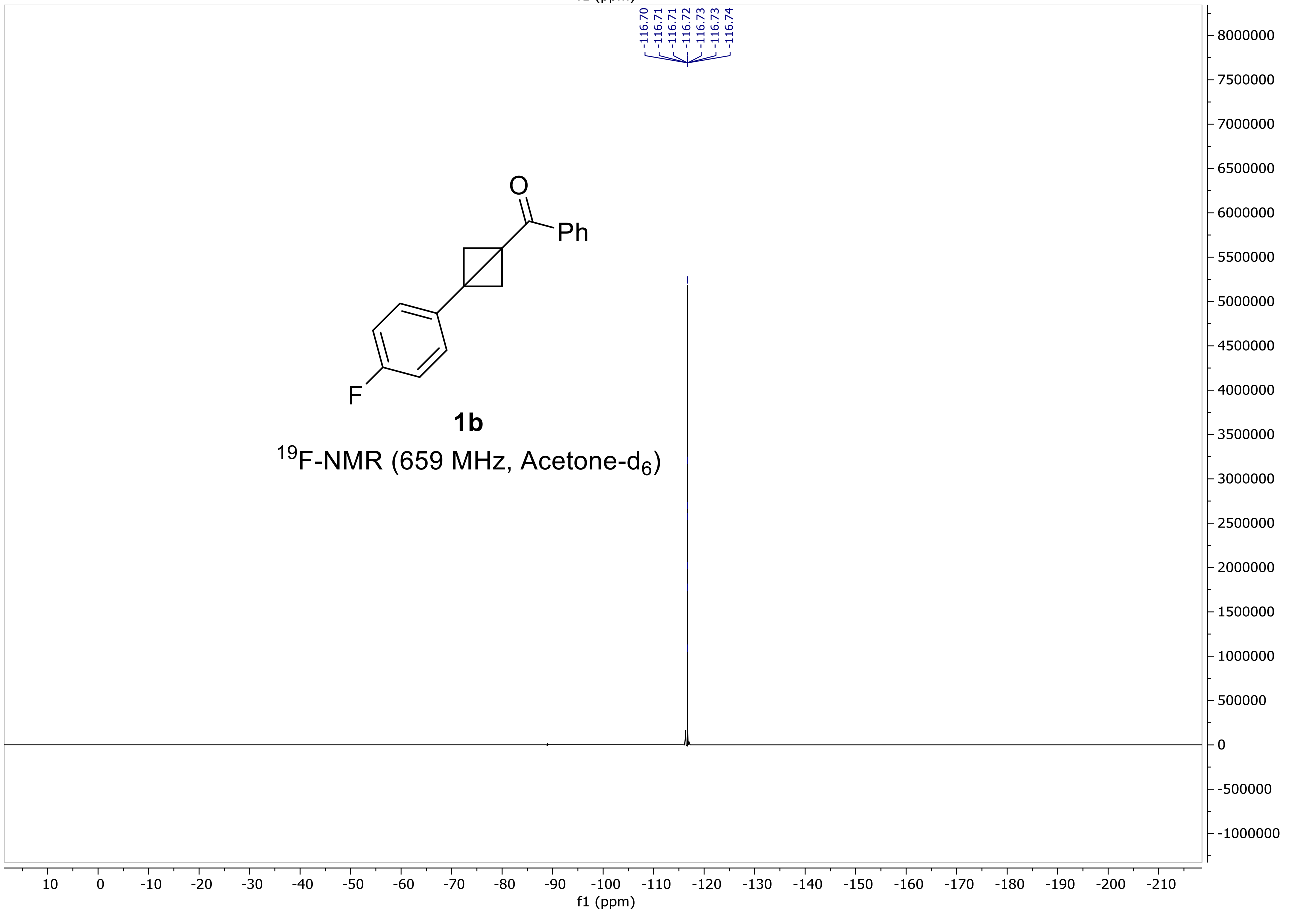
-116.70
-116.71
-116.71
-116.72
-116.73
-116.73
-116.74
O
Ph
F
1b
^{19}F-NMR (659 MHz, Acetone-d_6)
f1 (ppm)

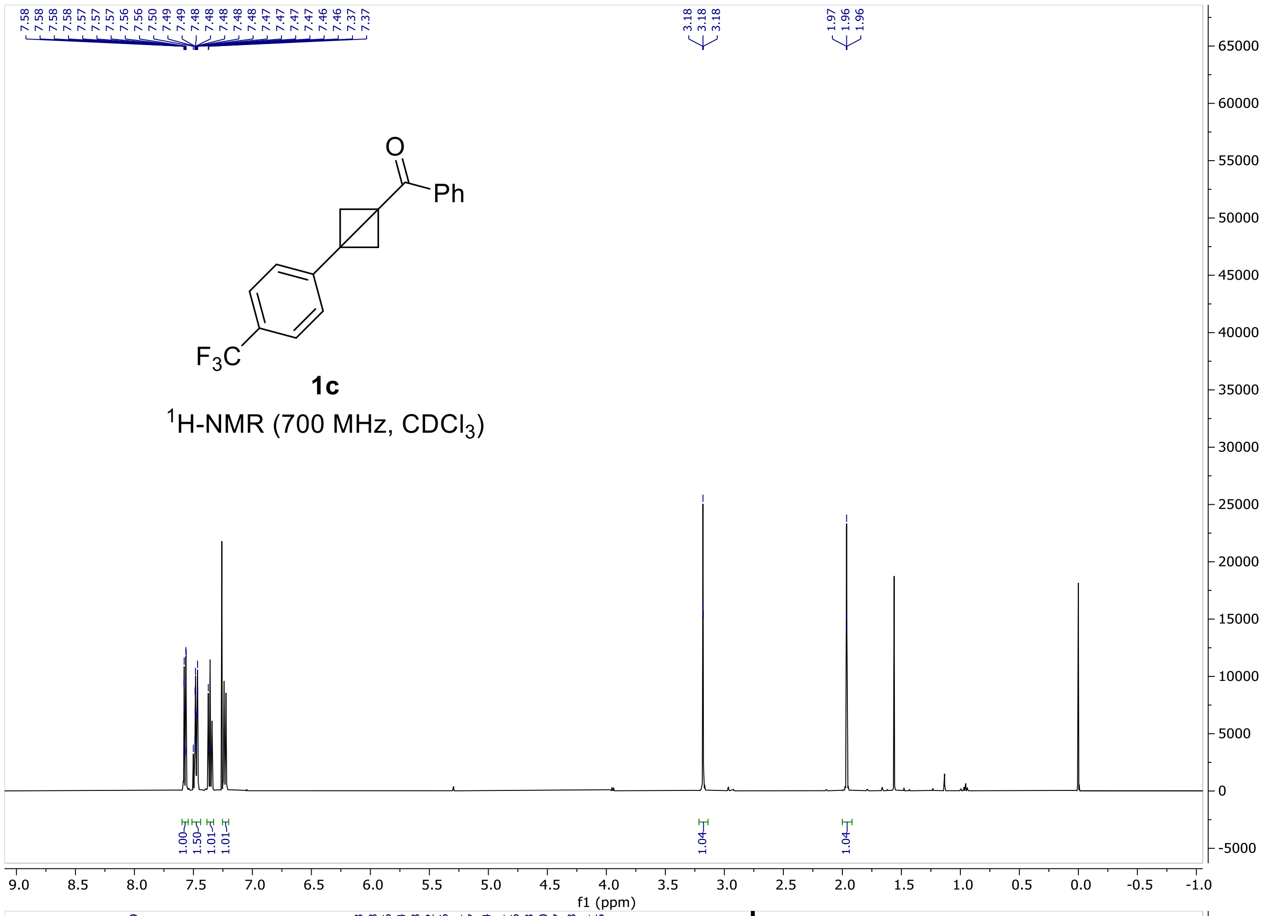
7.58 7.58 7.58 7.58 7.57 7.57 7.57 7.56 7.56 7.50 7.49 7.49 7.48 7.48 7.48 7.48 7.48 7.47 7.47 7.47 7.47 7.46 7.46 7.37 7.37
3.18 3.18 3.18
1.97 1.96 1.96
O
Ph
F3C
1c
1H-NMR (700 MHz, CDCl3)
1.00 1.50 1.01 1.01
1.04
1.04
f1 (ppm)

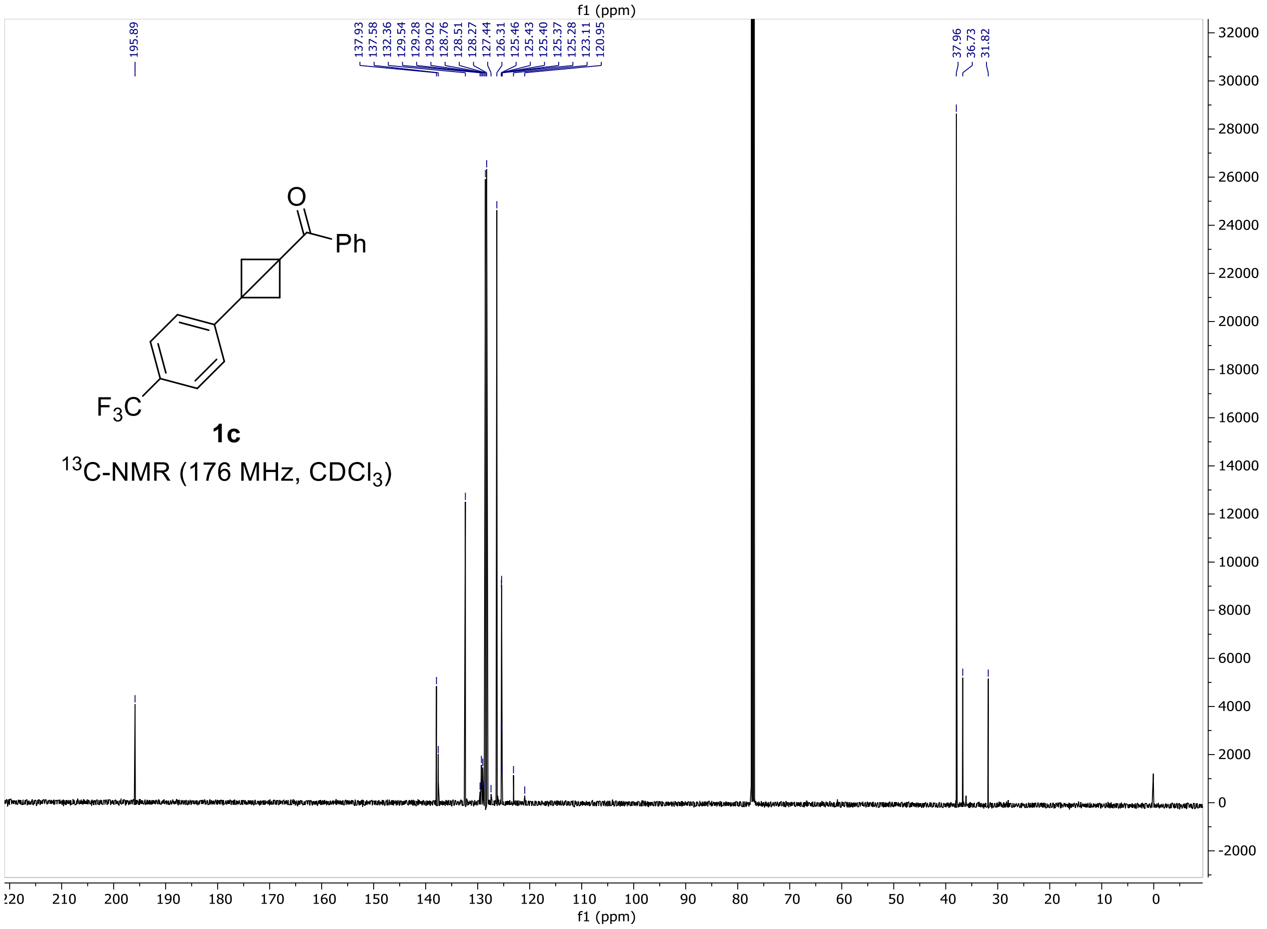
195.89
137.93 137.58 132.36 129.54 129.28 129.02 128.76 128.51 128.27 127.44 126.31 125.46 125.43 125.40 125.37 125.28 123.11 120.95
37.96 36.73 31.82
O
Ph
F3C
1c
13C-NMR (176 MHz, CDCl3)
f1 (ppm)

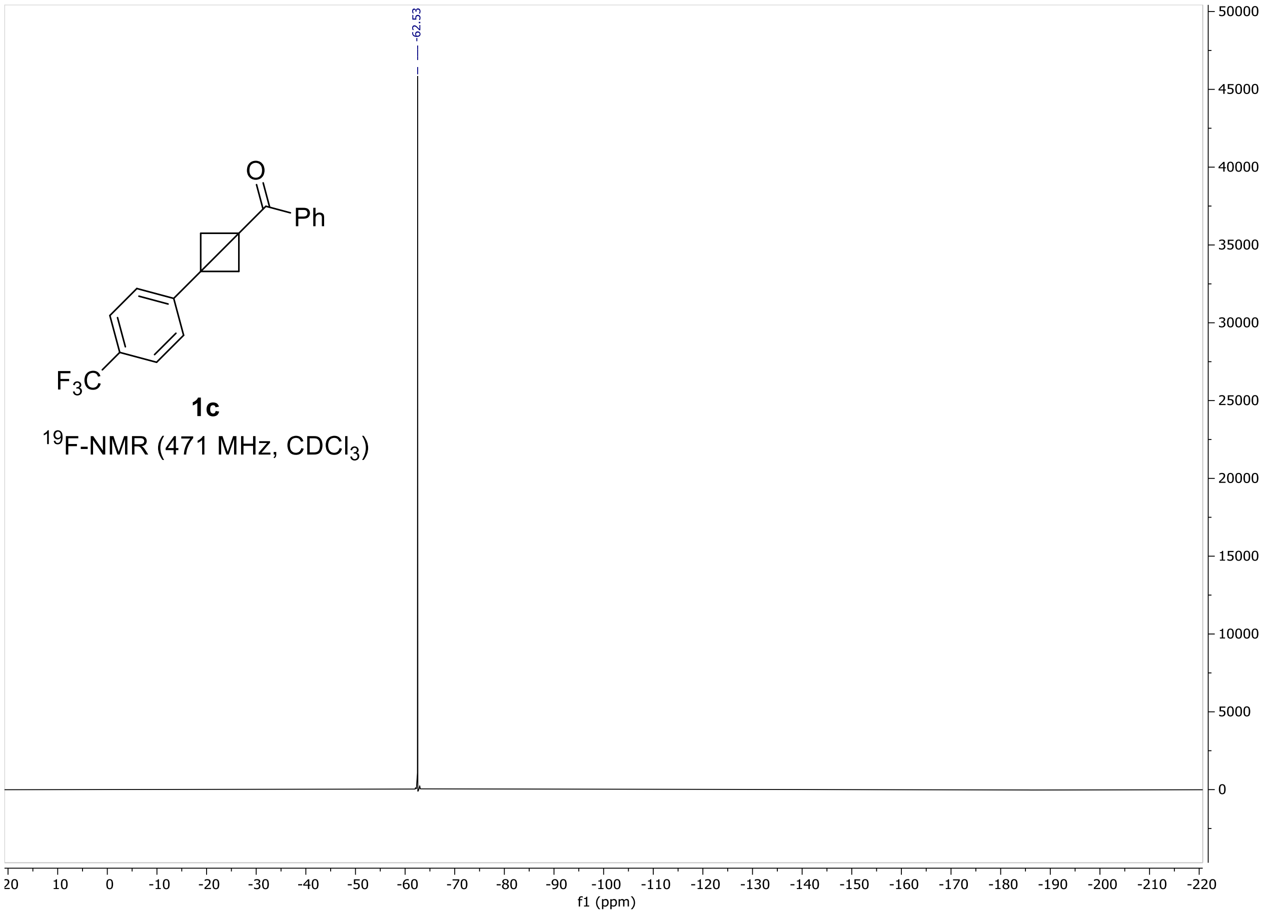
-62.53
O
Ph
F3C
1c
19F-NMR (471 MHz, CDCl3)
f1 (ppm)

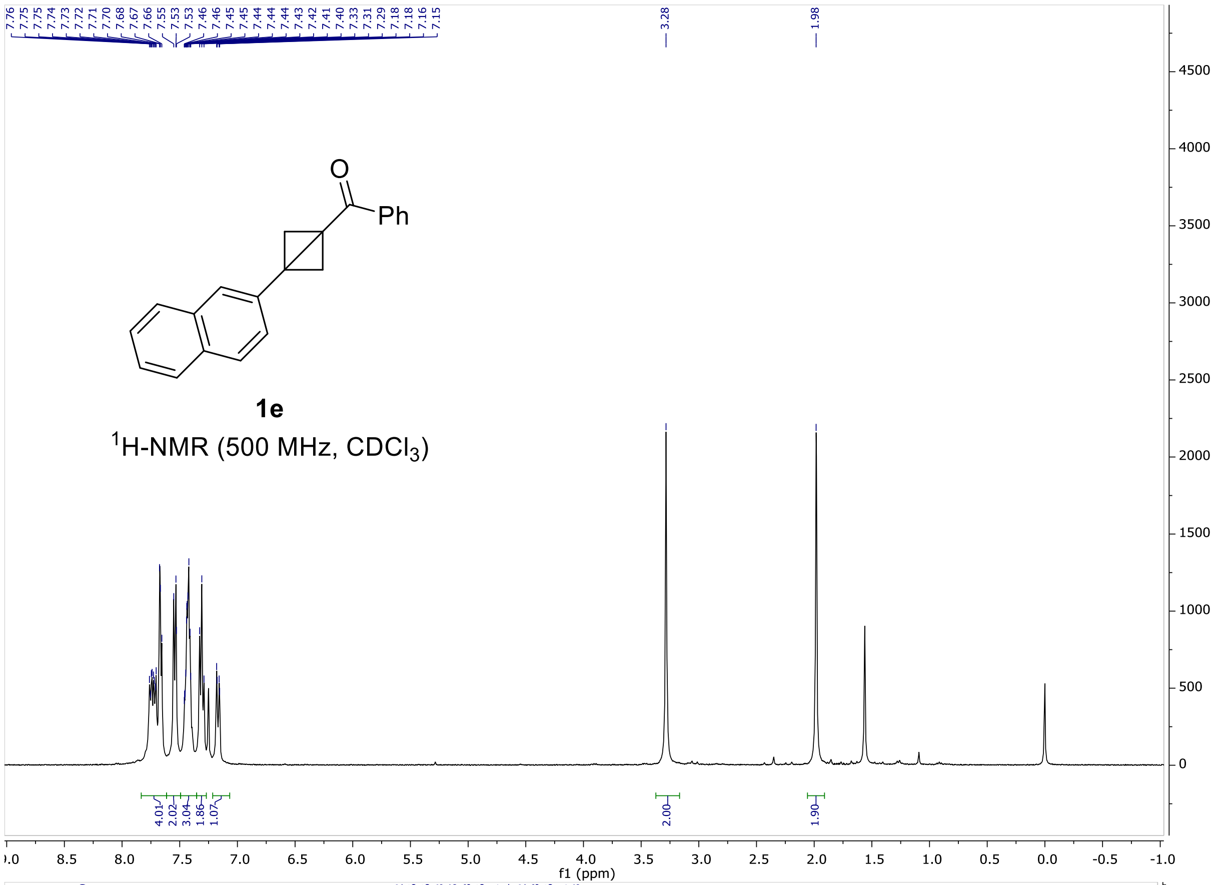

1e
^{1}H-NMR (500 MHz, CDCl$_3$)
f1 (ppm)

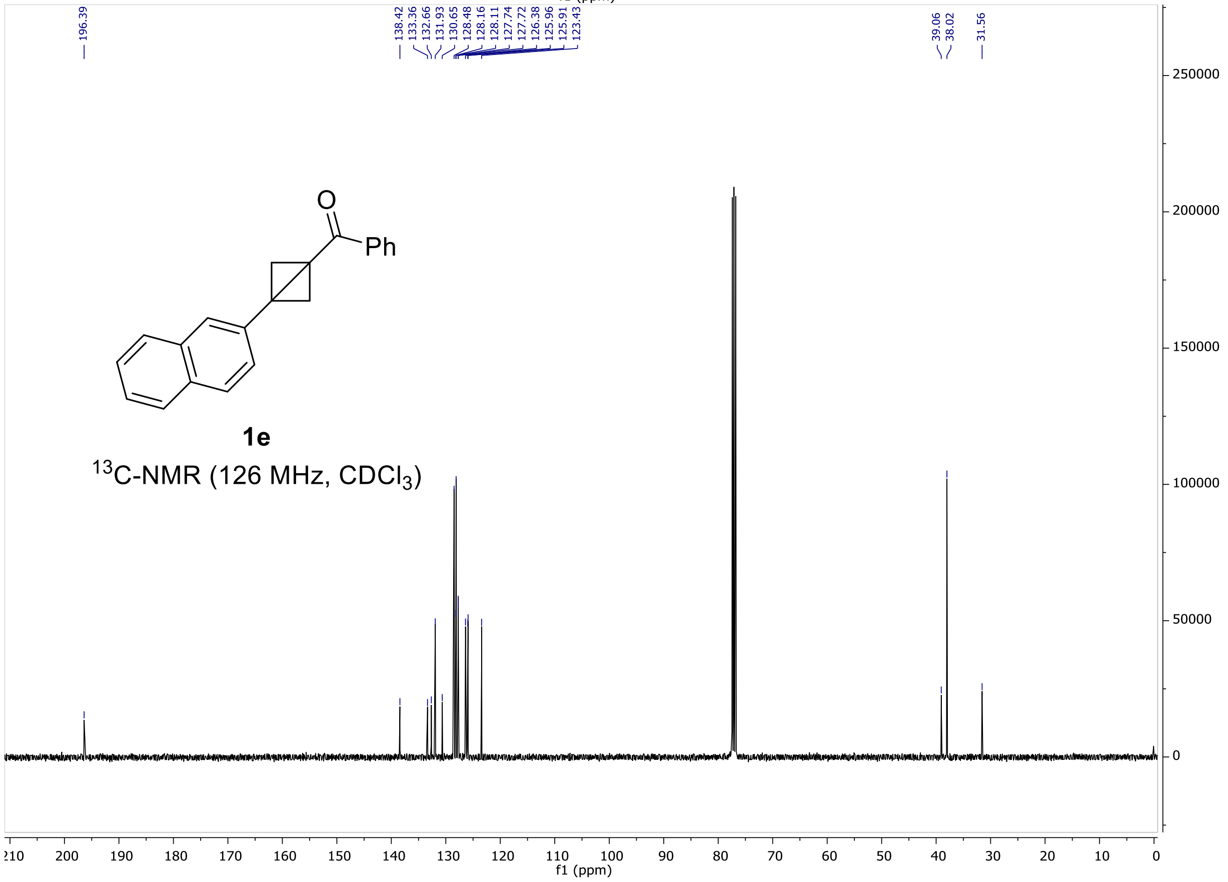

1e
^{13}C-NMR (126 MHz, CDCl$_3$)
f1 (ppm)

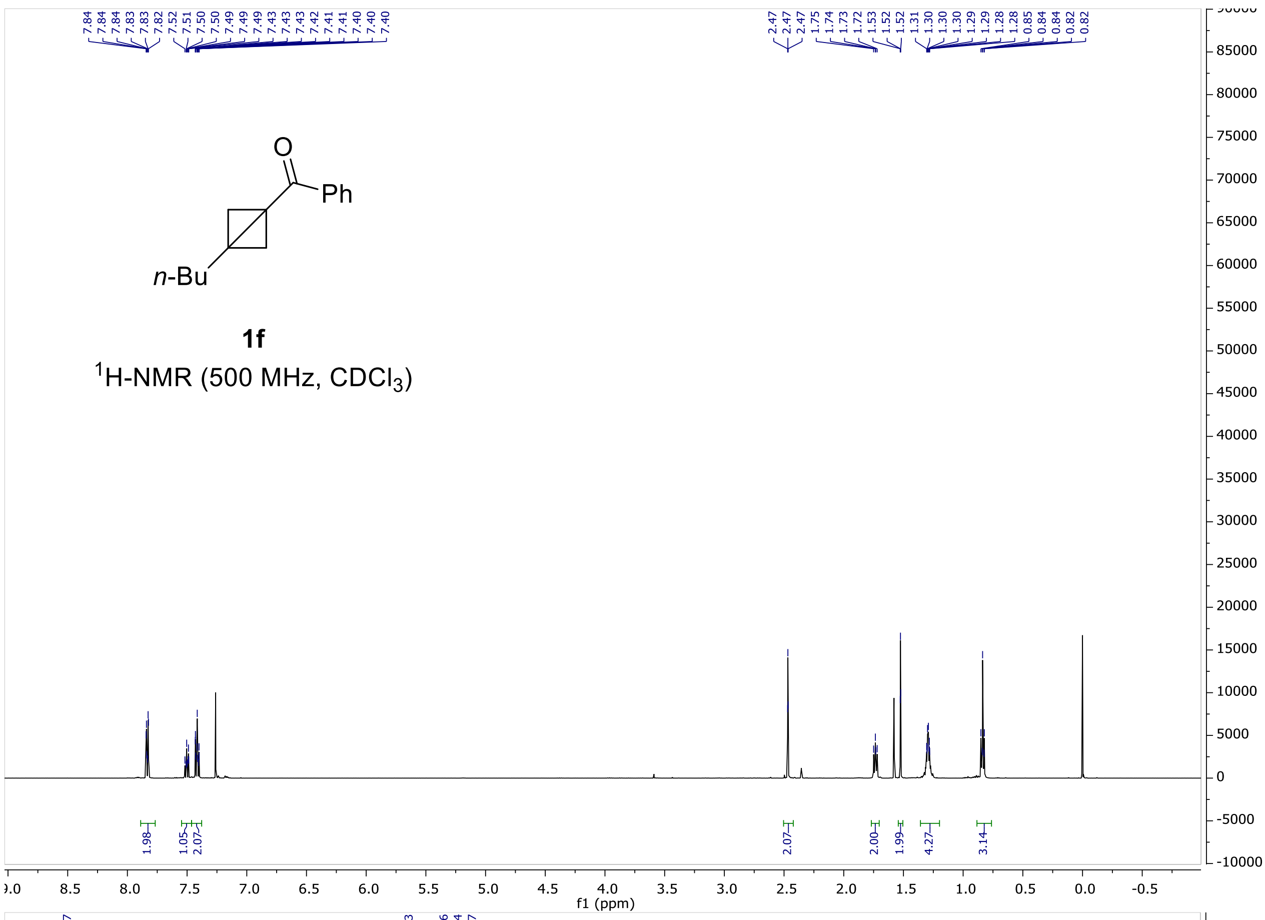

1f

^{1}H-NMR (500 MHz, $CDCl_3$)

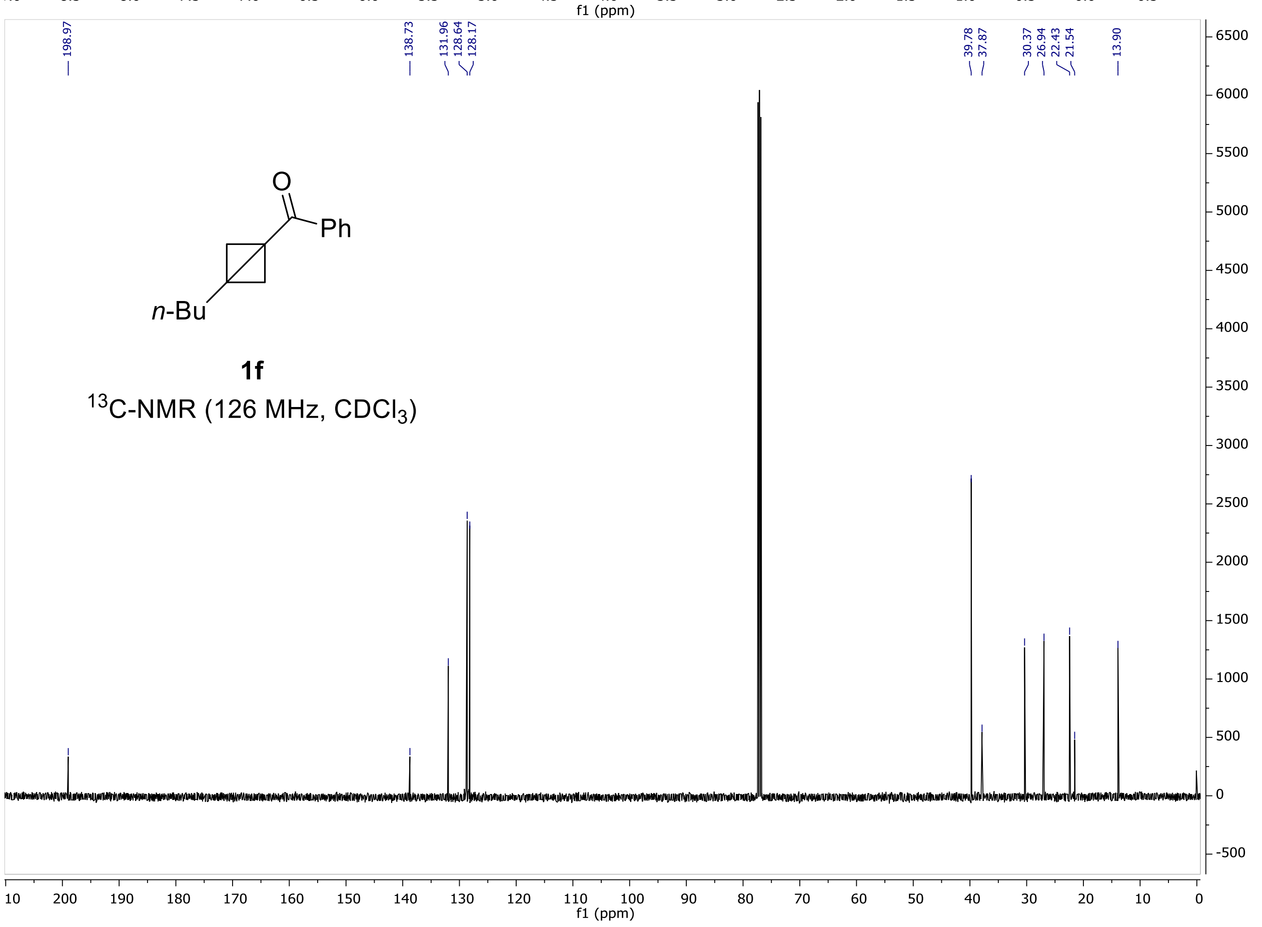

1f

^{13}C-NMR (126 MHz, $CDCl_3$)

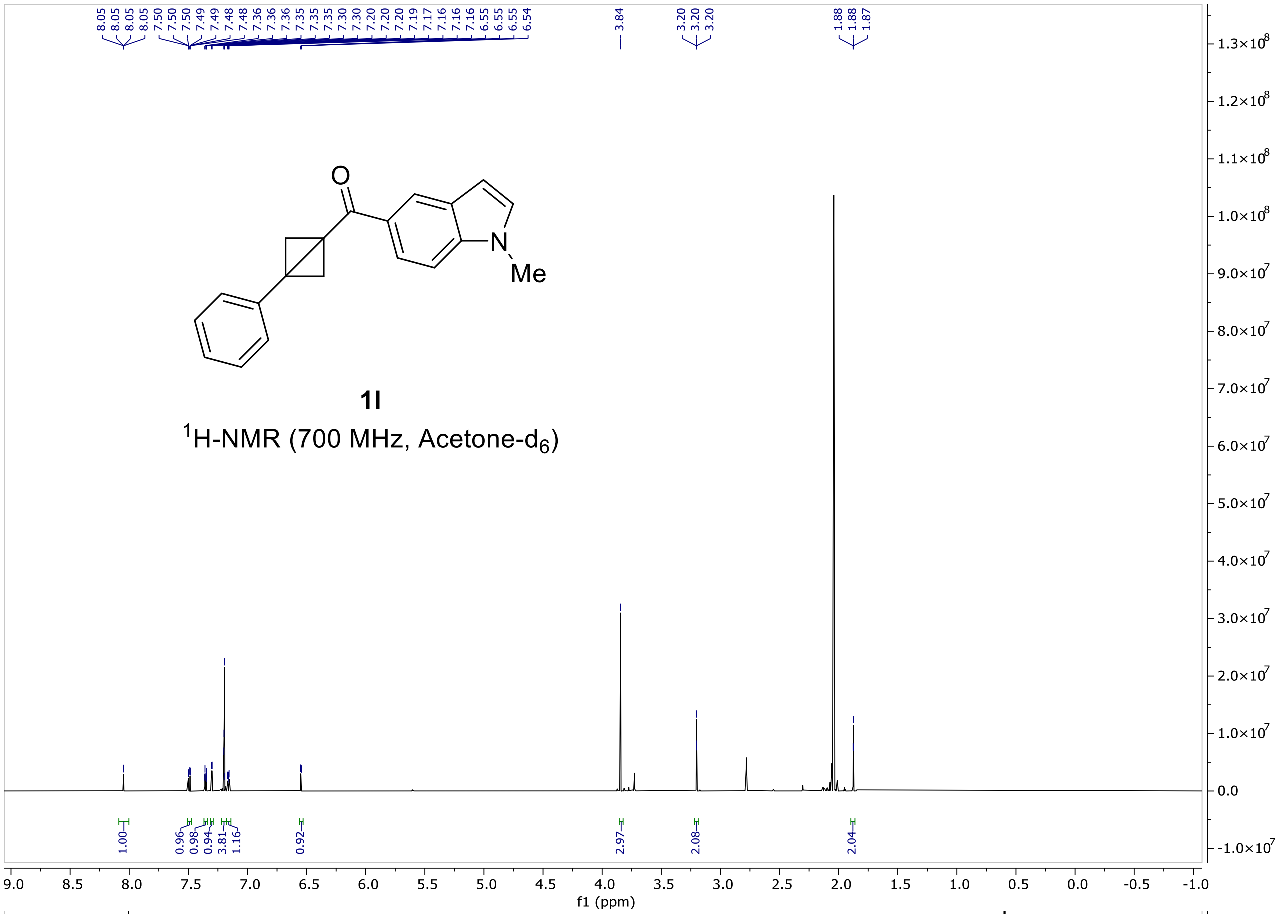

1l

^{1}H-NMR (700 MHz, Acetone-d$_6$)

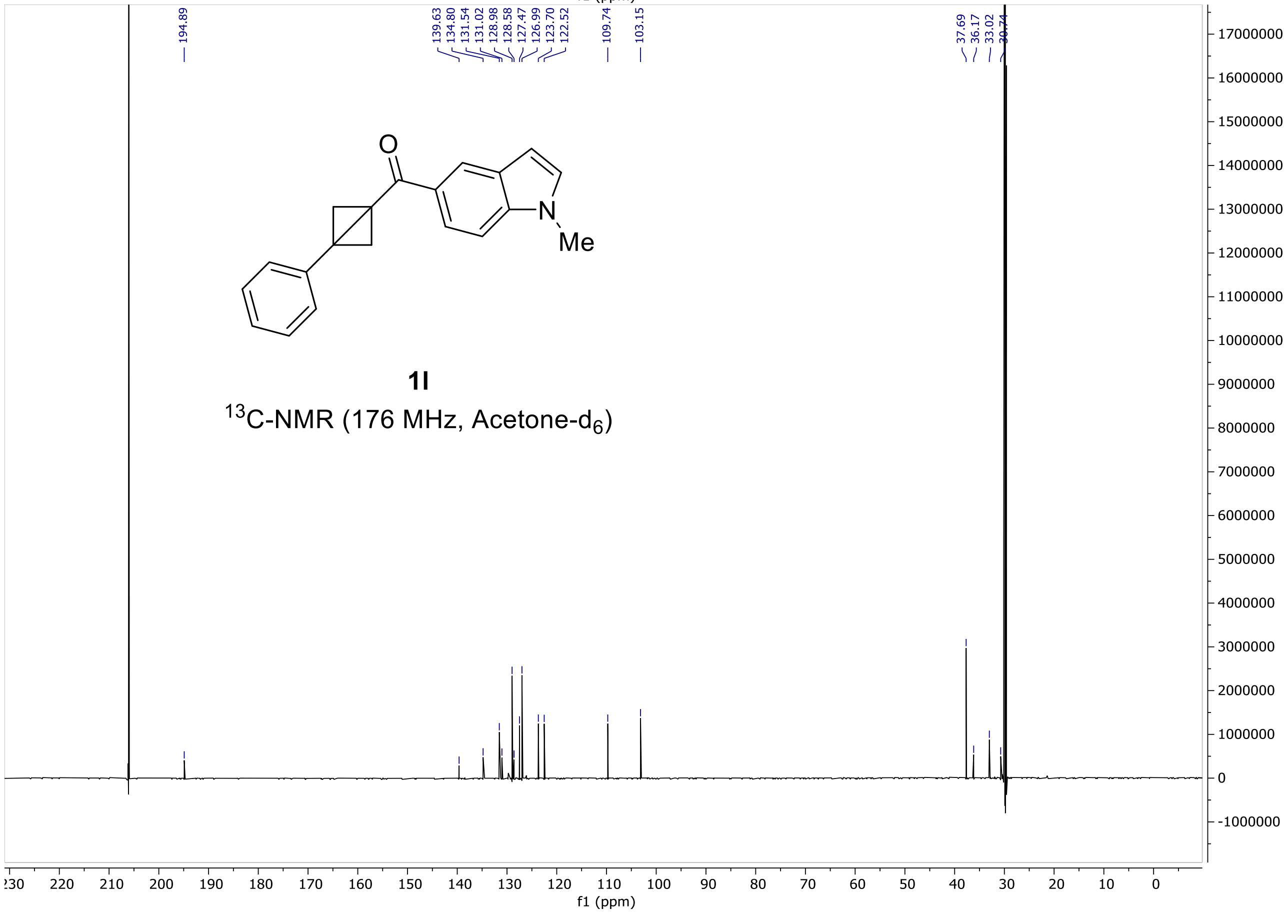

1l

^{13}C-NMR (176 MHz, Acetone-d$_6$)

Thioketone Products

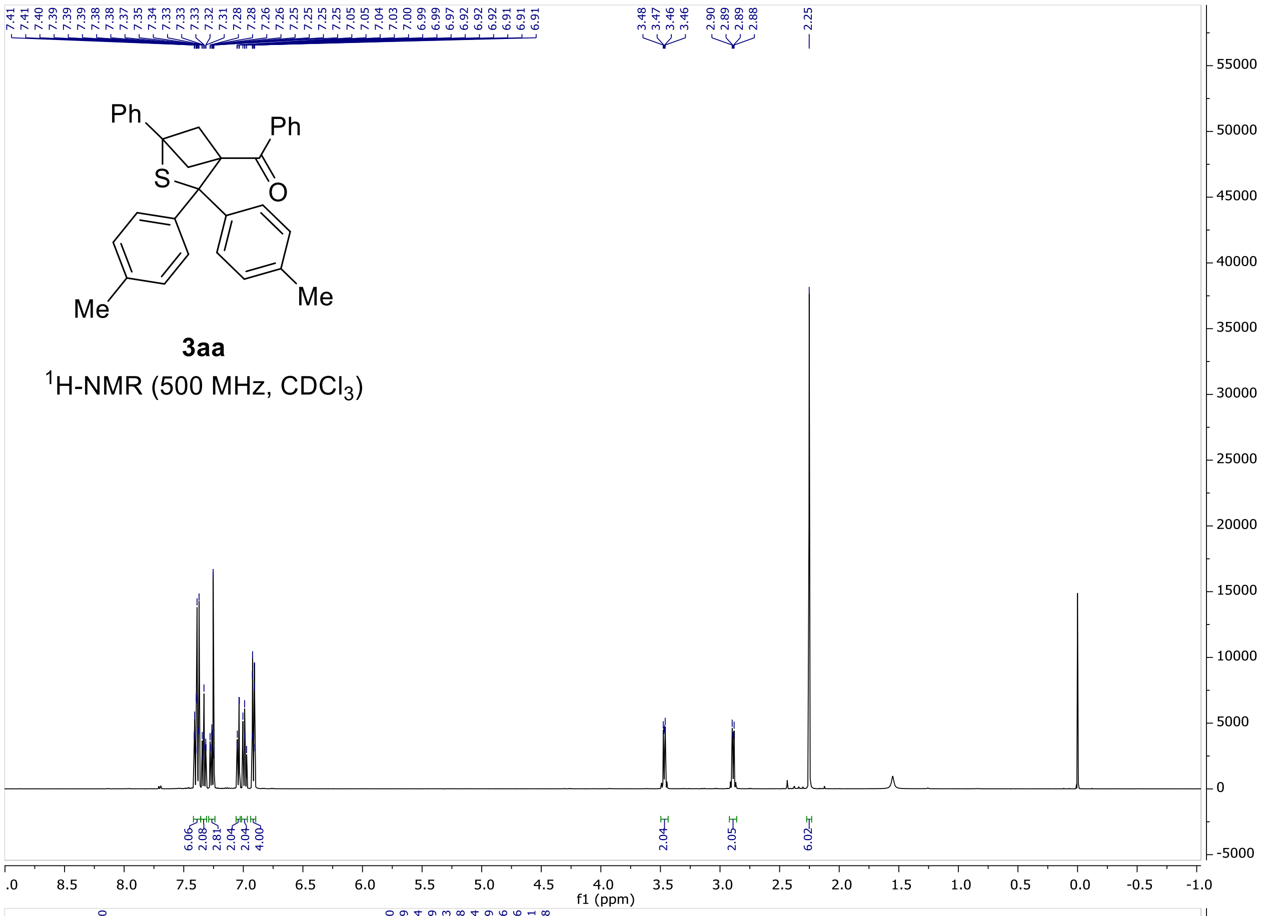

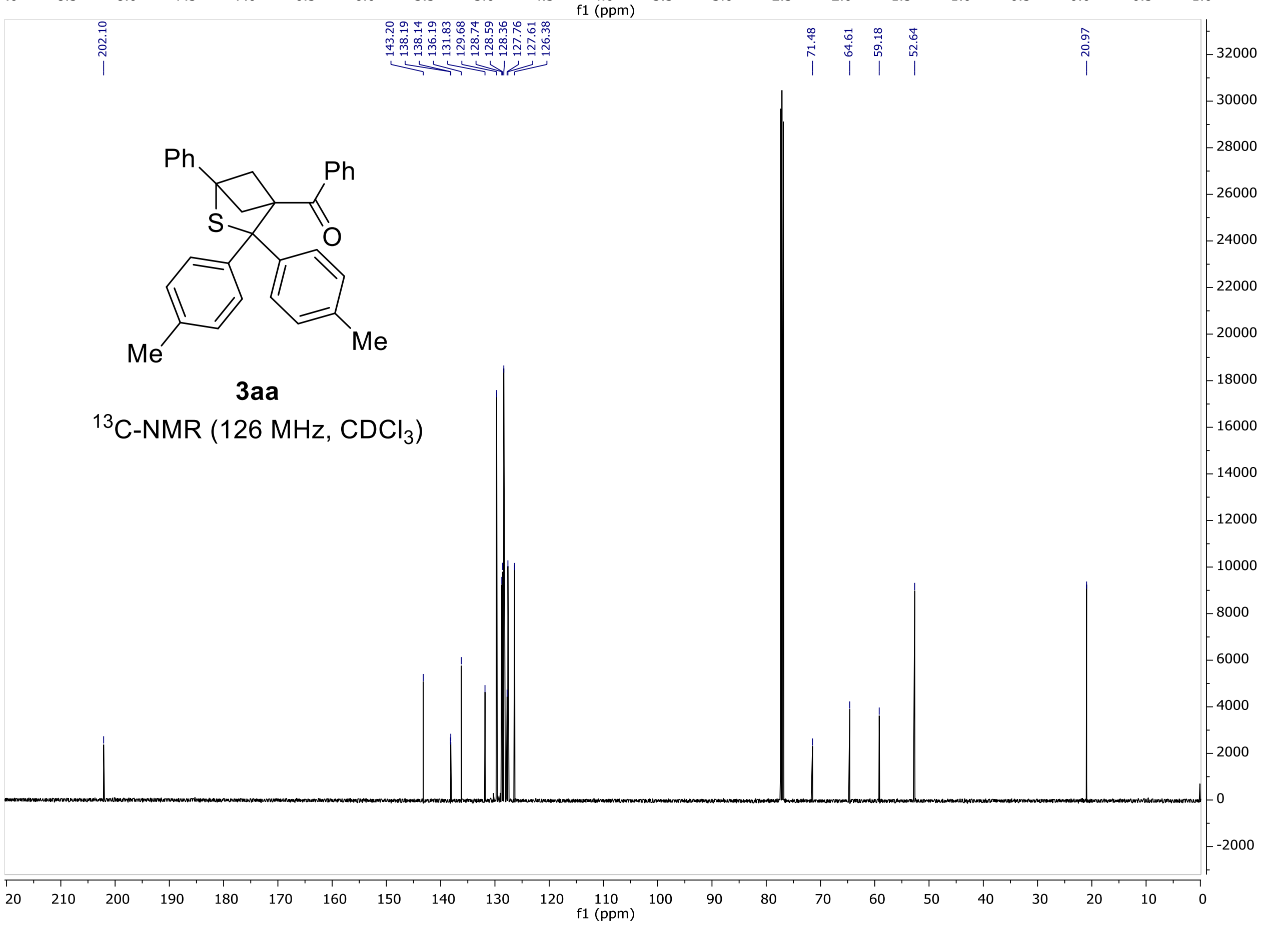

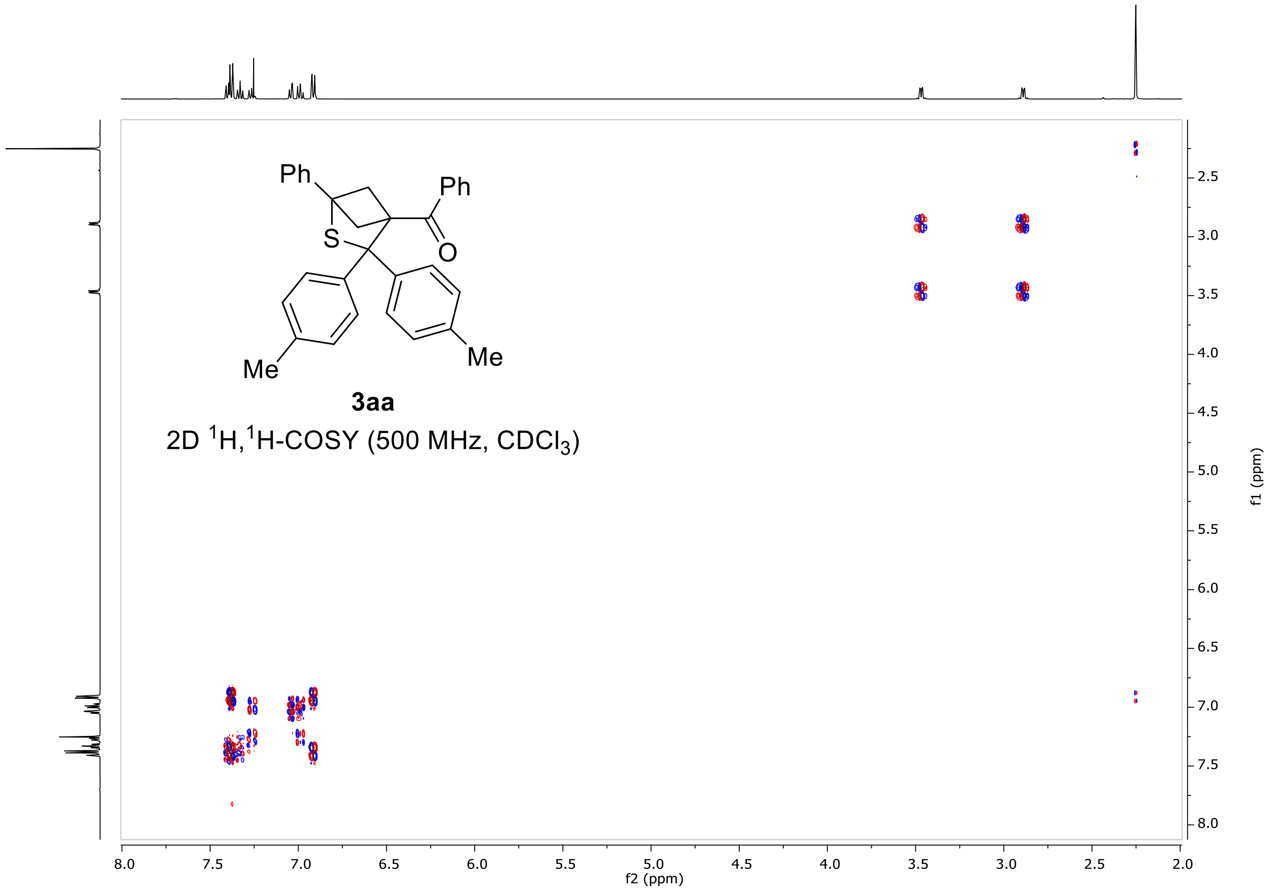
Ph
Ph
S
O
Me
Me
3aa
2D ^{1}H,^{1}H-COSY (500 MHz, $CDCl_3$)
f2 (ppm)
f1 (ppm)

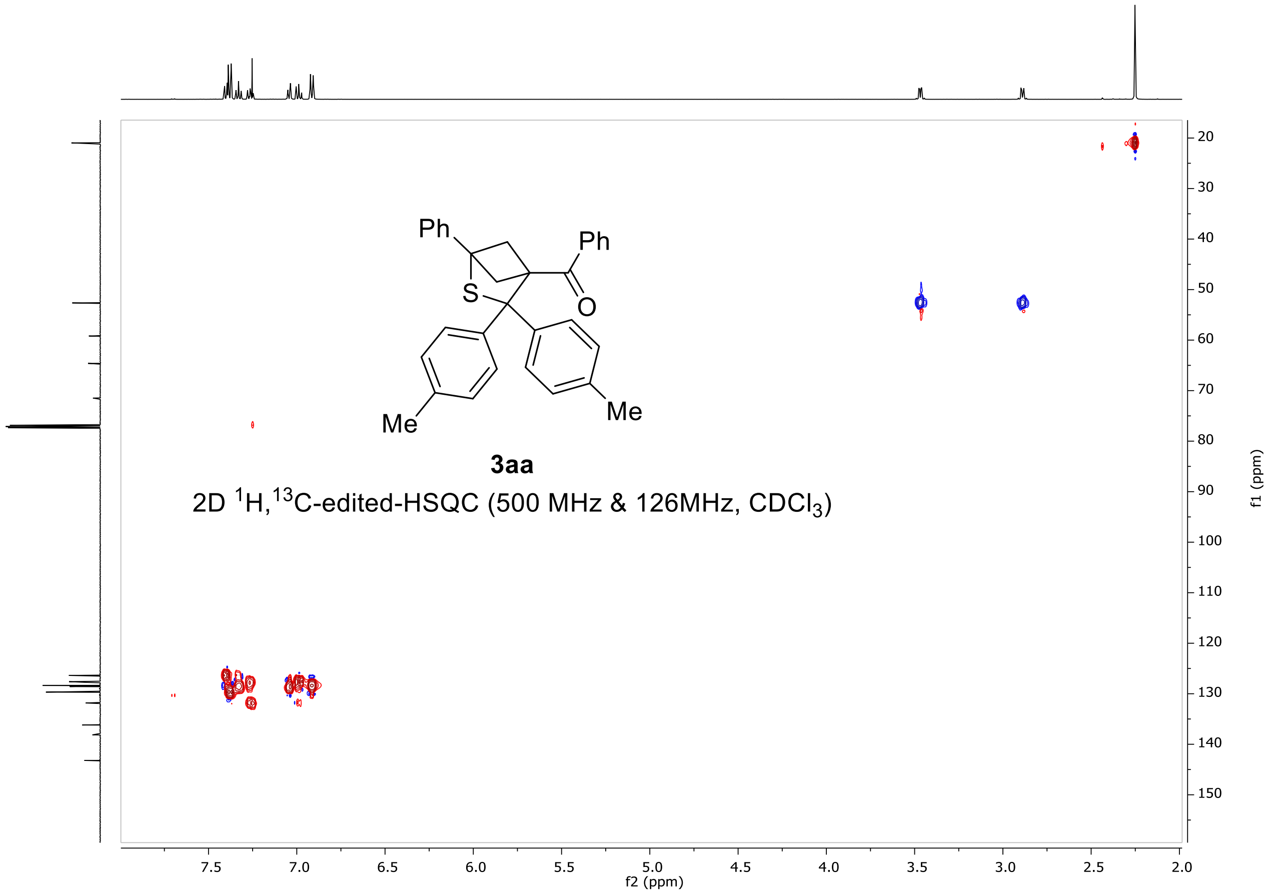
Ph
Ph
S
O
Me
Me
3aa
2D ^{1}H,^{13}C-edited-HSQC (500 MHz & 126MHz, $CDCl_3$)
f2 (ppm)
f1 (ppm)

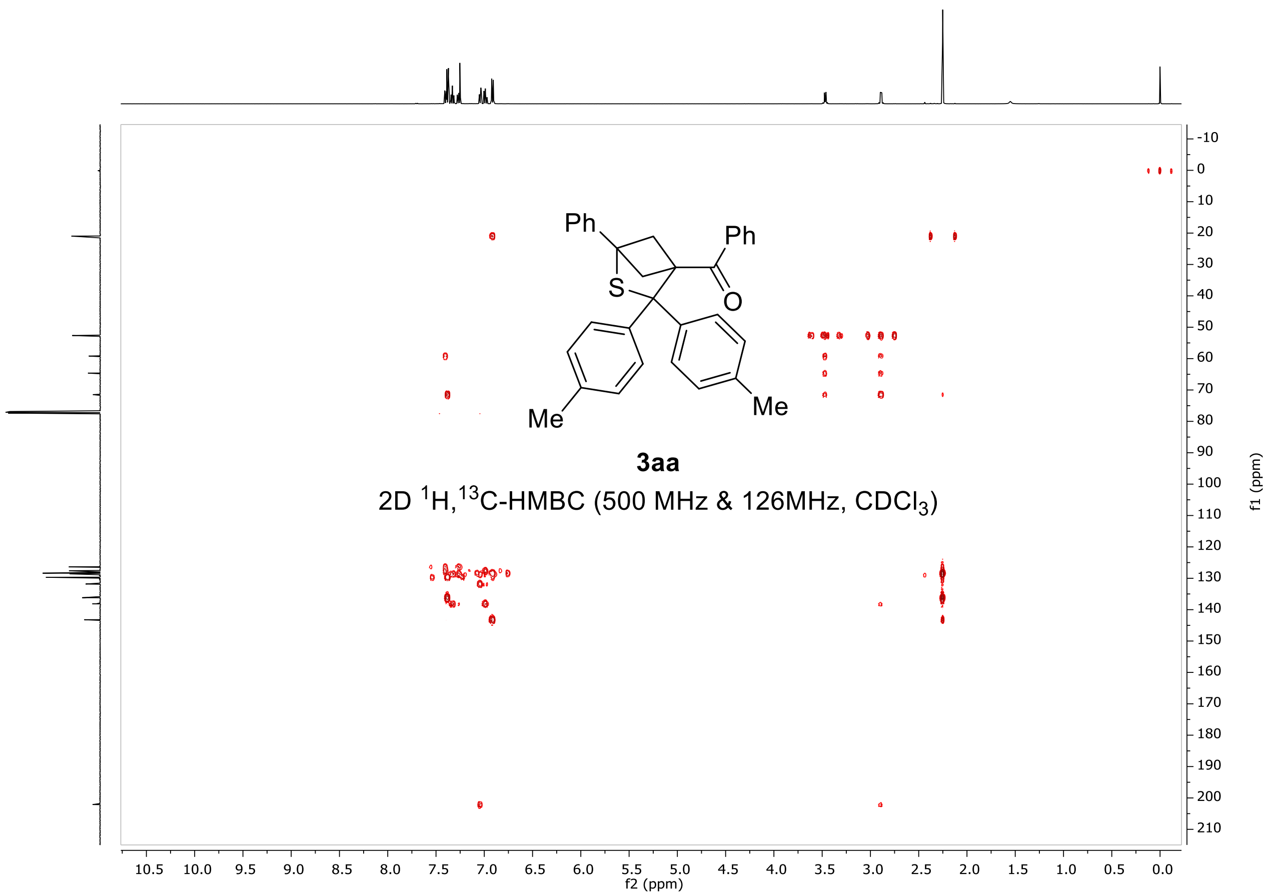

Ph
Ph
S
O
Me
Me
3aa
2D ^{1}H,^{13}C-HMBC (500 MHz & 126MHz, CDCl$_3$)
f1 (ppm)
f2 (ppm)

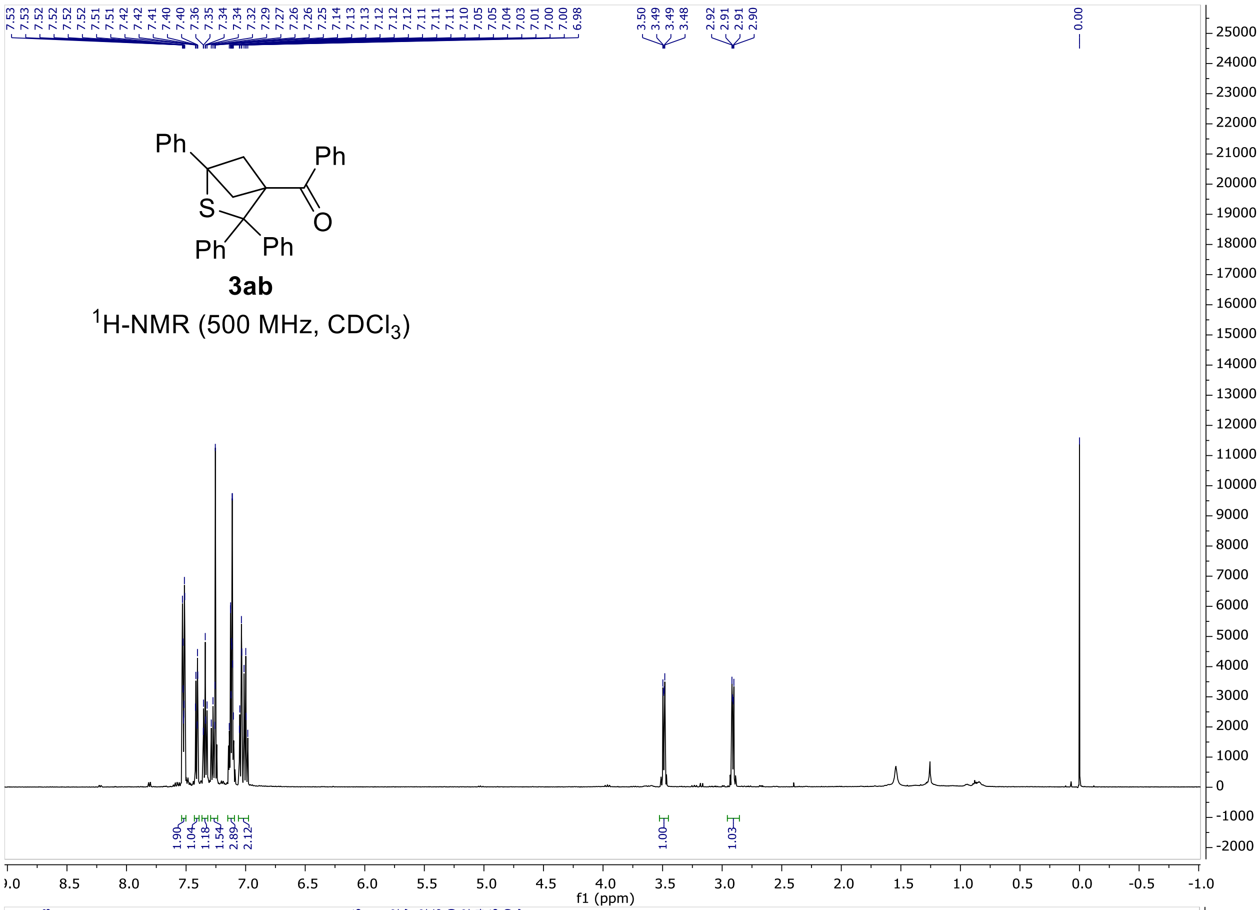
Ph
Ph
S
O
Ph
Ph
3ab
1H-NMR (500 MHz, CDCl3)
7.53 7.53 7.52 7.52 7.52 7.52 7.51 7.51 7.42 7.42 7.41 7.40 7.40 7.36 7.35 7.34 7.34 7.32 7.29 7.27 7.26 7.26 7.25 7.14 7.13 7.13 7.12 7.12 7.11 7.11 7.11 7.10 7.05 7.05 7.04 7.03 7.01 7.00 7.00 6.98
3.50 3.49 3.49 3.48
2.92 2.91 2.91 2.90
0.00
1.90 1.04 1.18 1.54 2.89 2.12
1.00
1.03
f1 (ppm)

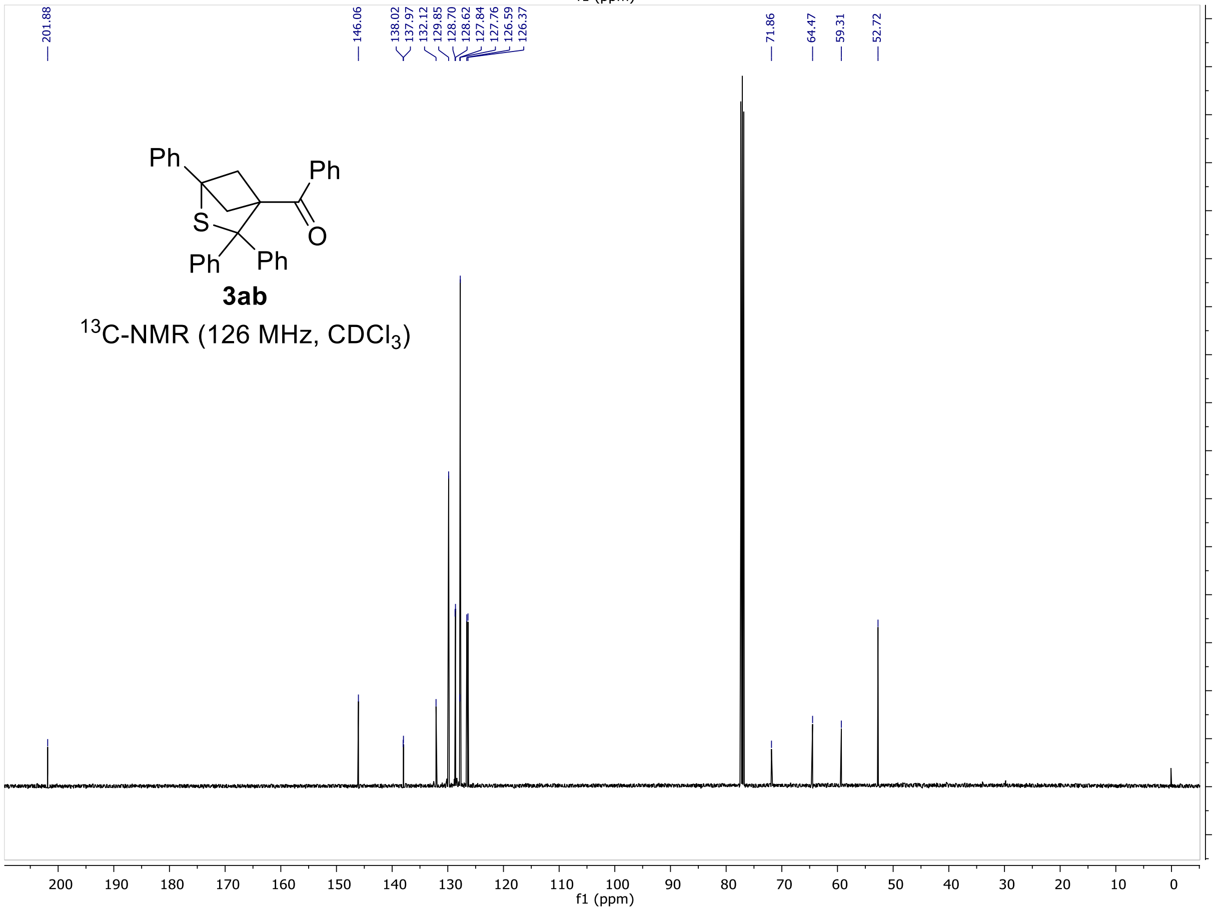
Ph
Ph
S
O
Ph
Ph
3ab
13C-NMR (126 MHz, CDCl3)
201.88
146.06
138.02 137.97 132.12 129.85 128.70 128.62 127.84 127.76 126.59 126.37
71.86
64.47
59.31
52.72
f1 (ppm)

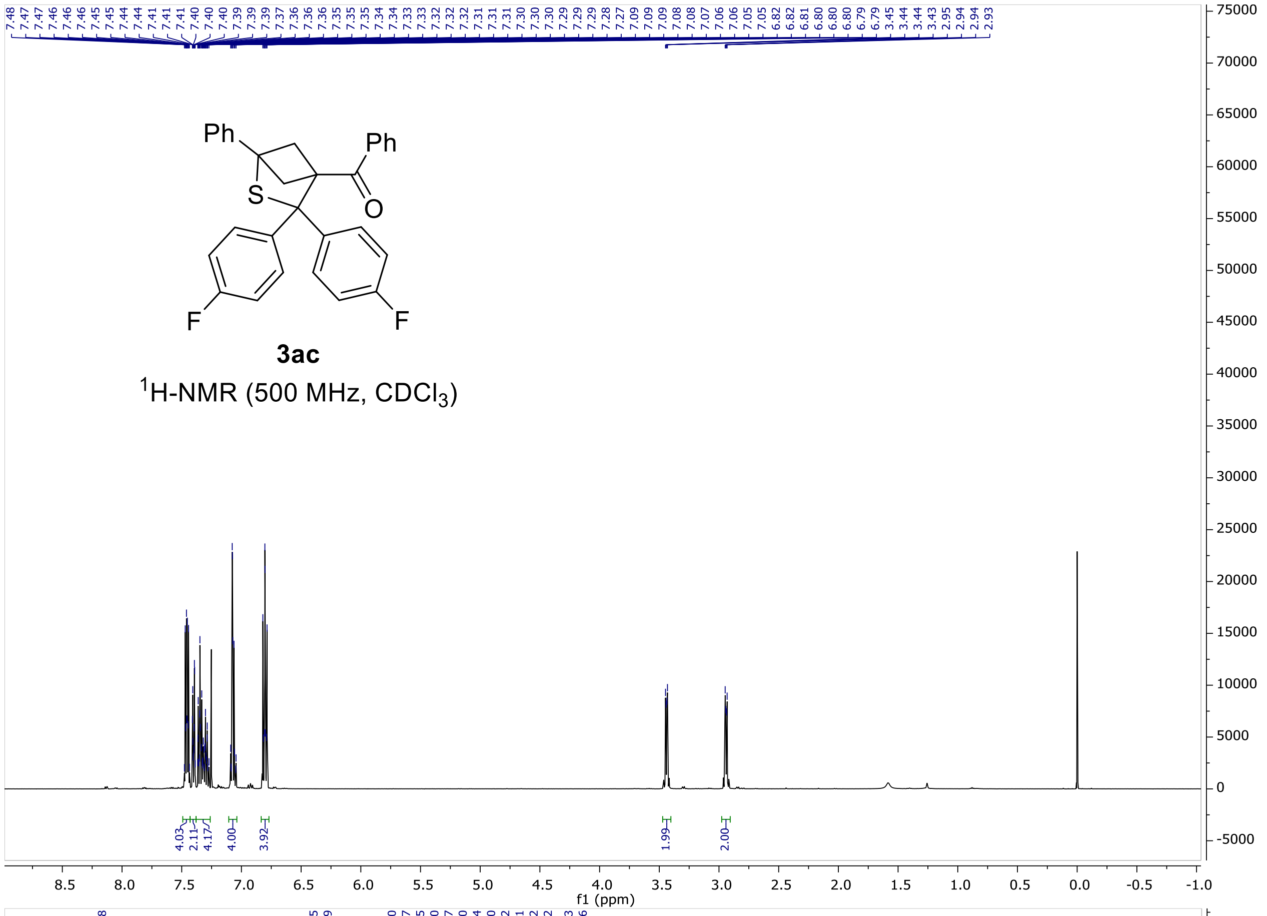
Ph
Ph
S
O
F
F
3ac
1H-NMR (500 MHz, CDCl3)
f1 (ppm)

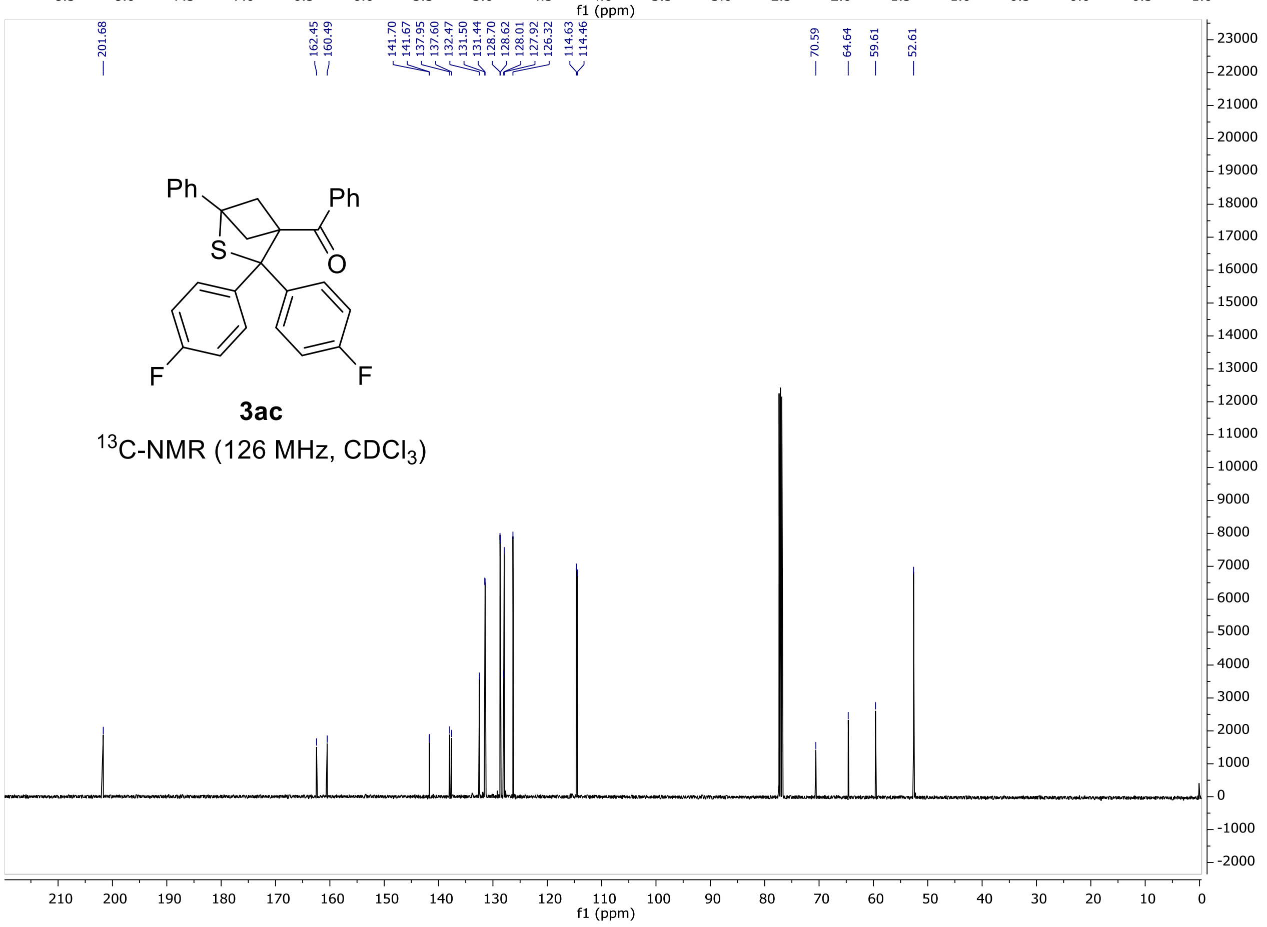
201.68
162.45
160.49
141.70
141.67
137.95
137.60
132.47
131.50
131.44
128.70
128.62
128.01
127.92
126.32
114.63
114.46
70.59
64.64
59.61
52.61
Ph
Ph
S
O
F
F
3ac
13C-NMR (126 MHz, CDCl3)
f1 (ppm)

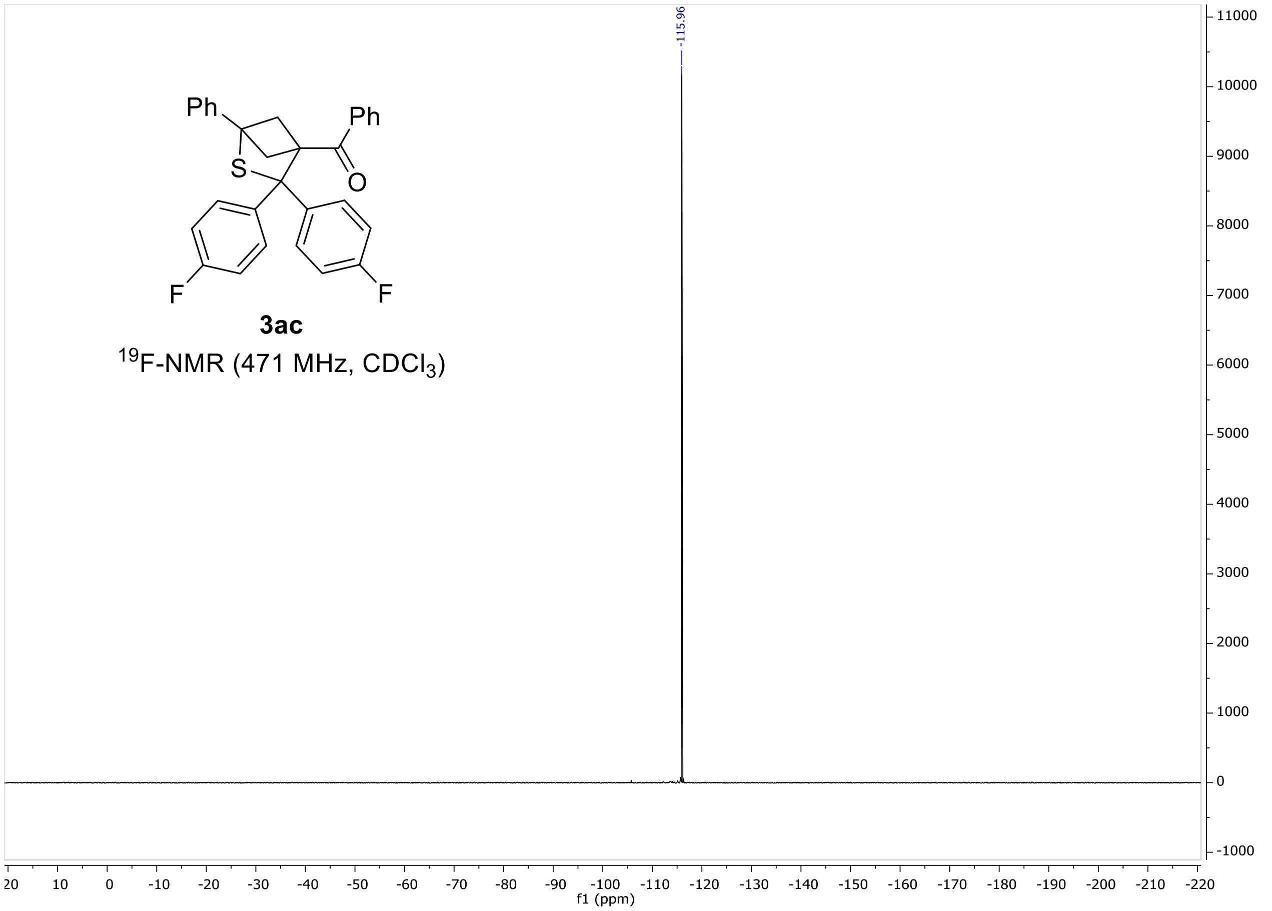
-115.96
Ph
Ph
S
O
F
F
3ac
19F-NMR (471 MHz, CDCl3)
11000
10000
9000
8000
7000
6000
5000
4000
3000
2000
1000
0
-1000
20 10 0 -10 -20 -30 -40 -50 -60 -70 -80 -90 -100 -110 -120 -130 -140 -150 -160 -170 -180 -190 -200 -210 -220
f1 (ppm)

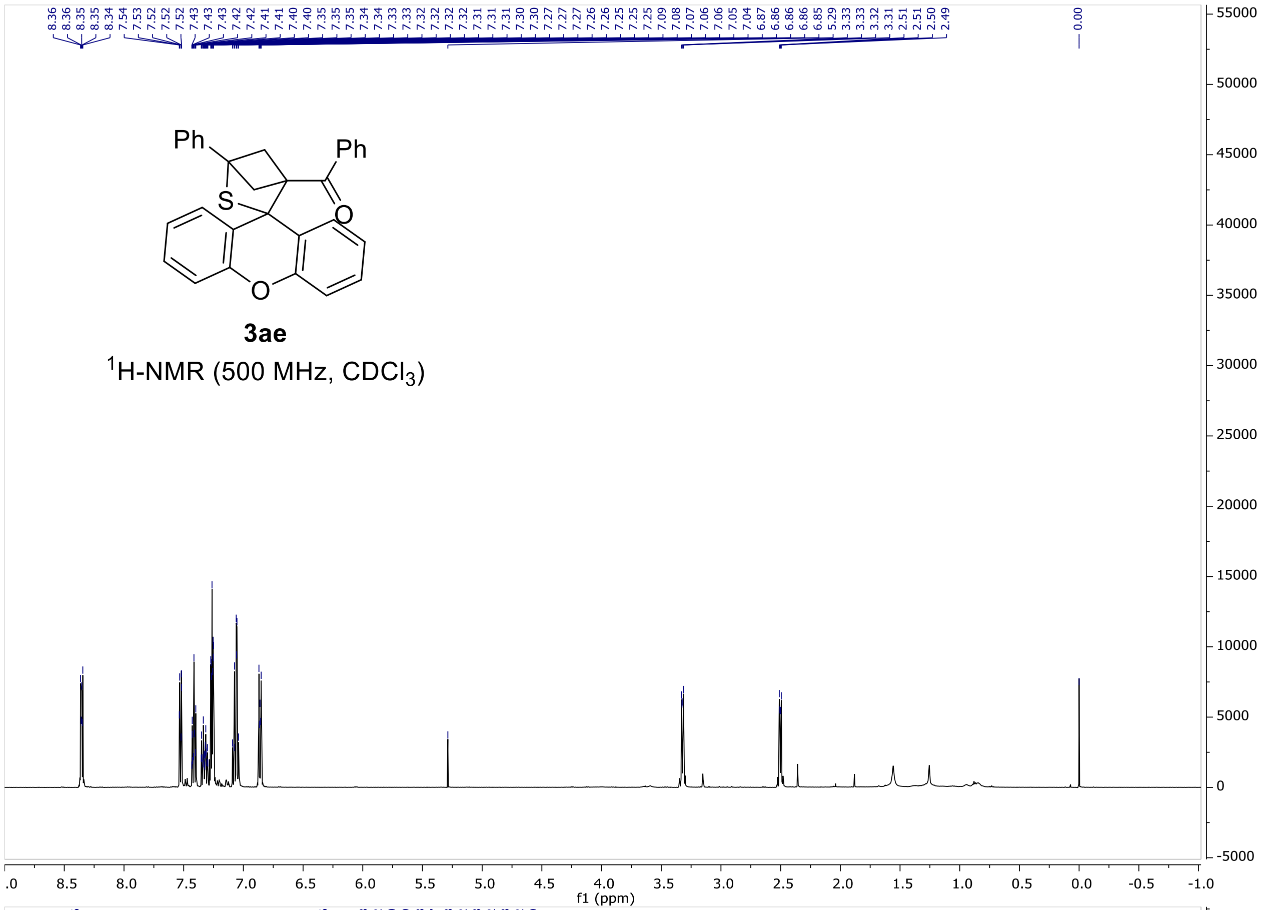
Ph
Ph
S
O
O
3ae
1H-NMR (500 MHz, CDCl3)
f1 (ppm)

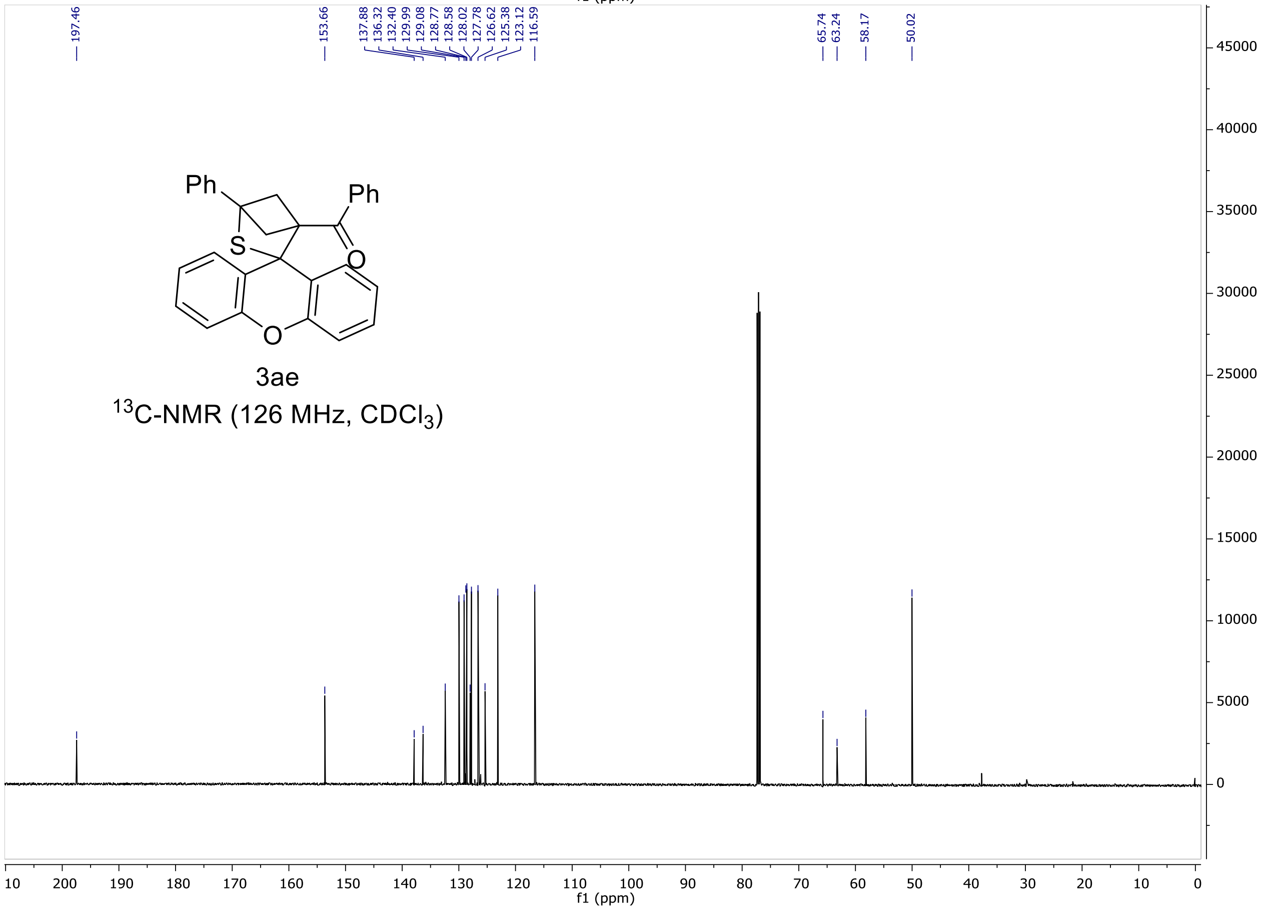
197.46
153.66
137.88
136.32
132.40
129.99
129.08
128.77
128.58
128.02
127.78
126.62
125.38
123.12
116.59
65.74
63.24
58.17
50.02
Ph
Ph
S
O
O
3ae
13C-NMR (126 MHz, CDCl3)
f1 (ppm)

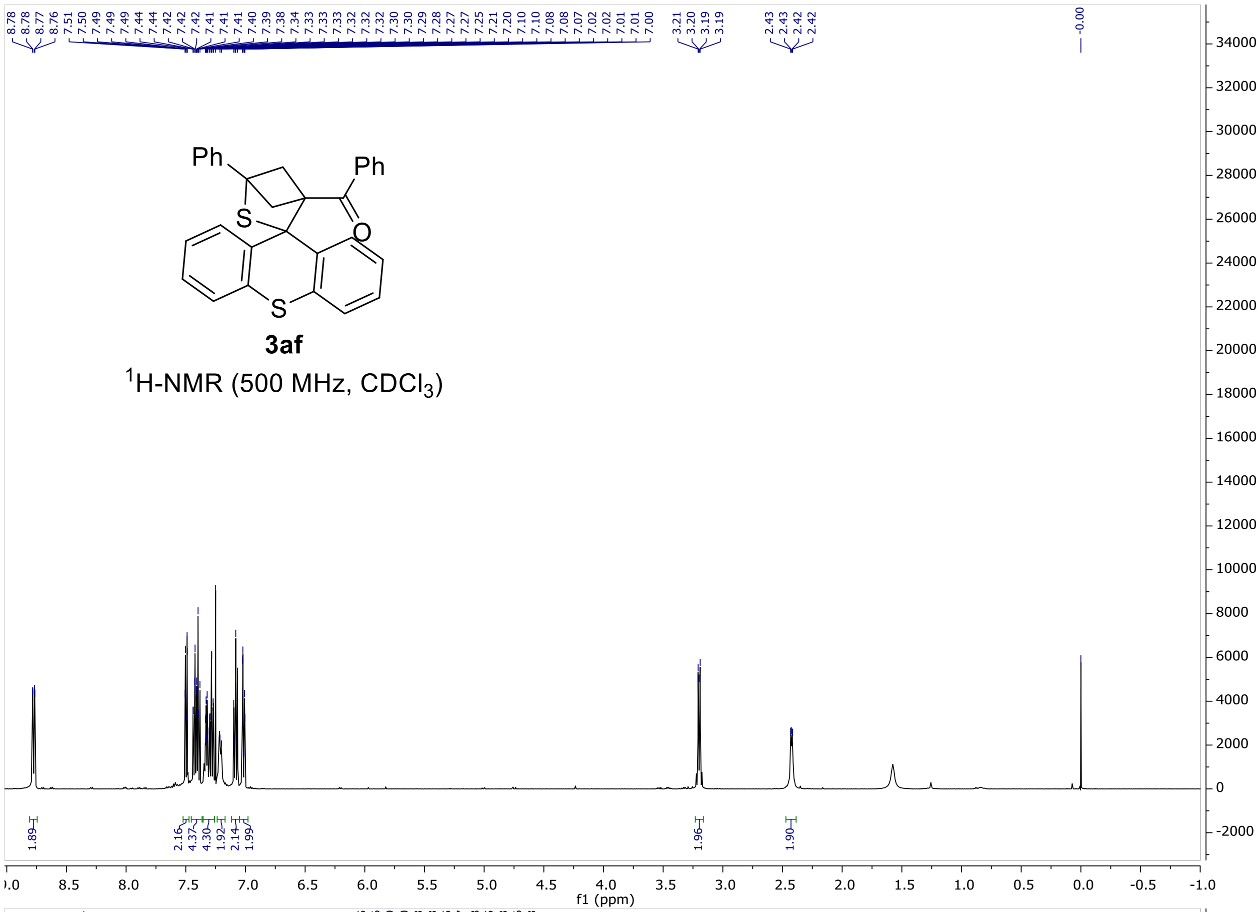
Ph
Ph
S
O
S
3af
1H-NMR (500 MHz, CDCl3)
f1 (ppm)

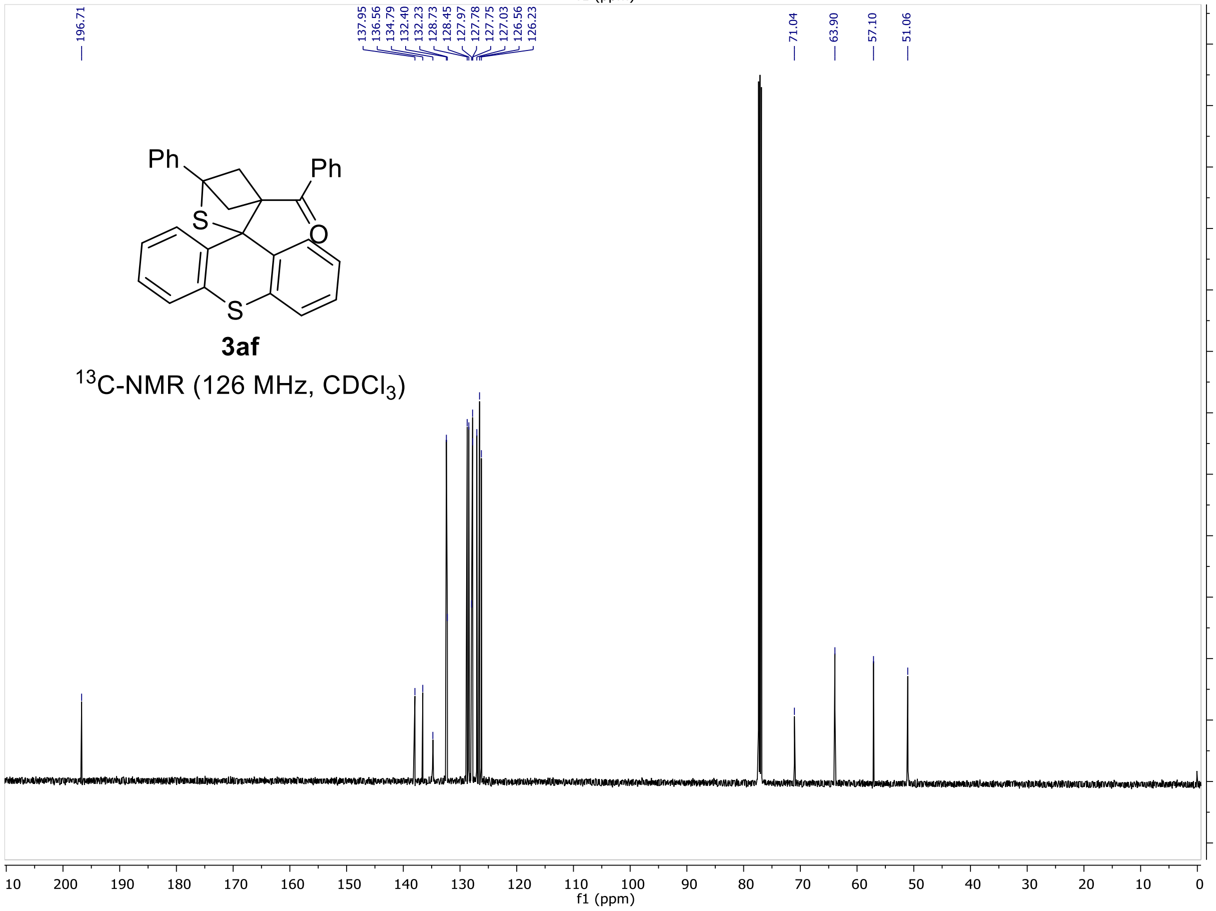
196.71
137.95
136.56
134.79
132.40
132.23
128.73
128.45
127.97
127.78
127.75
127.03
126.56
126.23
71.04
63.90
57.10
51.06
Ph
Ph
S
O
S
3af
13C-NMR (126 MHz, CDCl3)
f1 (ppm)

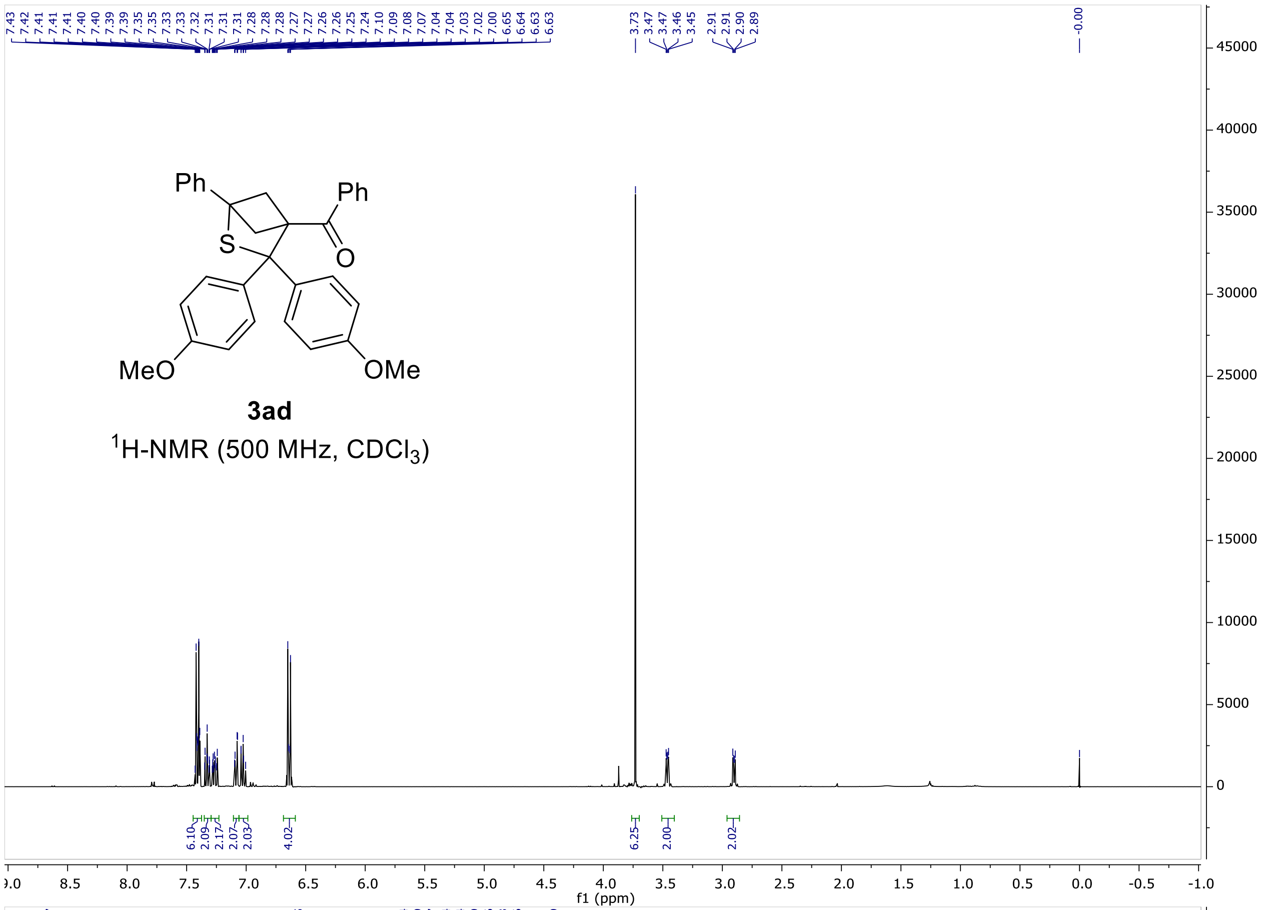

Ph
Ph
S
O
MeO
OMe
3ad
^{1}H-NMR (500 MHz, CDCl$_3$)
f1 (ppm)

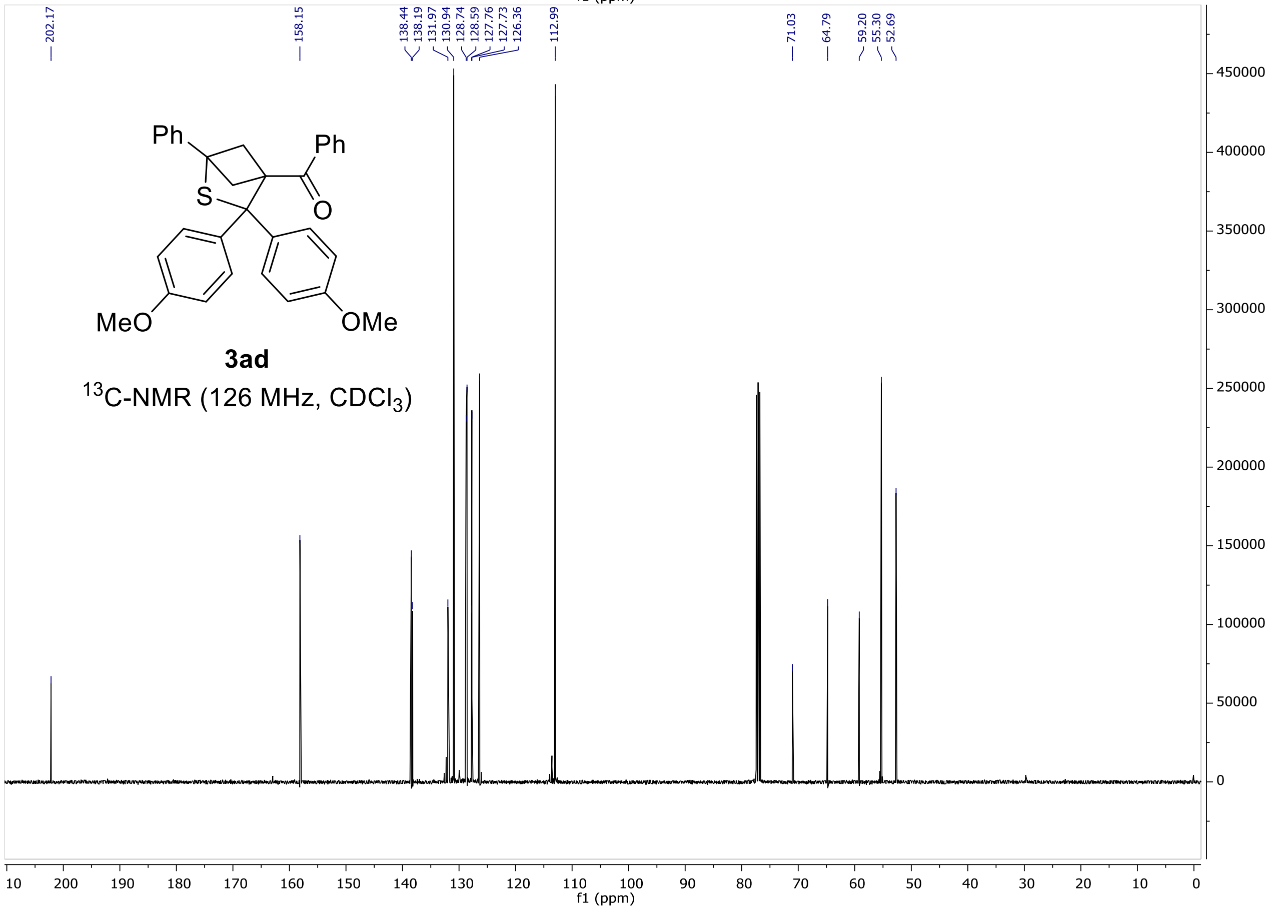

Ph
Ph
S
O
MeO
OMe
3ad
^{13}C-NMR (126 MHz, CDCl$_3$)
f1 (ppm)

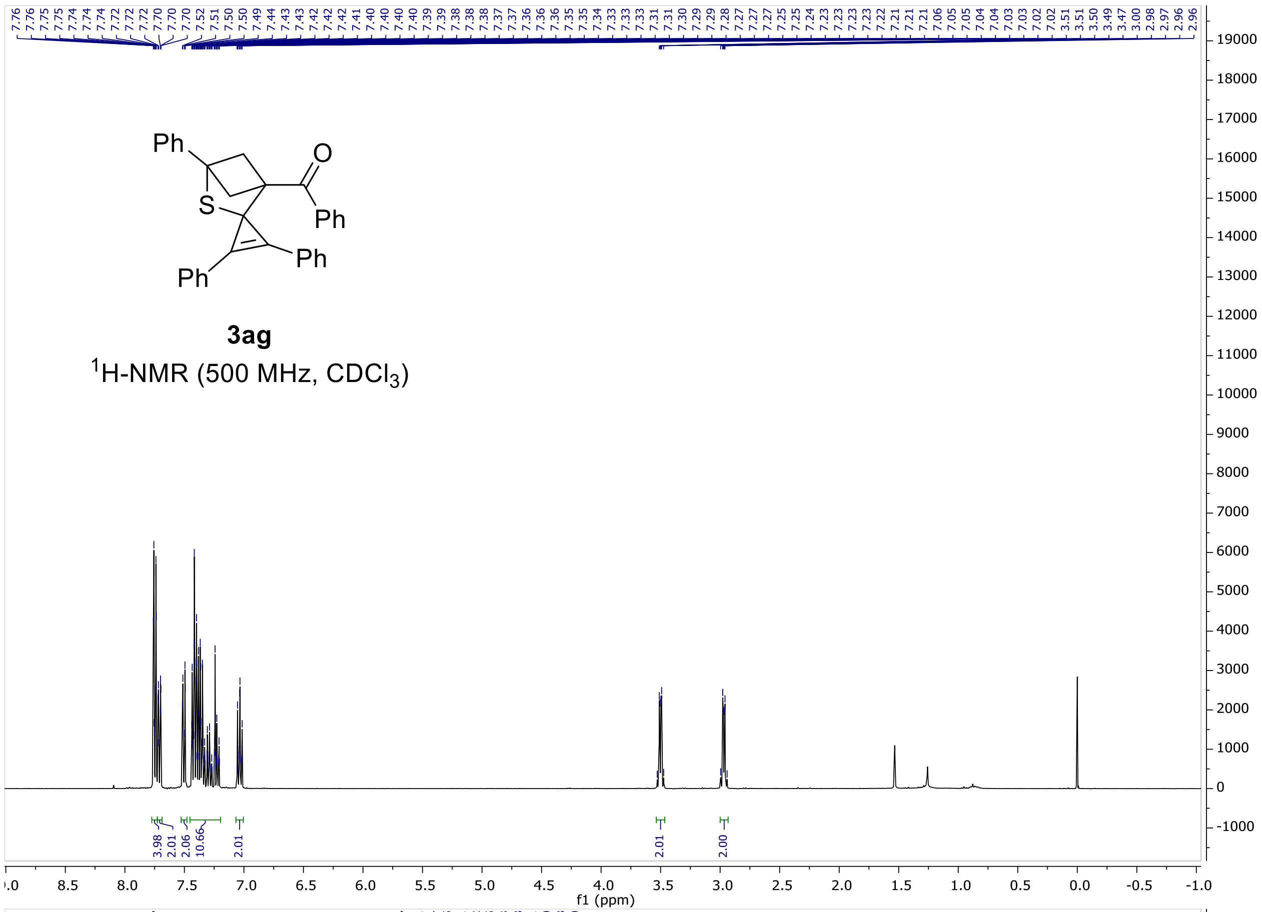
Ph
O
S
Ph
Ph
Ph
3ag
¹H-NMR (500 MHz, CDCl3)
3.98
2.01
2.06
10.66
2.01
2.01
2.00
f1 (ppm)

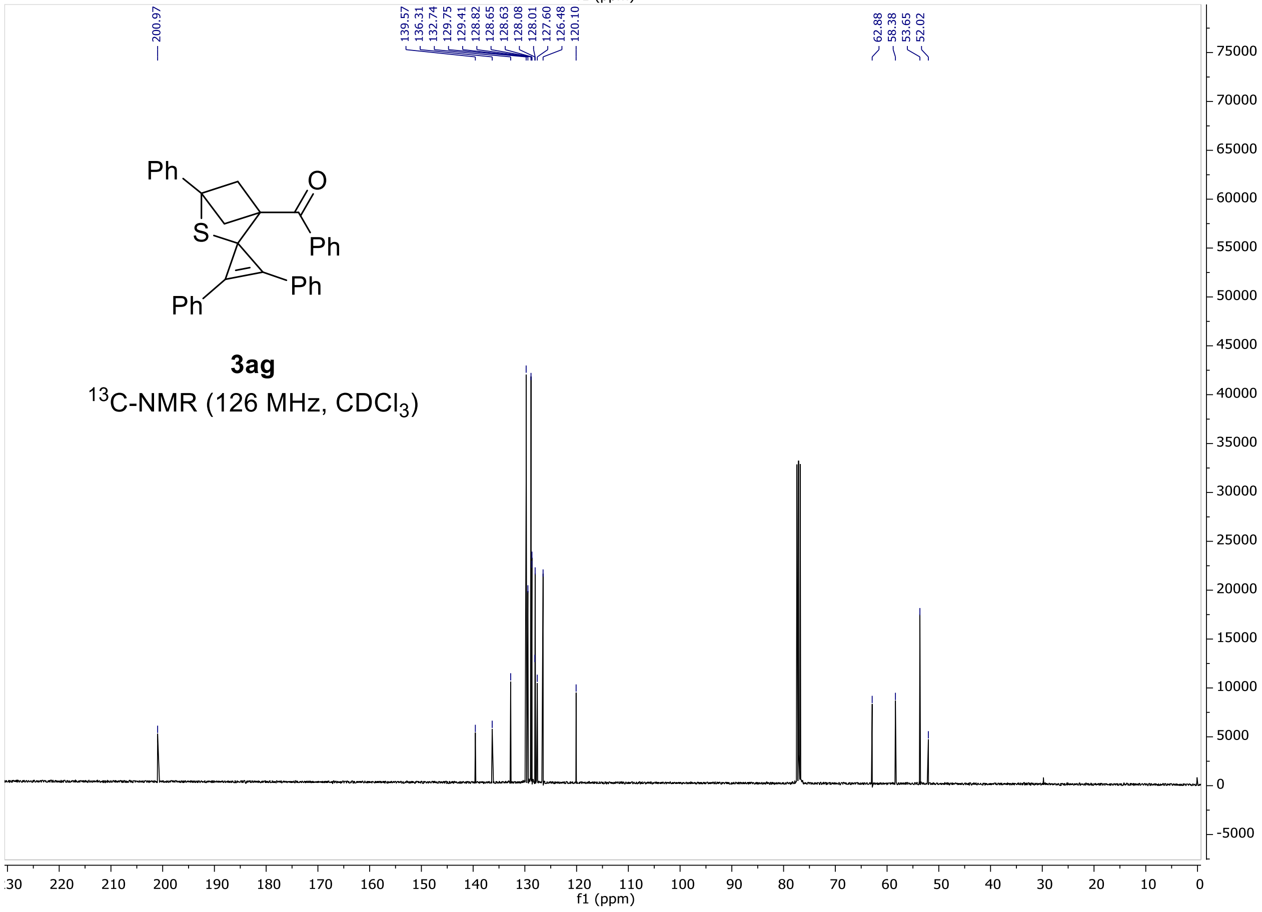
200.97
139.57
136.31
132.74
129.75
129.41
128.82
128.65
128.63
128.08
128.01
127.60
126.48
120.10
62.88
58.38
53.65
52.02
Ph
O
S
Ph
Ph
Ph
3ag
¹³C-NMR (126 MHz, CDCl3)
f1 (ppm)

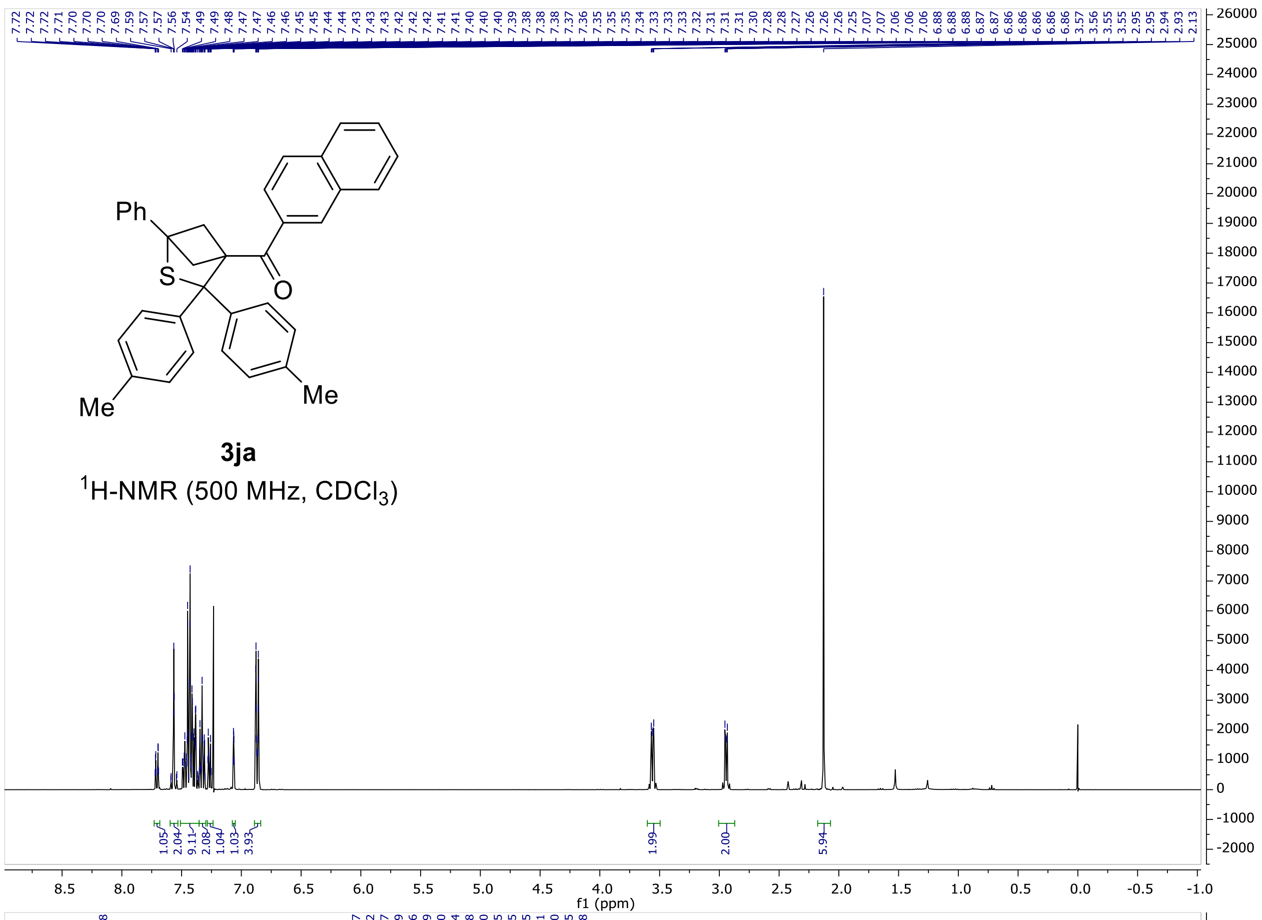
7.72 7.72 7.72 7.71 7.70 7.70 7.70 7.69 7.59 7.57 7.57 7.56 7.54 7.49 7.49 7.48 7.47 7.46 7.46 7.45 7.45 7.44 7.44 7.43 7.43 7.43 7.42 7.42 7.42 7.41 7.41 7.40 7.40 7.40 7.39 7.38 7.38 7.38 7.37 7.36 7.35 7.35 7.34 7.33 7.33 7.33 7.32 7.31 7.31 7.31 7.30 7.28 7.28 7.27 7.26 7.26 7.25 7.07 7.07 7.06 7.06 7.06 6.88 6.88 6.88 6.87 6.87 6.86 6.86 6.86 6.86 6.86 3.57 3.56 3.56 3.55 3.55 2.95 2.95 2.94 2.93 2.93 2.13
Ph
S
O
Me
Me
3ja
^{1}H-NMR (500 MHz, CDCl$_3$)
1.05 2.04 9.11 2.08 1.04 1.03 3.93 1.99 2.00 5.94
f1 (ppm)

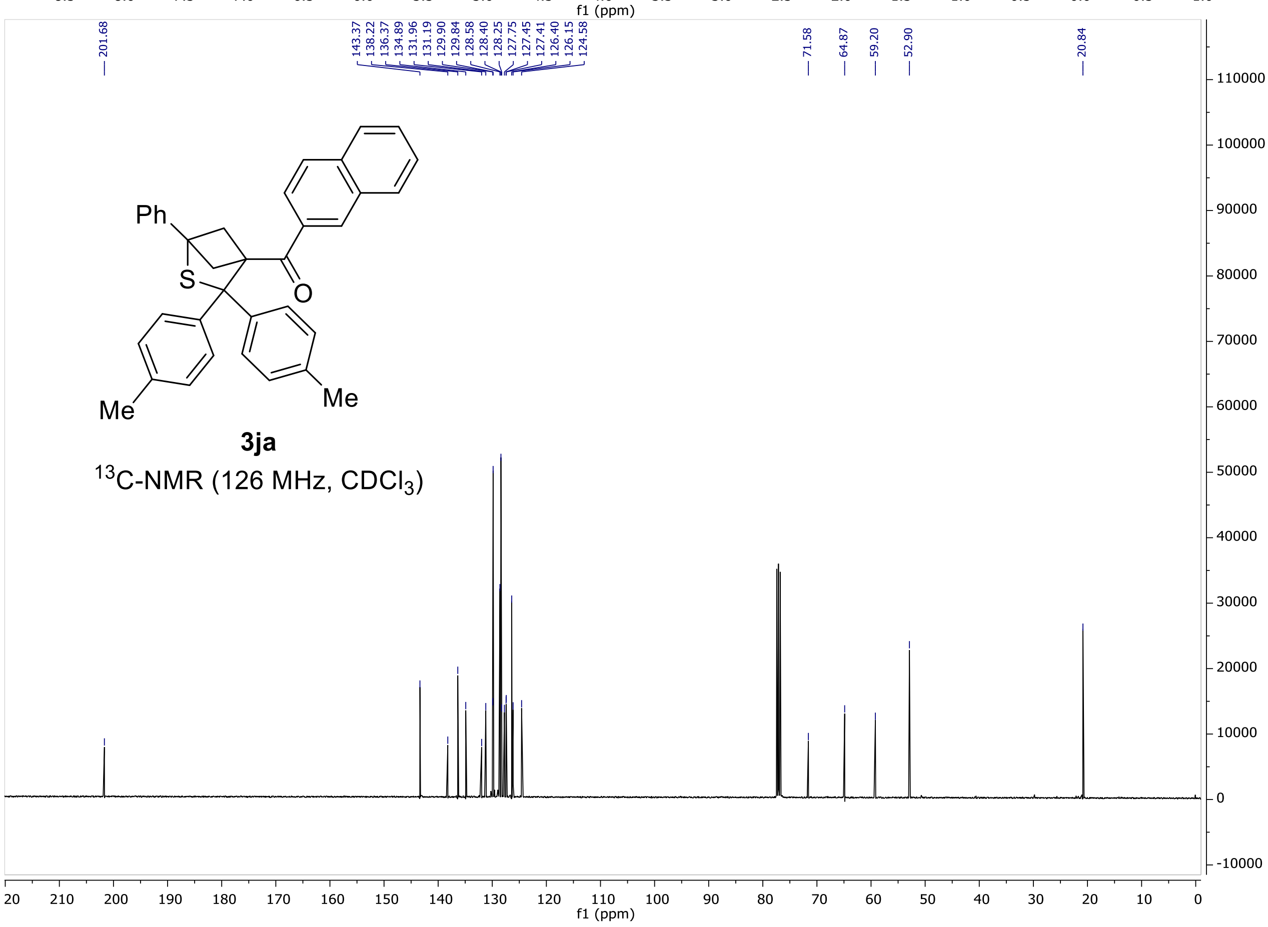
201.68
143.37 138.22 136.37 134.89 131.96 131.19 129.90 129.84 128.58 128.40 128.25 127.75 127.45 127.41 126.40 126.15 124.58
71.58 64.87 59.20 52.90 20.84
Ph
S
O
Me
Me
3ja
^{13}C-NMR (126 MHz, CDCl$_3$)
f1 (ppm)

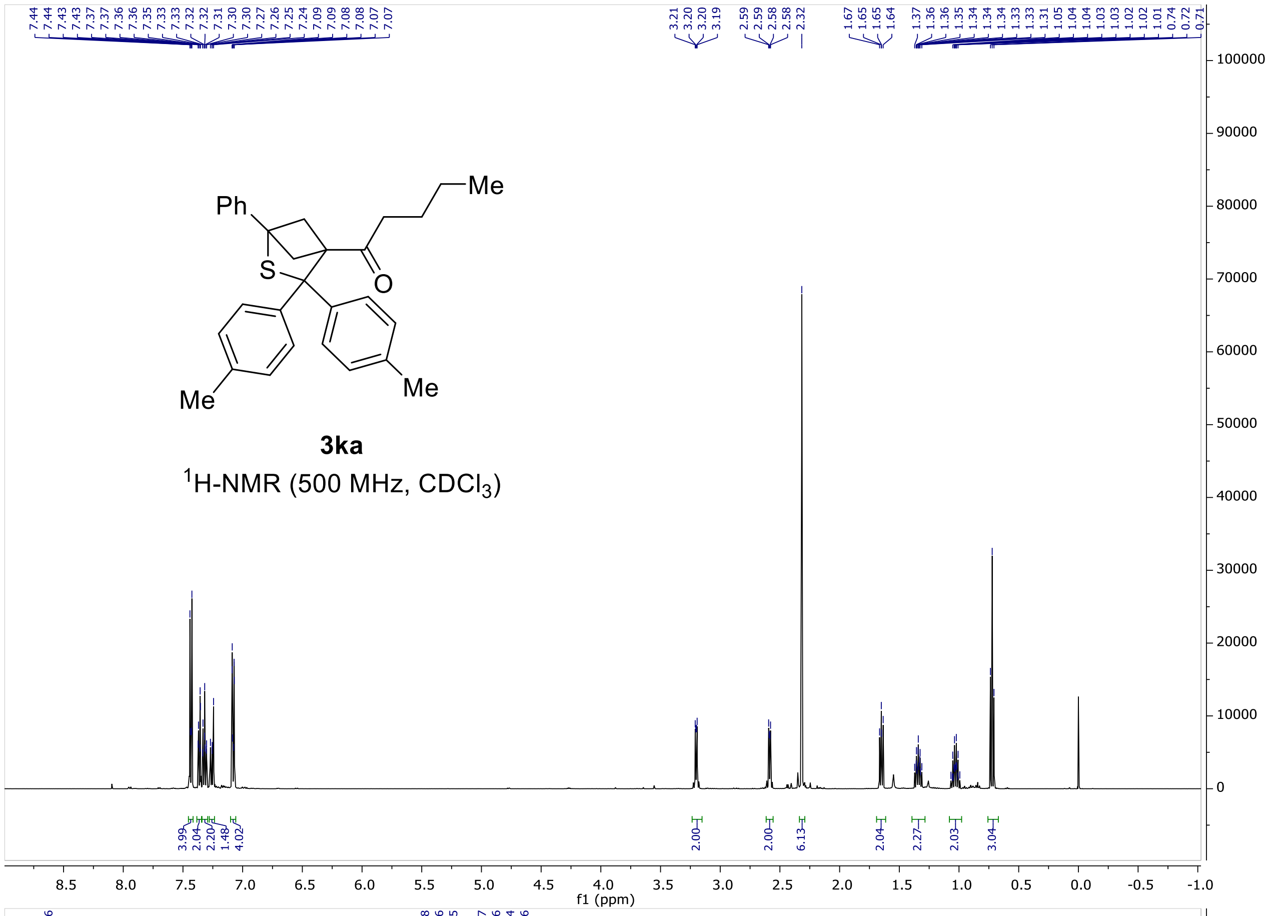
Ph
S
O
Me
Me
Me
3ka
1H-NMR (500 MHz, CDCl3)
f1 (ppm)

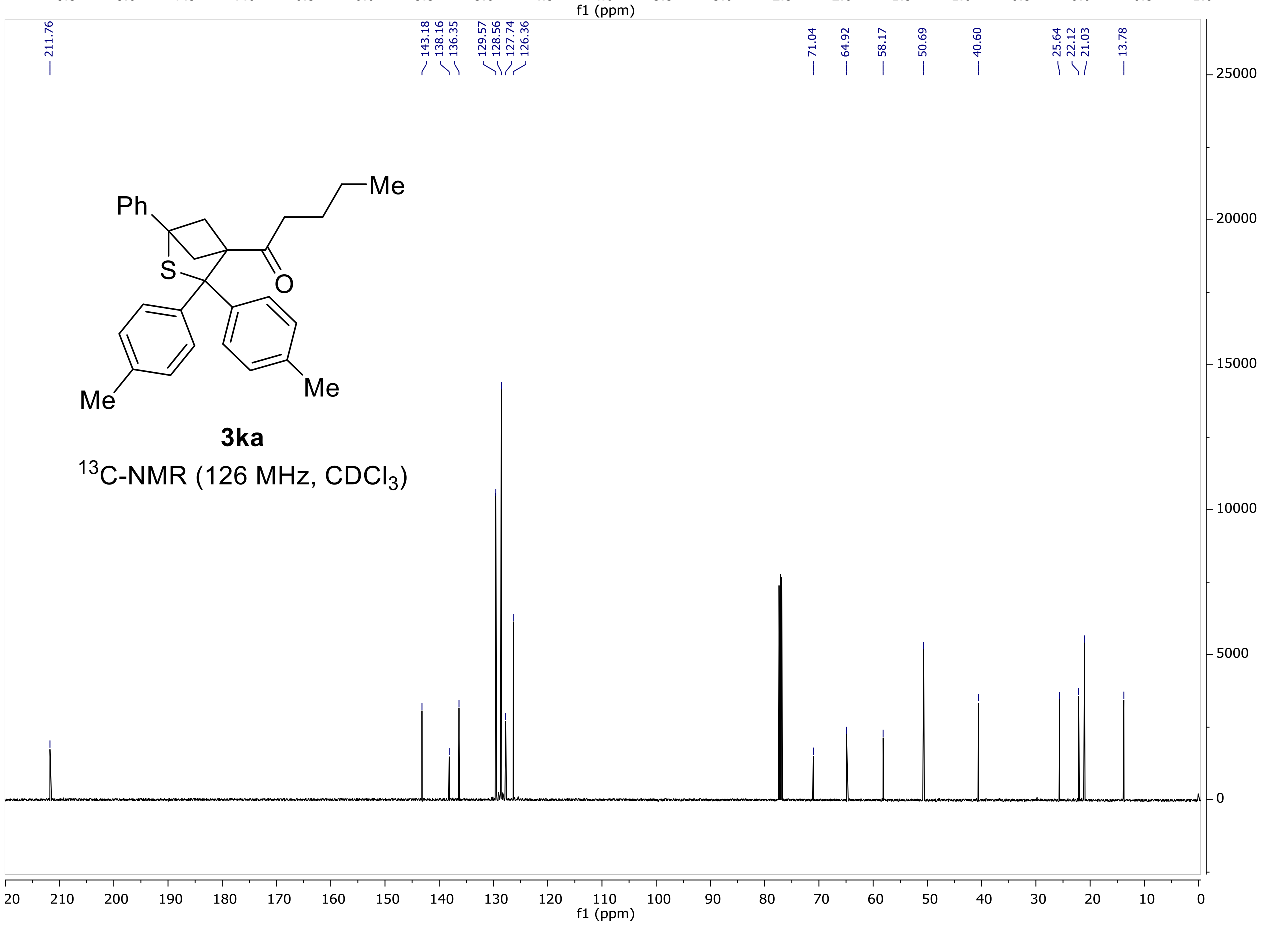
Ph
S
O
Me
Me
Me
3ka
13C-NMR (126 MHz, CDCl3)
f1 (ppm)

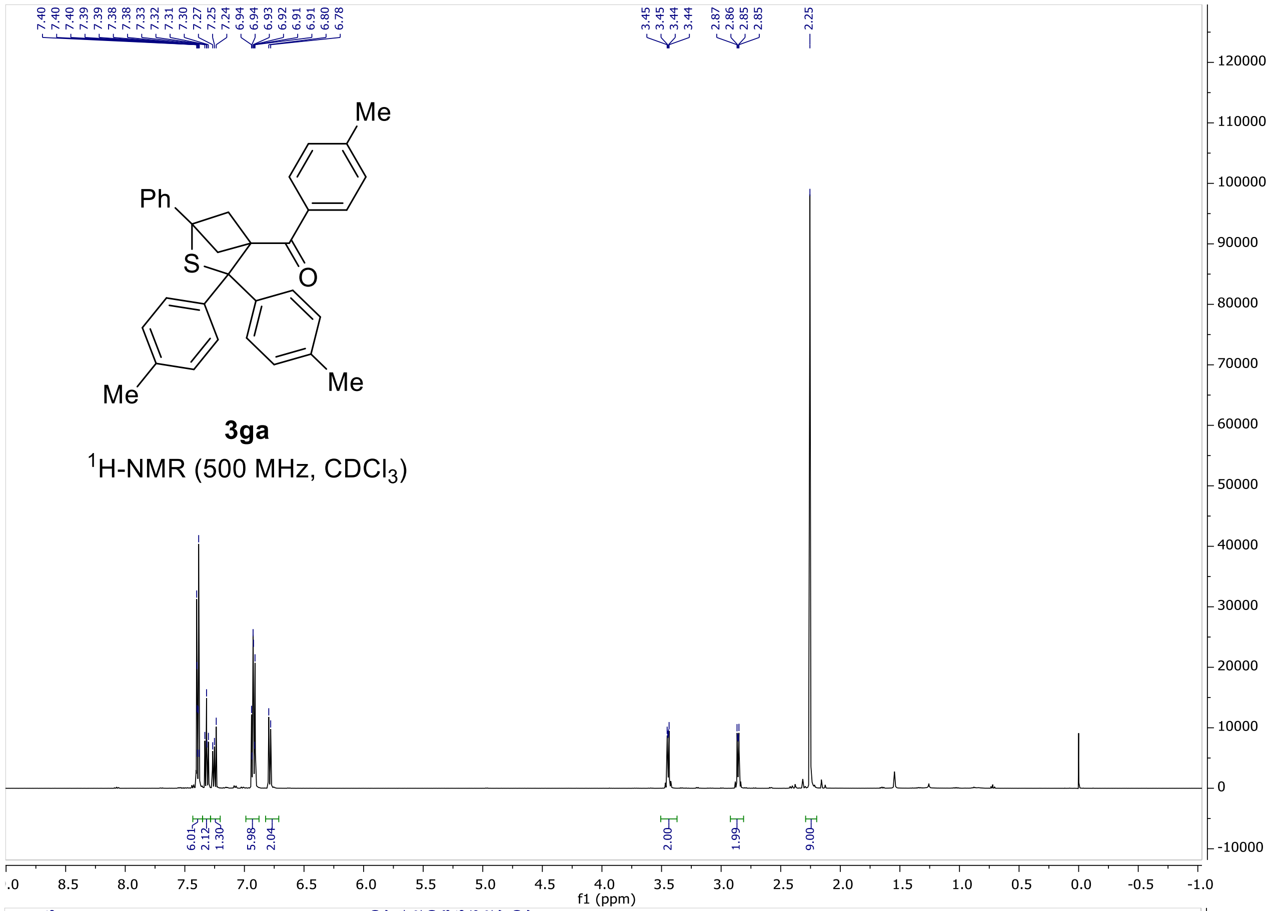

Ph
S
O
Me
Me
Me
3ga
^{1}H-NMR (500 MHz, CDCl$_3$)
f1 (ppm)

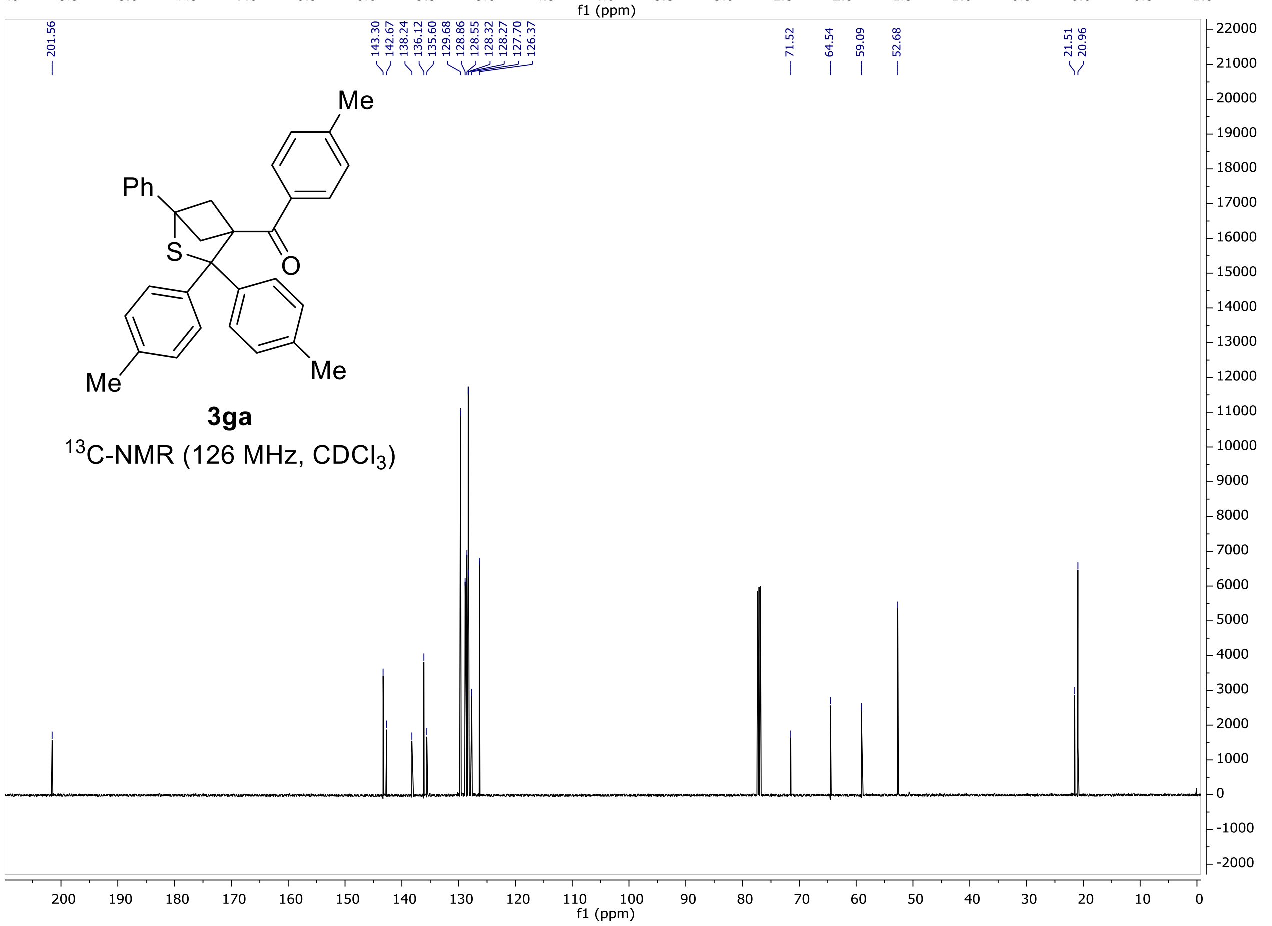

201.56
143.30
142.67
138.24
136.12
135.60
129.68
128.86
128.55
128.32
128.27
127.70
126.37
71.52
64.54
59.09
52.68
21.51
20.96
Ph
S
O
Me
Me
Me
3ga
^{13}C-NMR (126 MHz, CDCl$_3$)
f1 (ppm)

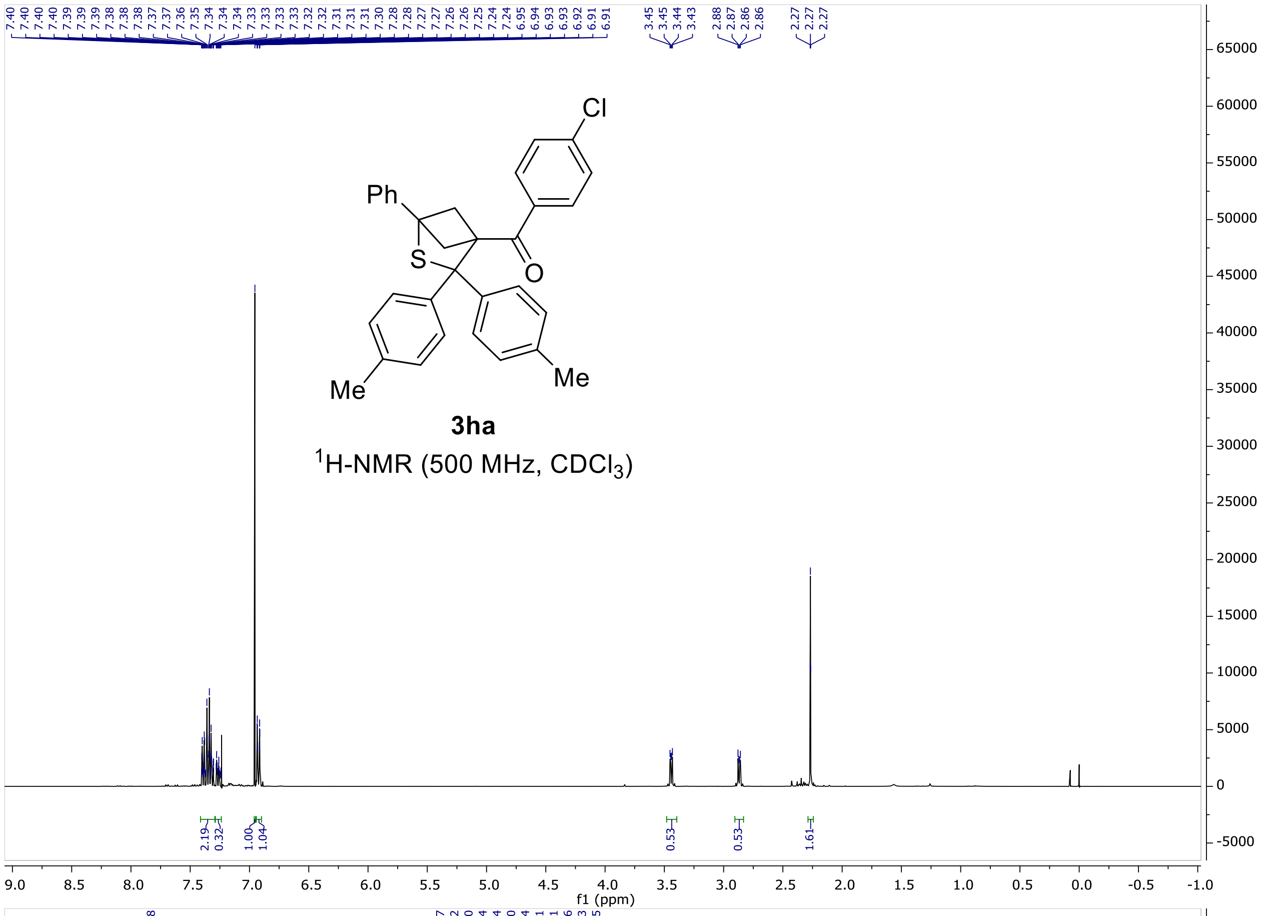

7.40
7.40
7.40
7.40
7.39
7.39
7.39
7.38
7.38
7.38
7.37
7.36
7.35
7.34
7.34
7.34
7.34
7.33
7.33
7.33
7.33
7.32
7.32
7.31
7.31
7.31
7.30
7.28
7.28
7.27
7.27
7.26
7.26
7.25
7.24
7.24
6.95
6.94
6.93
6.93
6.92
6.91
6.91
3.45
3.45
3.44
3.43
2.88
2.87
2.86
2.86
2.27
2.27
2.27
Cl
Ph
S
O
Me
Me
3ha
1H-NMR (500 MHz, CDCl3)
2.19
0.32
1.00
1.04
0.53
0.53
1.61
f1 (ppm)

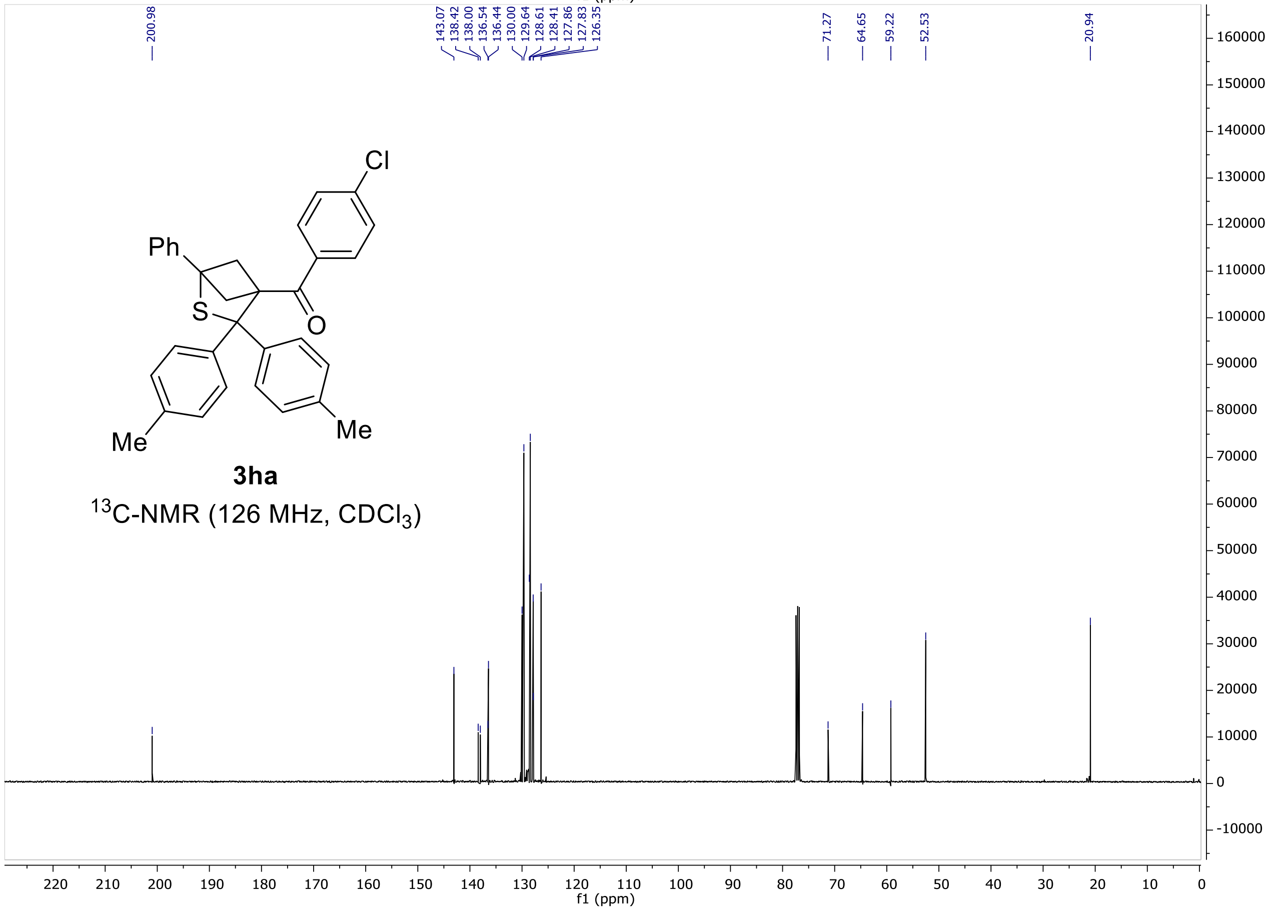

200.98
143.07
138.42
138.00
136.54
136.44
130.00
129.64
128.61
128.41
127.86
127.83
126.35
71.27
64.65
59.22
52.53
20.94
Cl
Ph
S
O
Me
Me
3ha
13C-NMR (126 MHz, CDCl3)
f1 (ppm)

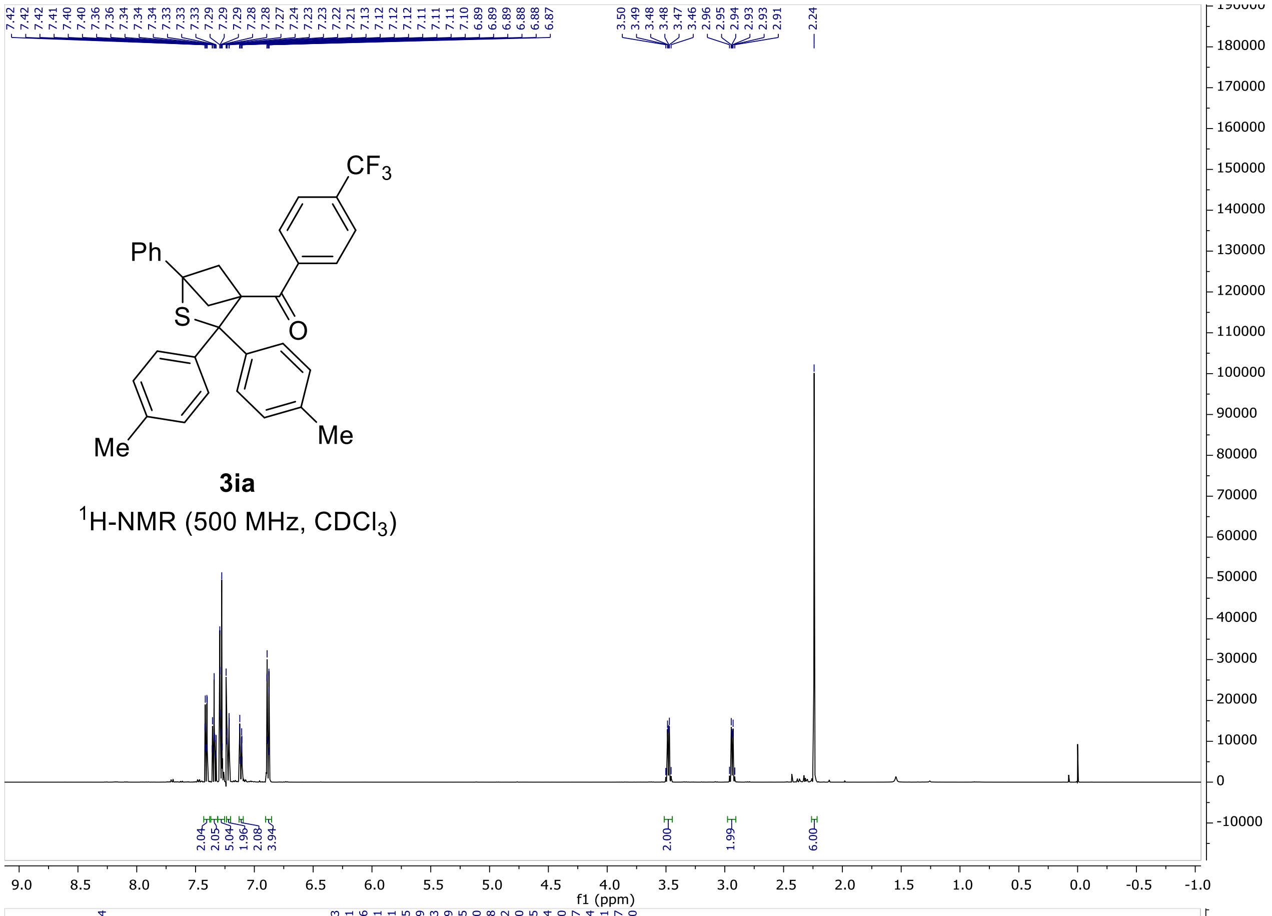

3ia

^{1}H-NMR (500 MHz, $CDCl_3$)

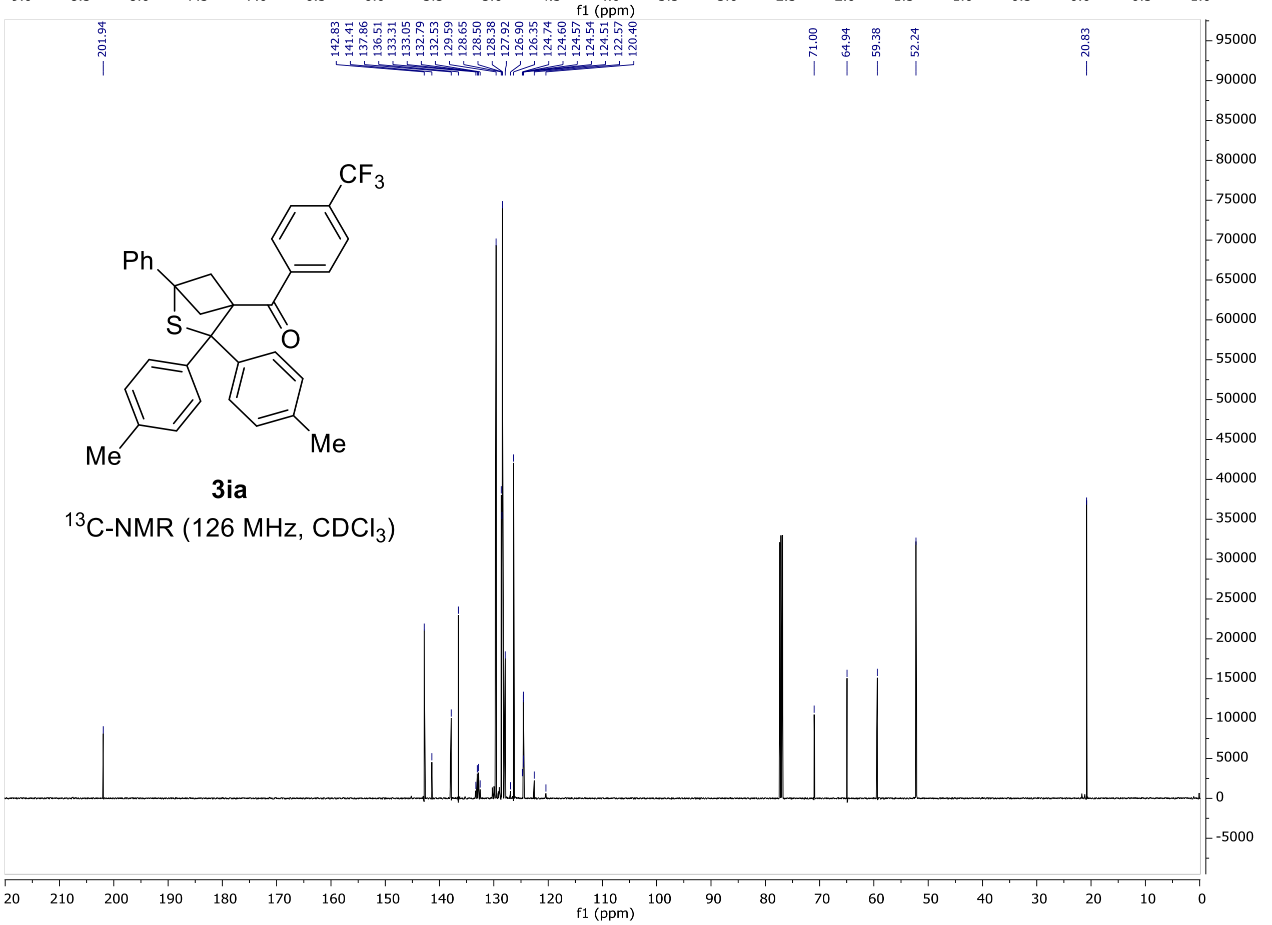

3ia

^{13}C-NMR (126 MHz, $CDCl_3$)

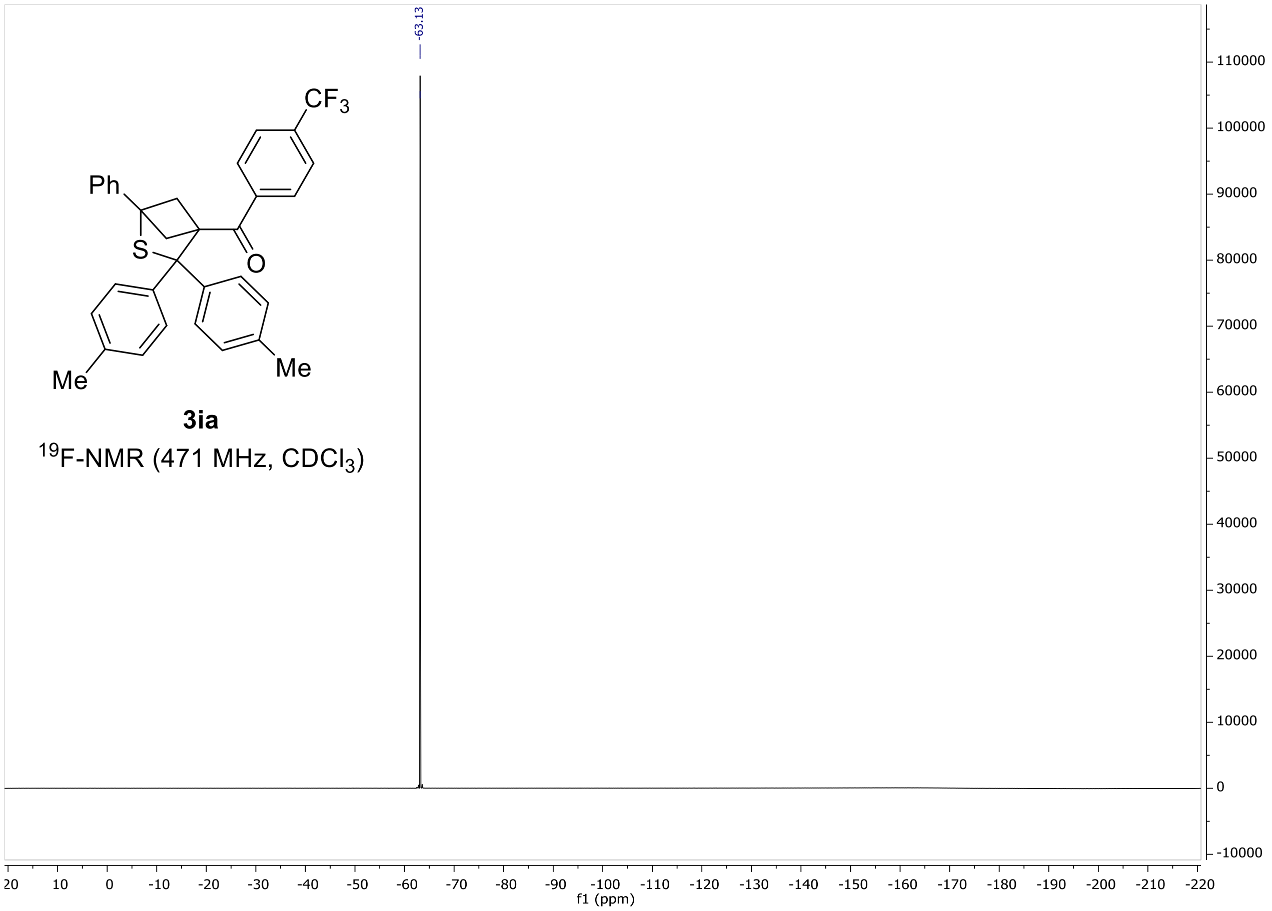
-63.13
CF3
Ph
S
O
Me
Me
3ia
19F-NMR (471 MHz, CDCl3)
110000
100000
90000
80000
70000
60000
50000
40000
30000
20000
10000
0
-10000
20
10
0
-10
-20
-30
-40
-50
-60
-70
-80
-90
-100
-110
-120
-130
-140
-150
-160
-170
-180
-190
-200
-210
-220
f1 (ppm)

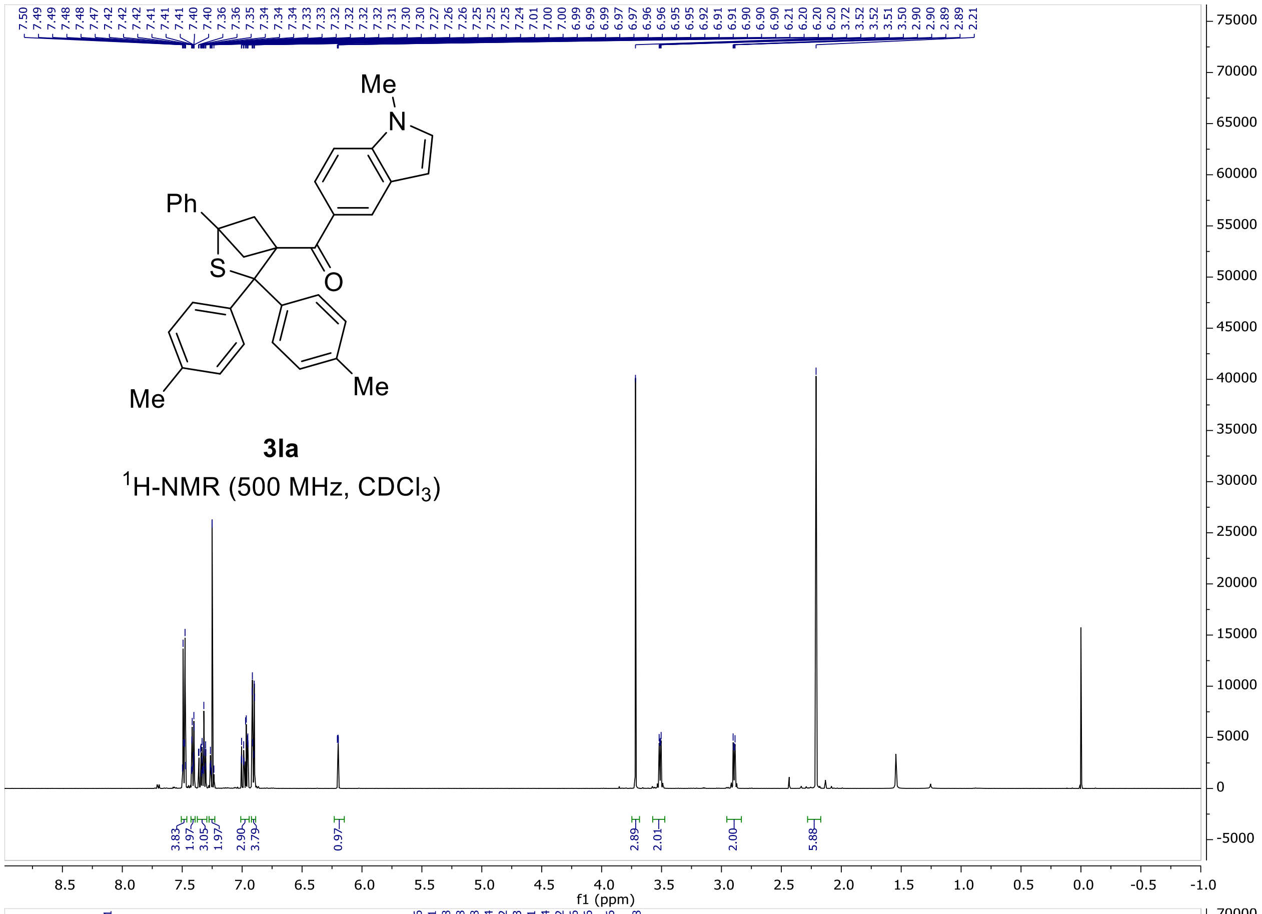
Me
N
Ph
S
O
Me
Me
3la
^{1}H-NMR (500 MHz, $CDCl_3$)
3.83
1.97
3.05
1.97
2.90
3.79
0.97
2.89
2.01
2.00
5.88
f1 (ppm)

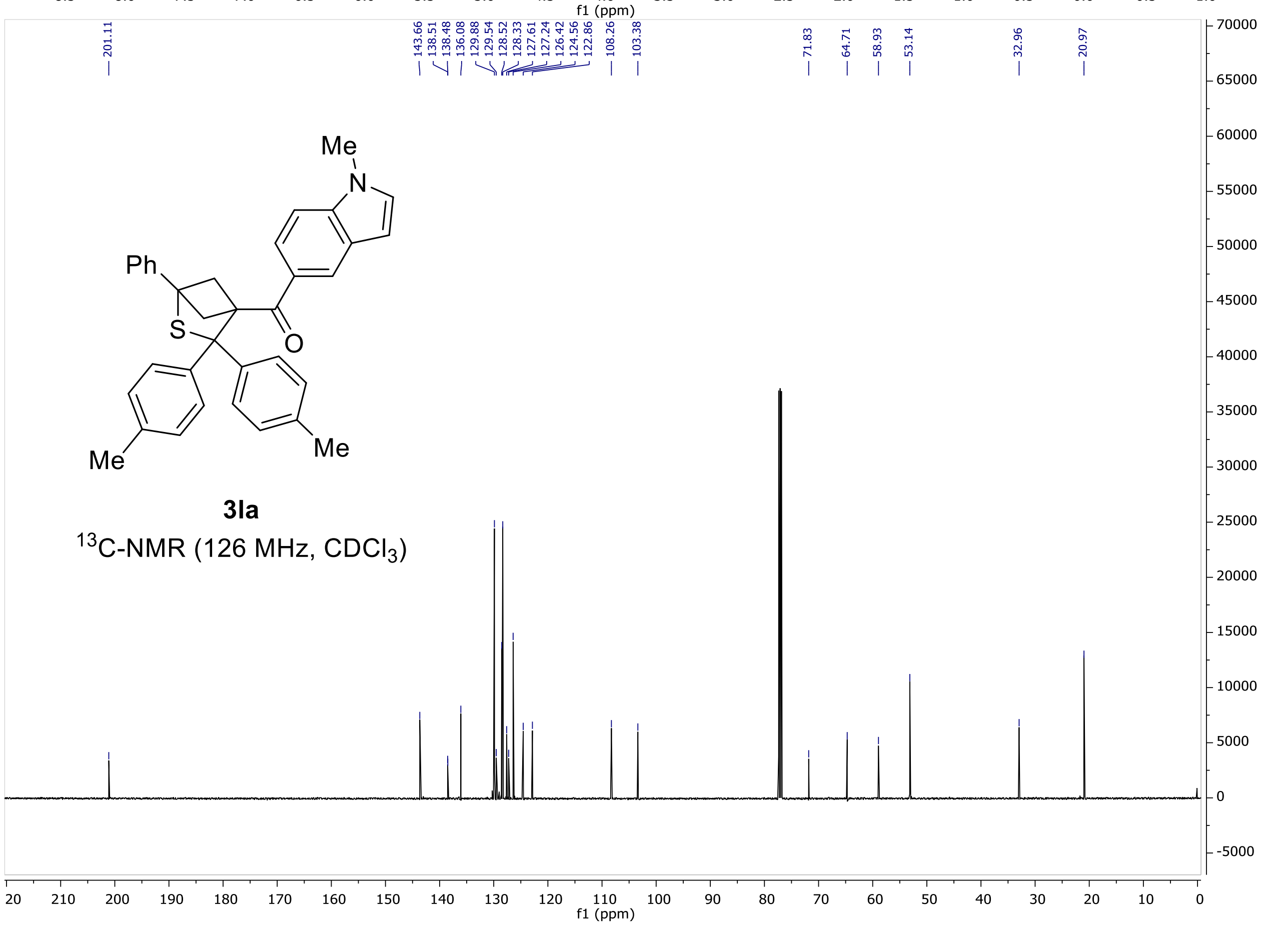
201.11
143.66
138.51
138.48
136.08
129.88
129.54
128.52
128.33
127.61
127.24
126.42
124.56
122.86
108.26
103.38
71.83
64.71
58.93
53.14
32.96
20.97
Me
N
Ph
S
O
Me
Me
3la
^{13}C-NMR (126 MHz, $CDCl_3$)
f1 (ppm)

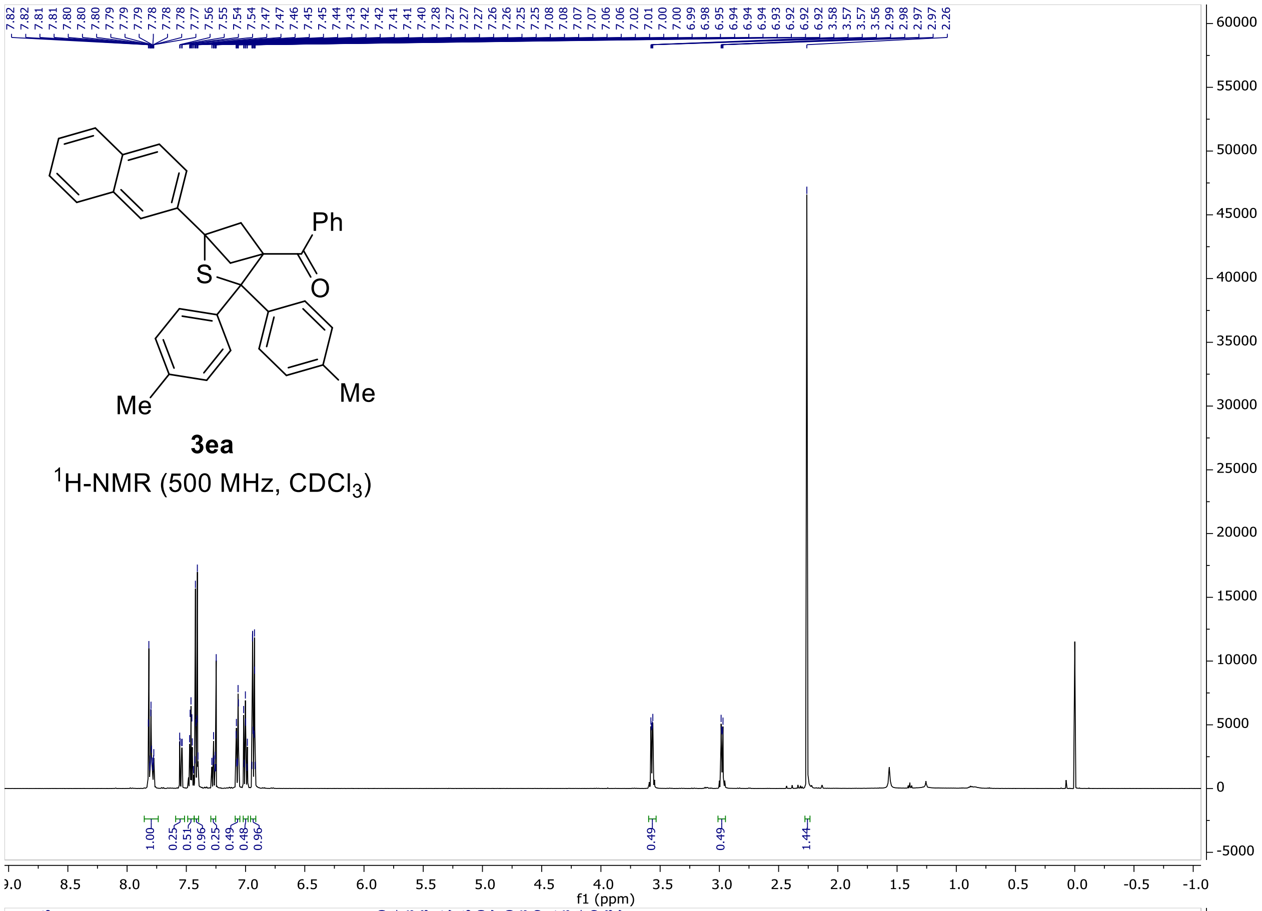

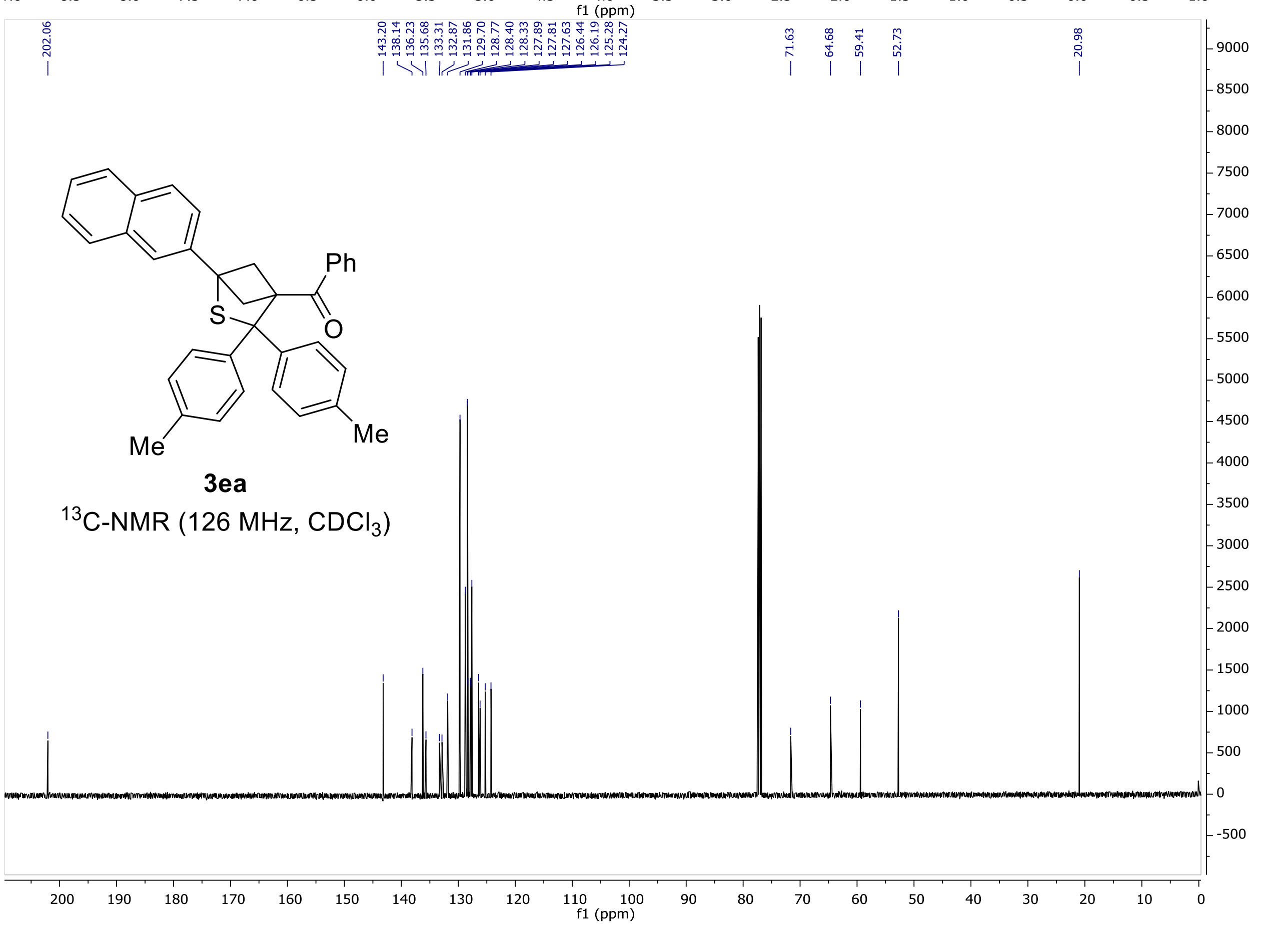

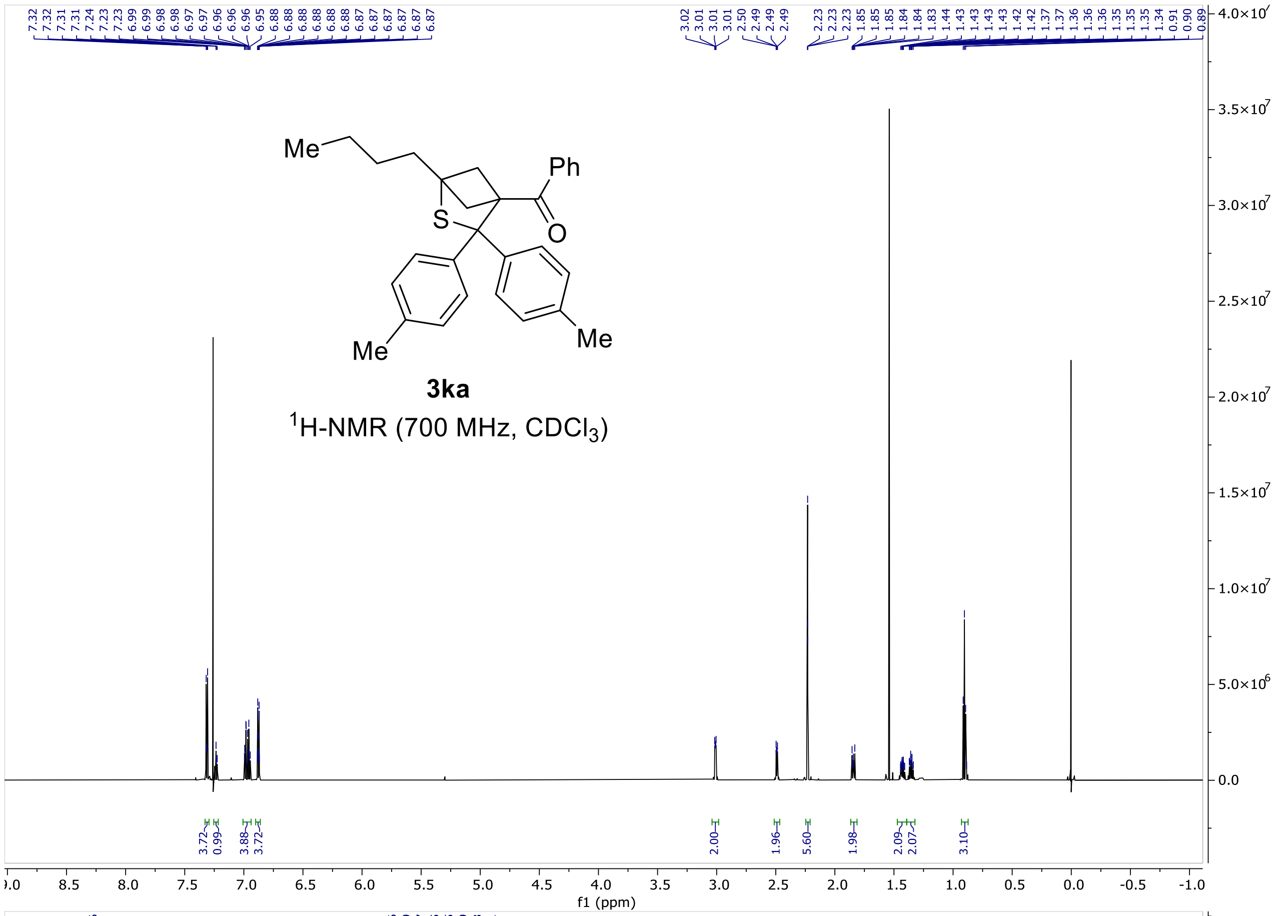
Me
Ph
S
O
Me
Me
3ka
1H-NMR (700 MHz, CDCl3)
f1 (ppm)

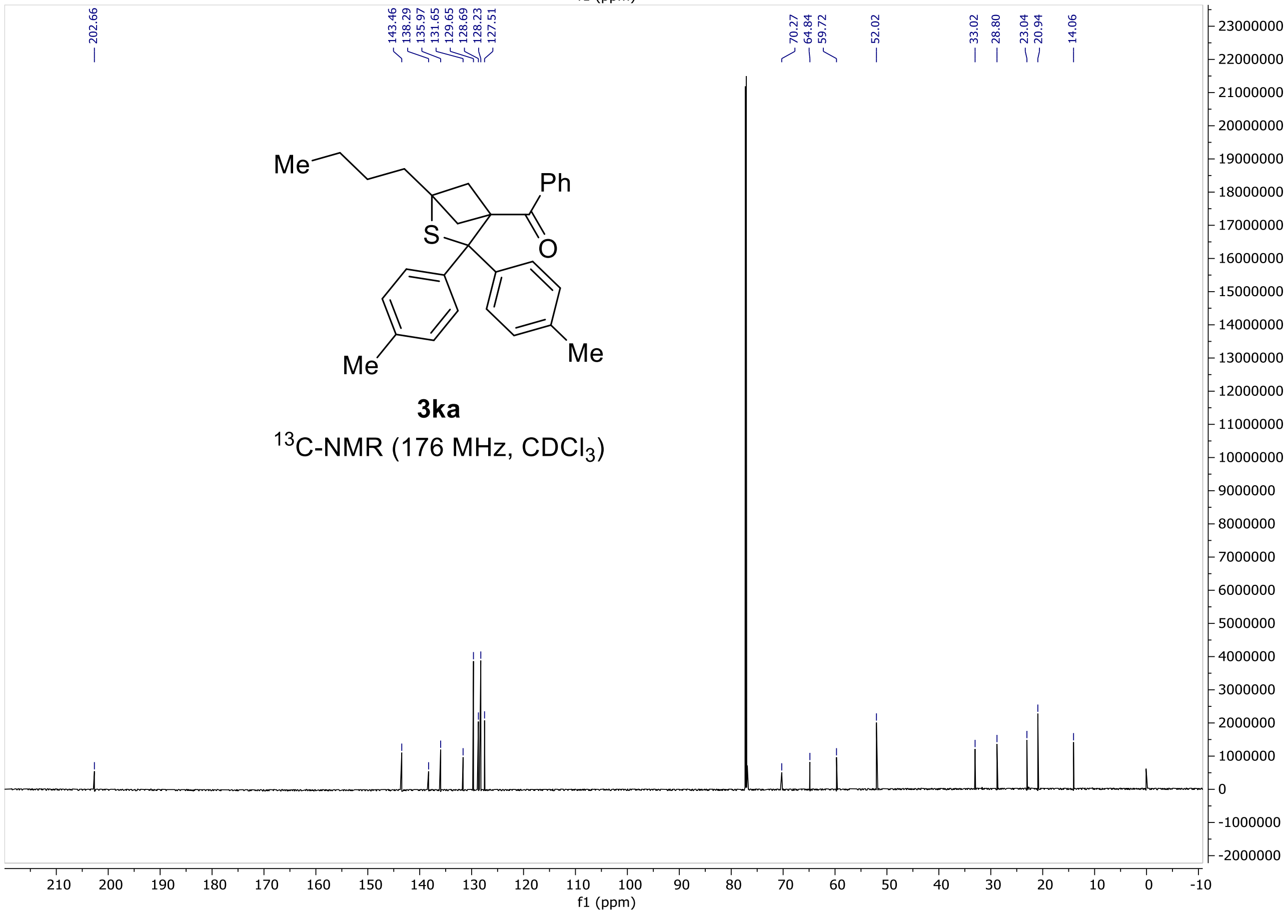
Me
Ph
S
O
Me
Me
3ka
13C-NMR (176 MHz, CDCl3)
f1 (ppm)

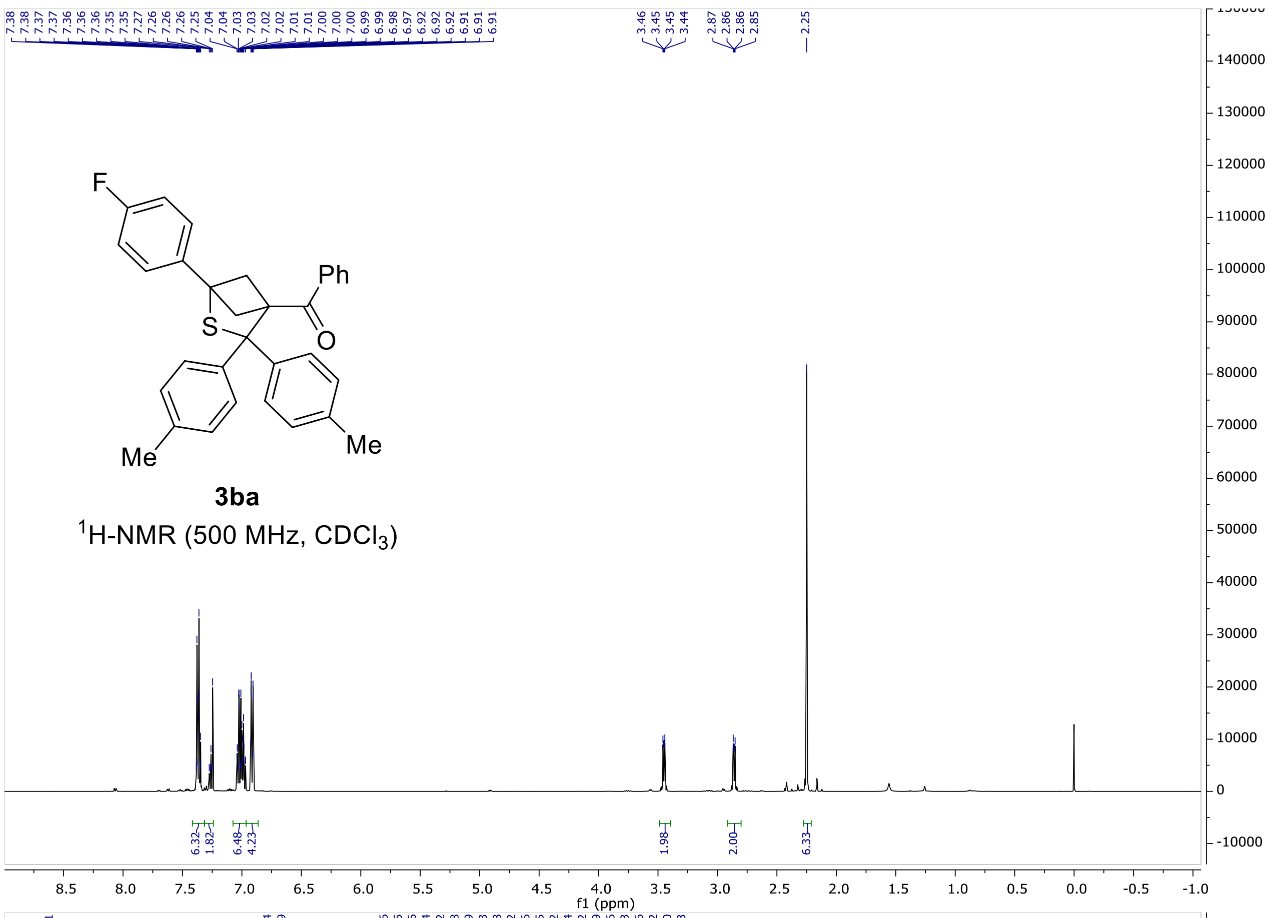
7.38 7.38 7.37 7.37 7.36 7.36 7.36 7.35 7.35 7.27 7.26 7.26 7.26 7.25 7.04 7.04 7.03 7.03 7.02 7.02 7.01 7.01 7.00 7.00 7.00 6.99 6.99 6.98 6.97 6.92 6.92 6.92 6.92 6.91 6.91 6.91
3.46 3.45 3.45 3.44 2.87 2.86 2.86 2.85 2.25
F
Ph
S
O
Me
Me
3ba
^{1}H-NMR (500 MHz, CDCl$_3$)
6.32 1.82 6.48 4.23 1.98 2.00 6.33
f1 (ppm)

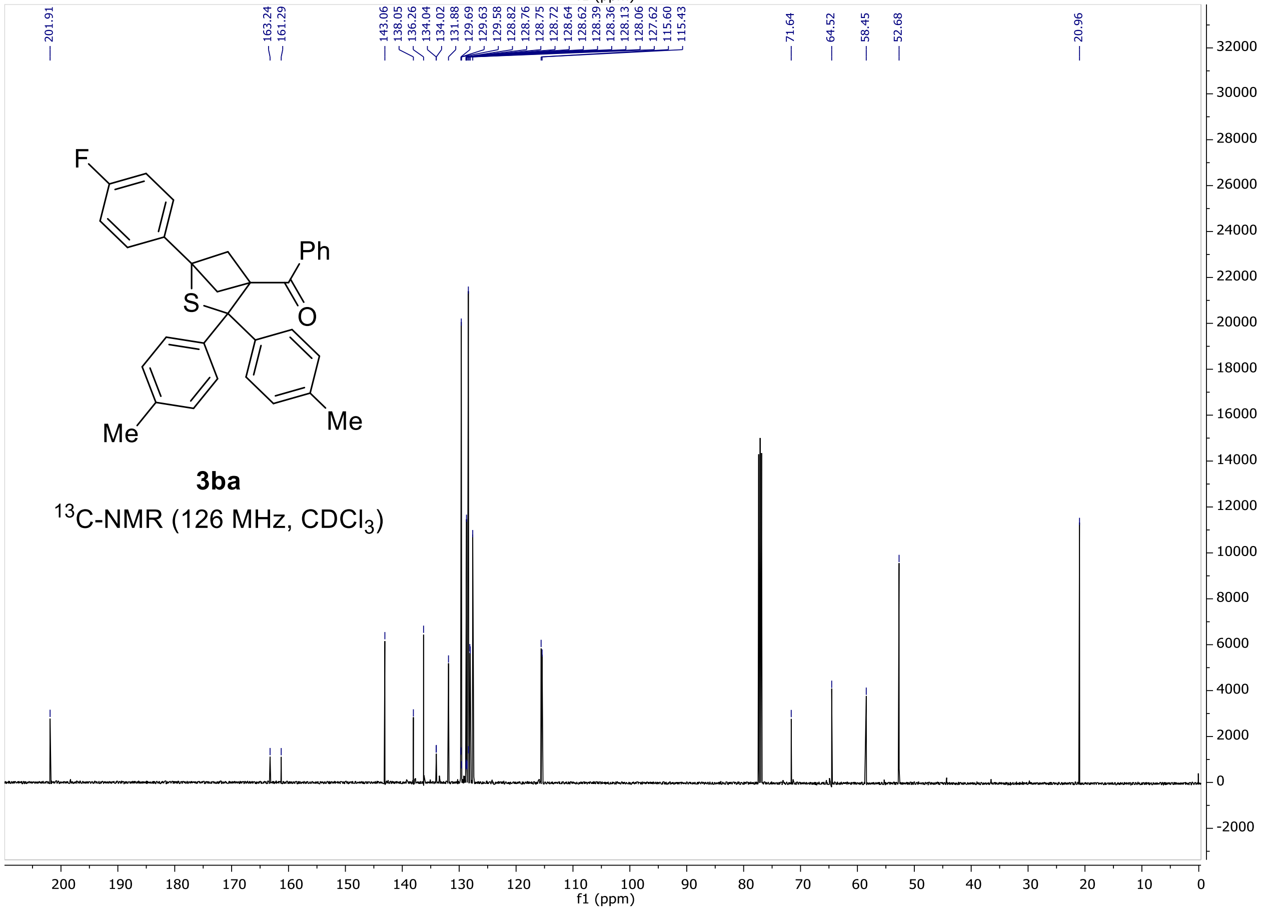
201.91 163.24 161.29 143.06 138.05 136.26 134.04 134.02 131.88 129.69 129.63 129.58 128.82 128.76 128.75 128.72 128.64 128.62 128.39 128.36 128.13 128.06 127.62 115.60 115.43 71.64 64.52 58.45 52.68 20.96
F
Ph
S
O
Me
Me
3ba
^{13}C-NMR (126 MHz, CDCl$_3$)
f1 (ppm)

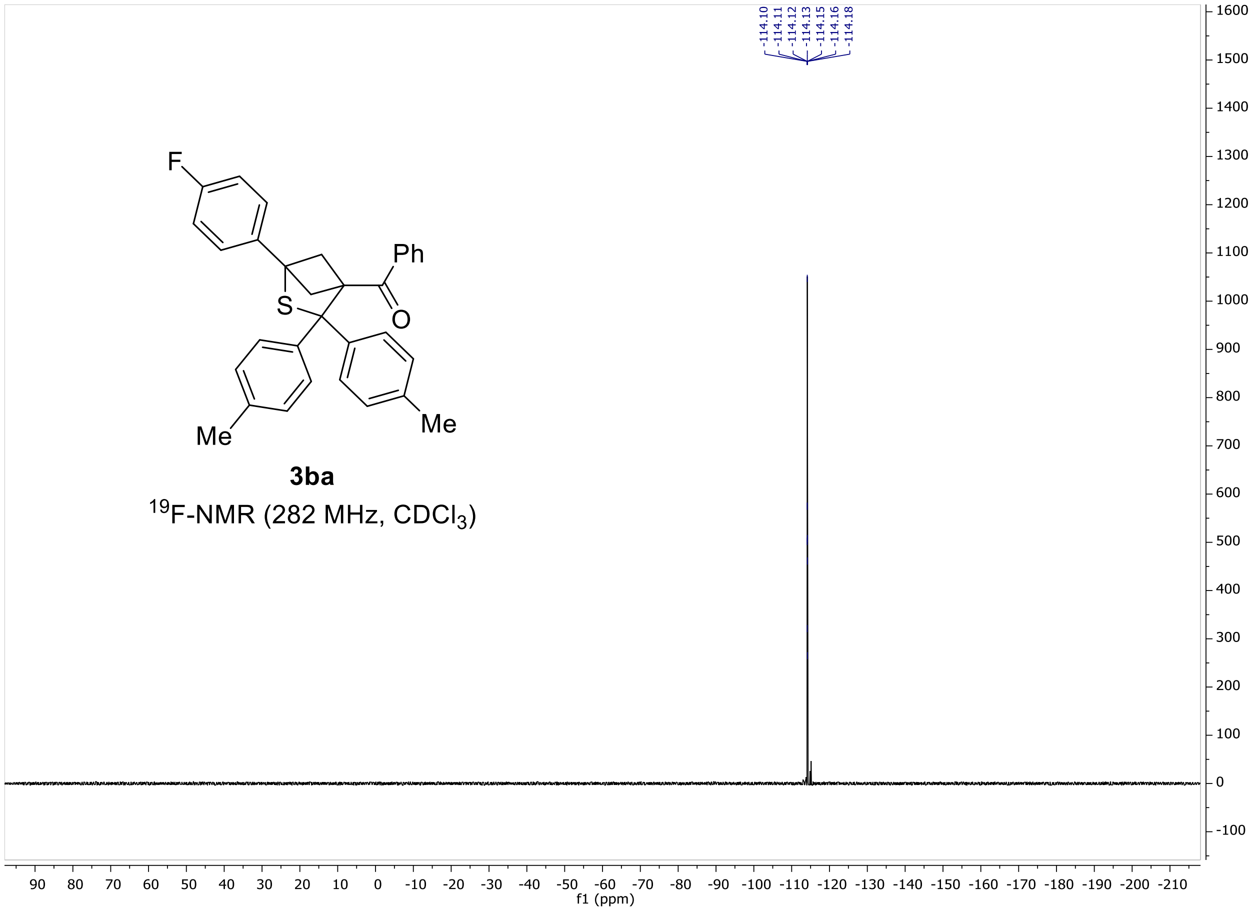

-114.10
-114.11
-114.12
-114.13
-114.15
-114.16
-114.18
F
Ph
S
O
Me
Me
3ba
^{19}F-NMR (282 MHz, CDCl$_3$)
f1 (ppm)

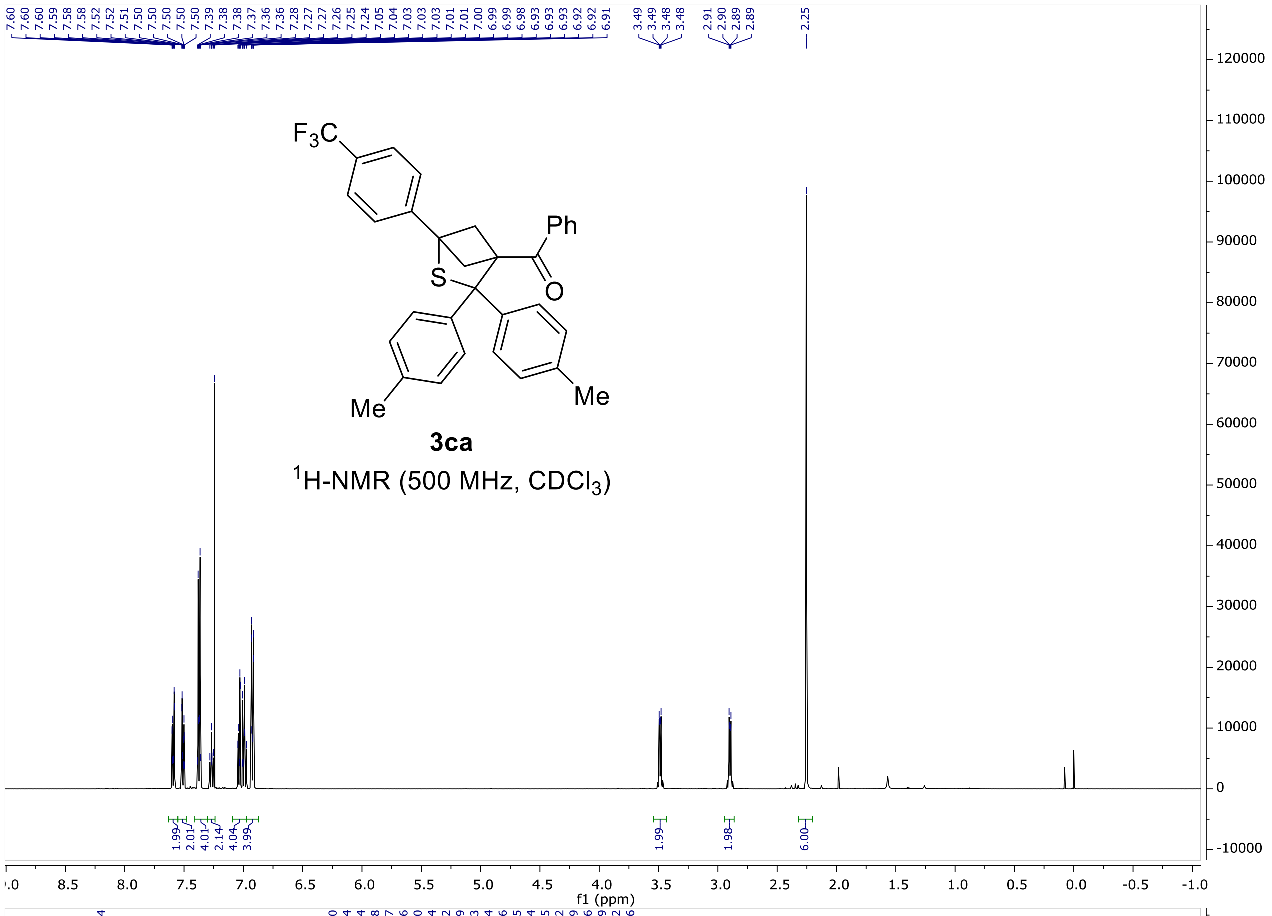

F3C
Ph
S
O
Me
Me
3ca
1H-NMR (500 MHz, CDCl3)
f1 (ppm)

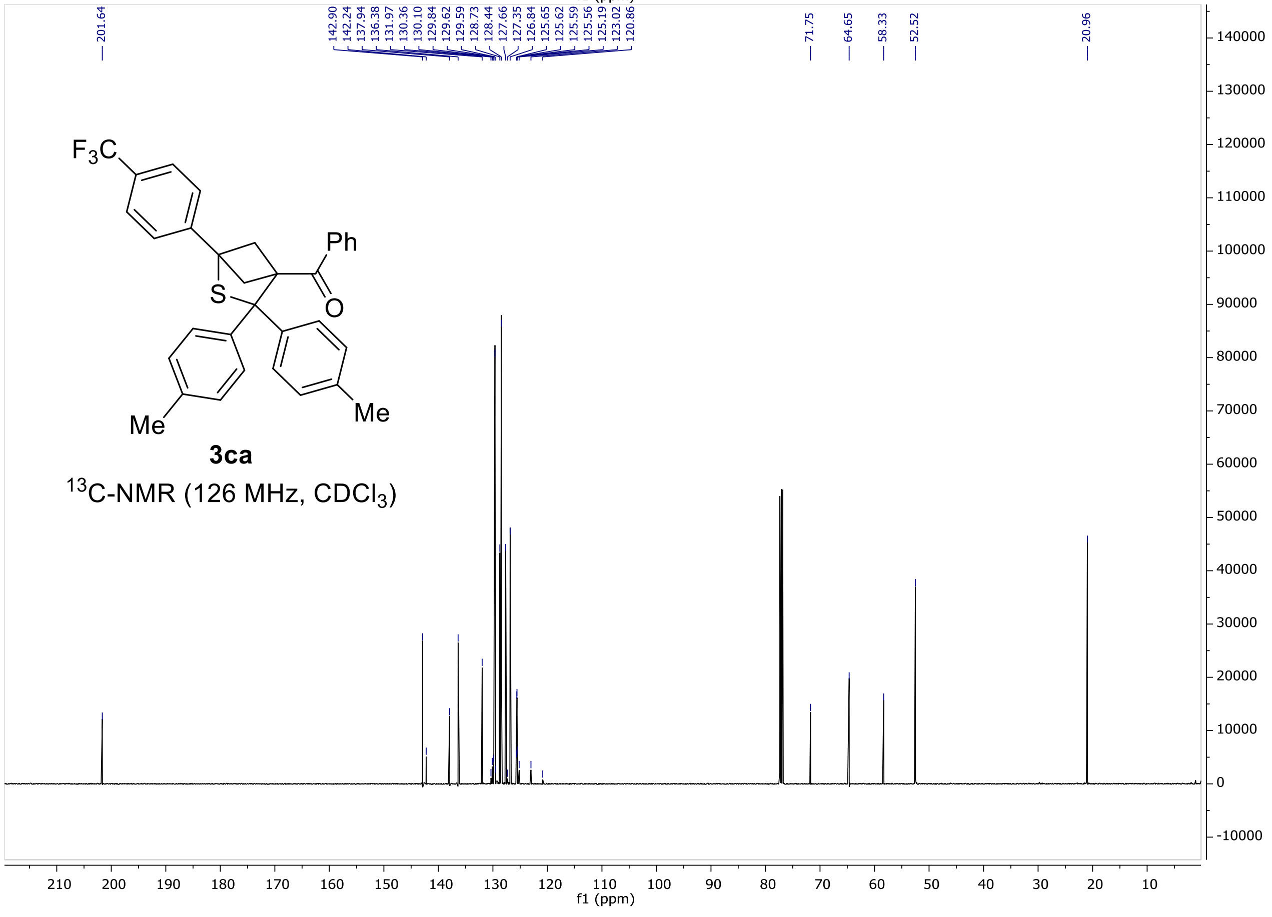

F3C
Ph
S
O
Me
Me
3ca
13C-NMR (126 MHz, CDCl3)
f1 (ppm)

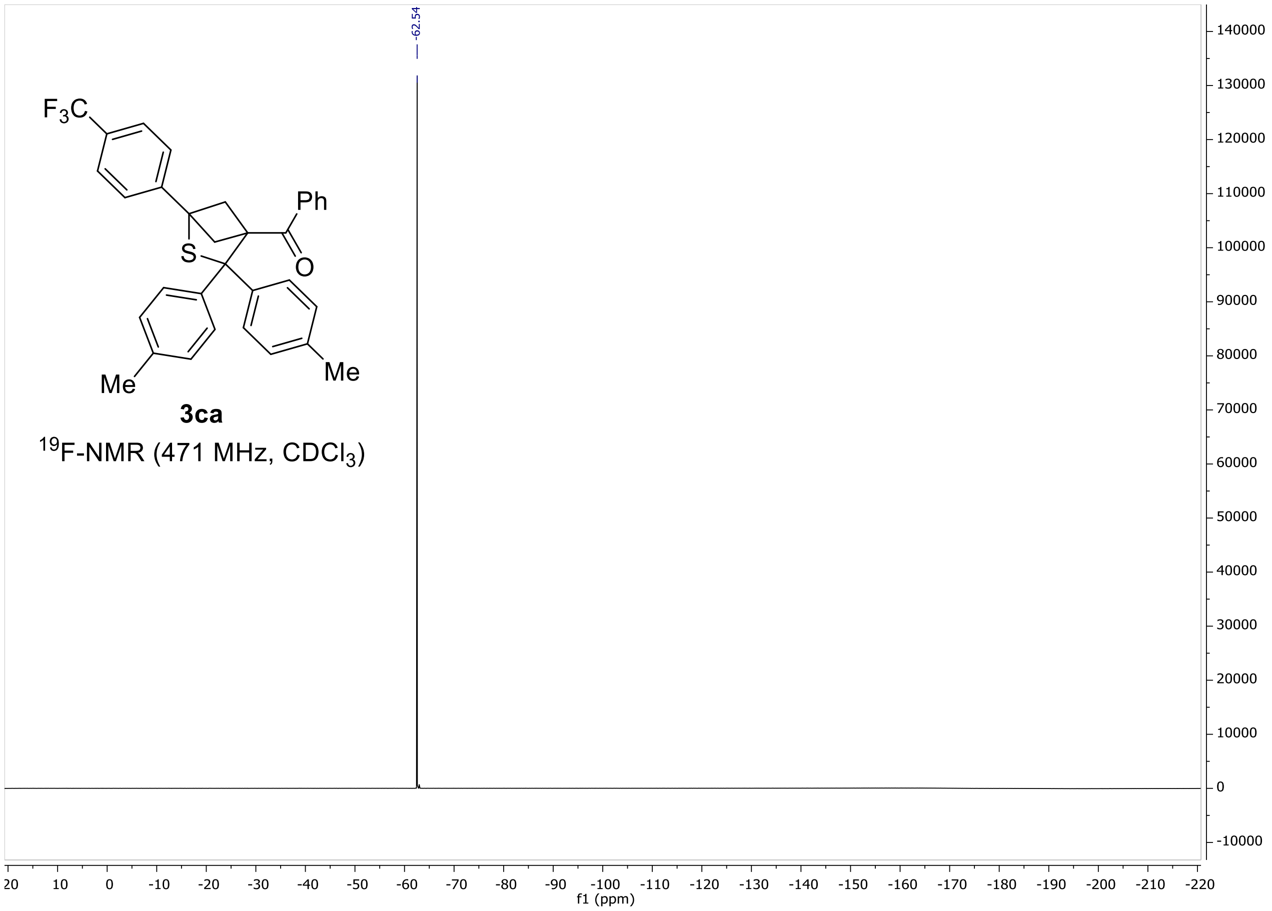

-62.54
F3C
Ph
S
O
Me
Me
3ca
19F-NMR (471 MHz, CDCl3)
f1 (ppm)

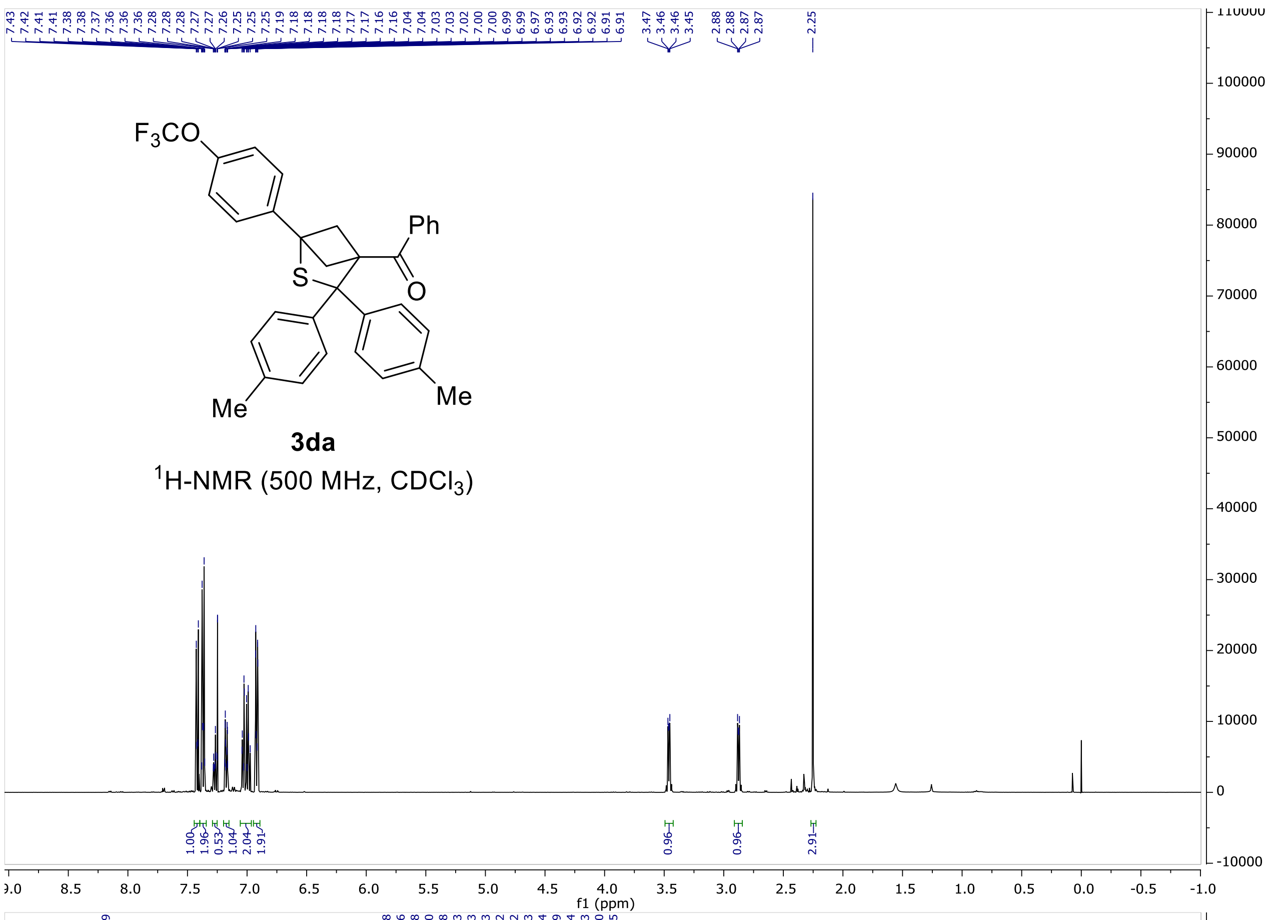
F3CO
Ph
S
O
Me
Me
3da
1H-NMR (500 MHz, CDCl3)
f1 (ppm)

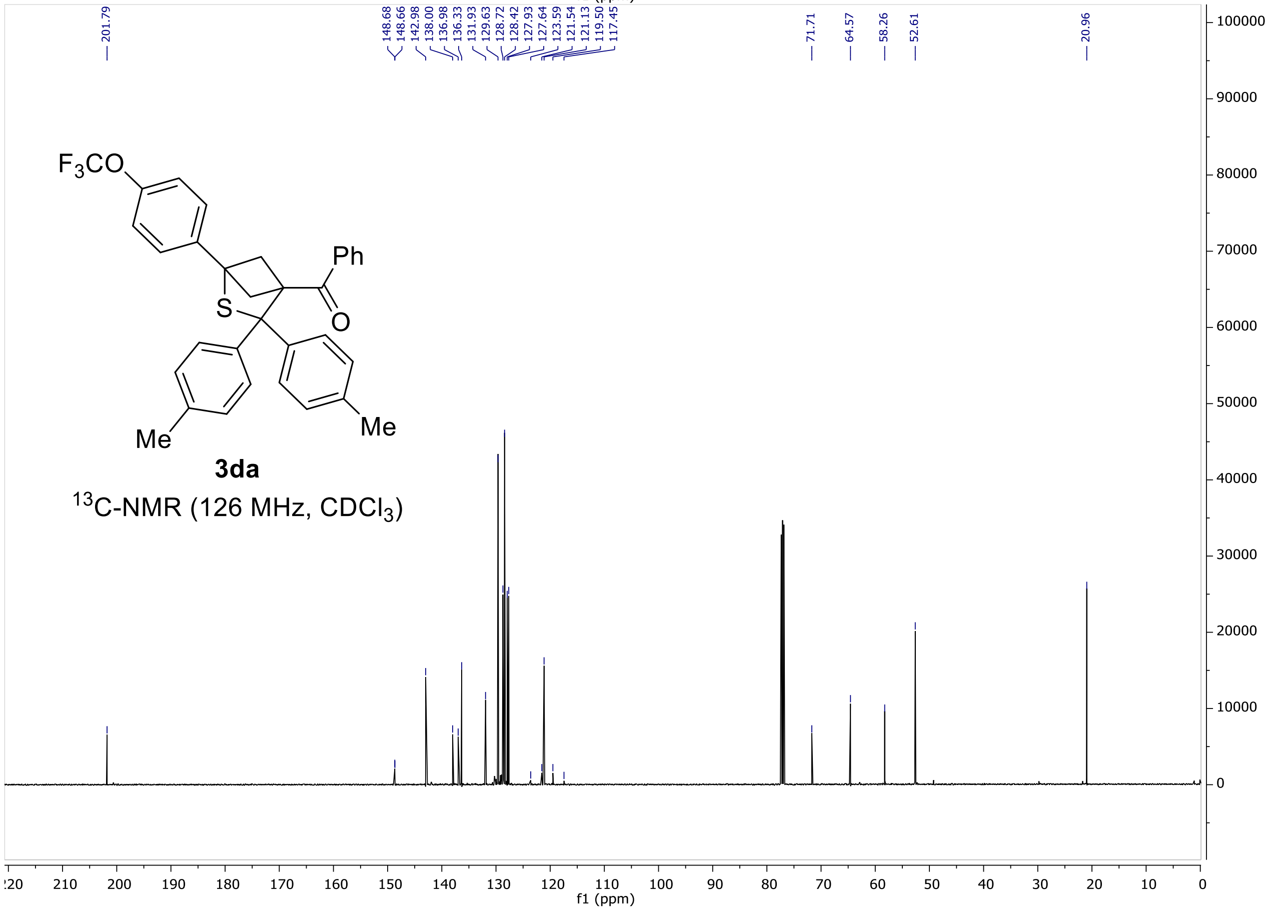
F3CO
Ph
S
O
Me
Me
3da
13C-NMR (126 MHz, CDCl3)
f1 (ppm)

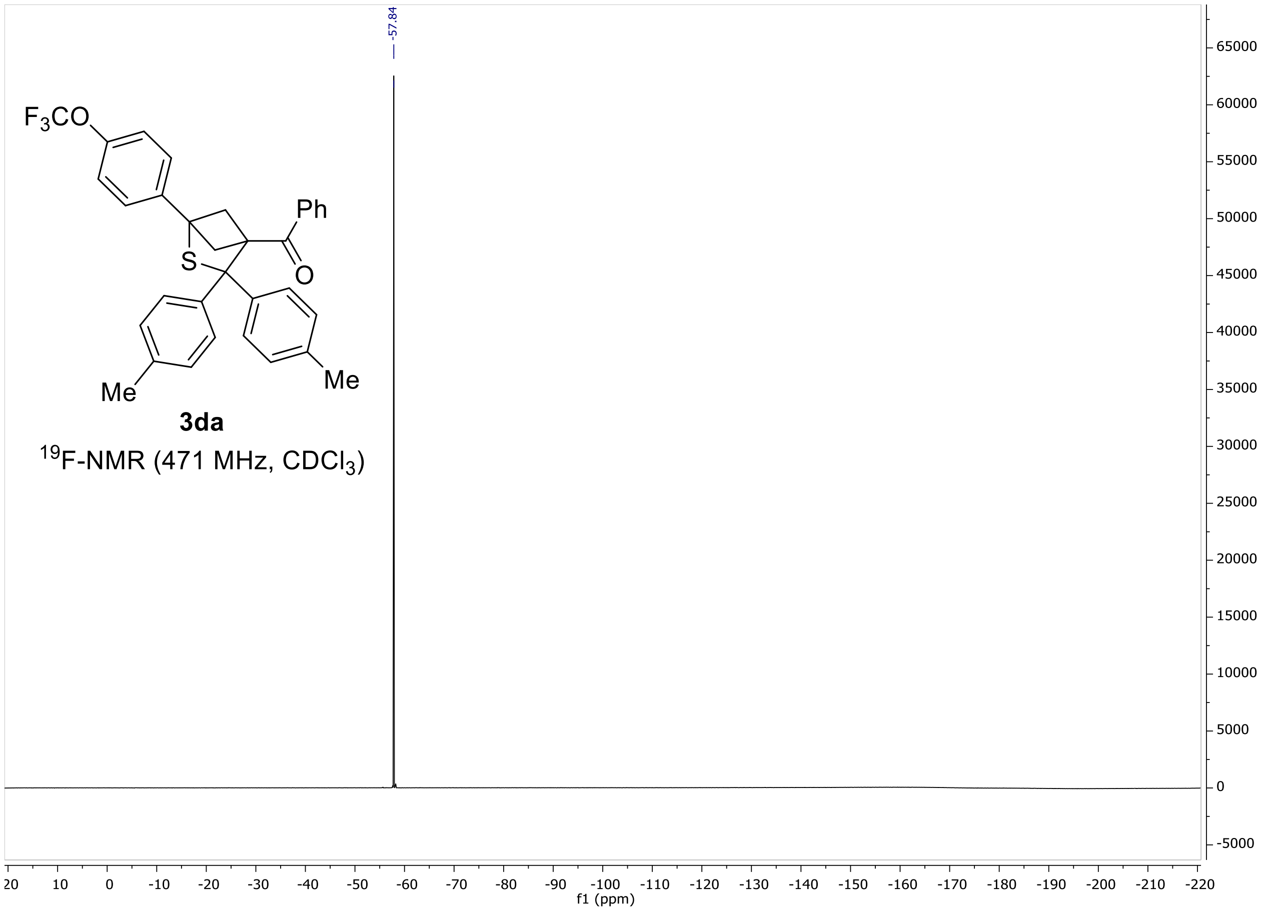
-57.84
F3CO
Ph
S
O
Me
Me
3da
19F-NMR (471 MHz, CDCl3)
65000
60000
55000
50000
45000
40000
35000
30000
25000
20000
15000
10000
5000
0
-5000
20
10
0
-10
-20
-30
-40
-50
-60
-70
-80
-90
-100
-110
-120
-130
-140
-150
-160
-170
-180
-190
-200
-210
-220
f1 (ppm)

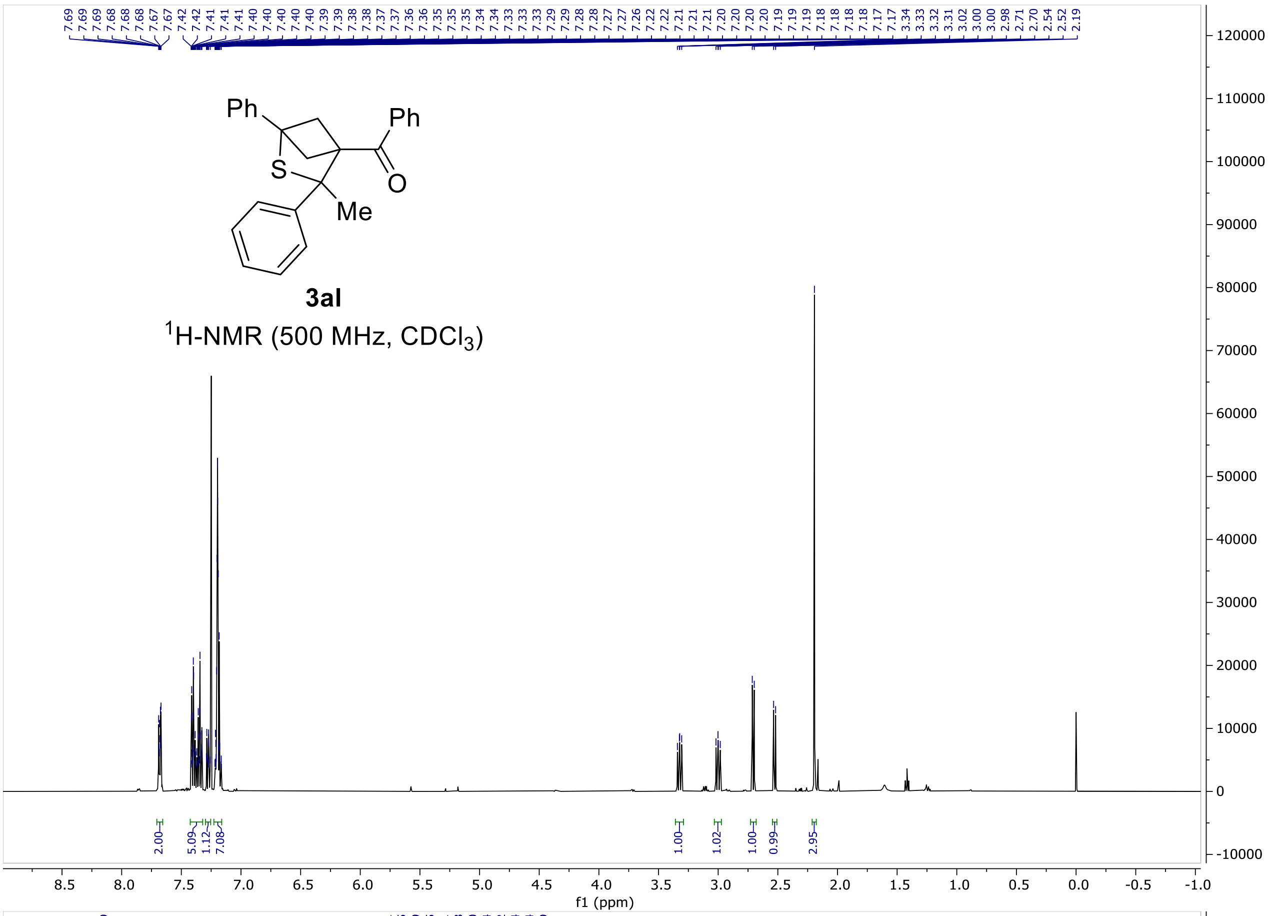
7.69 7.69 7.69 7.68 7.68 7.67 7.67 7.42 7.42 7.41 7.41 7.40 7.40 7.40 7.40 7.39 7.39 7.38 7.38 7.37 7.37 7.36 7.35 7.35 7.35 7.34 7.34 7.33 7.33 7.33 7.29 7.29 7.28 7.27 7.27 7.26 7.22 7.22 7.21 7.21 7.21 7.20 7.20 7.20 7.20 7.19 7.19 7.19 7.18 7.18 7.18 7.18 7.17 7.17 3.34 3.33 3.33 3.32 3.31 3.02 3.00 3.00 2.98 2.71 2.70 2.54 2.52 2.19
Ph
Ph
S
O
Me
3al
1H-NMR (500 MHz, CDCl3)
2.00
5.09
1.12
7.08
1.00
1.02
1.00
0.99
2.95
f1 (ppm)

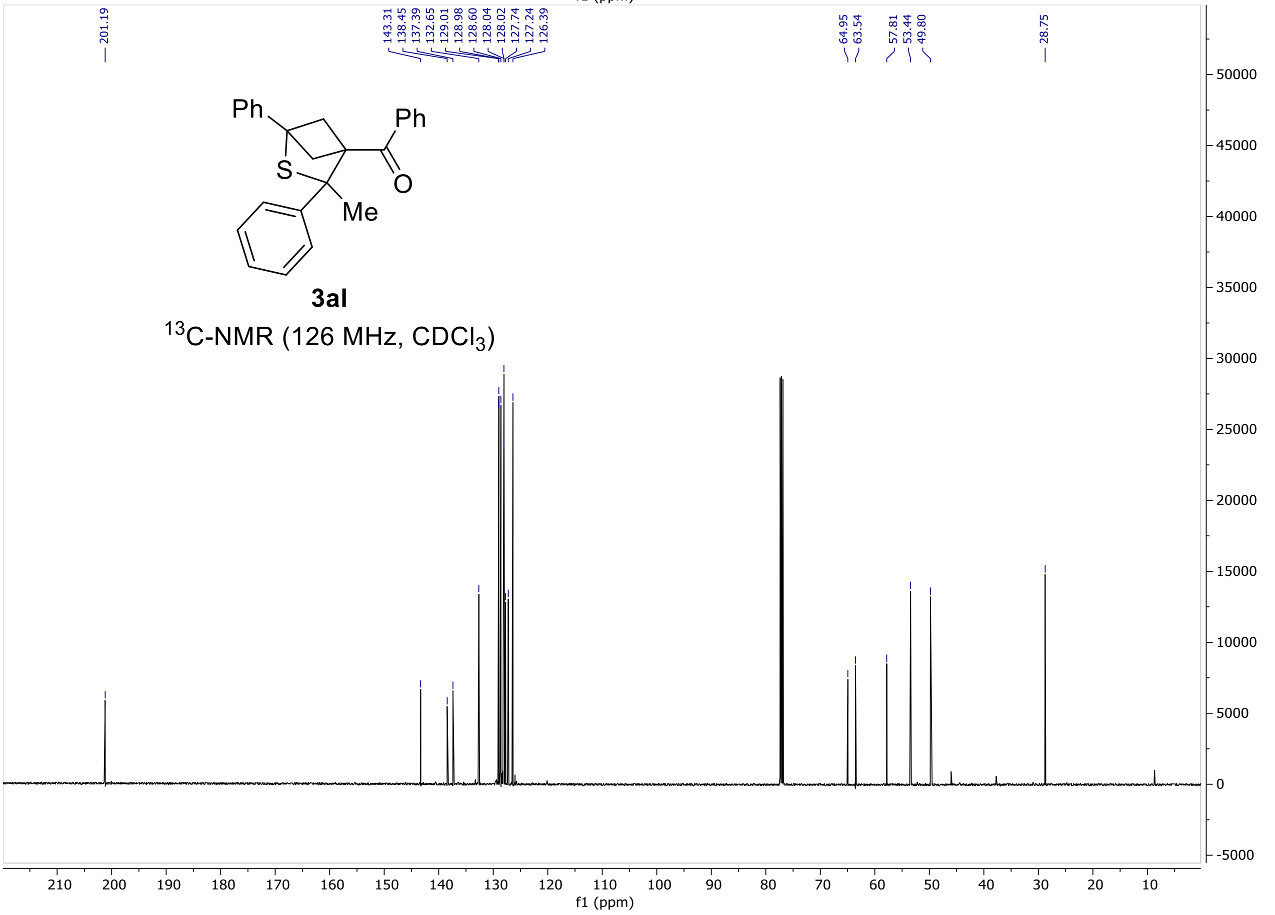
201.19
143.31 138.45 137.39 132.65 129.01 128.98 128.60 128.04 128.02 127.74 127.24 126.39
64.95 63.54
57.81 53.44 49.80
28.75
Ph
Ph
S
O
Me
3al
13C-NMR (126 MHz, CDCl3)
f1 (ppm)

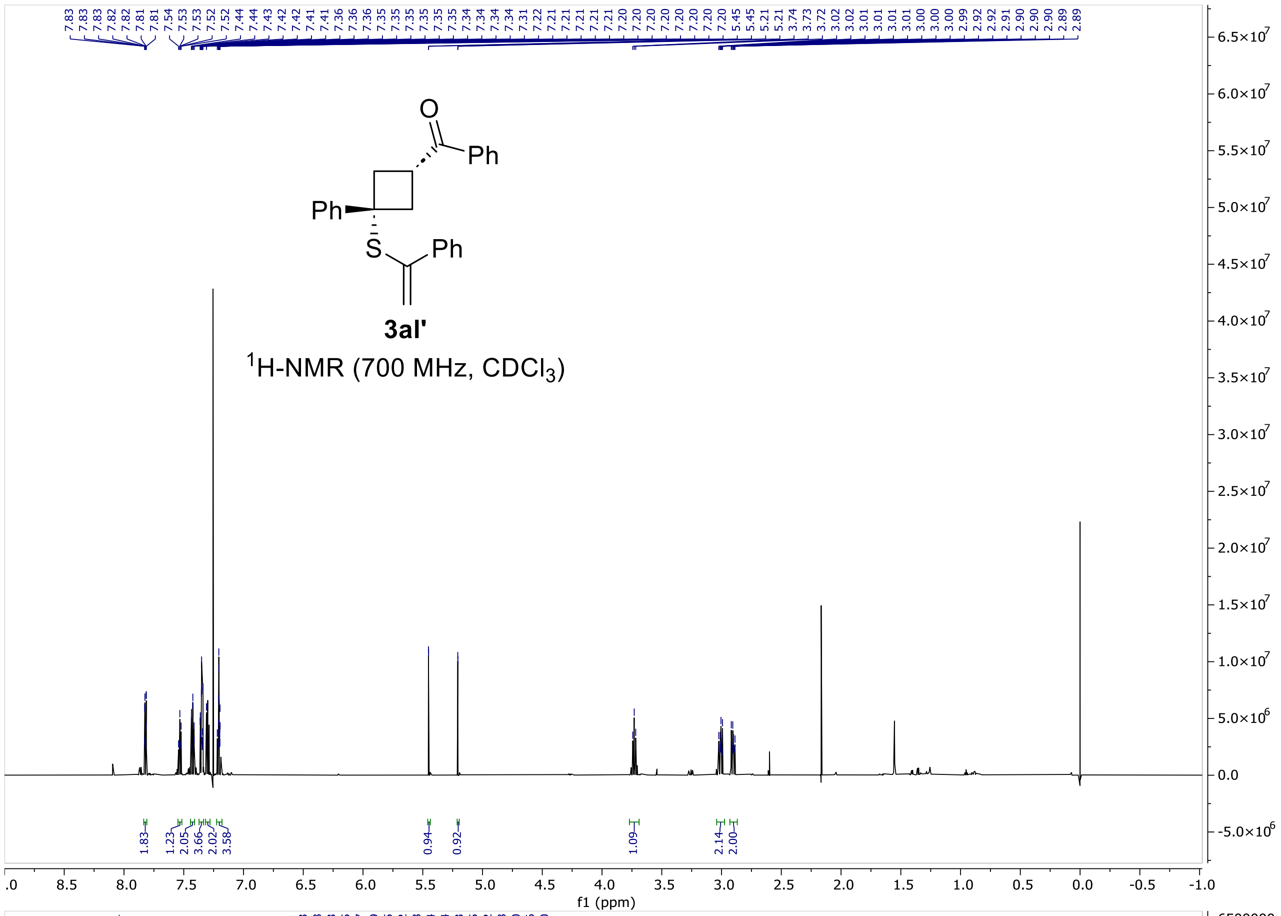
3al'
1H-NMR (700 MHz, CDCl3)
f1 (ppm)

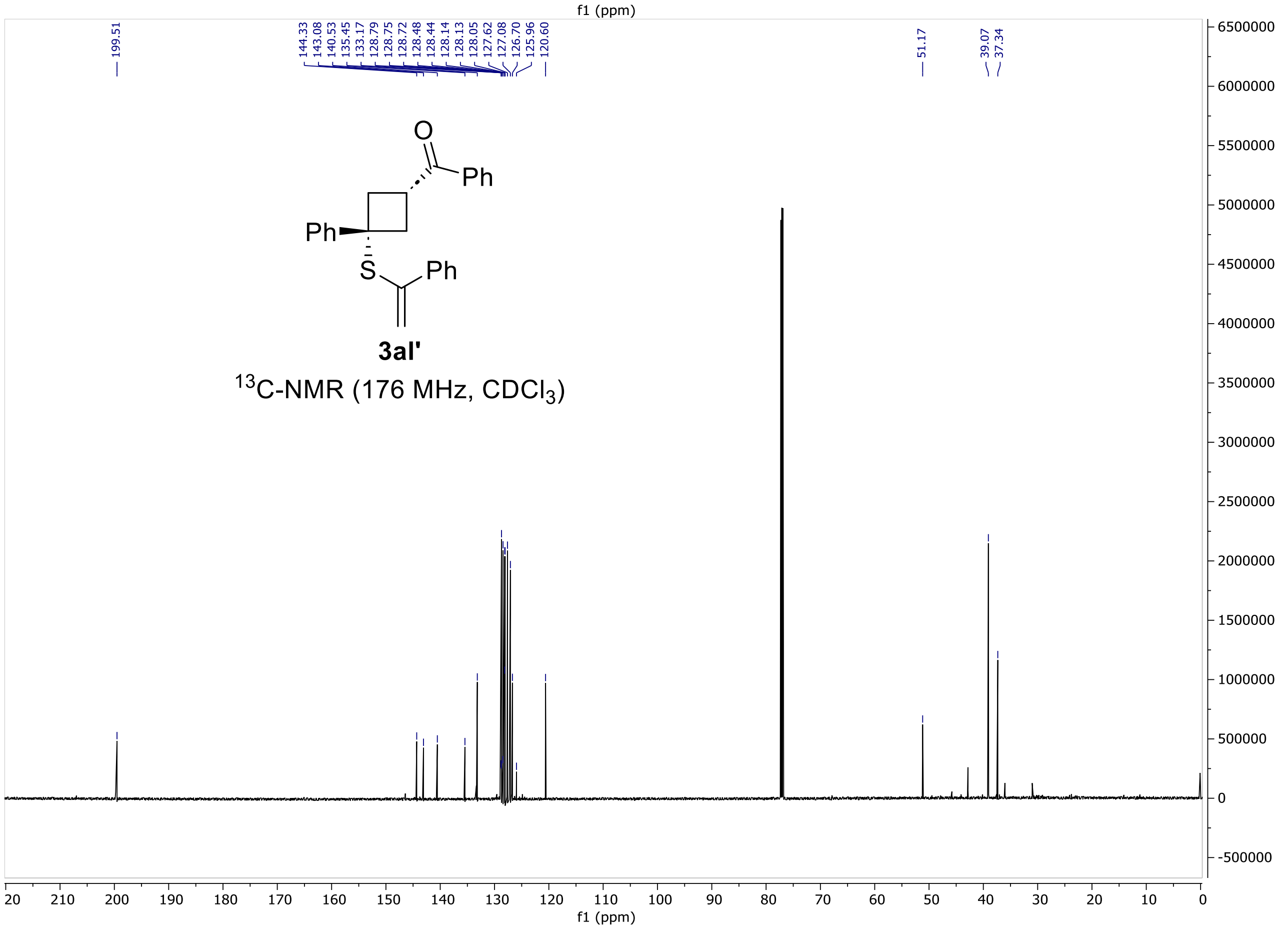
3al'
13C-NMR (176 MHz, CDCl3)
f1 (ppm)
199.51
51.17
39.07
37.34

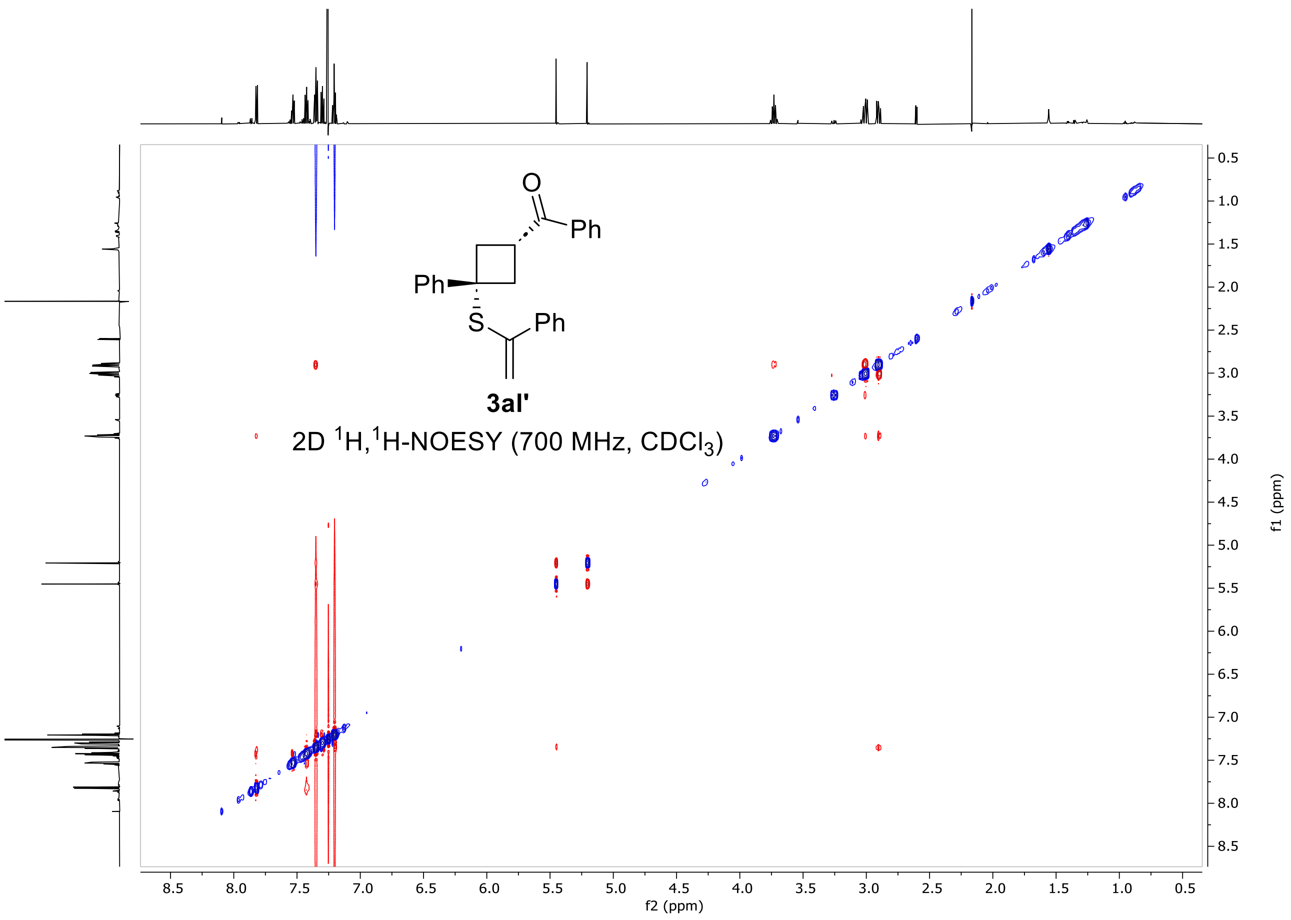

O
Ph
Ph
S
Ph
3al'
2D 1H,1H-NOESY (700 MHz, CDCl3)
f2 (ppm)
f1 (ppm)

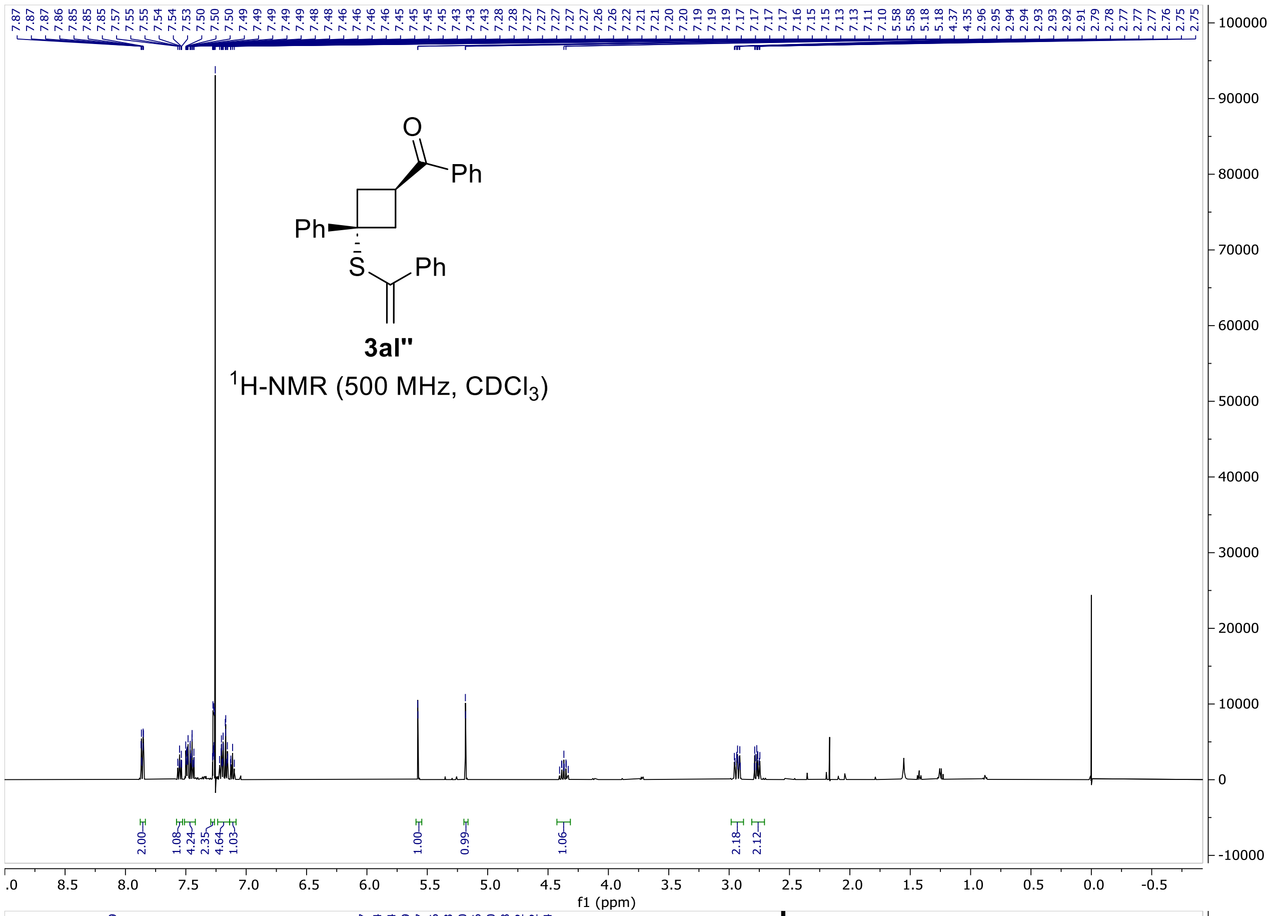
3al''
^{1}H-NMR (500 MHz, CDCl$_3$)
f1 (ppm)

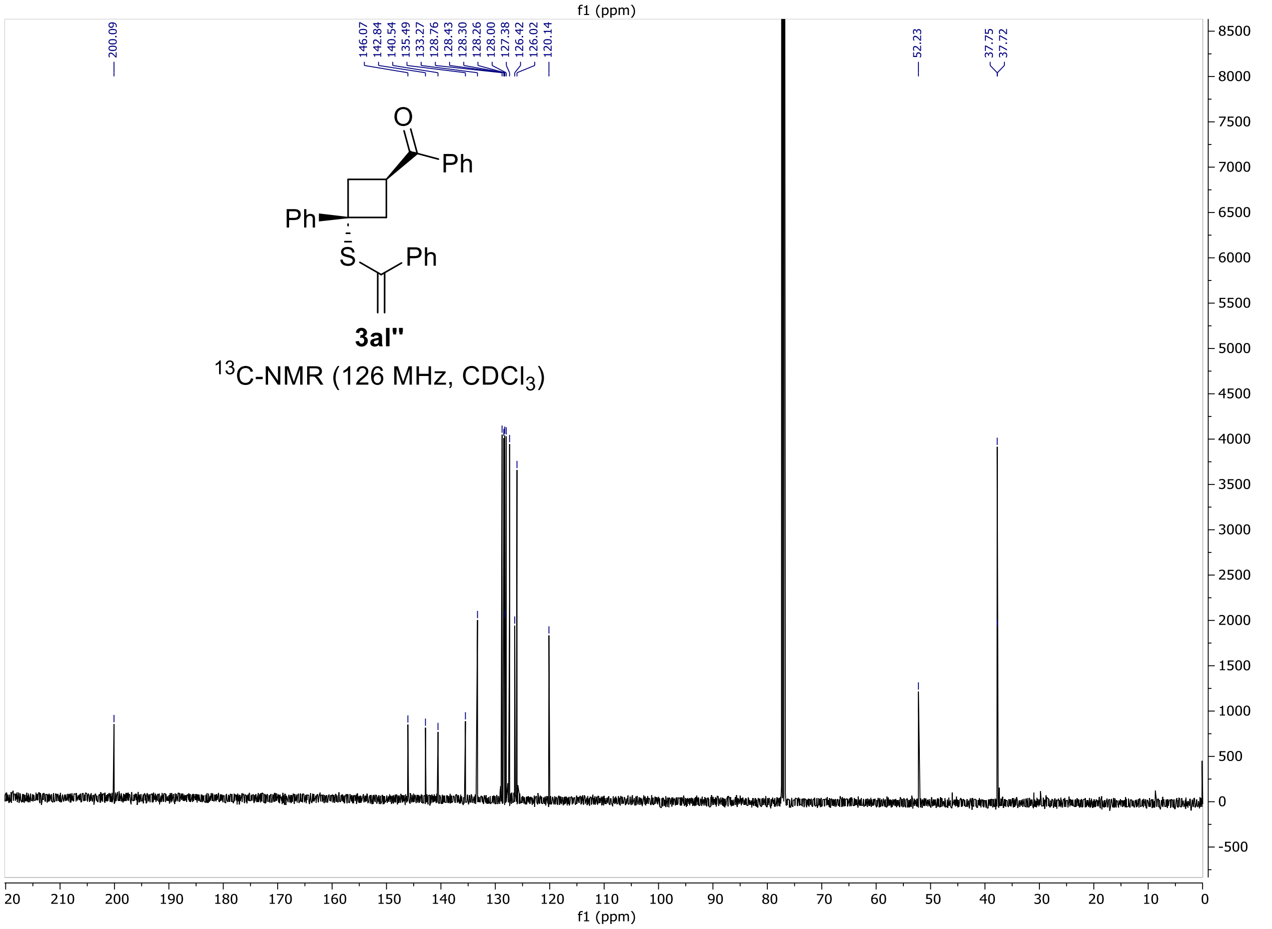
3al''
^{13}C-NMR (126 MHz, CDCl$_3$)
200.09
146.07
142.84
140.54
135.49
133.27
128.76
128.43
128.30
128.26
128.00
127.38
126.42
126.02
120.14
52.23
37.75
37.72
f1 (ppm)

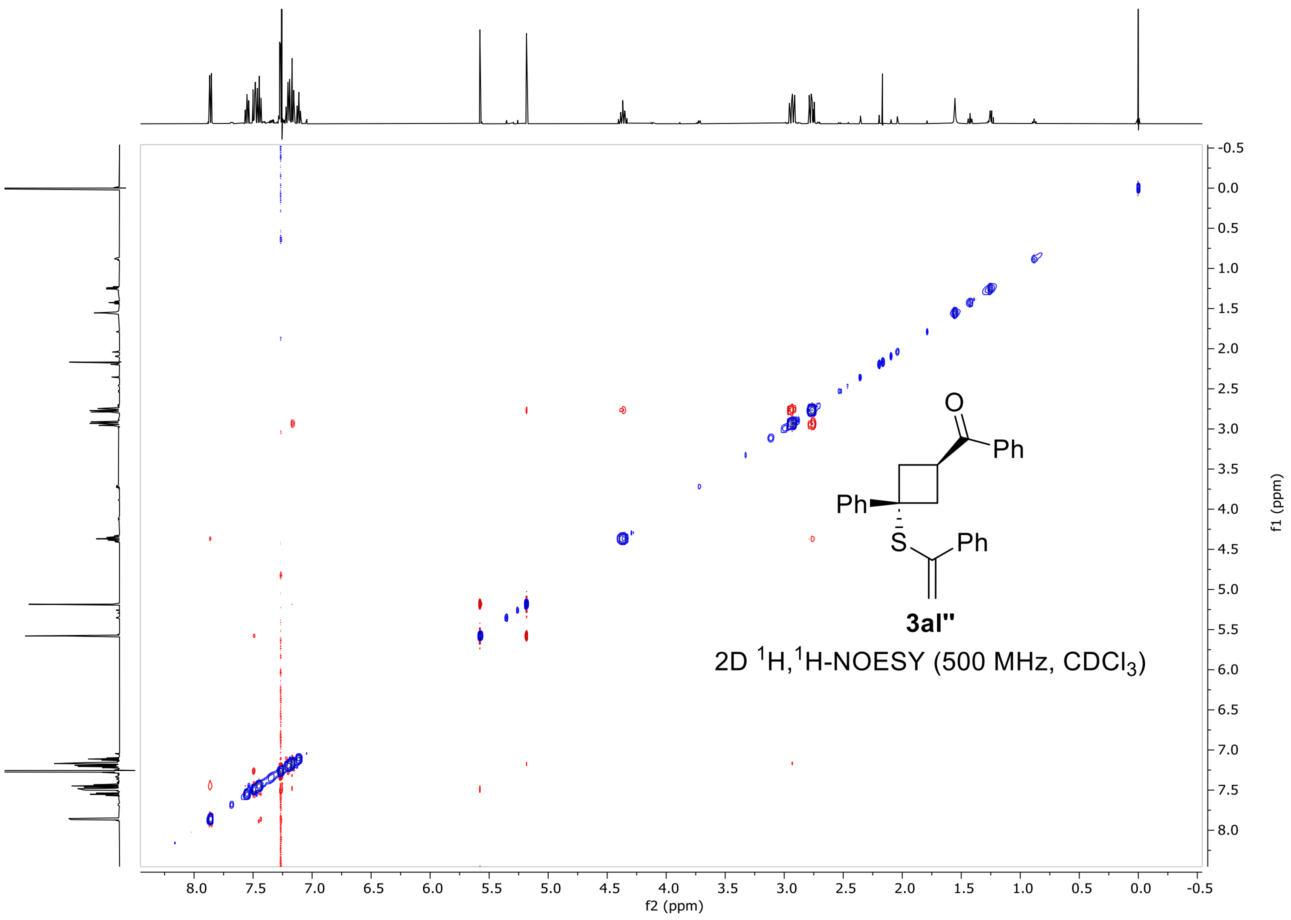

O
Ph
Ph
S
Ph
3al''
2D ^{1}H,^{1}H-NOESY (500 MHz, $CDCl_3$)
f1 (ppm)
f2 (ppm)

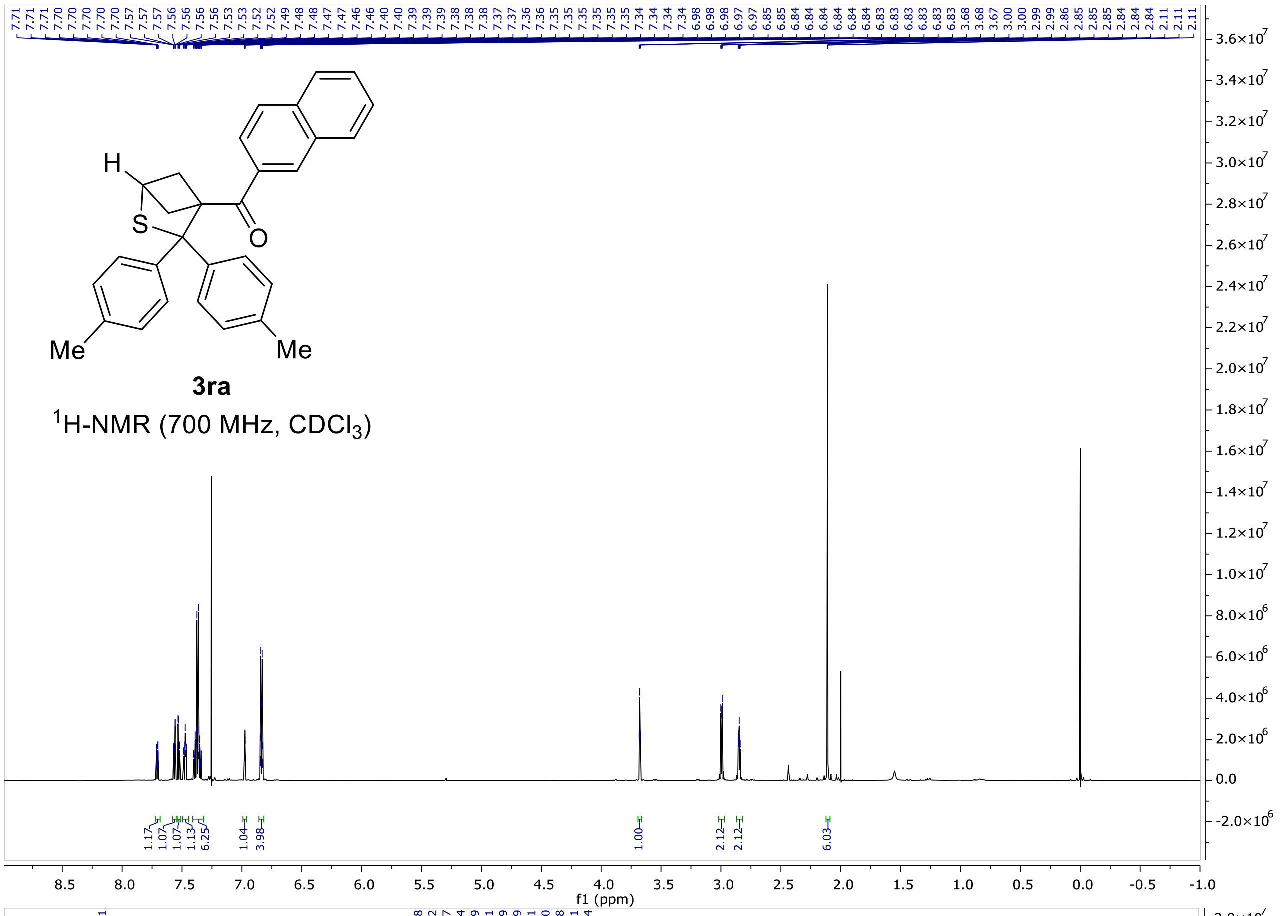
H
S
O
Me
Me
3ra
^{1}H-NMR (700 MHz, $CDCl_3$)
f1 (ppm)

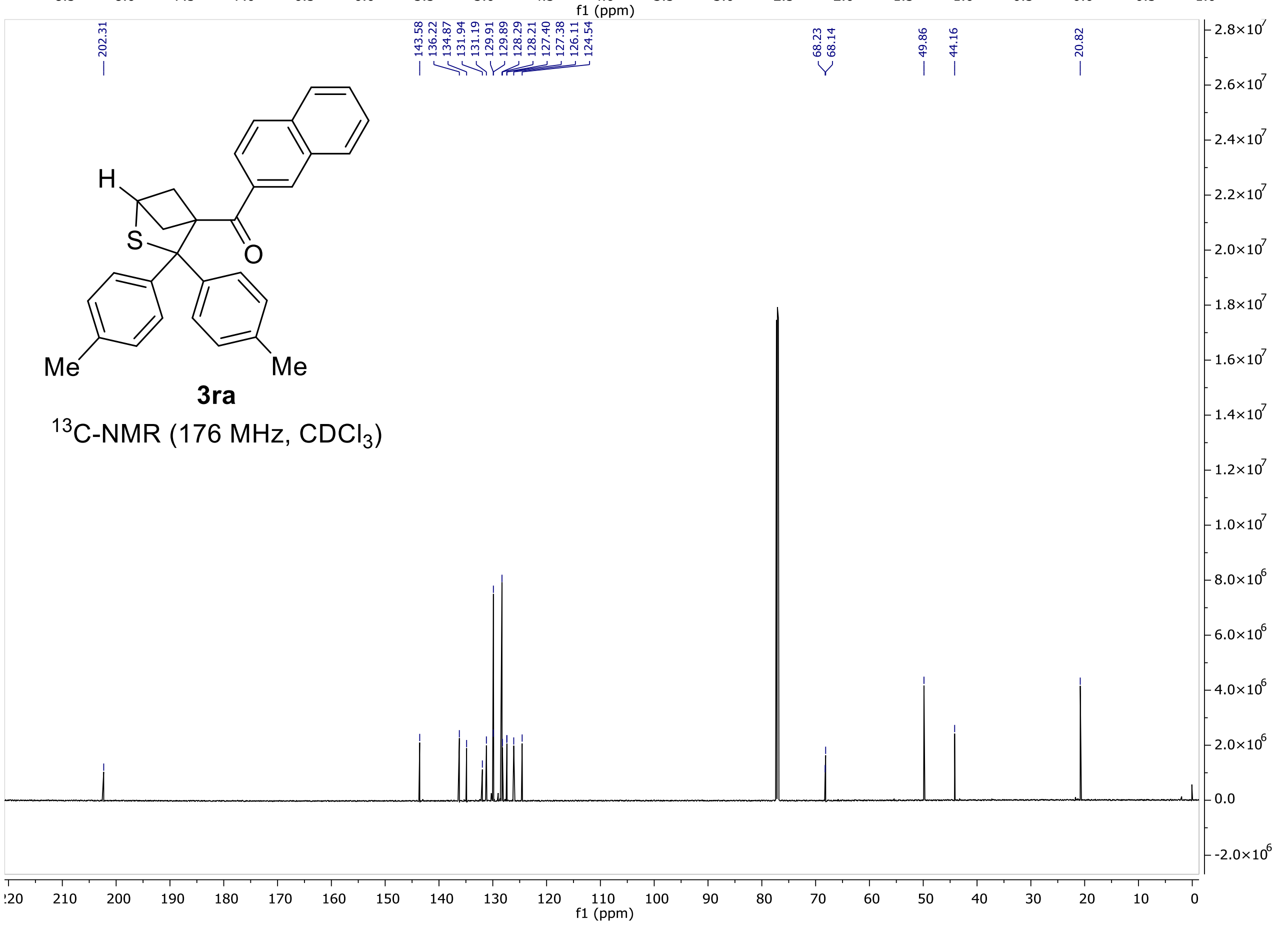
202.31
143.58
136.22
134.87
131.94
131.19
129.91
129.89
128.29
128.21
127.40
127.38
126.11
124.54
68.23
68.14
49.86
44.16
20.82
H
S
O
Me
Me
3ra
^{13}C-NMR (176 MHz, $CDCl_3$)
f1 (ppm)

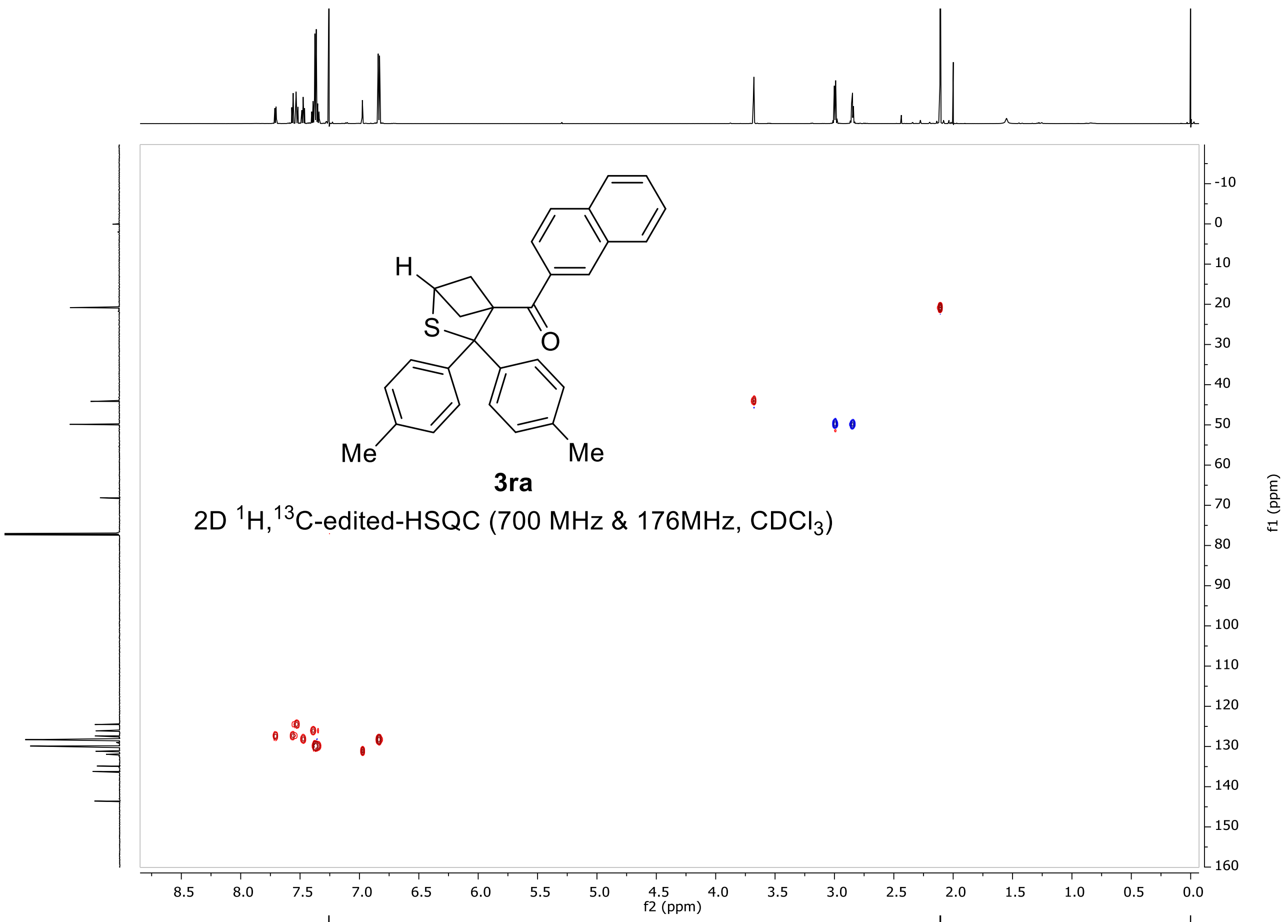
H
S
O
Me
Me
3ra
2D 1H,13C-edited-HSQC (700 MHz & 176MHz, CDCl3)
f2 (ppm)
f1 (ppm)

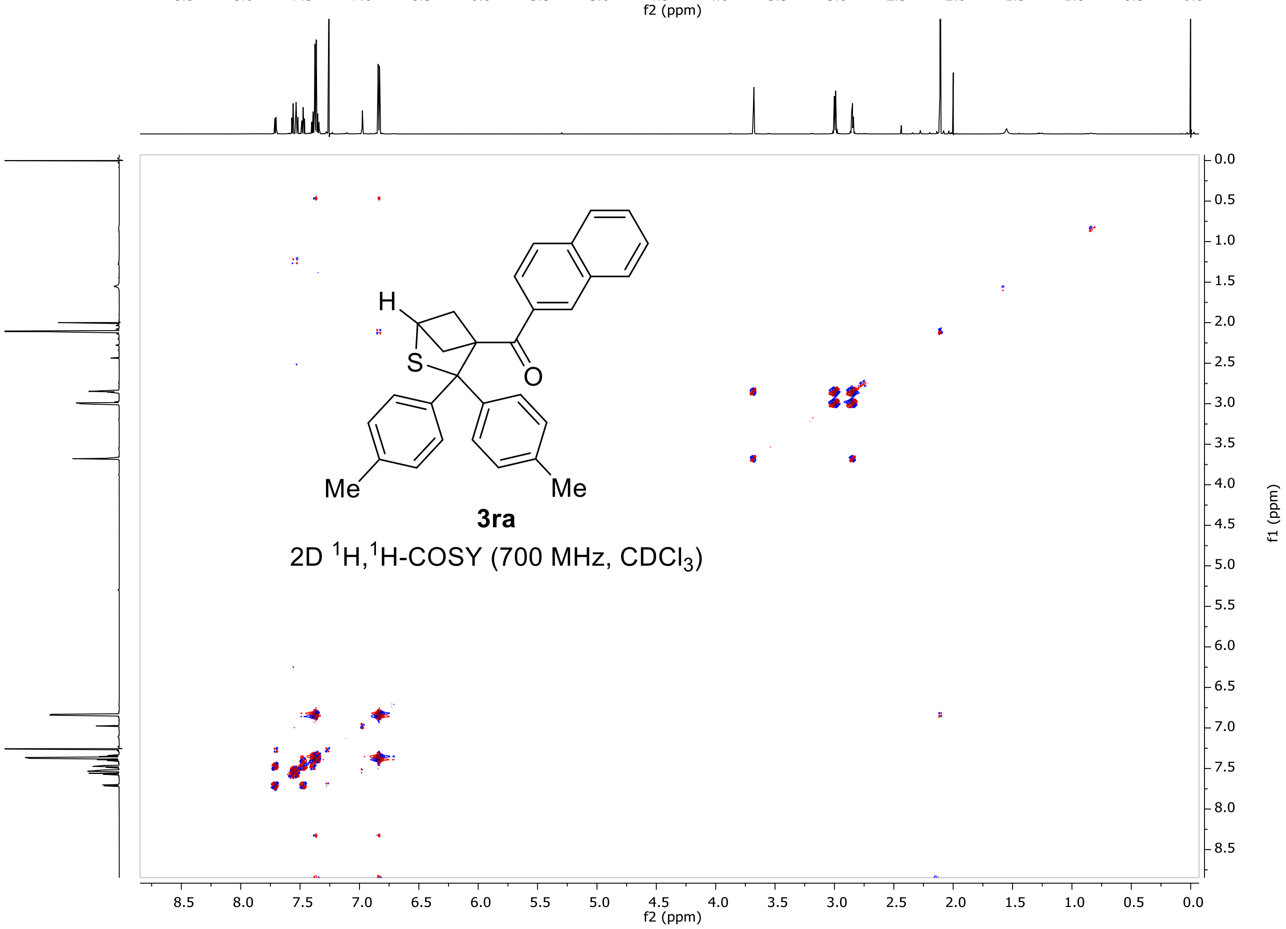
H
S
O
Me
Me
3ra
2D 1H,1H-COSY (700 MHz, CDCl3)
f2 (ppm)
f1 (ppm)

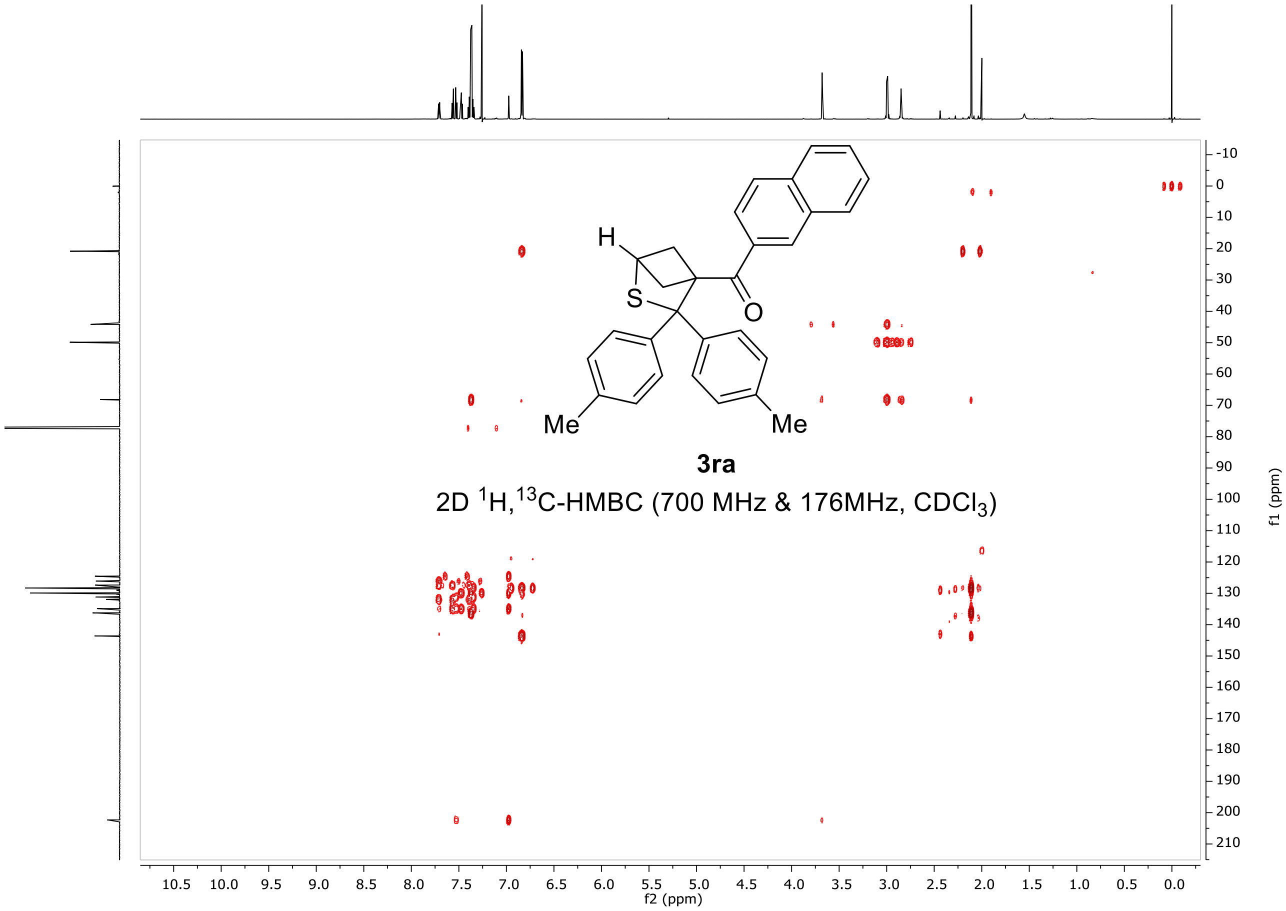
H
S
O
Me
Me
3ra
2D $^{1}H,^{13}C$-HMBC (700 MHz & 176MHz, $CDCl_3$)
f2 (ppm)
f1 (ppm)

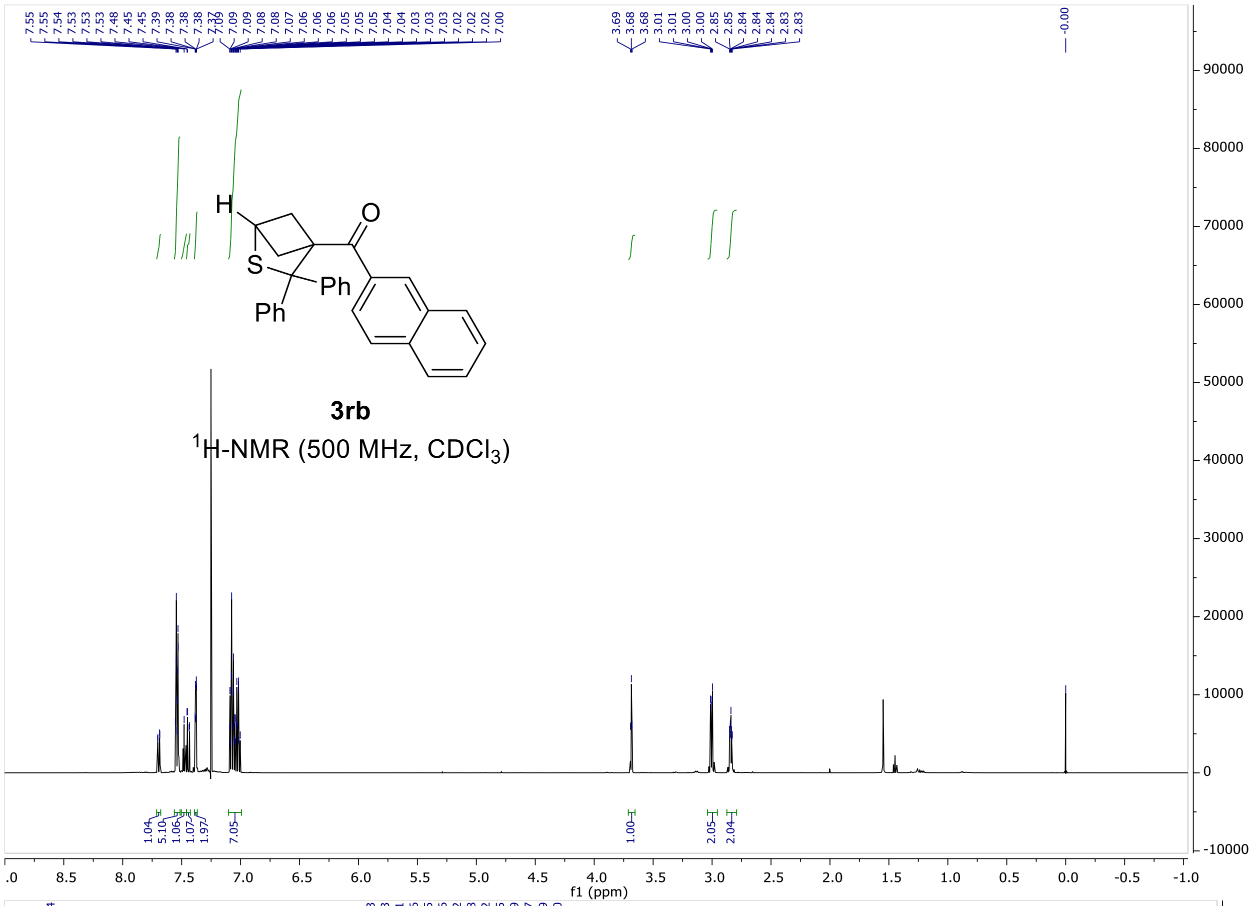

3rb
^{1}H-NMR (500 MHz, CDCl$_3$)
f1 (ppm)

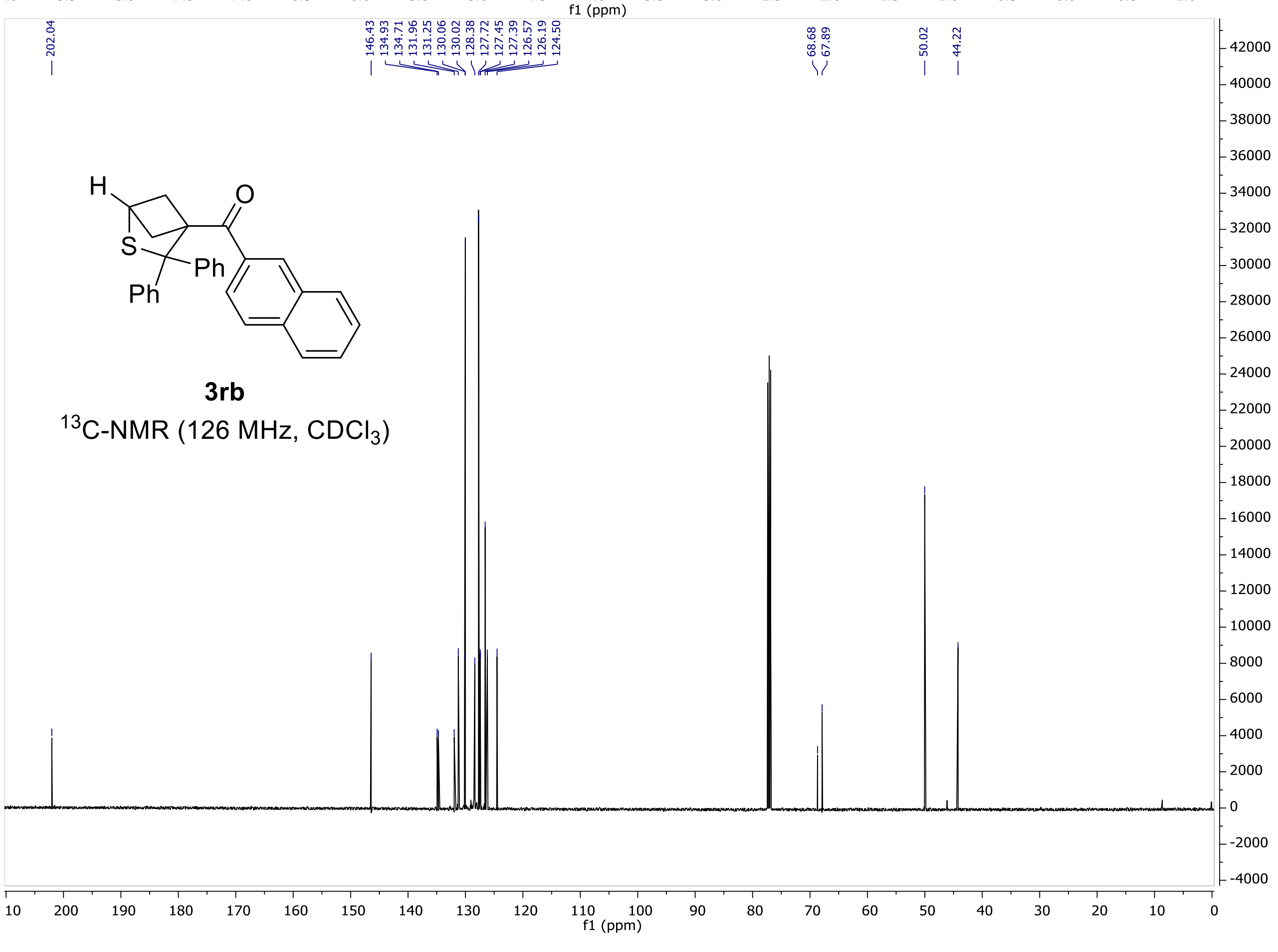

3rb
^{13}C-NMR (126 MHz, CDCl$_3$)
f1 (ppm)

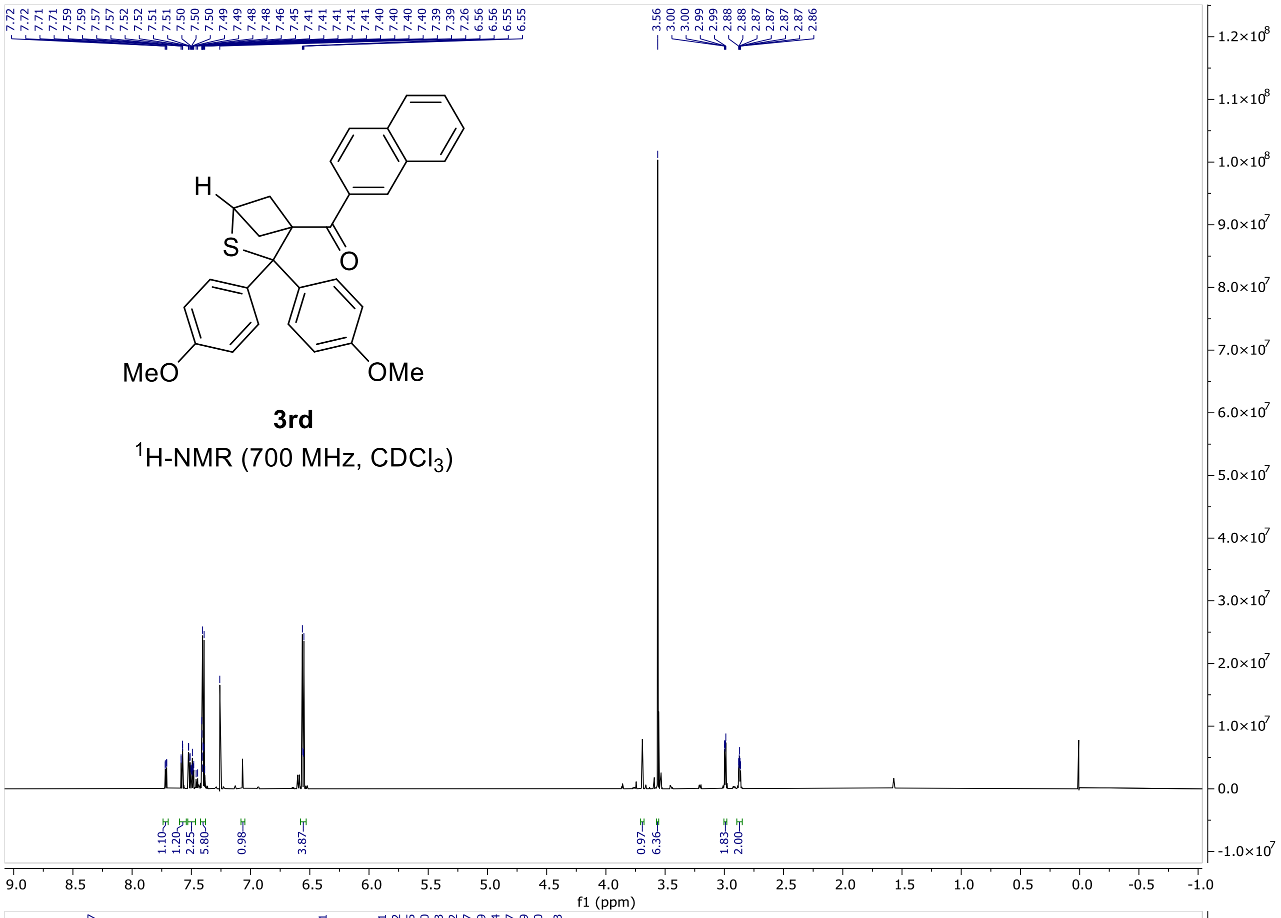
7.72 7.72 7.71 7.71 7.59 7.59 7.57 7.57 7.52 7.52 7.51 7.51 7.50 7.50 7.50 7.49 7.49 7.48 7.46 7.45 7.41 7.41 7.41 7.41 7.41 7.40 7.40 7.40 7.39 7.39 7.26 6.56 6.56 6.55 6.55
3.56 3.00 3.00 2.99 2.99 2.88 2.88 2.87 2.87 2.87 2.86
H
S
O
MeO
OMe
3rd
^{1}H-NMR (700 MHz, $CDCl_3$)
1.10 1.20 2.25 5.80 0.98 3.87 0.97 6.36 1.83 2.00
f1 (ppm)

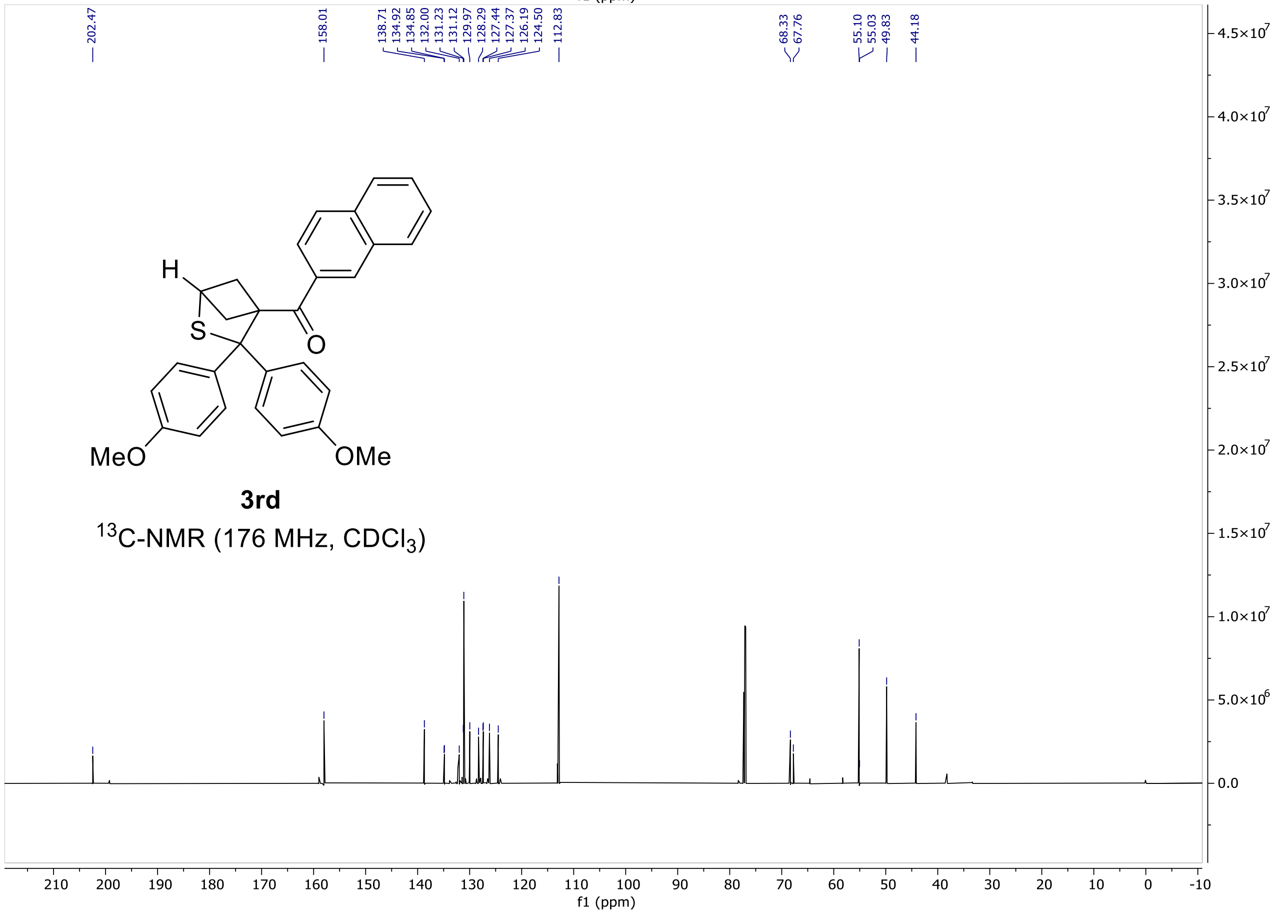
202.47
158.01
138.71 134.92 134.85 132.00 131.23 131.12 129.97 128.29 127.44 127.37 126.19 124.50
112.83
68.33 67.76
55.10 55.03 49.83
44.18
H
S
O
MeO
OMe
3rd
^{13}C-NMR (176 MHz, $CDCl_3$)
f1 (ppm)

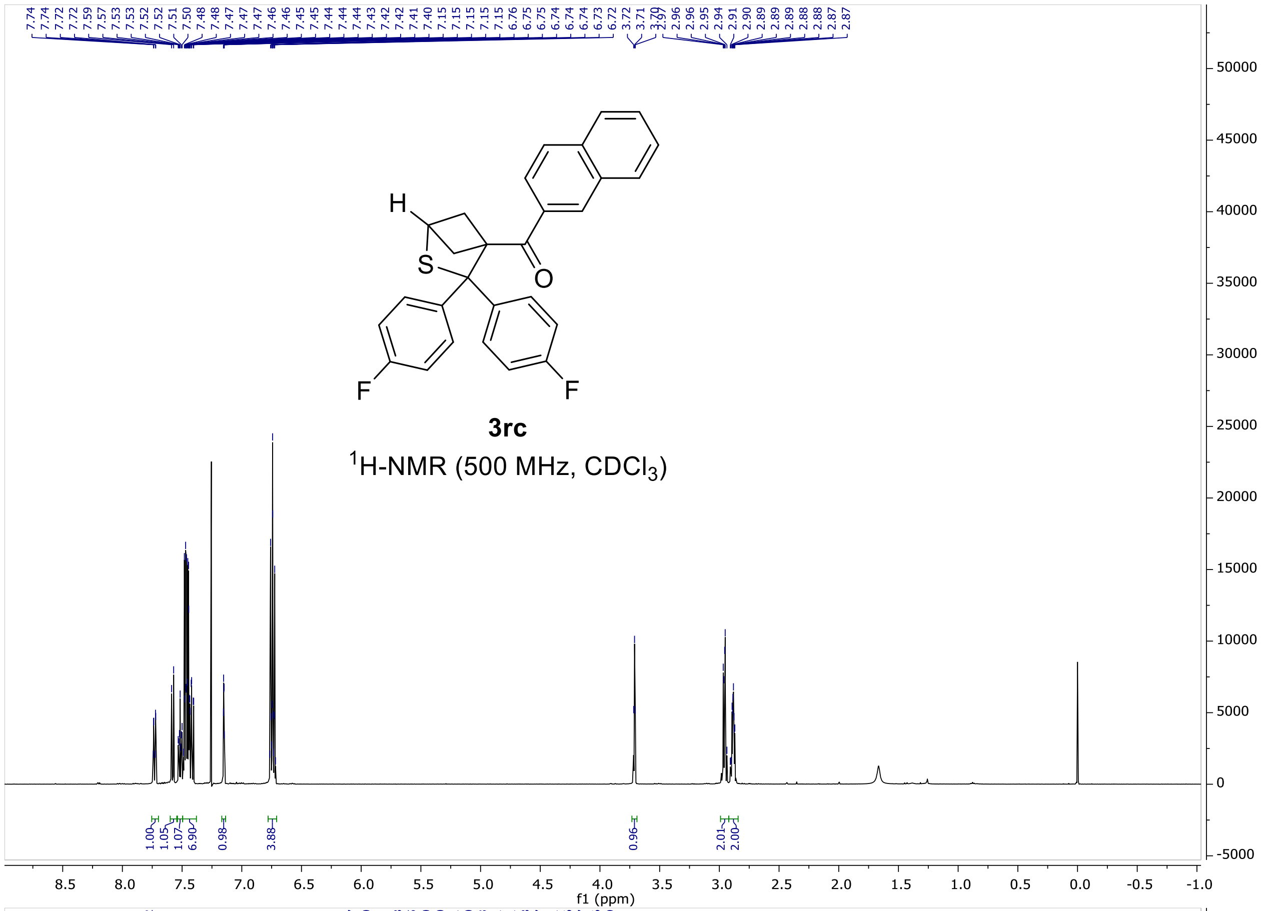

3rc
^{1}H-NMR (500 MHz, CDCl$_3$)
f1 (ppm)

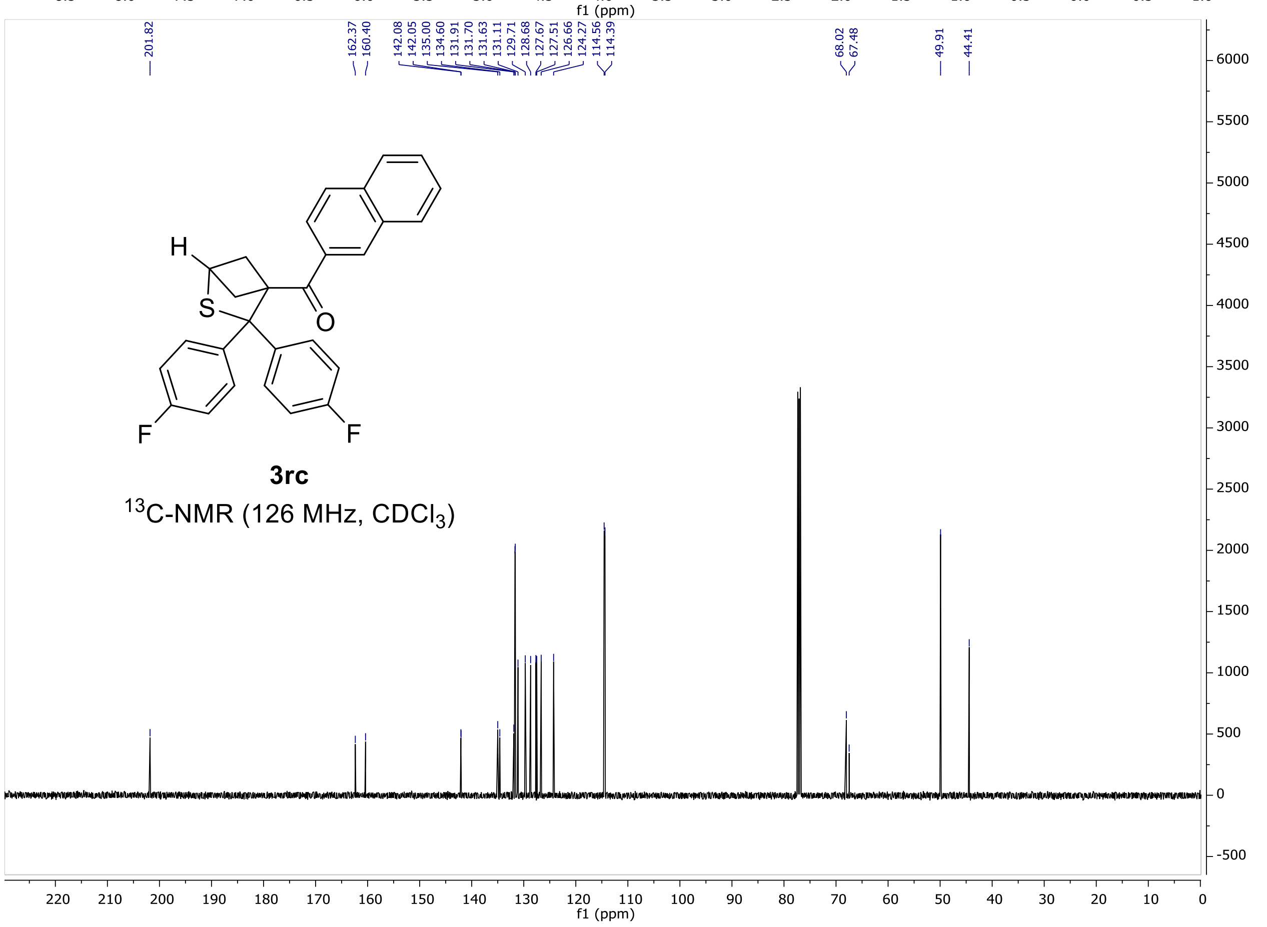

3rc
^{13}C-NMR (126 MHz, CDCl$_3$)
f1 (ppm)

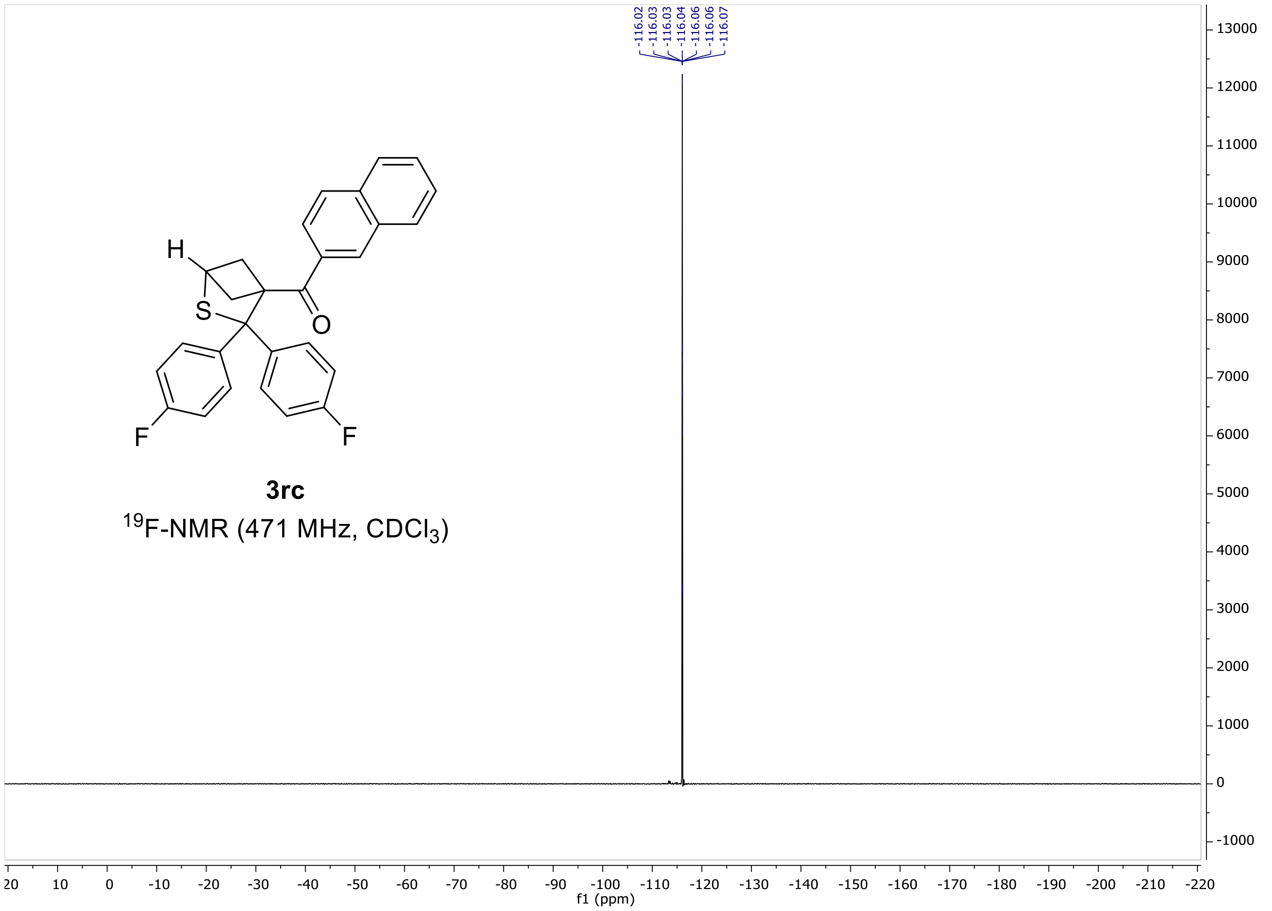

-116.02
-116.03
-116.03
-116.04
-116.06
-116.06
-116.07
H
S
O
F
F
3rc
^{19}F-NMR (471 MHz, CDCl$_3$)
f1 (ppm)

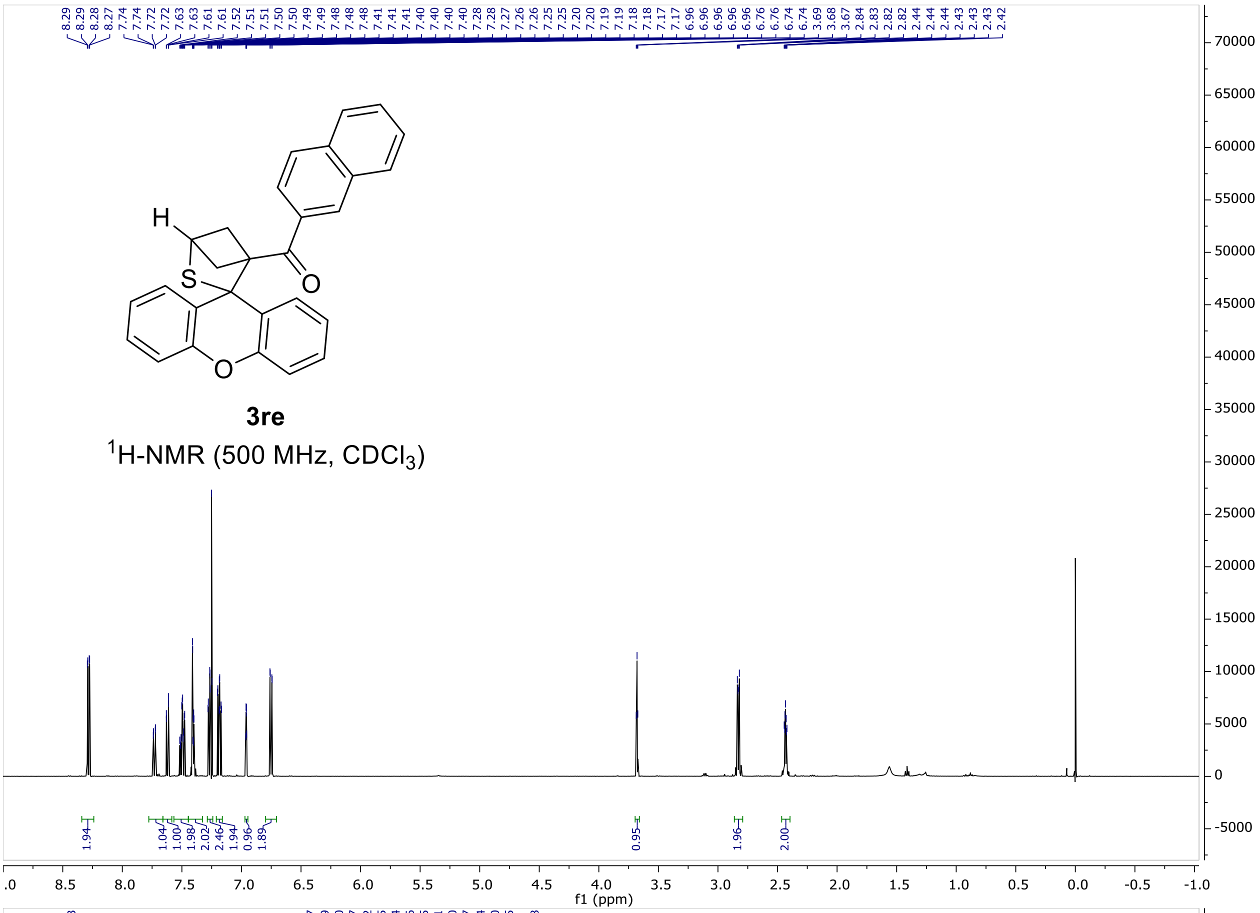
8.29 8.29 8.28 8.27 7.74 7.74 7.72 7.72 7.63 7.63 7.61 7.61 7.52 7.51 7.51 7.50 7.50 7.49 7.49 7.48 7.48 7.41 7.41 7.40 7.40 7.40 7.40 7.28 7.28 7.27 7.26 7.26 7.25 7.25 7.20 7.20 7.19 7.19 7.18 7.18 7.17 7.17 6.96 6.96 6.96 6.96 6.96 6.76 6.76 6.74 6.74 3.69 3.68 3.67 2.84 2.83 2.82 2.82 2.44 2.44 2.44 2.43 2.43 2.42
H
S
O
O
3re
1H-NMR (500 MHz, CDCl3)
1.94 1.04 1.00 1.98 2.02 2.46 1.94 0.96 1.89 0.95 1.96 2.00
f1 (ppm)

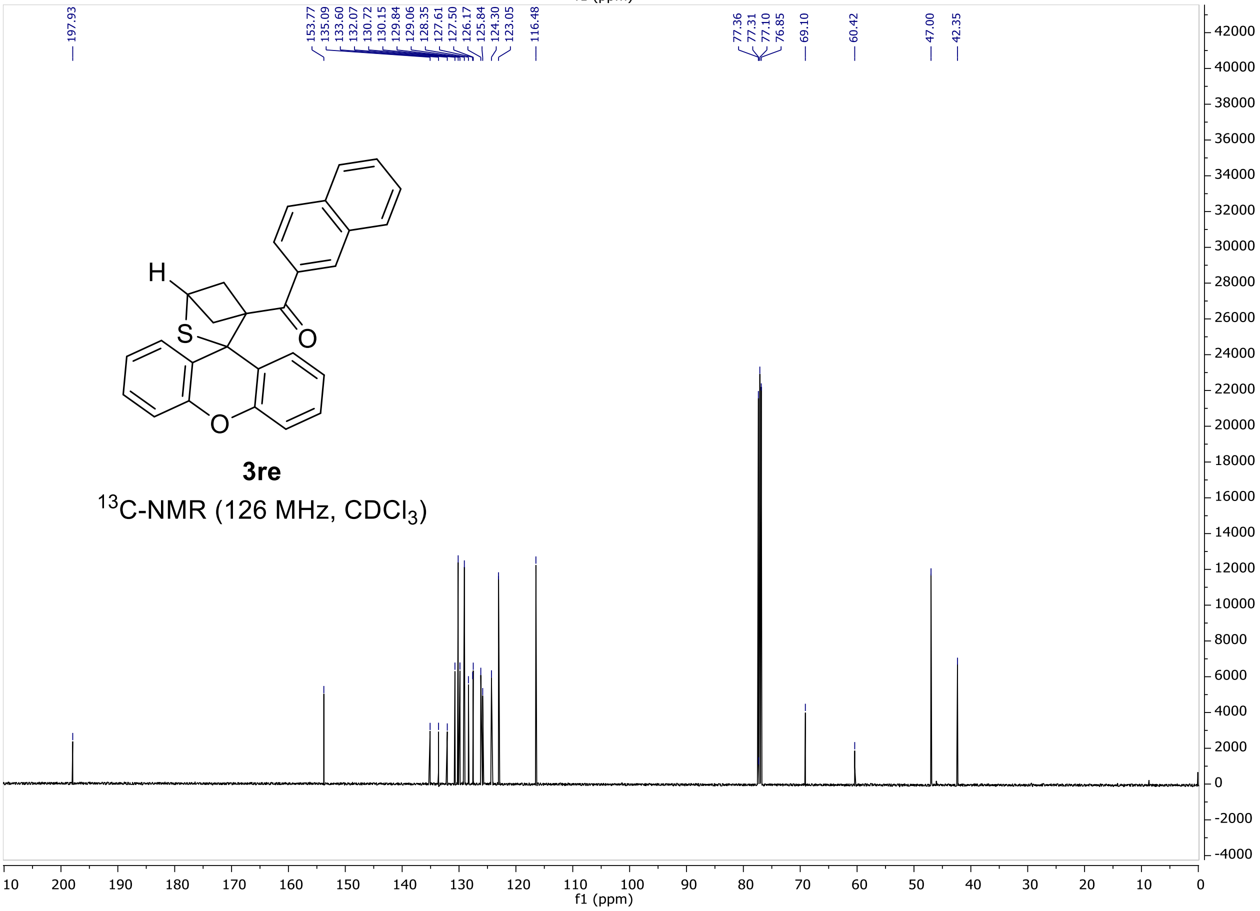
197.93 153.77 135.09 133.60 132.07 130.72 130.15 129.84 129.06 128.35 127.61 127.50 126.17 125.84 124.30 123.05 116.48 77.36 77.31 77.10 76.85 69.10 60.42 47.00 42.35
H
S
O
O
3re
13C-NMR (126 MHz, CDCl3)
f1 (ppm)

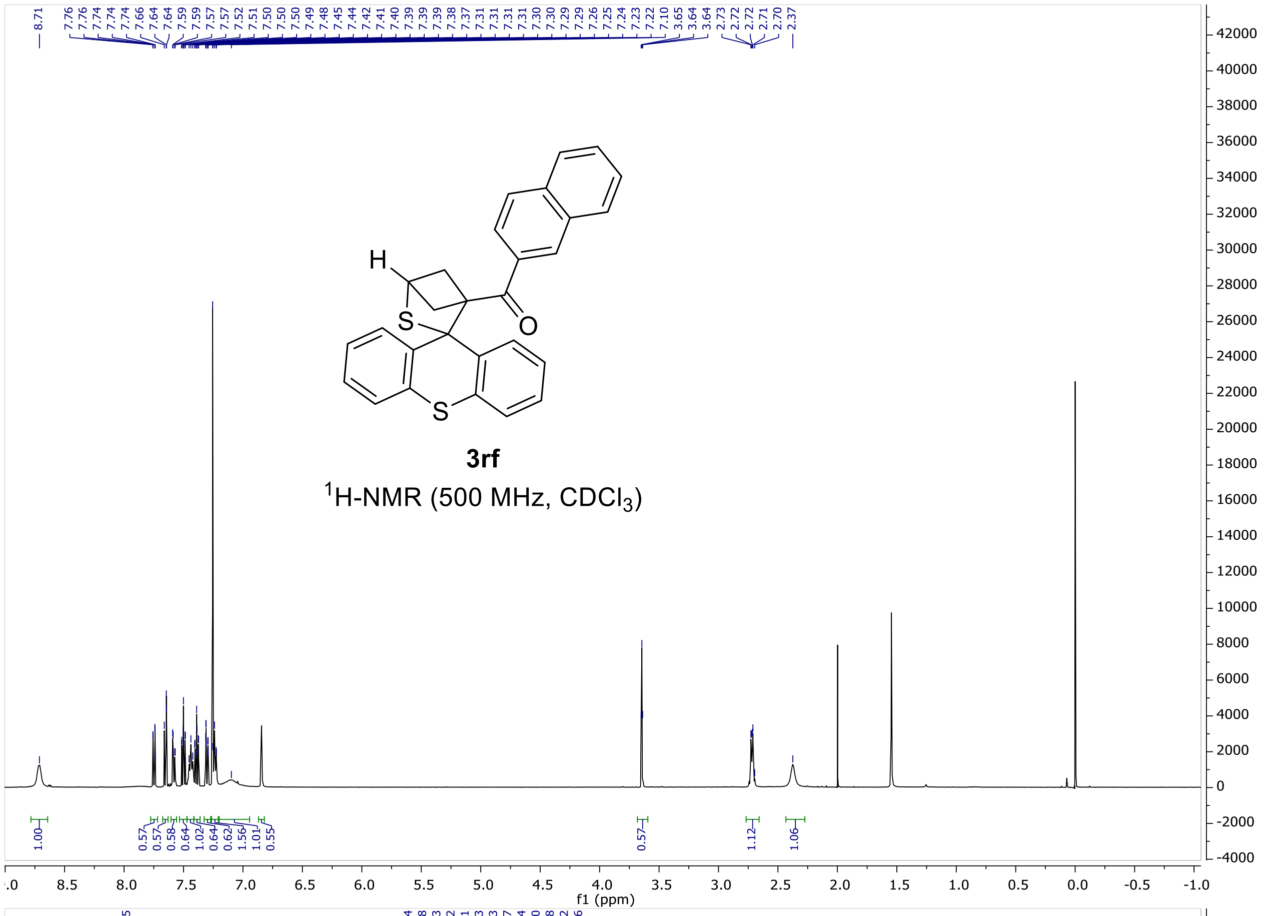
3rf
^{1}H-NMR (500 MHz, $CDCl_3$)
f1 (ppm)

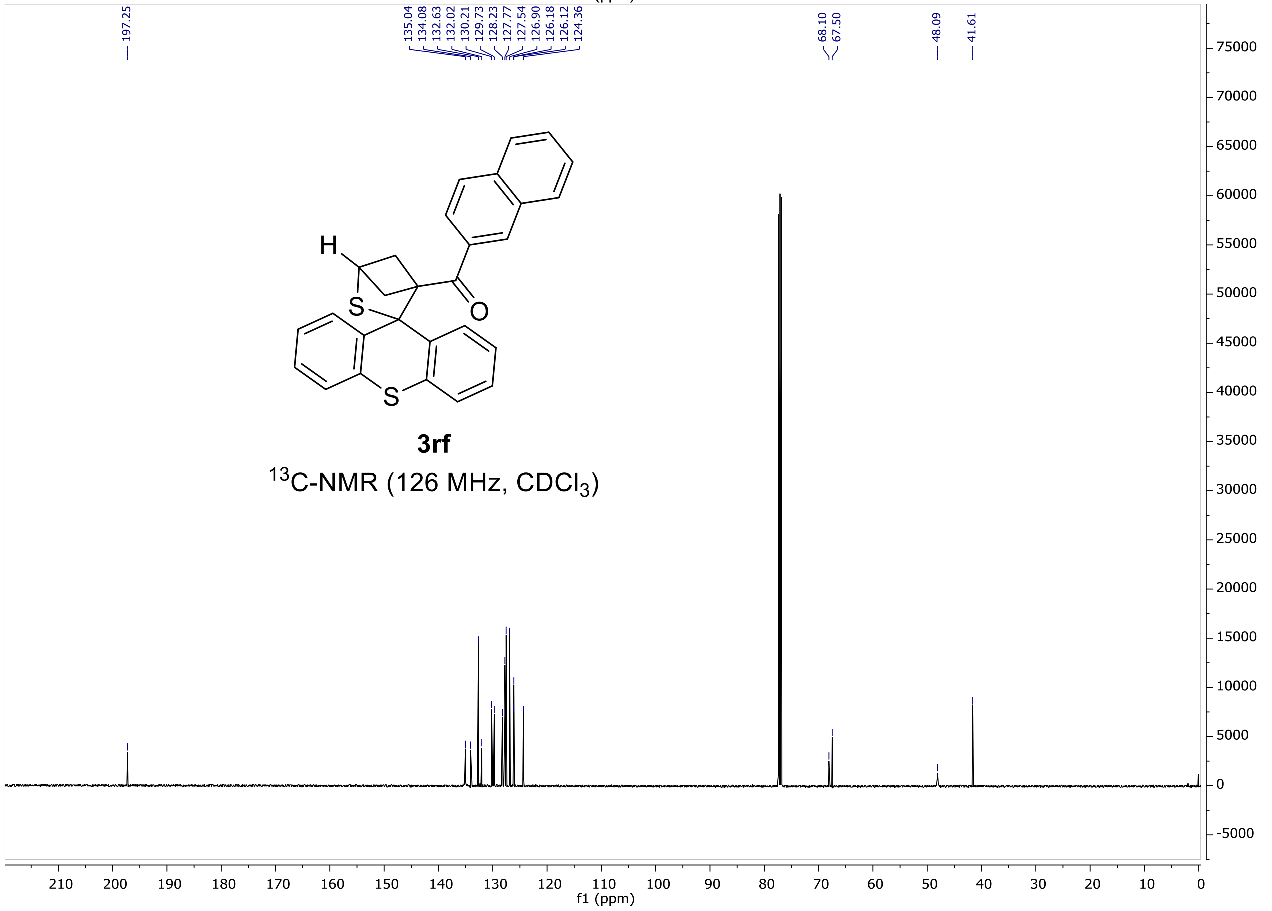
3rf
^{13}C-NMR (126 MHz, $CDCl_3$)
f1 (ppm)

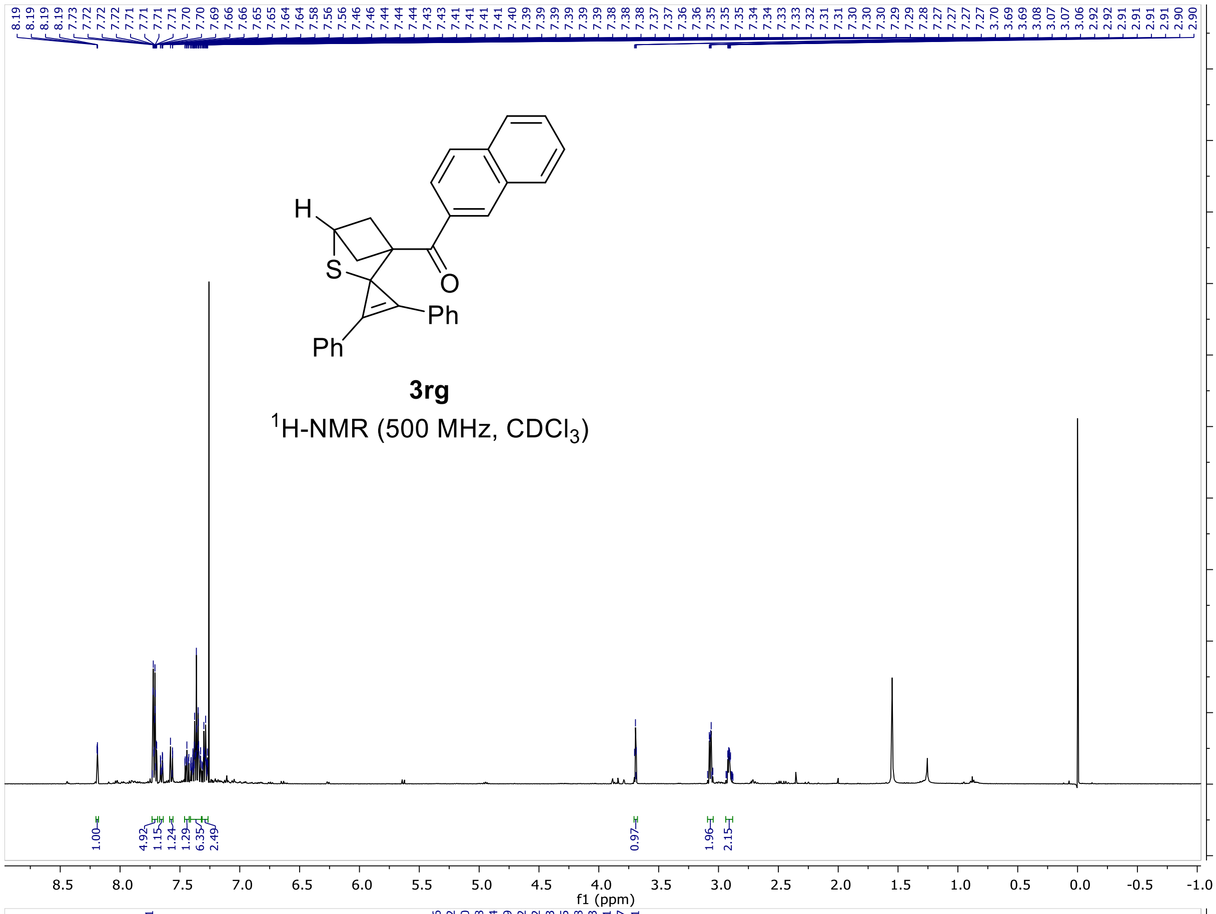
8.19 8.19 8.19 8.19 7.73 7.72 7.72 7.72 7.71 7.71 7.71 7.70 7.69 7.66 7.65 7.65 7.64 7.64 7.58 7.56 7.56 7.46 7.46 7.44 7.44 7.44 7.43 7.43 7.41 7.41 7.41 7.40 7.39 7.39 7.39 7.39 7.39 7.39 7.38 7.38 7.38 7.37 7.36 7.36 7.35 7.35 7.34 7.34 7.33 7.33 7.32 7.31 7.31 7.30 7.30 7.30 7.29 7.29 7.28 7.27 7.27 7.27 7.27 3.70 3.69 3.08 3.07 3.07 3.06 2.92 2.92 2.91 2.91 2.91 2.91 2.90 2.90
H
S
O
Ph
Ph
3rg
1H-NMR (500 MHz, CDCl3)
1.00 4.92 1.15 1.24 1.29 6.35 2.49 0.97 1.96 2.15
f1 (ppm)

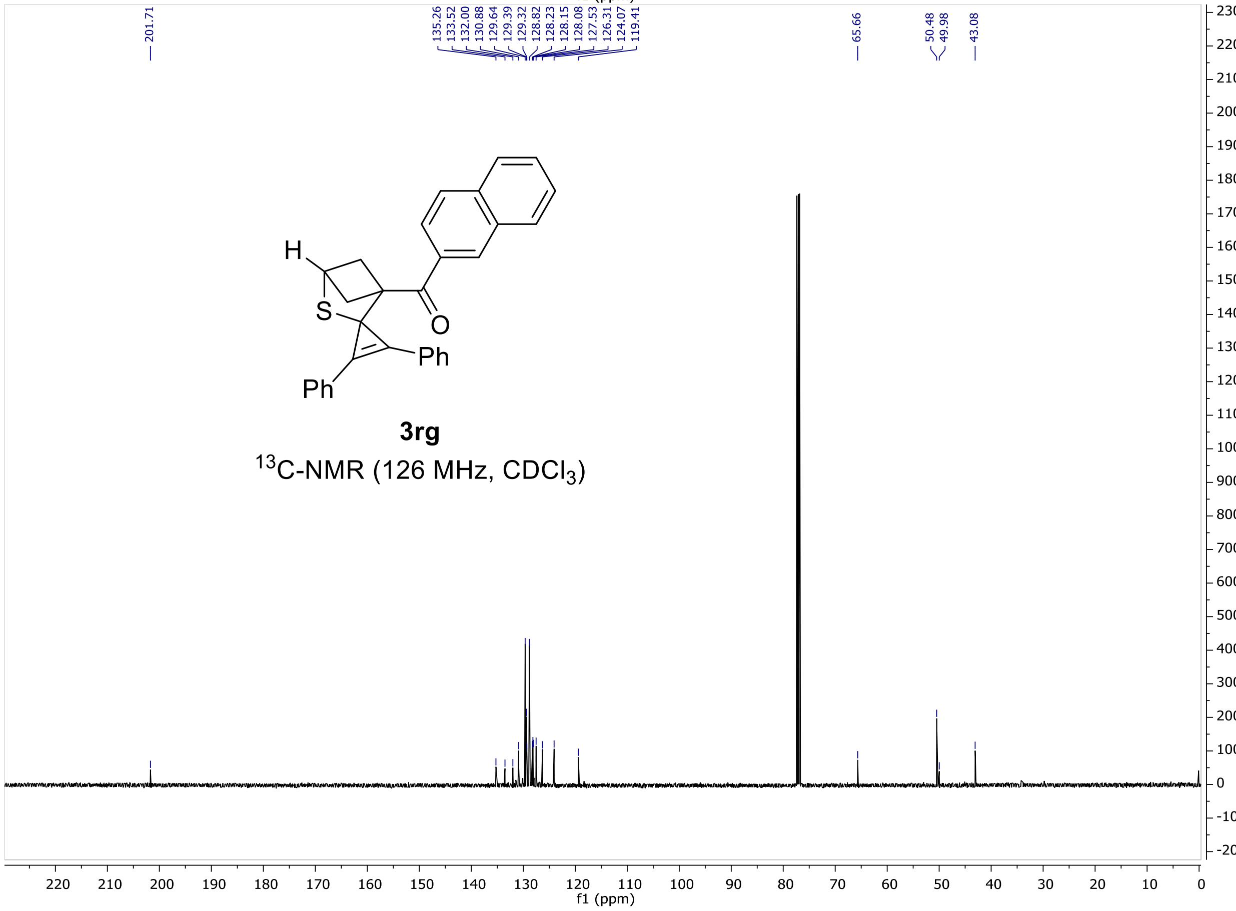
201.71
135.26 133.52 132.00 130.88 129.64 129.39 129.32 128.82 128.23 128.15 128.08 127.53 126.31 124.07 119.41
65.66
50.48 49.98
43.08
H
S
O
Ph
Ph
3rg
13C-NMR (126 MHz, CDCl3)
f1 (ppm)

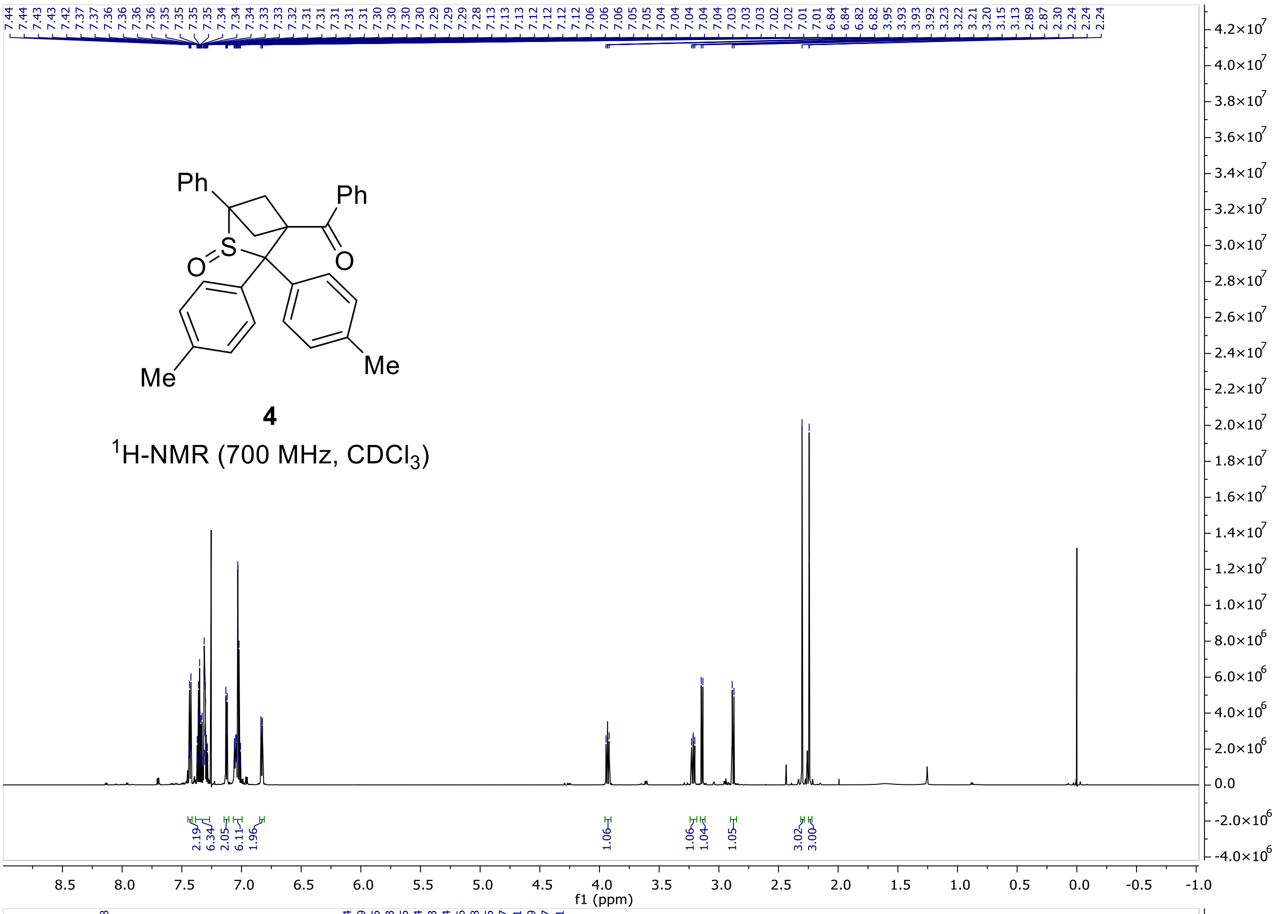
Ph
Ph
O
S
O
Me
Me
4
1H-NMR (700 MHz, CDCl3)
2.19
6.34
2.05
6.11
1.96
1.06
1.06
1.04
1.05
3.02
3.00
f1 (ppm)

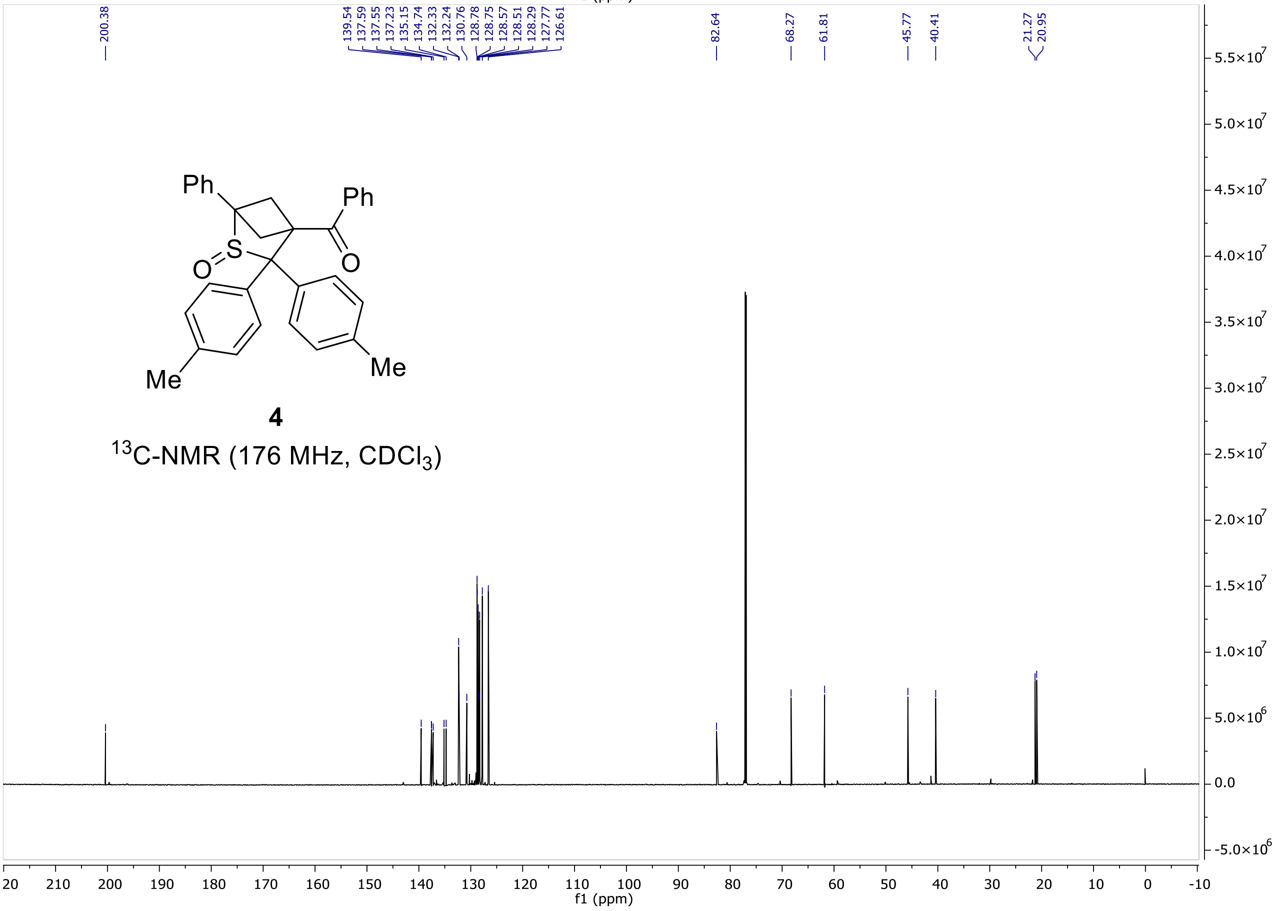
200.38
139.54
137.59
137.55
137.23
135.15
134.74
132.33
132.24
130.76
128.78
128.75
128.57
128.51
128.29
127.77
126.61
82.64
68.27
61.81
45.77
40.41
21.27
20.95
Ph
Ph
O
S
O
Me
Me
4
13C-NMR (176 MHz, CDCl3)
f1 (ppm)

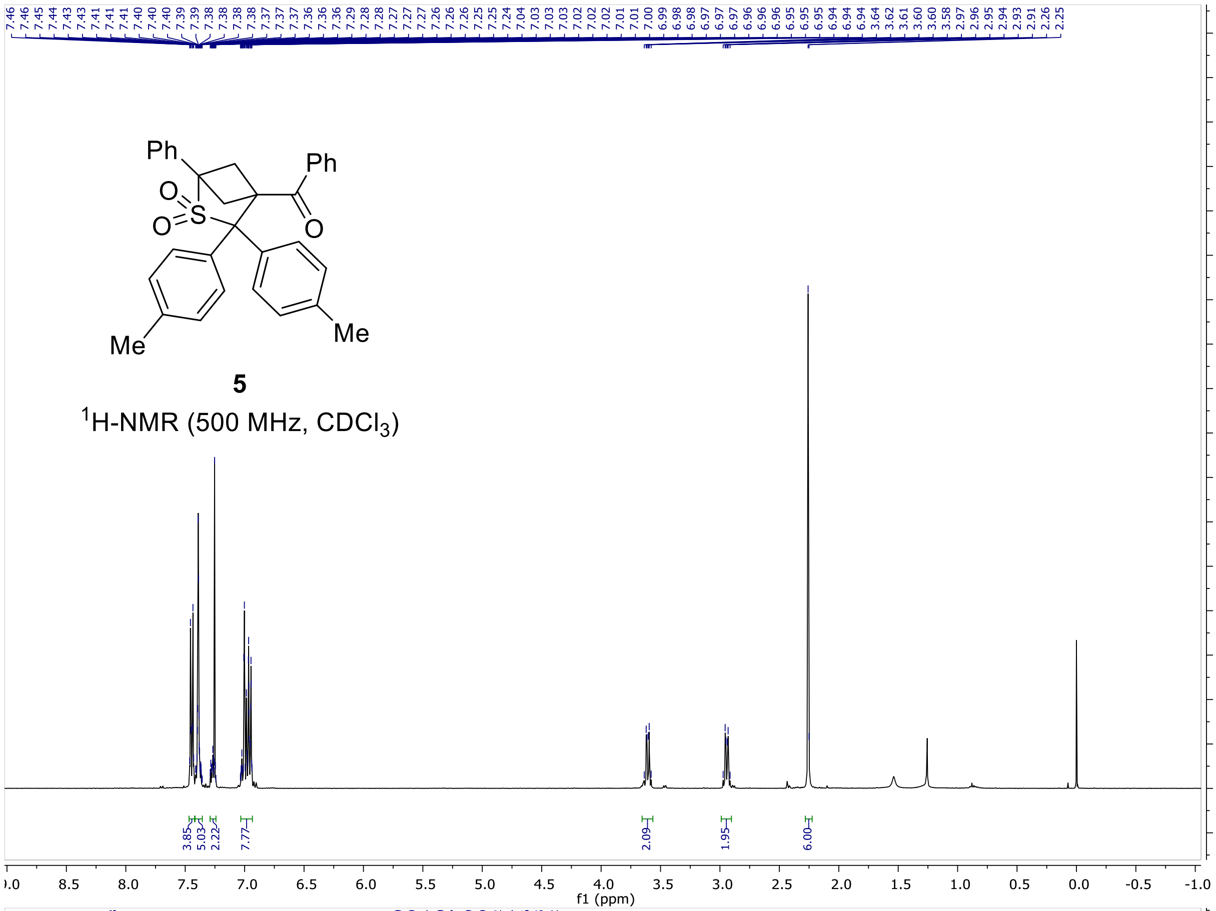
7.46 7.45 7.44 7.43 7.43 7.41 7.41 7.40 7.40 7.39 7.39 7.38 7.38 7.38 7.37 7.37 7.36 7.36 7.36 7.29 7.28 7.28 7.27 7.27 7.26 7.26 7.26 7.25 7.25 7.24 7.04 7.03 7.03 7.03 7.02 7.02 7.02 7.01 7.01 7.00 6.99 6.98 6.98 6.97 6.97 6.97 6.96 6.96 6.96 6.95 6.95 6.95 6.94 6.94 6.94 3.64 3.62 3.61 3.60 3.60 3.58 2.97 2.96 2.95 2.94 2.93 2.91 2.26 2.25
Ph
Ph
O
O=S
O
Me
Me
5
^{1}H-NMR (500 MHz, CDCl$_3$)
3.85 5.03 2.22 7.77 2.09 1.95 6.00
f1 (ppm)

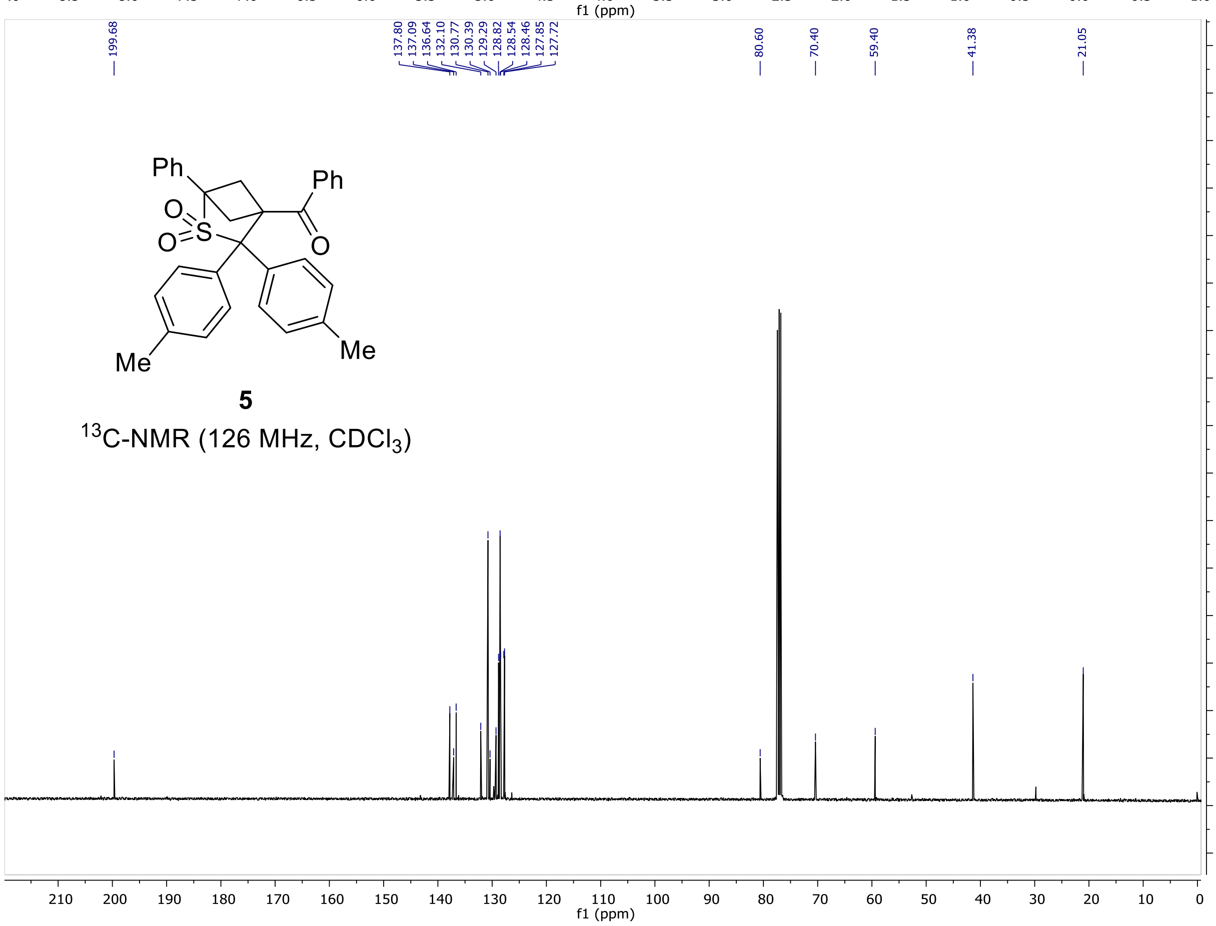
199.68
137.80 137.09 136.64 132.10 130.77 130.39 129.29 128.82 128.54 128.46 127.85 127.72
80.60
70.40
59.40
41.38
21.05
Ph
Ph
O
O=S
O
Me
Me
5
^{13}C-NMR (126 MHz, CDCl$_3$)
f1 (ppm)

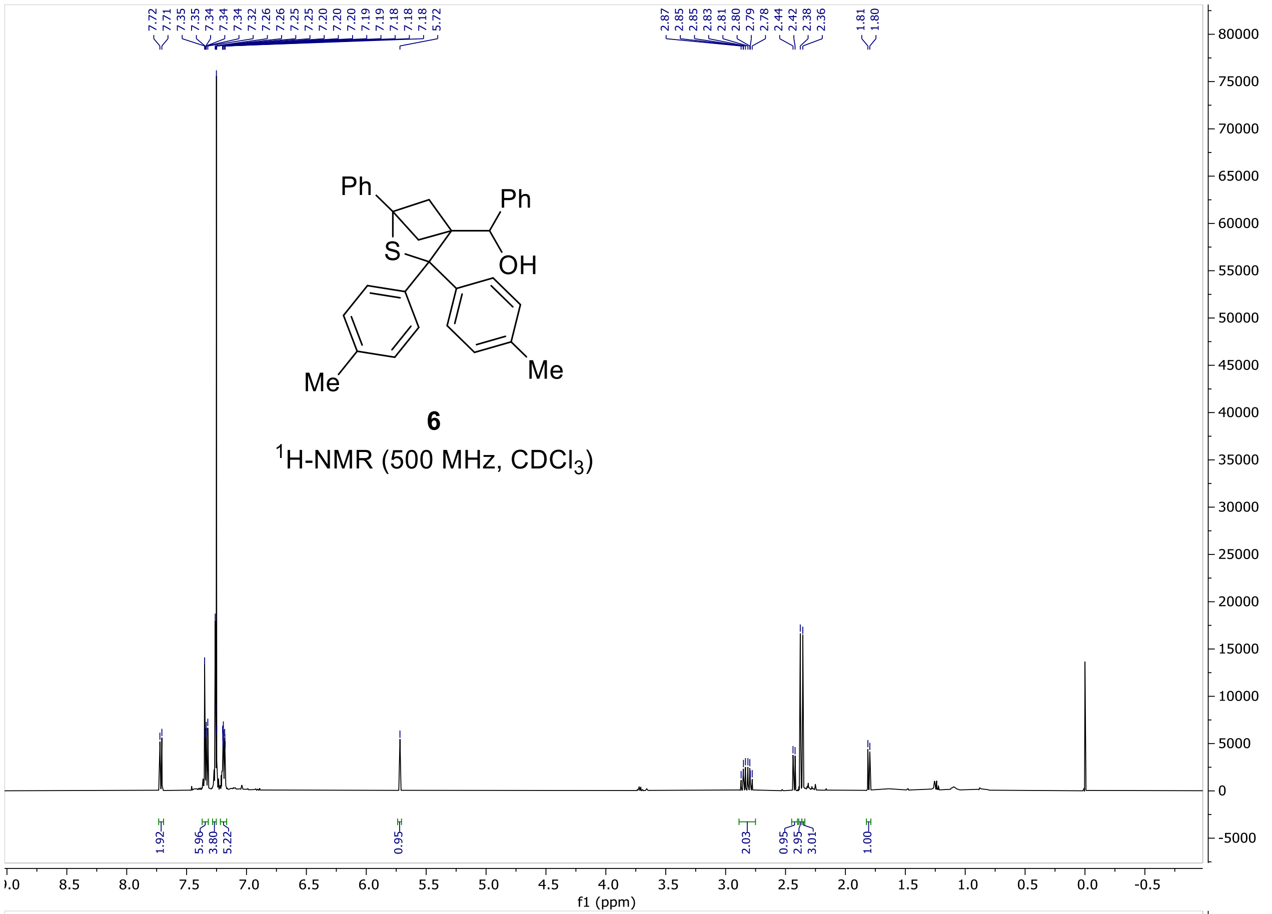

Ph
Ph
S
OH
Me
Me
6
^{1}H-NMR (500 MHz, CDCl$_3$)
f1 (ppm)

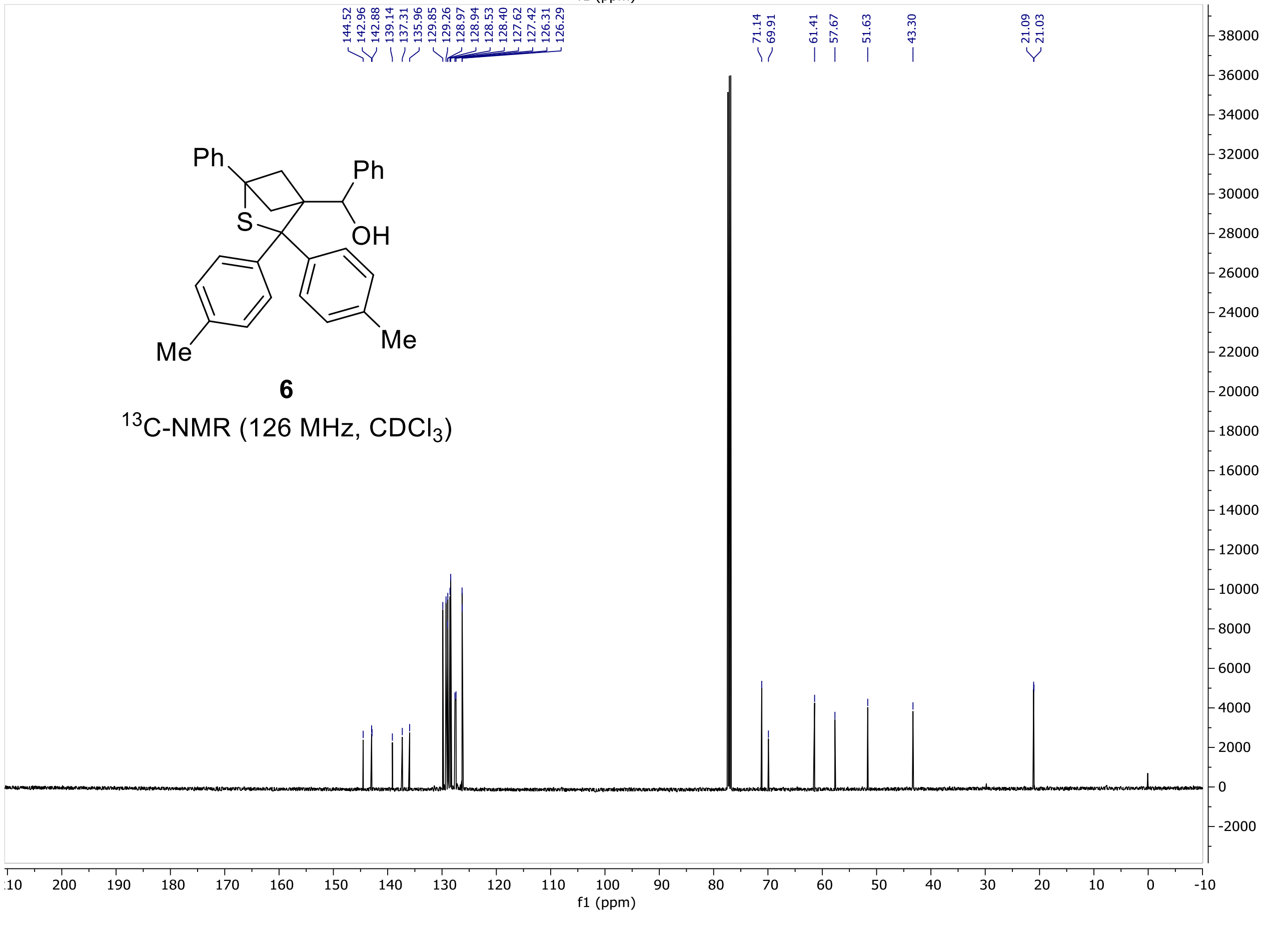

Ph
Ph
S
OH
Me
Me
6
^{13}C-NMR (126 MHz, CDCl$_3$)
f1 (ppm)

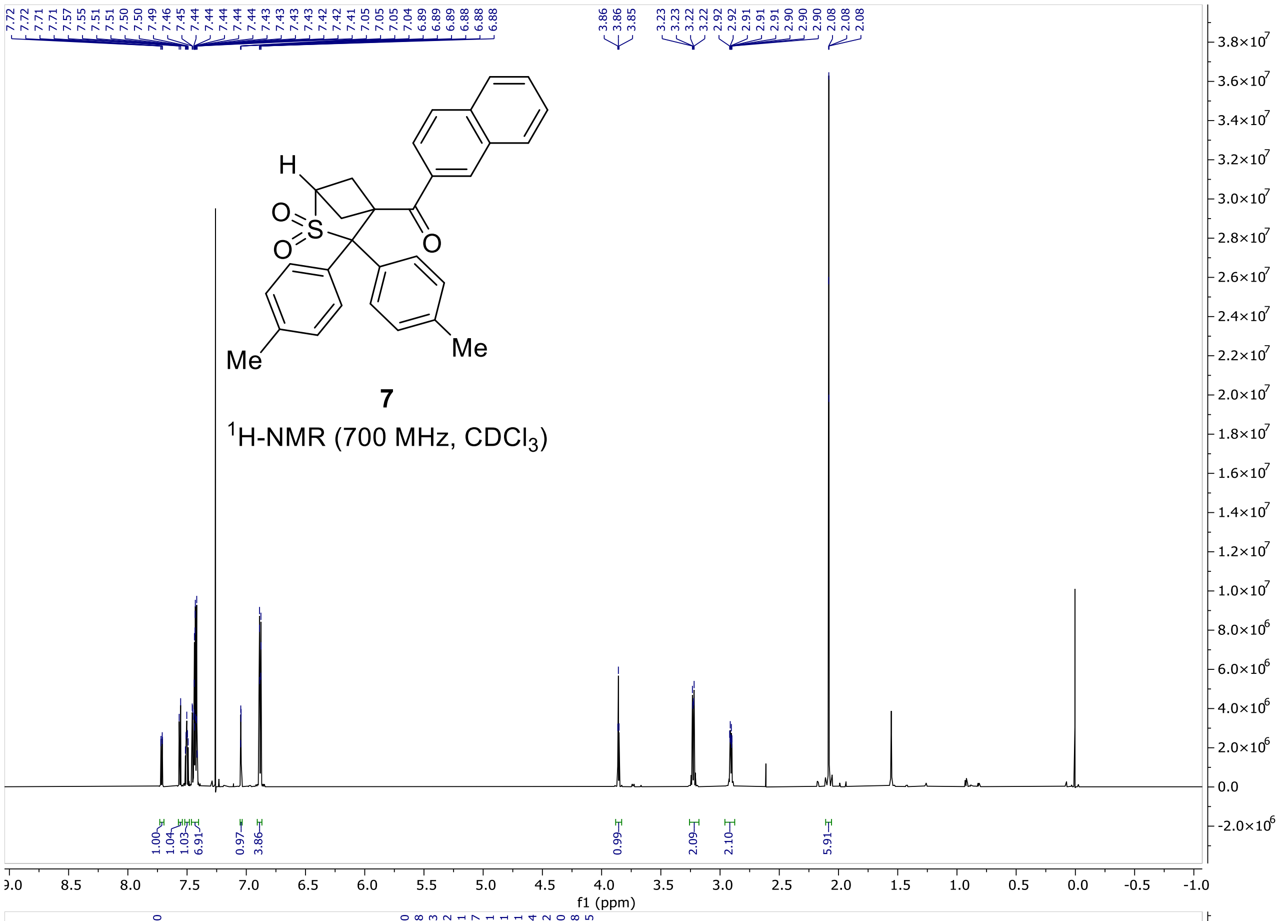

7.72 7.72 7.71 7.71 7.57 7.55 7.51 7.51 7.50 7.50 7.49 7.46 7.45 7.44 7.44 7.44 7.44 7.44 7.43 7.43 7.43 7.42 7.42 7.42 7.41 7.05 7.05 7.05 7.04 6.89 6.89 6.89 6.88 6.88 6.88
3.86 3.86 3.85
3.23 3.23 3.22 3.22 2.92 2.92 2.91 2.91 2.91 2.90 2.90 2.90 2.08 2.08 2.08
H
O
S
O
O
Me
Me
7
^{1}H-NMR (700 MHz, CDCl$_3$)
1.00 1.04 1.03 6.91 0.97 3.86 0.99 2.09 2.10 5.91
f1 (ppm)

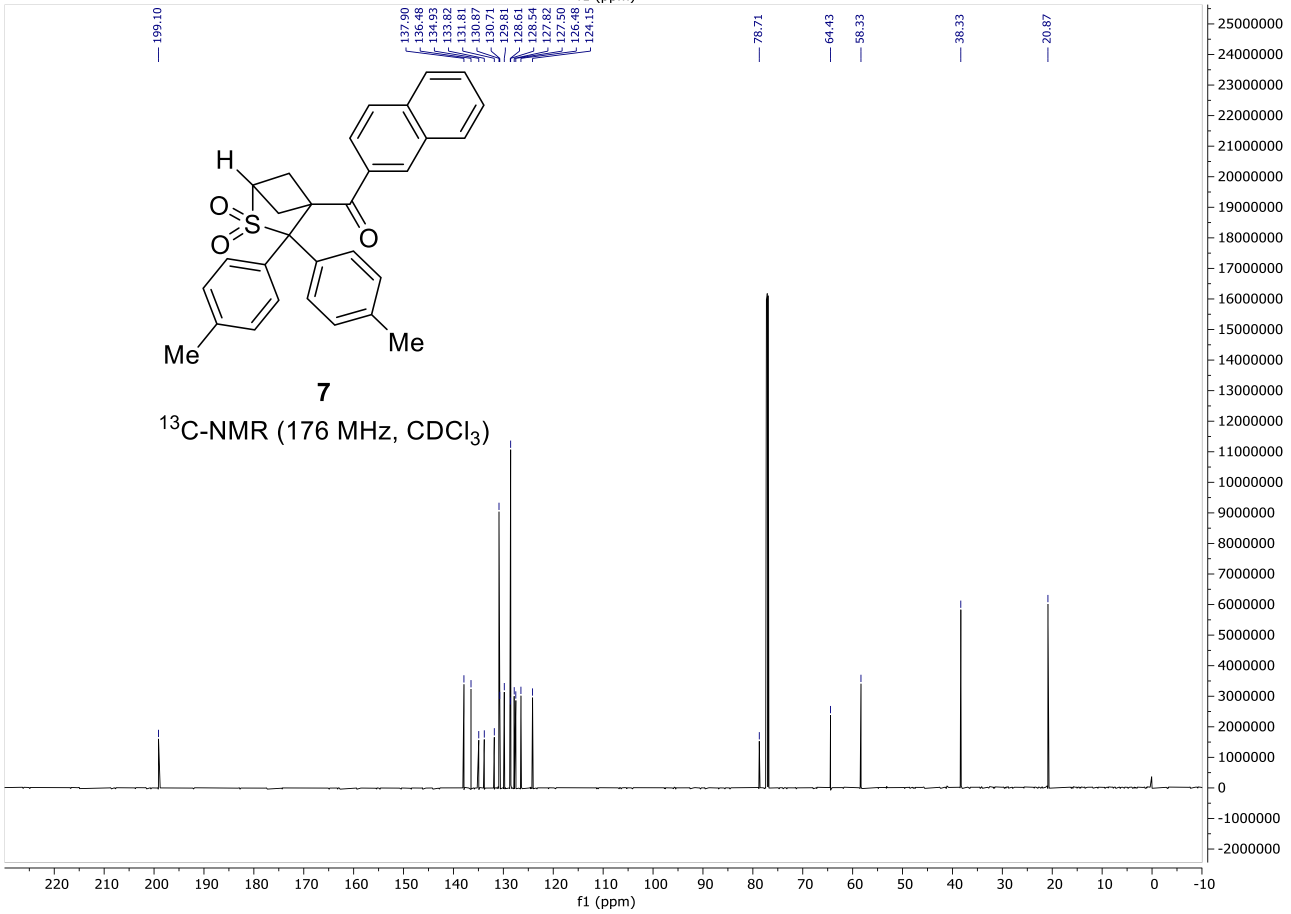

199.10
137.90 136.48 134.93 133.82 131.81 130.87 130.71 129.81 128.61 128.54 127.82 127.50 126.48 124.15
78.71
64.43
58.33
38.33
20.87
H
O
S
O
O
Me
Me
7
^{13}C-NMR (176 MHz, CDCl$_3$)
f1 (ppm)

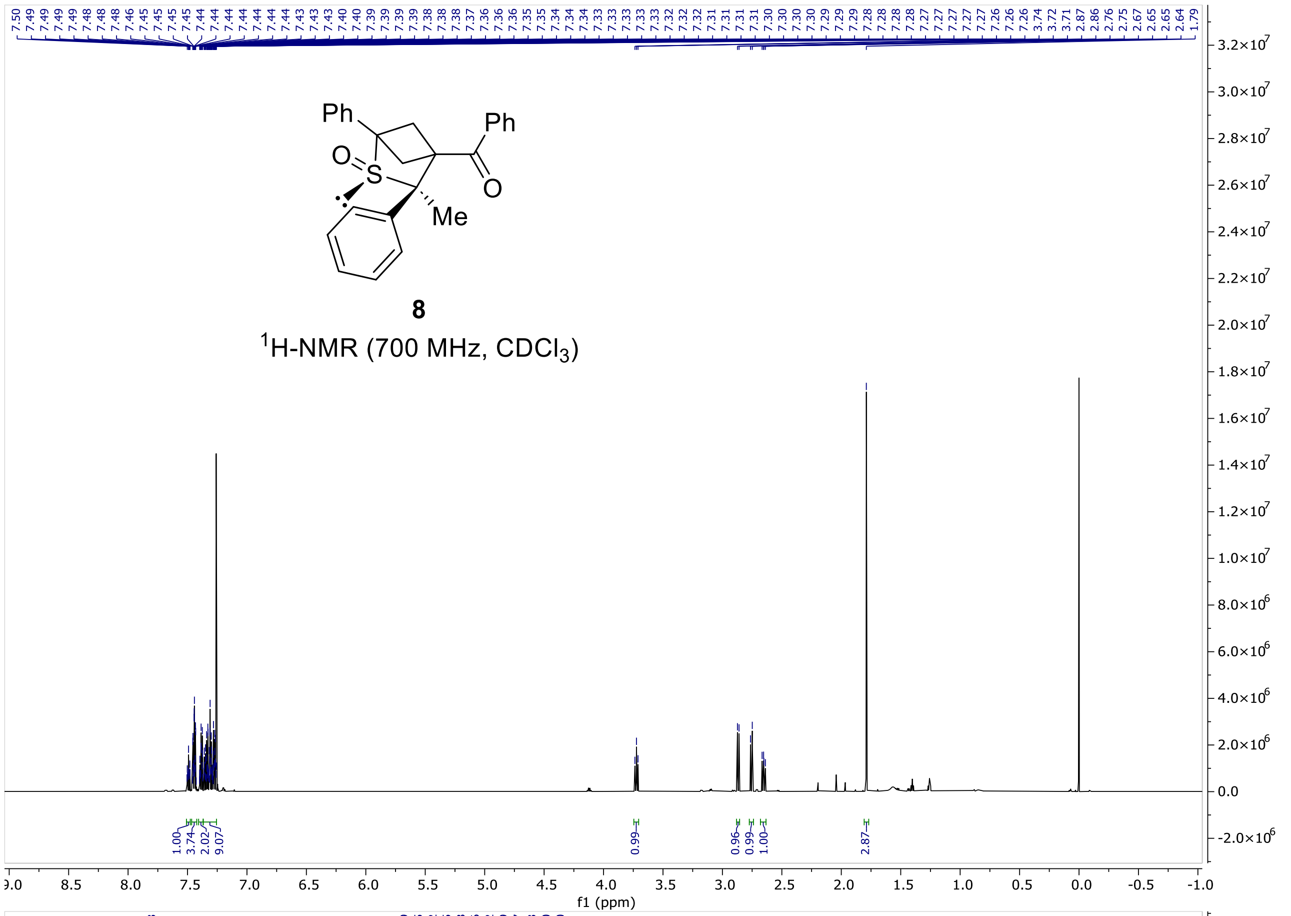

Ph
Ph
O
S
O
Me
8
^{1}H-NMR (700 MHz, $CDCl_3$)
f1 (ppm)

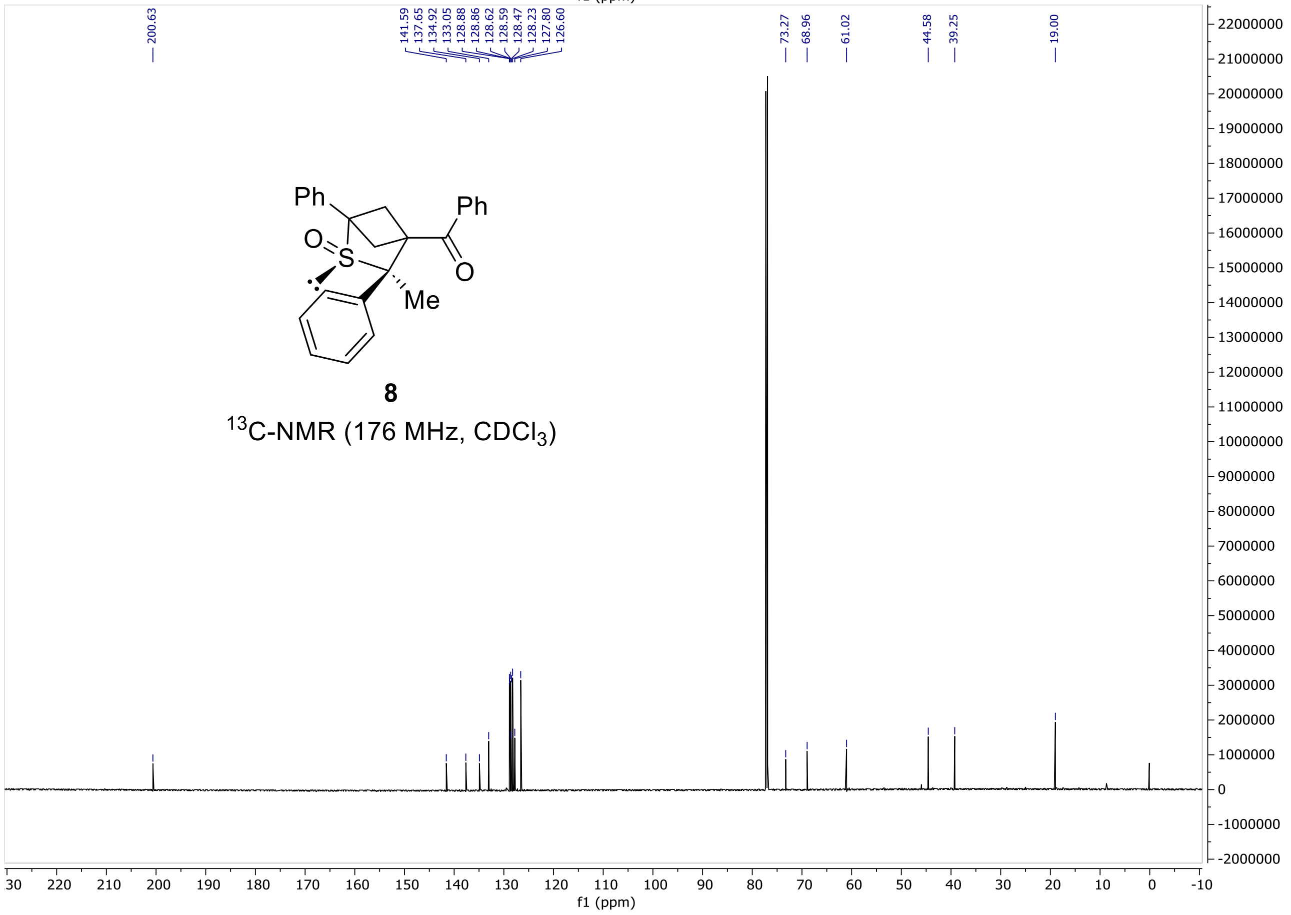

200.63
141.59
137.65
134.92
133.05
128.88
128.86
128.62
128.59
128.47
128.23
127.80
126.60
73.27
68.96
61.02
44.58
39.25
19.00
Ph
Ph
O
S
O
Me
8
^{13}C-NMR (176 MHz, $CDCl_3$)
f1 (ppm)

8. Crystal Structure Determinations

Structure Tables for compound 3aa

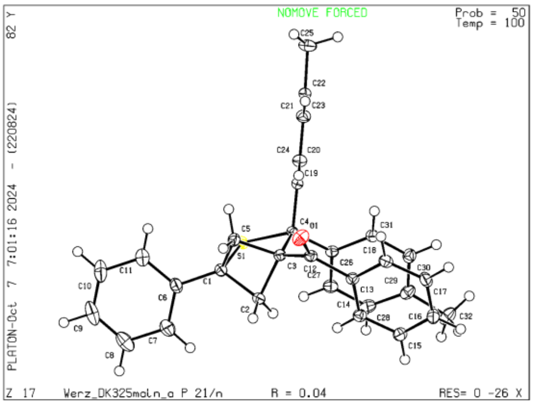

Crystals were obtained at room temperature by slow solvent evaporation from asolution of the compound dissolved in dichloromethane. A colourless, block-shaped crystal was mounted on a MiTeGen micromount with perfluoroether oil. Data for Werz_DK325main_a were collected from a shock-cooled single crystal at 100(2) K on a Bruker APEX2 QUAZAR three-circle diffractometer with a microfocus sealed X-ray tube using a mirror optics as monochromator and a Bruker APEXII detector. The diffractometer was equipped with an Oxford Cryostream 800 low temperature device and used MoK_α radiation (λ = 0.71073 Å). All data were integrated with SAINT V8.41 and a multi-scan absorption correction using SADABS 2016/2 was applied.[8,9] The structure was solved by direct methods with SHELXT and refined by full-matrix least-squares methods against F^2 using SHELXL-2019/2.[10,11] All non-hydrogen atoms were refined with anisotropic displacement parameters. All hydrogen atoms were refined isotropic on calculated positions using a riding model with their U_{iso} values constrained to 1.5 times the U_{eq} of their pivot atoms for terminal sp^3 carbon atoms and 1.2 times for all other carbon atoms. Crystallographic data for the structures reported in this paper have been deposited with the Cambridge Crystallographic Data Centre.[12] CCDC 2389052 contain the supplementary crystallographic data for this paper. These data can be obtained free of charge from The Cambridge Crystallographic Data Centre via www.ccdc.cam.ac.uk/structures. This report and the CIF file were generated using FinalCif.[13]

Table 1. Crystal data and structure refinement for Werz_DK325main_a

CCDC number	2389052
Empirical formula	$C_{32}H_{28}OS$
Formula weight	460.60
Temperature [K]	100(2)
Crystal system	monoclinic
Space group (number)	$P2_1/n$ (14)
a [Å]	11.698(3)
b [Å]	12.037(4)
c [Å]	17.473(5)
α [°]	90
β [°]	102.844(19)
γ [°]	90
Volume [Å^3]	2398.8(13)
Z	4
ρ_{calc} [gcm^{-3}]	1.275
μ [mm^{-1}]	0.158
F(000)	976
Crystal size [mm^3]	0.164×0.302×0.417
Crystal colour	colourless
Crystal shape	block
Radiation	MoK_α (λ=0.71073 Å)
2θ range [°]	3.83 to 61.16 (0.70 Å)
Index ranges	−16 ≤ h ≤ 16<br>−17 ≤ k ≤ 17<br>−24 ≤ l ≤ 24
Reflections collected	52228
Independent reflections	7347<br>R_{int} = 0.0400<br>R_{sigma} = 0.0245
Completeness to θ = 25.242°	100.0 %
Data / Restraints / Parameters	7347 / 0 / 309
Absorption correction T_{min}/T_{max} (method)	0.6890 / 0.7461 (multi-scan)
Goodness-of-fit on F^2	1.036
Final *R* indexes [$I \geq 2\sigma(I)$]	R_1 = 0.0429<br>wR_2 = 0.1124
Final *R* indexes [all data]	R_1 = 0.0518<br>wR_2 = 0.1197
Largest peak/hole [eÅ^{-3}]	0.66/−0.31

Table 2. Atomic coordinates and U_{eq} [Å^2] for Werz_DK325main_a

Atom	*x*	*y*	*z*	U_{eq}
S1	0.14739(3)	0.23378(2)	0.57163(2)	0.01651(8)
O1	0.52561(8)	0.24489(8)	0.49759(6)	0.0232(2)
C1	0.28790(11)	0.17961(10)	0.62914(7)	0.0171(2)
C2	0.32003(11)	0.09263(10)	0.57256(7)	0.0167(2)
H2A	0.254037	0.045090	0.545954	0.020
H2B	0.390990	0.048349	0.594703	0.020
C3	0.34230(10)	0.19744(10)	0.52434(7)	0.0145(2)
C4	0.21712(10)	0.24494(9)	0.48446(7)	0.0137(2)
C5	0.37839(11)	0.25769(10)	0.60484(7)	0.0168(2)
H5A	0.460521	0.244819	0.633096	0.020
H5B	0.357556	0.337489	0.603573	0.020
C6	0.28730(12)	0.15766(11)	0.71326(7)	0.0210(2)
C7	0.27446(13)	0.05092(13)	0.73997(8)	0.0266(3)
H7	0.263970	−0.009832	0.704389	0.032
C8	0.27697(14)	0.03286(15)	0.81946(9)	0.0340(3)
H8	0.270804	−0.040676	0.837874	0.041
C9	0.28823(14)	0.11995(17)	0.87095(9)	0.0359(4)
H9	0.285624	0.107240	0.924193	0.043
C10	0.3033(2)	0.22576(18)	0.84547(9)	0.0447(4)
H10	0.312798	0.286131	0.881349	0.054
C11	0.30463(19)	0.24455(15)	0.76699(9)	0.0393(4)
H11	0.317518	0.317544	0.750017	0.047
C12	0.43726(10)	0.18876(10)	0.47742(7)	0.0159(2)
C13	0.42650(10)	0.11290(10)	0.40864(7)	0.0155(2)
C14	0.36749(11)	0.01136(10)	0.40135(7)	0.0171(2)
H14	0.331282	−0.013482	0.441835	0.021
C15	0.36177(11)	−0.05360(11)	0.33448(7)	0.0192(2)
H15	0.322156	−0.123001	0.329784	0.023
C16	0.41362(12)	−0.01746(11)	0.27476(7)	0.0218(2)
H16	0.408510	−0.061503	0.229002	0.026
C17	0.47319(12)	0.08361(11)	0.28211(7)	0.0215(2)
H17	0.508539	0.108597	0.241247	0.026
C18	0.48091(11)	0.14761(10)	0.34884(7)	0.0186(2)
H18	0.523348	0.215506	0.354188	0.022
C19	0.21382(10)	0.36776(9)	0.46182(7)	0.0143(2)
C20	0.10390(10)	0.41916(10)	0.44200(7)	0.0151(2)
H20	0.035485	0.376617	0.441949	0.018
C21	0.09276(11)	0.53097(10)	0.42243(7)	0.0159(2)
H21	0.017156	0.564060	0.410159	0.019
C22	0.19144(11)	0.59547(10)	0.42056(7)	0.0170(2)
C23	0.30083(11)	0.54403(10)	0.43885(7)	0.0188(2)
H23	0.368989	0.586142	0.437369	0.023
C24	0.31227(11)	0.43148(10)	0.45937(7)	0.0174(2)
H24	0.387831	0.398313	0.471715	0.021
C25	0.18043(13)	0.71749(11)	0.40080(9)	0.0247(3)
H25A	0.170388	0.727239	0.344009	0.037
H25B	0.251475	0.756336	0.428087	0.037
H25C	0.112282	0.748218	0.417466	0.037
C26	0.15453(10)	0.17502(9)	0.41447(7)	0.0148(2)
C27	0.08627(11)	0.08211(10)	0.42150(8)	0.0188(2)
H27	0.068274	0.065586	0.470669	0.023
C28	0.04410(11)	0.01314(11)	0.35720(8)	0.0211(2)

H28	−0.001583	−0.050033	0.363562	0.025
C29	0.06752(11)	0.03493(11)	0.28401(8)	0.0208(2)
C30	0.13015(11)	0.13157(11)	0.27586(7)	0.0192(2)
H30	0.143989	0.150377	0.225894	0.023
C31	0.17240(10)	0.20042(10)	0.33960(7)	0.0164(2)
H31	0.214148	0.265888	0.332419	0.020
C32	0.03105(13)	−0.04517(13)	0.21657(9)	0.0287(3)
H32A	−0.006250	−0.004098	0.169159	0.043
H32B	−0.024592	−0.099184	0.229314	0.043
H32C	0.100298	−0.084243	0.207446	0.043

U_{eq} is defined as 1/3 of the trace of the orthogonalized U_{ij} tensor.

Table 3. Anisotropic displacement parameters [Å^2] for Werz_DK325main_a. The anisotropic displacement factor exponent takes the form: $-2\pi^2[\, h^2(a^*)^2U_{11} + k^2(b^*)^2U_{22} + \ldots + 2hka^*b^*U_{12}\,]$

Atom	U_{11}	U_{22}	U_{33}	U_{23}	U_{13}	U_{12}
S1	0.01808(14)	0.01680(14)	0.01606(14)	0.00243(10)	0.00678(10)	0.00185(10)
O1	0.0177(4)	0.0234(5)	0.0293(5)	−0.0064(4)	0.0068(4)	−0.0041(3)
C1	0.0202(5)	0.0166(5)	0.0145(5)	0.0013(4)	0.0041(4)	0.0027(4)
C2	0.0200(5)	0.0145(5)	0.0158(5)	0.0022(4)	0.0046(4)	0.0021(4)
C3	0.0154(5)	0.0130(5)	0.0154(5)	0.0000(4)	0.0037(4)	0.0007(4)
C4	0.0148(5)	0.0127(5)	0.0140(5)	0.0012(4)	0.0042(4)	−0.0005(4)
C5	0.0178(5)	0.0172(5)	0.0147(5)	−0.0014(4)	0.0021(4)	0.0009(4)
C6	0.0235(6)	0.0247(6)	0.0148(5)	0.0031(4)	0.0042(4)	0.0045(5)
C7	0.0281(7)	0.0296(7)	0.0237(6)	0.0047(5)	0.0094(5)	−0.0023(5)
C8	0.0305(7)	0.0431(9)	0.0301(7)	0.0140(6)	0.0107(6)	−0.0024(6)
C9	0.0282(7)	0.0628(11)	0.0159(6)	0.0077(6)	0.0033(5)	0.0005(7)
C10	0.0647(12)	0.0521(11)	0.0161(7)	−0.0037(7)	0.0065(7)	0.0043(9)
C11	0.0668(12)	0.0310(8)	0.0196(7)	−0.0004(6)	0.0087(7)	0.0026(8)
C12	0.0165(5)	0.0138(5)	0.0177(5)	0.0010(4)	0.0041(4)	0.0017(4)
C13	0.0146(5)	0.0146(5)	0.0169(5)	0.0004(4)	0.0029(4)	0.0023(4)
C14	0.0167(5)	0.0164(5)	0.0189(5)	0.0007(4)	0.0053(4)	0.0011(4)
C15	0.0205(6)	0.0172(5)	0.0196(5)	−0.0009(4)	0.0039(4)	0.0001(4)
C16	0.0260(6)	0.0236(6)	0.0156(5)	−0.0010(4)	0.0042(5)	0.0027(5)
C17	0.0253(6)	0.0244(6)	0.0157(5)	0.0041(4)	0.0065(5)	0.0016(5)
C18	0.0196(5)	0.0171(5)	0.0196(5)	0.0028(4)	0.0054(4)	0.0015(4)
C19	0.0174(5)	0.0126(5)	0.0134(5)	0.0003(4)	0.0042(4)	−0.0004(4)
C20	0.0162(5)	0.0151(5)	0.0145(5)	0.0003(4)	0.0044(4)	−0.0014(4)
C21	0.0178(5)	0.0161(5)	0.0137(5)	0.0009(4)	0.0035(4)	0.0021(4)
C22	0.0223(6)	0.0132(5)	0.0151(5)	0.0005(4)	0.0035(4)	−0.0014(4)
C23	0.0195(5)	0.0161(5)	0.0208(6)	0.0012(4)	0.0045(4)	−0.0037(4)
C24	0.0163(5)	0.0154(5)	0.0207(5)	0.0012(4)	0.0043(4)	−0.0001(4)
C25	0.0268(6)	0.0147(5)	0.0311(7)	0.0050(5)	0.0027(5)	−0.0005(5)
C26	0.0138(5)	0.0135(5)	0.0169(5)	0.0003(4)	0.0027(4)	0.0003(4)
C27	0.0176(5)	0.0172(5)	0.0220(6)	0.0010(4)	0.0052(4)	−0.0018(4)
C28	0.0184(5)	0.0165(5)	0.0276(6)	−0.0018(5)	0.0037(5)	−0.0038(4)
C29	0.0176(5)	0.0195(6)	0.0235(6)	−0.0055(5)	0.0007(5)	0.0008(4)
C30	0.0189(5)	0.0206(6)	0.0170(5)	−0.0015(4)	0.0017(4)	0.0021(4)
C31	0.0165(5)	0.0151(5)	0.0171(5)	0.0005(4)	0.0030(4)	−0.0003(4)
C32	0.0284(7)	0.0265(7)	0.0292(7)	−0.0118(6)	0.0022(6)	−0.0036(5)

Table 4. Bond lengths and angles for Werz_DK325main_a

Atom–Atom	Length [Å]
S1–C1	1.8445(13)
S1–C4	1.8856(12)
O1–C12	1.2195(15)
C1–C6	1.4948(17)
C1–C2	1.5428(17)
C1–C5	1.5440(18)
C2–C3	1.5706(17)
C2–H2A	0.9900
C2–H2B	0.9900
C3–C12	1.5237(17)
C3–C5	1.5552(17)

C3–C4	1.5822(17)
C4–C19	1.5288(17)
C4–C26	1.5303(16)
C5–H5A	0.9900
C5–H5B	0.9900
C6–C7	1.386(2)
C6–C11	1.390(2)
C7–C8	1.400(2)
C7–H7	0.9500
C8–C9	1.369(3)
C8–H8	0.9500
C9–C10	1.373(3)
C9–H9	0.9500
C10–C11	1.393(2)
C10–H10	0.9500
C11–H11	0.9500
C12–C13	1.4921(17)
C13–C14	1.3957(17)
C13–C18	1.4025(17)
C14–C15	1.3950(17)
C14–H14	0.9500
C15–C16	1.3883(18)
C15–H15	0.9500
C16–C17	1.394(2)
C16–H16	0.9500
C17–C18	1.3835(18)
C17–H17	0.9500
C18–H18	0.9500
C19–C24	1.3922(16)
C19–C20	1.3993(17)
C20–C21	1.3876(17)
C20–H20	0.9500
C21–C22	1.3977(17)
C21–H21	0.9500
C22–C23	1.3933(18)
C22–C25	1.5078(18)
C23–C24	1.4002(17)
C23–H23	0.9500
C24–H24	0.9500
C25–H25A	0.9800
C25–H25B	0.9800
C25–H25C	0.9800
C26–C27	1.3953(17)
C26–C31	1.4036(17)
C27–C28	1.3958(18)
C27–H27	0.9500
C28–C29	1.3910(19)
C28–H28	0.9500
C29–C30	1.3988(19)
C29–C32	1.5088(18)
C30–C31	1.3881(17)
C30–H30	0.9500
C31–H31	0.9500
C32–H32A	0.9800
C32–H32B	0.9800
C32–H32C	0.9800

**Atom–Atom–Atom**	**Angle [°]**
C1–S1–C4	88.40(6)
C6–C1–C2	124.41(11)
C6–C1–C5	122.10(11)
C2–C1–C5	87.55(9)
C6–C1–S1	113.56(9)
C2–C1–S1	101.85(8)
C5–C1–S1	102.56(8)
C1–C2–C3	83.79(9)
C1–C2–H2A	114.7
C3–C2–H2A	114.7
C1–C2–H2B	114.7
C3–C2–H2B	114.7
H2A–C2–H2B	111.8
C12–C3–C5	115.78(10)
C12–C3–C2	117.47(10)
C5–C3–C2	86.18(9)
C12–C3–C4	120.13(10)
C5–C3–C4	105.56(9)
C2–C3–C4	106.19(9)
C19–C4–C26	110.18(9)
C19–C4–C3	115.53(9)
C26–C4–C3	112.63(9)
C19–C4–S1	106.70(8)
C26–C4–S1	112.98(8)
C3–C4–S1	98.21(7)
C1–C5–C3	84.27(9)
C1–C5–H5A	114.6
C3–C5–H5A	114.6
C1–C5–H5B	114.6
C3–C5–H5B	114.6
H5A–C5–H5B	111.7
C7–C6–C11	118.71(13)
C7–C6–C1	121.29(12)
C11–C6–C1	119.95(13)
C6–C7–C8	119.88(15)
C6–C7–H7	120.1
C8–C7–H7	120.1
C9–C8–C7	120.73(15)
C9–C8–H8	119.6
C7–C8–H8	119.6
C8–C9–C10	119.85(14)
C8–C9–H9	120.1
C10–C9–H9	120.1
C9–C10–C11	119.99(17)
C9–C10–H10	120.0
C11–C10–H10	120.0
C6–C11–C10	120.71(16)
C6–C11–H11	119.6
C10–C11–H11	119.6
O1–C12–C13	119.09(11)
O1–C12–C3	118.61(11)
C13–C12–C3	122.29(10)
C14–C13–C18	119.34(11)
C14–C13–C12	124.12(11)
C18–C13–C12	116.54(11)
C15–C14–C13	119.87(11)
C15–C14–H14	120.1
C13–C14–H14	120.1

C16–C15–C14	120.42(12)
C16–C15–H15	119.8
C14–C15–H15	119.8
C15–C16–C17	119.82(12)
C15–C16–H16	120.1
C17–C16–H16	120.1
C18–C17–C16	120.09(12)
C18–C17–H17	120.0
C16–C17–H17	120.0
C17–C18–C13	120.43(12)
C17–C18–H18	119.8
C13–C18–H18	119.8
C24–C19–C20	118.02(11)
C24–C19–C4	124.56(11)
C20–C19–C4	117.42(10)
C21–C20–C19	121.38(11)
C21–C20–H20	119.3
C19–C20–H20	119.3
C20–C21–C22	120.78(11)
C20–C21–H21	119.6
C22–C21–H21	119.6
C23–C22–C21	117.97(11)
C23–C22–C25	120.85(11)
C21–C22–C25	121.17(12)
C22–C23–C24	121.29(11)
C22–C23–H23	119.4
C24–C23–H23	119.4
C19–C24–C23	120.55(11)
C19–C24–H24	119.7
C23–C24–H24	119.7
C22–C25–H25A	109.5
C22–C25–H25B	109.5
H25A–C25–H25B	109.5
C22–C25–H25C	109.5
H25A–C25–H25C	109.5
H25B–C25–H25C	109.5
C27–C26–C31	117.60(11)
C27–C26–C4	123.50(11)
C31–C26–C4	118.76(10)
C26–C27–C28	120.84(12)
C26–C27–H27	119.6
C28–C27–H27	119.6
C29–C28–C27	121.41(12)
C29–C28–H28	119.3
C27–C28–H28	119.3
C28–C29–C30	117.72(12)
C28–C29–C32	121.21(13)
C30–C29–C32	121.02(13)
C31–C30–C29	121.06(12)
C31–C30–H30	119.5
C29–C30–H30	119.5
C30–C31–C26	121.16(11)
C30–C31–H31	119.4
C26–C31–H31	119.4
C29–C32–H32A	109.5
C29–C32–H32B	109.5
H32A–C32–H32B	109.5
C29–C32–H32C	109.5
H32A–C32–H32C	109.5
H32B–C32–H32C	109.5

Table 5. Torsion angles for Werz_DK325main_a

Atom–Atom–Atom–Atom	Torsion Angle [°]
C4–S1–C1–C6	177.40(10)
C4–S1–C1–C2	−46.42(8)
C4–S1–C1–C5	43.68(8)
C6–C1–C2–C3	−159.49(12)
C5–C1–C2–C3	−31.52(8)
S1–C1–C2–C3	70.80(8)
C1–C2–C3–C12	148.47(11)
C1–C2–C3–C5	31.31(8)
C1–C2–C3–C4	−73.80(10)
C12–C3–C4–C19	−66.61(13)
C5–C3–C4–C19	66.52(12)
C2–C3–C4–C19	157.02(9)
C12–C3–C4–C26	61.22(13)
C5–C3–C4–C26	−165.66(9)
C2–C3–C4–C26	−75.15(11)
C12–C3–C4–S1	−179.61(9)
C5–C3–C4–S1	−46.48(9)
C2–C3–C4–S1	44.03(9)
C1–S1–C4–C19	−118.65(8)
C1–S1–C4–C26	120.13(9)
C1–S1–C4–C3	1.22(7)
C6–C1–C5–C3	161.69(11)
C2–C1–C5–C3	31.84(8)
S1–C1–C5–C3	−69.76(8)
C12–C3–C5–C1	−150.01(10)
C2–C3–C5–C1	−31.25(8)
C4–C3–C5–C1	74.50(10)
C2–C1–C6–C7	−23.5(2)
C5–C1–C6–C7	−135.11(14)
S1–C1–C6–C7	101.28(13)
C2–C1–C6–C11	153.85(15)
C5–C1–C6–C11	42.23(19)
S1–C1–C6–C11	−81.38(16)
C11–C6–C7–C8	1.2(2)
C1–C6–C7–C8	178.53(13)
C6–C7–C8–C9	2.1(2)
C7–C8–C9–C10	−3.4(3)
C8–C9–C10–C11	1.3(3)
C7–C6–C11–C10	−3.2(3)
C1–C6–C11–C10	179.42(17)
C9–C10–C11–C6	2.0(3)
C5–C3–C12–O1	−13.00(16)
C2–C3–C12–O1	−112.66(13)
C4–C3–C12–O1	115.66(13)
C5–C3–C12–C13	166.06(10)
C2–C3–C12–C13	66.40(15)

C4–C3–C12–C13	−65.28(15)
O1–C12–C13–C14	147.36(12)
C3–C12–C13–C14	−31.69(17)
O1–C12–C13–C18	−32.04(16)
C3–C12–C13–C18	148.90(11)
C18–C13–C14–C15	−0.91(17)
C12–C13–C14–C15	179.70(11)
C13–C14–C15–C16	−0.54(19)
C14–C15–C16–C17	0.9(2)
C15–C16–C17–C18	0.2(2)
C16–C17–C18–C13	−1.64(19)
C14–C13–C18–C17	2.00(18)
C12–C13–C18–C17	−178.56(11)
C26–C4–C19–C24	−115.66(13)
C3–C4–C19–C24	13.38(16)
S1–C4–C19–C24	121.35(11)
C26–C4–C19–C20	64.13(13)
C3–C4–C19–C20	−166.83(10)
S1–C4–C19–C20	−58.86(12)
C24–C19–C20–C21	−1.68(17)
C4–C19–C20–C21	178.52(10)
C19–C20–C21–C22	1.16(18)
C20–C21–C22–C23	0.04(17)
C20–C21–C22–C25	−178.87(12)

C21–C22–C23–C24	−0.69(18)
C25–C22–C23–C24	178.23(12)
C20–C19–C24–C23	1.02(18)
C4–C19–C24–C23	−179.19(11)
C22–C23–C24–C19	0.15(19)
C19–C4–C26–C27	−143.05(11)
C3–C4–C26–C27	86.36(14)
S1–C4–C26–C27	−23.81(14)
C19–C4–C26–C31	41.38(14)
C3–C4–C26–C31	−89.22(13)
S1–C4–C26–C31	160.61(9)
C31–C26–C27–C28	4.15(18)
C4–C26–C27–C28	−171.48(11)
C26–C27–C28–C29	−0.6(2)
C27–C28–C29–C30	−3.06(19)
C27–C28–C29–C32	174.48(12)
C28–C29–C30–C31	3.10(19)
C32–C29–C30–C31	−174.44(12)
C29–C30–C31–C26	0.50(19)
C27–C26–C31–C30	−4.11(18)
C4–C26–C31–C30	171.73(11)

Structure Tables for compound 3ae

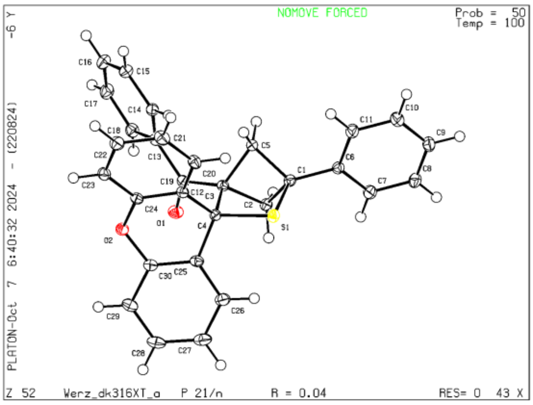

Crystals were obtained at room temperature by slow solvent evaporation from asolution of the compound dissolved in dichloromethane. A colourless, block-shaped crystal was mounted on a MiTeGen micromount with perfluoroether oil. Data for Werz_dk316XT_a were collected from a shock-cooled single crystal at 100(2) K on a Bruker D8 VENTURE dual wavelength Mo/Cu three-circle diffractometer with a microfocus sealed X-ray tube using a mirror optics as monochromator and a Bruker PHOTON III detector. The diffractometer was equipped with an Oxford Cryostream 800 low temperature device and used MoK_α radiation (λ = 0.71073 Å). All data were integrated with SAINT V8.41 and a multi-scan absorption correction using SADABS 2016/2 was applied.[8,9] The structure was solved by direct methods with SHELXT and refined by full-matrix least-squares methods against F^2 using SHELXL-2019/2.[10,11] All non-hydrogen atoms were refined with anisotropic displacement parameters. All hydrogen atoms were refined isotropic on calculated positions using a riding model with their U_{iso} values constrained to 1.5 times the U_{eq} of their pivot atoms for terminal sp^3 carbon atoms and 1.2 times for all other carbon atoms. Crystallographic data for the structures reported in this paper have been deposited with the Cambridge Crystallographic Data Centre.[12] CCDC 2389046 contain the supplementary crystallographic data for this paper. These data can be obtained free of charge from The Cambridge Crystallographic Data Centre via www.ccdc.cam.ac.uk/structures. This report and the CIF file were generated using FinalCif.[13]

Table 6. Crystal data and structure refinement for Werz_dk316XT_a

CCDC number	2389046
Empirical formula	$C_{30}H_{22}O_2S$
Formula weight	446.53
Temperature [K]	100(2)
Crystal system	monoclinic
Space group (number)	$P2_1/n$ (14)
a [Å]	9.9735(15)
b [Å]	6.9462(16)
c [Å]	31.359(7)
α [°]	90
β [°]	97.373(7)
γ [°]	90
Volume [Å^3]	2154.5(8)
Z	4
ρ_{calc} [gcm^{-3}]	1.377
μ [mm^{-1}]	0.177
F(000)	936
Crystal size [mm^3]	0.061×0.074×0.244
Crystal colour	colourless
Crystal shape	block
Radiation	MoK_α (λ=0.71073 Å)
2θ range [°]	2.62 to 61.11 (0.70 Å)
Index ranges	−14 ≤ h ≤ 14<br>−9 ≤ k ≤ 9<br>−44 ≤ l ≤ 44
Reflections collected	172409
Independent reflections	6590<br>R_{int} = 0.0623<br>R_{sigma} = 0.0177
Completeness to θ = 25.242°	99.9 %
Data / Restraints / Parameters	6590 / 0 / 298
Absorption correction T_{min}/T_{max} (method)	0.7182 / 0.7461 (multi-scan)
Goodness-of-fit on F^2	1.038
Final *R* indexes [I≥2σ(I)]	R_1 = 0.0370<br>wR_2 = 0.0908
Final *R* indexes [all data]	R_1 = 0.0455<br>wR_2 = 0.0972
Largest peak/hole [eÅ^{-3}]	0.44/−0.25

Table 7. Atomic coordinates and U_{eq} [Å^2] for Werz_dk316XT_a

Atom	*x*	*y*	*z*	U_{eq}
S1	0.73883(3)	0.12890(4)	0.43213(2)	0.01392(7)
O1	0.53365(9)	0.27833(13)	0.29282(3)	0.01790(17)
O2	0.45553(8)	−0.15424(12)	0.32151(3)	0.01550(16)
C1	0.69819(11)	0.38512(15)	0.41996(3)	0.01260(19)
C2	0.70380(11)	0.39350(16)	0.37104(3)	0.01346(19)
H2A	0.781269	0.324534	0.361310	0.016
H2B	0.692656	0.523930	0.358354	0.016
C3	0.57105(10)	0.27368(15)	0.36847(3)	0.01165(18)
C4	0.61738(10)	0.06376(15)	0.38439(3)	0.01171(18)
C5	0.54231(10)	0.38331(15)	0.40957(3)	0.01263(19)
H5A	0.500590	0.511781	0.404170	0.015
H5B	0.495096	0.306524	0.429685	0.015
C6	0.77039(11)	0.52063(16)	0.45195(3)	0.01369(19)
C7	0.89059(12)	0.60922(17)	0.44458(4)	0.0178(2)
H7	0.926546	0.586407	0.418412	0.021
C8	0.95821(12)	0.73105(17)	0.47540(4)	0.0194(2)
H8	1.040426	0.790328	0.470215	0.023
C9	0.90638(12)	0.76645(17)	0.51361(4)	0.0193(2)
H9	0.953209	0.848685	0.534699	0.023
C10	0.78531(13)	0.68064(19)	0.52083(4)	0.0210(2)
H10	0.748749	0.705541	0.546804	0.025
C11	0.71767(12)	0.55862(18)	0.49018(4)	0.0185(2)
H11	0.634948	0.500686	0.495304	0.022
C12	0.48038(11)	0.28574(15)	0.32570(3)	0.01271(19)
C13	0.33165(11)	0.31953(16)	0.32340(3)	0.01333(19)
C14	0.25813(11)	0.27389(16)	0.35702(3)	0.01399(19)
H14	0.302561	0.218218	0.382688	0.017
C15	0.11939(11)	0.30995(17)	0.35296(4)	0.0173(2)
H15	0.068969	0.275421	0.375529	0.021
C16	0.05501(12)	0.39643(19)	0.31588(4)	0.0200(2)
H16	−0.039169	0.422625	0.313332	0.024
C17	0.12813(12)	0.44476(19)	0.28247(4)	0.0199(2)
H17	0.084176	0.505435	0.257330	0.024
C18	0.26555(12)	0.40406(17)	0.28595(4)	0.0166(2)
H18	0.314857	0.433755	0.262813	0.020
C19	0.49750(11)	−0.05183(15)	0.39575(3)	0.01239(19)
C20	0.45474(12)	−0.05880(16)	0.43656(4)	0.0158(2)
H20	0.509516	−0.003013	0.460400	0.019
C21	0.33326(13)	−0.14622(18)	0.44267(4)	0.0193(2)
H21	0.305573	−0.149738	0.470542	0.023
C22	0.25222(12)	−0.22850(18)	0.40805(4)	0.0197(2)
H22	0.167867	−0.284363	0.412136	0.024
C23	0.29415(11)	−0.22927(16)	0.36758(4)	0.0163(2)
H23	0.239629	−0.286715	0.343905	0.020
C24	0.41719(11)	−0.14472(15)	0.36215(3)	0.01276(19)
C25	0.68046(11)	−0.04670(16)	0.35019(3)	0.01372(19)
C26	0.81858(12)	−0.05020(17)	0.34602(4)	0.0185(2)
H26	0.881145	0.012012	0.366970	0.022
C27	0.86528(13)	−0.14380(19)	0.31152(4)	0.0225(2)
H27	0.959018	−0.142137	0.308761	0.027
C28	0.77570(13)	−0.23970(18)	0.28107(4)	0.0211(2)
H28	0.808137	−0.302650	0.257524	0.025

C29	0.63897(12)	−0.24320(17)	0.28518(4)	0.0174(2)
H29	0.577206	−0.310820	0.264942	0.021
C30	0.59357(11)	−0.14602(16)	0.31944(4)	0.01405(19)

U_{eq} is defined as 1/3 of the trace of the orthogonalized U_{ij} tensor.

Table 8. Anisotropic displacement parameters [Å^2] for Werz_dk316XT_a. The anisotropic displacement factor exponent takes the form: $-2\pi^2[\,h^2(a^*)^2U_{11} + k^2(b^*)^2U_{22} + \ldots + 2hka^*b^*U_{12}\,]$

Atom	U_{11}	U_{22}	U_{33}	U_{23}	U_{13}	U_{12}
S1	0.01359(12)	0.01266(12)	0.01484(12)	0.00106(9)	−0.00072(9)	0.00006(9)
O1	0.0189(4)	0.0216(4)	0.0139(4)	0.0006(3)	0.0052(3)	0.0016(3)
O2	0.0151(4)	0.0199(4)	0.0120(3)	−0.0018(3)	0.0039(3)	−0.0010(3)
C1	0.0121(4)	0.0118(4)	0.0141(4)	0.0006(4)	0.0023(4)	−0.0007(3)
C2	0.0133(4)	0.0131(5)	0.0144(5)	0.0008(4)	0.0034(4)	−0.0016(4)
C3	0.0121(4)	0.0115(4)	0.0117(4)	0.0000(3)	0.0027(3)	0.0000(3)
C4	0.0122(4)	0.0110(4)	0.0119(4)	0.0000(3)	0.0015(3)	0.0001(3)
C5	0.0115(4)	0.0129(5)	0.0134(4)	−0.0013(4)	0.0012(3)	0.0003(4)
C6	0.0126(4)	0.0125(5)	0.0155(5)	0.0001(4)	0.0003(4)	0.0004(4)
C7	0.0165(5)	0.0177(5)	0.0196(5)	−0.0018(4)	0.0046(4)	−0.0032(4)
C8	0.0151(5)	0.0178(5)	0.0252(6)	−0.0022(4)	0.0023(4)	−0.0045(4)
C9	0.0185(5)	0.0163(5)	0.0221(5)	−0.0037(4)	−0.0015(4)	−0.0011(4)
C10	0.0209(5)	0.0244(6)	0.0178(5)	−0.0055(4)	0.0032(4)	−0.0019(5)
C11	0.0158(5)	0.0220(6)	0.0181(5)	−0.0027(4)	0.0035(4)	−0.0038(4)
C12	0.0143(5)	0.0106(4)	0.0133(4)	0.0004(4)	0.0021(4)	0.0000(4)
C13	0.0137(5)	0.0129(5)	0.0132(5)	−0.0017(4)	0.0011(4)	−0.0001(4)
C14	0.0140(5)	0.0137(5)	0.0142(5)	0.0000(4)	0.0015(4)	0.0001(4)
C15	0.0145(5)	0.0189(5)	0.0190(5)	−0.0007(4)	0.0037(4)	−0.0007(4)
C16	0.0138(5)	0.0227(6)	0.0228(6)	−0.0009(4)	−0.0010(4)	0.0007(4)
C17	0.0185(5)	0.0227(6)	0.0171(5)	0.0008(4)	−0.0035(4)	0.0013(4)
C18	0.0184(5)	0.0179(5)	0.0133(5)	0.0003(4)	0.0009(4)	0.0003(4)
C19	0.0136(4)	0.0099(4)	0.0140(5)	0.0005(4)	0.0035(4)	−0.0007(3)
C20	0.0196(5)	0.0145(5)	0.0139(5)	−0.0006(4)	0.0040(4)	−0.0021(4)
C21	0.0237(6)	0.0187(5)	0.0174(5)	−0.0002(4)	0.0099(4)	−0.0044(4)
C22	0.0185(5)	0.0184(5)	0.0239(6)	−0.0023(4)	0.0095(4)	−0.0052(4)
C23	0.0163(5)	0.0139(5)	0.0191(5)	−0.0024(4)	0.0042(4)	−0.0024(4)
C24	0.0149(5)	0.0112(4)	0.0126(4)	0.0004(4)	0.0037(4)	0.0007(4)
C25	0.0148(5)	0.0115(4)	0.0157(5)	0.0010(4)	0.0051(4)	0.0011(4)
C26	0.0156(5)	0.0166(5)	0.0243(6)	−0.0004(4)	0.0061(4)	0.0007(4)
C27	0.0191(5)	0.0205(6)	0.0303(6)	0.0010(5)	0.0121(5)	0.0032(4)
C28	0.0260(6)	0.0190(5)	0.0207(5)	0.0013(4)	0.0125(5)	0.0054(4)
C29	0.0231(5)	0.0152(5)	0.0149(5)	0.0007(4)	0.0061(4)	0.0028(4)
C30	0.0156(5)	0.0127(5)	0.0146(5)	0.0018(4)	0.0048(4)	0.0016(4)

Table 9. Bond lengths and angles for Werz_dk316XT_a

Atom–Atom	Length [Å]
S1–C1	1.8541(12)
S1–C4	1.8568(11)
O1–C12	1.2202(13)
O2–C24	1.3784(13)
O2–C30	1.3877(13)
C1–C6	1.4919(15)
C1–C2	1.5432(15)
C1–C5	1.5470(15)
C2–C3	1.5572(15)
C2–H2A	0.9900
C2–H2B	0.9900
C3–C12	1.5205(15)
C3–C5	1.5552(15)
C3–C4	1.5906(15)
C4–C25	1.5192(15)
C4–C19	1.5200(15)
C5–H5A	0.9900
C5–H5B	0.9900
C6–C7	1.3930(15)
C6–C11	1.3946(16)
C7–C8	1.3920(16)
C7–H7	0.9500
C8–C9	1.3866(17)
C8–H8	0.9500
C9–C10	1.3910(17)
C9–H9	0.9500
C10–C11	1.3895(17)
C10–H10	0.9500
C11–H11	0.9500

C12–C13	1.4944(15)
C13–C14	1.3955(15)
C13–C18	1.4000(15)
C14–C15	1.3958(15)
C14–H14	0.9500
C15–C16	1.3907(17)
C15–H15	0.9500
C16–C17	1.3922(18)
C16–H16	0.9500
C17–C18	1.3899(16)
C17–H17	0.9500
C18–H18	0.9500
C19–C24	1.3969(15)
C19–C20	1.4009(15)
C20–C21	1.3903(16)
C20–H20	0.9500
C21–C22	1.3906(17)
C21–H21	0.9500
C22–C23	1.3861(16)
C22–H22	0.9500
C23–C24	1.3904(15)
C23–H23	0.9500
C25–C30	1.3936(16)
C25–C26	1.4006(15)
C26–C27	1.3926(17)
C26–H26	0.9500
C27–C28	1.3909(19)
C27–H27	0.9500
C28–C29	1.3863(17)
C28–H28	0.9500
C29–C30	1.3929(15)
C29–H29	0.9500
**Atom–Atom–Atom**	**Angle [°]**
C1–S1–C4	88.00(5)
C24–O2–C30	115.84(9)
C6–C1–C2	124.67(9)
C6–C1–C5	122.14(9)
C2–C1–C5	87.41(8)
C6–C1–S1	113.15(8)
C2–C1–S1	101.89(7)
C5–C1–S1	102.87(7)
C1–C2–C3	83.82(7)
C1–C2–H2A	114.7
C3–C2–H2A	114.7
C1–C2–H2B	114.7
C3–C2–H2B	114.7
H2A–C2–H2B	111.8
C12–C3–C5	123.51(9)
C12–C3–C2	114.95(9)
C5–C3–C2	86.63(8)
C12–C3–C4	116.32(8)
C5–C3–C4	105.40(8)
C2–C3–C4	105.24(8)
C25–C4–C19	107.94(9)
C25–C4–C3	111.80(8)
C19–C4–C3	110.63(8)
C25–C4–S1	113.95(7)

C19–C4–S1	113.03(7)
C3–C4–S1	99.38(7)
C1–C5–C3	83.76(7)
C1–C5–H5A	114.7
C3–C5–H5A	114.7
C1–C5–H5B	114.7
C3–C5–H5B	114.7
H5A–C5–H5B	111.8
C7–C6–C11	119.11(10)
C7–C6–C1	121.22(10)
C11–C6–C1	119.66(10)
C8–C7–C6	120.26(11)
C8–C7–H7	119.9
C6–C7–H7	119.9
C9–C8–C7	120.44(11)
C9–C8–H8	119.8
C7–C8–H8	119.8
C8–C9–C10	119.50(11)
C8–C9–H9	120.3
C10–C9–H9	120.3
C11–C10–C9	120.22(11)
C11–C10–H10	119.9
C9–C10–H10	119.9
C10–C11–C6	120.46(11)
C10–C11–H11	119.8
C6–C11–H11	119.8
O1–C12–C13	120.20(10)
O1–C12–C3	117.96(10)
C13–C12–C3	121.70(9)
C14–C13–C18	119.50(10)
C14–C13–C12	122.85(10)
C18–C13–C12	117.64(10)
C13–C14–C15	120.09(10)
C13–C14–H14	120.0
C15–C14–H14	120.0
C16–C15–C14	119.97(11)
C16–C15–H15	120.0
C14–C15–H15	120.0
C15–C16–C17	120.21(11)
C15–C16–H16	119.9
C17–C16–H16	119.9
C18–C17–C16	119.88(11)
C18–C17–H17	120.1
C16–C17–H17	120.1
C17–C18–C13	120.31(11)
C17–C18–H18	119.8
C13–C18–H18	119.8
C24–C19–C20	117.44(10)
C24–C19–C4	117.35(9)
C20–C19–C4	124.97(10)
C21–C20–C19	120.92(10)
C21–C20–H20	119.5
C19–C20–H20	119.5
C20–C21–C22	120.09(11)
C20–C21–H21	120.0
C22–C21–H21	120.0
C23–C22–C21	120.20(11)
C23–C22–H22	119.9

C21–C22–H22	119.9
C22–C23–C24	119.02(11)
C22–C23–H23	120.5
C24–C23–H23	120.5
O2–C24–C23	116.54(10)
O2–C24–C19	121.28(10)
C23–C24–C19	122.17(10)
C30–C25–C26	117.29(10)
C30–C25–C4	117.48(9)
C26–C25–C4	125.17(10)
C27–C26–C25	120.77(11)
C27–C26–H26	119.6
C25–C26–H26	119.6
C28–C27–C26	120.50(11)
C28–C27–H27	119.8
C26–C27–H27	119.8
C29–C28–C27	119.83(11)
C29–C28–H28	120.1
C27–C28–H28	120.1
C28–C29–C30	118.96(11)
C28–C29–H29	120.5
C30–C29–H29	120.5
O2–C30–C29	116.02(10)
O2–C30–C25	121.37(10)
C29–C30–C25	122.60(10)

Table 10. Torsion angles for Werz_dk316XT_a

Atom–Atom–Atom–Atom	Torsion Angle [°]
C4–S1–C1–C6	177.97(8)
C4–S1–C1–C2	−45.79(7)
C4–S1–C1–C5	44.25(7)
C6–C1–C2–C3	−159.74(10)
C5–C1–C2–C3	−31.70(7)
S1–C1–C2–C3	70.91(7)
C1–C2–C3–C12	157.18(9)
C1–C2–C3–C5	31.54(7)
C1–C2–C3–C4	−73.51(8)
C12–C3–C4–C25	52.54(12)
C5–C3–C4–C25	−166.64(8)
C2–C3–C4–C25	−75.95(10)
C12–C3–C4–C19	−67.79(11)
C5–C3–C4–C19	73.03(10)
C2–C3–C4–C19	163.71(8)
C12–C3–C4–S1	173.14(7)
C5–C3–C4–S1	−46.03(8)
C2–C3–C4–S1	44.65(8)
C1–S1–C4–C25	119.73(8)
C1–S1–C4–C19	−116.55(8)
C1–S1–C4–C3	0.71(7)
C6–C1–C5–C3	161.84(10)
C2–C1–C5–C3	31.75(7)
S1–C1–C5–C3	−69.85(7)
C12–C3–C5–C1	−149.35(10)
C2–C3–C5–C1	−31.46(7)
C4–C3–C5–C1	73.43(8)
C2–C1–C6–C7	−28.46(16)
C5–C1–C6–C7	−140.15(11)
S1–C1–C6–C7	96.16(11)
C2–C1–C6–C11	152.09(11)
C5–C1–C6–C11	40.40(15)
S1–C1–C6–C11	−83.29(12)
C11–C6–C7–C8	1.23(18)
C1–C6–C7–C8	−178.23(11)
C6–C7–C8–C9	−0.39(19)
C7–C8–C9–C10	−0.60(19)
C8–C9–C10–C11	0.74(19)
C9–C10–C11–C6	0.11(19)
C7–C6–C11–C10	−1.09(18)
C1–C6–C11–C10	178.37(11)
C5–C3–C12–O1	145.77(11)
C2–C3–C12–O1	42.45(14)
C4–C3–C12–O1	−81.16(12)
C5–C3–C12–C13	−29.99(15)
C2–C3–C12–C13	−133.30(10)
C4–C3–C12–C13	103.09(11)
O1–C12–C13–C14	160.36(11)
C3–C12–C13–C14	−23.98(16)
O1–C12–C13–C18	−20.87(16)
C3–C12–C13–C18	154.79(10)
C18–C13–C14–C15	0.91(17)
C12–C13–C14–C15	179.66(10)
C13–C14–C15–C16	−1.82(17)
C14–C15–C16–C17	0.93(19)
C15–C16–C17–C18	0.87(19)
C16–C17–C18–C13	−1.78(18)
C14–C13–C18–C17	0.89(17)
C12–C13–C18–C17	−177.93(11)
C25–C4–C19–C24	−38.86(13)
C3–C4–C19–C24	83.75(11)
S1–C4–C19–C24	−165.82(8)
C25–C4–C19–C20	146.91(11)
C3–C4–C19–C20	−90.48(12)
S1–C4–C19–C20	19.96(14)
C24–C19–C20–C21	−3.34(17)
C4–C19–C20–C21	170.88(11)
C19–C20–C21–C22	0.04(18)
C20–C21–C22–C23	2.08(19)
C21–C22–C23–C24	−0.77(18)
C30–O2–C24–C23	−154.18(10)
C30–O2–C24–C19	26.84(14)
C22–C23–C24–O2	178.30(10)
C22–C23–C24–C19	−2.73(17)
C20–C19–C24–O2	−176.34(10)
C4–C19–C24–O2	8.99(15)
C20–C19–C24–C23	4.73(16)
C4–C19–C24–C23	−169.94(10)
C19–C4–C25–C30	36.38(13)
C3–C4–C25–C30	−85.51(12)

S1–C4–C25–C30	162.80(8)
C19–C4–C25–C26	−146.43(11)
C3–C4–C25–C26	91.68(13)
S1–C4–C25–C26	−20.01(14)
C30–C25–C26–C27	2.26(17)
C4–C25–C26–C27	−174.93(11)
C25–C26–C27–C28	−1.57(19)
C26–C27–C28–C29	−0.36(19)
C27–C28–C29–C30	1.48(18)

C24–O2–C30–C29	150.15(10)
C24–O2–C30–C25	−29.52(15)
C28–C29–C30–O2	179.60(10)
C28–C29–C30–C25	−0.73(17)
C26–C25–C30–O2	178.53(10)
C4–C25–C30–O2	−4.06(15)
C26–C25–C30–C29	−1.13(16)
C4–C25–C30–C29	176.29(10)

Structure Tables for compound 3ra

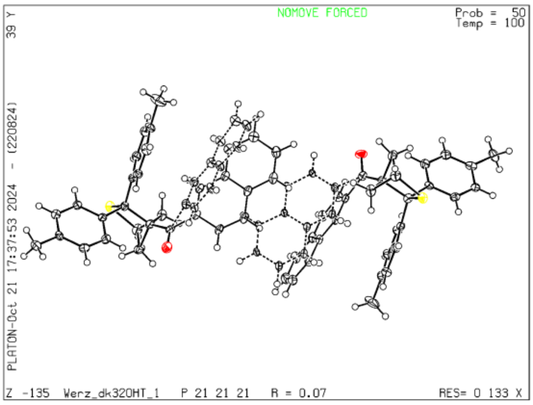

Crystals were obtained at room temperature by slow solvent evaporation from asolution of the compound dissolved in dichloromethane. A colourless, plate-shaped crystal was mounted on a MiTeGen micromount with perfluoroether oil. Data for Werz_dk320HT_1 were collected from a shock-cooled single crystal at 100(2) K on a Bruker APEX2 QUAZAR three-circle diffractometer with a microfocus sealed X-ray tube using a mirror optics as monochromator and a Bruker APEXII detector. The diffractometer was equipped with an Oxford Cryostream 800 low temperature device and used MoK_α radiation (λ = 0.71073 Å). All data were integrated with SAINT V8.41 and a multi-scan absorption correction using SADABS 2016/2 was applied.[8,9] The structure was solved by direct methods with SHELXT and refined by full-matrix least-squares methods against F^2 using SHELXL-2019/2.[10,11] All non-hydrogen atoms were refined with anisotropic displacement parameters. All hydrogen atoms were refined isotropic on calculated positions using a riding model with their U_{iso} values constrained to 1.5 times the U_{eq} of their pivot atoms for terminal sp^3 carbon atoms and 1.2 times for all other carbon atoms. Disordered moieties were refined using bond lengths restraints and displacement parameter restraints. Some parts of the disorder model were introduced by the program DSR[12]. Crystallographic data for the structures reported in this paper have been deposited with the Cambridge Crystallographic Data Centre.[6] CCDC 2392683 contain the supplementary crystallographic data for this paper. These data can be obtained free of charge from The Cambridge Crystallographic Data Centre via www.ccdc.cam.ac.uk/structures. This report and the CIF file were generated using FinalCif.[13]

Table 11. Crystal data and structure refinement for Werz_dk320HT_1

CCDC number	2392683
Empirical formula	$C_{30}H_{26}OS$
Formula weight	434.57
Temperature [K]	100(2)
Crystal system	orthorhombic
Space group (number)	$P2_12_12_1$ (19)
a [Å]	7.683(5)
b [Å]	15.401(8)
c [Å]	37.98(2)
α [°]	90
β [°]	90
γ [°]	90
Volume [Å^3]	4495(4)
Z	8
ρ_{calc} [gcm^{-3}]	1.284
μ [mm^{-1}]	0.165
F(000)	1840
Crystal size [mm^3]	0.036×0.138×0.289
Crystal colour	colourless
Crystal shape	plate
Radiation	MoK_α (λ=0.71073 Å)
2θ range [°]	2.14 to 58.31 (0.73 Å)
Index ranges	−10 ≤ h ≤ 10<br>−21 ≤ k ≤ 21<br>−51 ≤ l ≤ 51
Reflections collected	152829
Independent reflections	12109<br>R_{int} = 0.1031<br>R_{sigma} = 0.0522
Completeness to θ = 25.242°	99.9 %
Data / Restraints / Parameters	12109 / 2618 / 763
Absorption correction T_{min}/T_{max} (method)	0.6829 / 0.7458 (multi-scan)
Goodness-of-fit on F^2	1.057
Final *R* indexes [$I≥2σ(I)$]	R_1 = 0.0700<br>wR_2 = 0.1693
Final *R* indexes [all data]	R_1 = 0.0891<br>wR_2 = 0.1791
Largest peak/hole [eÅ^{-3}]	0.95/−0.46
Flack X parameter	0.42(15)

Refinement details for Werz_dk320HT_1

Refined as a 2-component inversion twin.

Table 12. Atomic coordinates and U_{eq} [Å^2] for Werz_dk320HT_1

Atom	*x*	*y*	*z*	U_{eq}
S1	0.79457(17)	0.51583(8)	0.70981(3)	0.0225(3)
S2	0.19050(17)	0.47840(8)	0.28925(3)	0.0225(3)
O1	0.7259(6)	0.3111(3)	0.61257(11)	0.0342(10)
O2	0.3121(7)	0.6771(3)	0.38653(10)	0.0388(11)
C1	0.5785(7)	0.4871(4)	0.69335(13)	0.0257(11)
H1	0.473638	0.503306	0.707496	0.031
C2	0.5896(7)	0.5157(4)	0.65471(13)	0.0240(10)
H2A	0.647603	0.572313	0.650900	0.029
H2B	0.478921	0.511395	0.641489	0.029
C3	0.7117(7)	0.4349(3)	0.65109(13)	0.0219(10)
C4	0.8874(6)	0.4597(3)	0.66975(12)	0.0181(9)
C5	0.5977(7)	0.3936(4)	0.68032(14)	0.0269(12)
H5A	0.488395	0.366774	0.671801	0.032
H5B	0.661278	0.354612	0.696576	0.032
C6	0.7133(6)	0.3902(3)	0.61515(13)	0.0191(9)
C7	0.9990(6)	0.3855(3)	0.68428(12)	0.0172(10)
C8	0.9804(7)	0.2985(3)	0.67471(14)	0.0243(11)
H8	0.892669	0.282125	0.658376	0.029
C9	1.0912(8)	0.2347(3)	0.68920(14)	0.0263(11)
H9	1.079171	0.175932	0.682030	0.032
C10	1.2172(7)	0.2566(3)	0.71371(13)	0.0192(10)
C11	1.2346(6)	0.3432(3)	0.72346(14)	0.0207(10)
H11	1.319363	0.359229	0.740464	0.025
C12	1.1290(6)	0.4062(3)	0.70853(13)	0.0196(9)
H12	1.145580	0.465258	0.714987	0.023
C13	1.3331(8)	0.1885(4)	0.72994(14)	0.0288(12)
H13A	1.452992	0.210167	0.730829	0.043
H13B	1.328864	0.135551	0.715685	0.043
H13C	1.292871	0.175677	0.753861	0.043
C14	0.9986(6)	0.5192(3)	0.64693(12)	0.0166(9)
C15	0.9902(7)	0.6100(3)	0.64841(14)	0.0217(10)
H15	0.917147	0.637063	0.665340	0.026
C16	1.0878(7)	0.6613(4)	0.62537(13)	0.0248(11)
H16	1.080814	0.722754	0.627039	0.030
C17	1.1940(7)	0.6243(3)	0.60024(14)	0.0250(11)
C18	1.2001(7)	0.5324(4)	0.59811(14)	0.0252(11)
H18	1.271287	0.505399	0.580849	0.030
C19	1.1032(6)	0.4817(3)	0.62095(12)	0.0208(10)
H19	1.107946	0.420300	0.618922	0.025
C20	1.3037(10)	0.6793(4)	0.5765(2)	0.0457(17)
H20A	1.292678	0.658494	0.552209	0.069
H20B	1.425690	0.675649	0.583876	0.069
H20C	1.264563	0.739785	0.577830	0.069
C21	0.4073(7)	0.5037(4)	0.30518(14)	0.0274(11)
H21	0.510574	0.486356	0.290778	0.033
C22	0.3955(7)	0.4738(4)	0.34401(13)	0.0264(11)
H22A	0.506873	0.475743	0.357093	0.032
H22B	0.333811	0.418020	0.347609	0.032
C23	0.2771(6)	0.5578(3)	0.34826(13)	0.0192(10)

C24	0.0995(6)	0.5348(3)	0.32916(12)	0.0183(9)
C25	0.3940(7)	0.5984(4)	0.31896(13)	0.0259(11)
H25A	0.332596	0.638875	0.302963	0.031
H25B	0.505028	0.623198	0.327530	0.031
C26	0.2713(7)	0.6017(4)	0.38380(14)	0.0232(11)
C27	−0.0140(6)	0.4765(4)	0.35200(12)	0.0198(10)
C28	−0.0051(7)	0.3870(3)	0.35191(13)	0.0223(10)
H28	0.067482	0.358324	0.335324	0.027
C29	−0.1017(7)	0.3380(4)	0.37594(14)	0.0267(11)
H29	−0.092436	0.276497	0.375380	0.032
C30	−0.2108(7)	0.3763(4)	0.40062(15)	0.0293(12)
C31	−0.2227(7)	0.4664(4)	0.40014(14)	0.0253(11)
H31	−0.295904	0.494867	0.416678	0.030
C32	−0.1288(7)	0.5161(4)	0.37575(12)	0.0219(10)
H32	−0.143217	0.577341	0.375307	0.026
C33	−0.3127(9)	0.3232(5)	0.42628(19)	0.0491(19)
H33A	−0.375745	0.361784	0.442346	0.074
H33B	−0.233237	0.286259	0.439817	0.074
H33C	−0.395968	0.286672	0.413521	0.074
C34	−0.0043(6)	0.6125(3)	0.31570(12)	0.0182(10)
C35	−0.1360(6)	0.5947(3)	0.29091(13)	0.0203(10)
H35	−0.154095	0.536631	0.283367	0.024
C36	−0.2394(6)	0.6602(3)	0.27733(14)	0.0204(10)
H36	−0.325952	0.646321	0.260387	0.024
C37	−0.2188(7)	0.7466(3)	0.28807(16)	0.0239(11)
C38	−0.0906(8)	0.7634(4)	0.31250(14)	0.0267(11)
H38	−0.074840	0.821355	0.320492	0.032
C39	0.0170(8)	0.6981(3)	0.32590(14)	0.0251(11)
H39	0.105978	0.712642	0.342239	0.030
C40	−0.3311(9)	0.8163(4)	0.27225(15)	0.0314(13)
H40A	−0.292036	0.873245	0.280659	0.047
H40B	−0.452425	0.807044	0.279240	0.047
H40C	−0.321943	0.814175	0.246528	0.047
C1_1	0.2294(6)	0.4665(4)	0.42170(14)	0.0195(10)
H1_1	0.283764	0.431571	0.404263	0.023
C2_1	0.2090(6)	0.5545(3)	0.41593(12)	0.0202(9)
C3_1	0.1269(7)	0.6067(4)	0.44238(13)	0.0223(9)
H3_1	0.113344	0.667350	0.438603	0.027
C4_1	0.0681(7)	0.5706(3)	0.47282(13)	0.0233(10)
H4_1	0.012150	0.605988	0.489889	0.028
C4A_1	0.0895(7)	0.4804(3)	0.47934(12)	0.0201(8)
C5_1	0.0330(8)	0.4408(4)	0.51099(14)	0.0252(10)
H5_1	−0.019554	0.475425	0.528746	0.030
C6_1	0.0529(8)	0.3534(4)	0.51648(15)	0.0283(11)
H6_1	0.010837	0.327882	0.537595	0.034
C7_1	0.1353(8)	0.3014(4)	0.49098(14)	0.0255(10)
H7_1	0.152309	0.241209	0.495259	0.031
C8_1	0.1917(8)	0.3379(3)	0.45958(13)	0.0228(10)
H8_1	0.245157	0.302478	0.442212	0.027
C8A_1	0.1699(7)	0.4276(3)	0.45339(12)	0.0183(8)
C1_2	0.7216(7)	0.5259(3)	0.57728(13)	0.0185(9)
H1_2	0.785465	0.555562	0.595004	0.022
C2_2	0.6708(6)	0.4406(3)	0.58275(12)	0.0180(9)
C3_2	0.5748(7)	0.3973(3)	0.55609(13)	0.0202(9)
H3_2	0.542111	0.338381	0.559438	0.024

C4_2	0.5279(7)	0.4392(3)	0.52538(13)	0.0221(9)
H4_2	0.460086	0.409740	0.508209	0.027
C4A_2	0.5807(6)	0.5254(3)	0.51953(12)	0.0198(8)
C5_2	0.5378(8)	0.5708(4)	0.48808(14)	0.0244(10)
H5_2	0.472196	0.542111	0.470358	0.029
C6_2	0.5886(7)	0.6543(4)	0.48285(14)	0.0257(10)
H6_2	0.555456	0.683677	0.461911	0.031
C7_2	0.6908(8)	0.6979(4)	0.50846(14)	0.0252(10)
H7_2	0.728524	0.755767	0.504443	0.030
C8_2	0.7351(8)	0.6557(3)	0.53922(14)	0.0217(10)
H8_2	0.803882	0.684772	0.556327	0.026
C8A_2	0.6792(7)	0.5694(3)	0.54559(12)	0.0184(8)
C1_3	0.276(5)	0.473(2)	0.4220(8)	0.018(3)
H1_3	0.213280	0.443963	0.403836	0.021
C2_3	0.326(5)	0.559(2)	0.4175(8)	0.018(3)
C3_3	0.419(5)	0.602(2)	0.4444(8)	0.015(5)
H3_3	0.453598	0.660615	0.441430	0.018
C4_3	0.459(6)	0.5573(18)	0.4756(8)	0.016(5)
H4_3	0.519794	0.586415	0.493832	0.020
C4A_3	0.409(5)	0.4702(17)	0.4801(7)	0.015(4)
C5_3	0.456(6)	0.426(2)	0.5114(8)	0.016(5)
H5_3	0.525423	0.454217	0.528781	0.019
C6_3	0.397(5)	0.341(2)	0.5162(8)	0.015(5)
H6_3	0.418139	0.311980	0.537939	0.018
C7_3	0.308(6)	0.298(2)	0.4891(9)	0.019(4)
H7_3	0.274463	0.238836	0.492147	0.023
C8_3	0.269(6)	0.340(2)	0.4576(9)	0.019(3)
H8_3	0.209112	0.310482	0.439288	0.023
C8A_3	0.318(6)	0.4274(18)	0.4530(8)	0.018(3)
C1_4	0.781(5)	0.532(2)	0.5785(8)	0.019(3)
H1_4	0.735705	0.567580	0.596651	0.023
C2_4	0.794(4)	0.442(2)	0.5832(8)	0.020(3)
C3_4	0.860(5)	0.388(2)	0.5566(8)	0.019(4)
H3_4	0.865033	0.326821	0.560043	0.023
C4_4	0.918(6)	0.4246(18)	0.5250(9)	0.020(4)
H4_4	0.966022	0.388575	0.507189	0.025
C4A_4	0.905(6)	0.5150(18)	0.5196(8)	0.020(4)
C5_4	0.955(6)	0.553(2)	0.4875(8)	0.022(5)
H5_4	1.001894	0.518763	0.469171	0.026
C6_4	0.933(6)	0.643(2)	0.4827(9)	0.021(5)
H6_4	0.950325	0.667556	0.460020	0.026
C7_4	0.888(6)	0.697(2)	0.5110(9)	0.024(4)
H7_4	0.902874	0.758574	0.509480	0.028
C8_4	0.819(7)	0.6587(19)	0.5416(10)	0.022(3)
H8_4	0.760764	0.693007	0.558712	0.027
C8A_4	0.838(6)	0.5684(18)	0.5464(8)	0.020(3)

U_{eq} is defined as 1/3 of the trace of the orthogonalized U_{ij} tensor.

Table 13. Anisotropic displacement parameters [Å^2] for Werz_dk320HT_1. The anisotropic displacement factor exponent takes the form: $-2\pi^2[\, h^2(a^*)^2U_{11} + k^2(b^*)^2U_{22} + \ldots + 2hka^*b^*U_{12}\,]$

Atom	U_{11}	U_{22}	U_{33}	U_{23}	U_{13}	U_{12}
S1	0.0232(6)	0.0286(6)	0.0157(5)	−0.0030(5)	0.0028(5)	0.0039(5)
S2	0.0229(6)	0.0272(6)	0.0173(5)	−0.0021(5)	0.0026(5)	0.0029(5)
O1	0.047(3)	0.028(2)	0.027(2)	−0.0038(16)	−0.0103(18)	−0.0070(18)
O2	0.060(3)	0.032(2)	0.0245(19)	−0.0014(16)	−0.004(2)	−0.027(2)
C1	0.016(2)	0.041(3)	0.020(2)	−0.004(2)	0.0049(18)	0.000(2)

C2	0.018(2)	0.033(3)	0.021(2)	−0.002(2)	0.0010(18)	0.003(2)
C3	0.020(2)	0.027(3)	0.018(2)	−0.0002(19)	−0.0010(19)	−0.005(2)
C4	0.017(2)	0.022(2)	0.015(2)	−0.0025(18)	0.0005(17)	−0.0005(18)
C5	0.017(2)	0.035(3)	0.029(3)	−0.001(2)	0.003(2)	−0.006(2)
C6	0.016(2)	0.022(2)	0.019(2)	−0.0027(18)	−0.0001(18)	−0.0053(19)
C7	0.016(2)	0.021(2)	0.015(2)	0.0000(18)	0.0029(17)	−0.0026(19)
C8	0.028(3)	0.021(2)	0.024(3)	−0.003(2)	−0.005(2)	−0.003(2)
C9	0.033(3)	0.015(2)	0.030(3)	−0.002(2)	−0.004(2)	−0.002(2)
C10	0.021(2)	0.024(2)	0.012(2)	0.0031(18)	0.0026(19)	0.0004(19)
C11	0.018(2)	0.024(3)	0.019(2)	−0.002(2)	0.0025(18)	0.0010(19)
C12	0.018(2)	0.020(2)	0.020(2)	−0.0009(19)	0.0009(19)	−0.0005(18)
C13	0.034(3)	0.026(3)	0.027(3)	0.005(2)	−0.001(2)	−0.001(2)
C14	0.016(2)	0.016(2)	0.017(2)	0.0001(18)	−0.0024(16)	0.0012(18)
C15	0.018(2)	0.023(3)	0.024(2)	0.000(2)	−0.0018(19)	0.003(2)
C16	0.019(2)	0.025(3)	0.030(3)	0.004(2)	−0.001(2)	0.003(2)
C17	0.017(2)	0.028(3)	0.030(3)	0.013(2)	0.003(2)	0.002(2)
C18	0.017(2)	0.035(3)	0.023(2)	0.002(2)	0.0066(19)	0.007(2)
C19	0.019(2)	0.021(2)	0.022(2)	0.0001(19)	0.0002(18)	0.000(2)
C20	0.040(4)	0.040(4)	0.058(4)	0.024(3)	0.021(3)	0.010(3)
C21	0.018(2)	0.035(3)	0.029(3)	0.001(2)	0.003(2)	0.003(2)
C22	0.018(2)	0.036(3)	0.025(2)	0.000(2)	0.0026(19)	0.008(2)
C23	0.017(2)	0.024(2)	0.017(2)	0.0013(18)	0.0032(18)	−0.0016(19)
C24	0.017(2)	0.019(2)	0.019(2)	−0.0052(18)	0.0021(18)	0.0021(18)
C25	0.019(2)	0.038(3)	0.020(2)	0.000(2)	0.0011(19)	−0.005(2)
C26	0.020(2)	0.027(3)	0.022(3)	0.002(2)	−0.0039(19)	−0.004(2)
C27	0.019(2)	0.024(2)	0.016(2)	−0.0003(19)	−0.0010(17)	−0.004(2)
C28	0.026(3)	0.021(2)	0.019(2)	−0.0003(19)	−0.0013(19)	0.000(2)
C29	0.030(3)	0.021(3)	0.029(3)	0.005(2)	−0.004(2)	−0.003(2)
C30	0.021(3)	0.038(3)	0.029(3)	0.011(2)	−0.001(2)	−0.004(2)
C31	0.021(2)	0.035(3)	0.020(2)	0.006(2)	0.0041(18)	0.000(2)
C32	0.023(2)	0.023(2)	0.020(2)	−0.0010(19)	0.0020(18)	−0.001(2)
C33	0.032(3)	0.057(4)	0.058(4)	0.037(4)	0.013(3)	0.002(3)
C34	0.019(2)	0.019(2)	0.016(2)	0.0035(18)	0.0041(18)	−0.0012(19)
C35	0.021(2)	0.021(2)	0.020(2)	−0.0023(19)	0.003(2)	−0.0030(19)
C36	0.017(2)	0.025(3)	0.018(2)	0.003(2)	0.0020(18)	−0.0041(19)
C37	0.027(2)	0.018(2)	0.026(3)	0.001(2)	0.009(2)	0.000(2)
C38	0.031(3)	0.020(3)	0.029(3)	0.000(2)	−0.002(2)	−0.002(2)
C39	0.032(3)	0.023(3)	0.021(2)	0.000(2)	−0.004(2)	−0.003(2)
C40	0.040(3)	0.025(3)	0.029(3)	0.005(2)	−0.001(3)	0.012(3)
C1_1	0.018(2)	0.0274(19)	0.0131(17)	−0.0034(15)	−0.0044(16)	−0.0004(18)
C2_1	0.017(2)	0.0283(19)	0.0151(18)	−0.0022(15)	−0.0053(16)	−0.0040(17)
C3_1	0.023(2)	0.024(2)	0.0201(19)	−0.0019(15)	−0.0030(17)	−0.0008(18)
C4_1	0.023(2)	0.0258(19)	0.0210(19)	−0.0018(16)	−0.0011(17)	0.0042(18)
C4A_1	0.017(2)	0.0260(18)	0.0178(17)	−0.0018(14)	−0.0014(15)	0.0036(17)
C5_1	0.024(2)	0.032(2)	0.0193(19)	−0.0006(16)	0.0025(17)	0.0049(19)
C6_1	0.029(3)	0.032(2)	0.024(2)	0.0019(17)	−0.0002(19)	0.000(2)
C7_1	0.029(3)	0.024(2)	0.024(2)	0.0012(16)	−0.0051(18)	−0.0011(19)
C8_1	0.028(3)	0.0216(17)	0.0191(19)	−0.0043(15)	−0.0034(18)	0.0010(19)
C8A_1	0.015(2)	0.0243(17)	0.0158(16)	−0.0023(13)	−0.0039(14)	0.0009(15)
C1_2	0.018(2)	0.0207(18)	0.0174(18)	−0.0058(14)	−0.0016(16)	−0.0006(17)
C2_2	0.016(2)	0.0201(18)	0.0180(18)	−0.0068(15)	−0.0026(16)	−0.0008(16)
C3_2	0.018(2)	0.020(2)	0.0225(19)	−0.0085(15)	−0.0054(17)	0.0010(17)
C4_2	0.021(2)	0.0254(19)	0.0205(19)	−0.0079(16)	−0.0042(17)	0.0024(17)
C4A_2	0.014(2)	0.0245(18)	0.0209(17)	−0.0059(14)	−0.0024(15)	0.0047(15)
C5_2	0.023(2)	0.031(2)	0.0192(19)	−0.0046(16)	−0.0031(18)	0.0046(18)
C6_2	0.023(2)	0.031(2)	0.023(2)	0.0005(17)	−0.0012(18)	0.0083(18)
C7_2	0.023(2)	0.027(2)	0.025(2)	−0.0017(16)	0.0023(17)	0.0025(19)
C8_2	0.020(2)	0.0226(18)	0.022(2)	−0.0045(16)	0.0014(17)	0.0004(17)

C8A_2	0.014(2)	0.0223(17)	0.0190(17)	−0.0043(13)	−0.0005(14)	0.0029(15)
C1_3	0.017(6)	0.024(5)	0.013(5)	0.000(4)	−0.004(5)	−0.001(5)
C2_3	0.018(6)	0.024(5)	0.011(6)	−0.001(4)	−0.001(5)	−0.001(5)
C3_3	0.017(10)	0.020(6)	0.008(7)	0.002(5)	0.001(7)	−0.002(6)
C4_3	0.019(10)	0.021(6)	0.010(7)	0.003(5)	0.000(7)	−0.002(6)
C4A_3	0.014(8)	0.020(6)	0.011(5)	0.003(5)	−0.001(6)	−0.001(6)
C5_3	0.015(10)	0.022(7)	0.012(6)	0.004(6)	−0.001(6)	0.000(7)
C6_3	0.013(10)	0.021(7)	0.010(6)	0.004(6)	0.000(6)	0.001(7)
C7_3	0.020(7)	0.024(6)	0.014(5)	0.001(5)	−0.004(6)	0.000(6)
C8_3	0.021(6)	0.023(4)	0.014(5)	0.001(4)	−0.006(5)	−0.002(5)
C8A_3	0.018(6)	0.023(4)	0.014(5)	0.001(4)	−0.004(5)	−0.001(4)
C1_4	0.016(7)	0.023(5)	0.019(5)	0.000(4)	0.000(5)	0.001(5)
C2_4	0.018(7)	0.023(5)	0.018(6)	0.001(4)	−0.001(6)	0.001(5)
C3_4	0.019(9)	0.022(5)	0.017(6)	0.003(5)	−0.001(6)	0.003(6)
C4_4	0.019(10)	0.022(5)	0.020(7)	0.006(5)	0.002(7)	0.009(7)
C4A_4	0.017(9)	0.022(5)	0.020(6)	0.005(5)	0.003(6)	0.008(6)
C5_4	0.020(11)	0.023(6)	0.022(6)	0.007(6)	0.006(7)	0.012(7)
C6_4	0.020(11)	0.023(6)	0.021(6)	0.006(5)	0.003(7)	0.011(7)
C7_4	0.023(7)	0.024(6)	0.024(6)	0.003(5)	0.006(6)	0.006(6)
C8_4	0.022(6)	0.023(4)	0.022(5)	0.002(4)	0.004(5)	0.004(5)
C8A_4	0.017(6)	0.023(4)	0.021(5)	0.002(4)	0.003(5)	0.004(4)

Table 14. Bond lengths and angles for Werz_dk320HT_1

**Atom–Atom**	**Length [Å]**
S1–C1	1.829(6)
S1–C4	1.890(5)
S2–C21	1.815(6)
S2–C24	1.882(5)
O1–C6	1.227(7)
O2–C26	1.207(7)
C1–C5	1.530(8)
C1–C2	1.535(7)
C1–H1	1.0000
C2–C3	1.564(8)
C2–H2A	0.9900
C2–H2B	0.9900
C3–C6	1.529(7)
C3–C5	1.551(7)
C3–C4	1.572(7)
C4–C14	1.523(7)
C4–C7	1.532(7)
C5–H5A	0.9900
C5–H5B	0.9900
C6–C2_2	1.491(7)
C6–C2_4	1.58(3)
C7–C8	1.395(7)
C7–C12	1.396(7)
C8–C9	1.412(8)
C8–H8	0.9500
C9–C10	1.384(8)
C9–H9	0.9500
C10–C11	1.390(7)
C10–C13	1.507(7)
C11–C12	1.387(7)
C11–H11	0.9500
C12–H12	0.9500
C13–H13A	0.9800
C13–H13B	0.9800
C13–H13C	0.9800
C14–C19	1.398(7)
C14–C15	1.401(7)
C15–C16	1.398(7)
C15–H15	0.9500
C16–C17	1.379(7)
C16–H16	0.9500
C17–C18	1.419(8)
C17–C20	1.497(8)
C18–C19	1.384(7)
C18–H18	0.9500
C19–H19	0.9500
C20–H20A	0.9800
C20–H20B	0.9800
C20–H20C	0.9800
C21–C22	1.548(7)
C21–C25	1.553(8)
C21–H21	1.0000
C22–C23	1.591(7)
C22–H22A	0.9900
C22–H22B	0.9900
C23–C26	1.510(7)
C23–C25	1.560(7)
C23–C24	1.586(7)
C24–C27	1.522(7)
C24–C34	1.527(7)
C25–H25A	0.9900
C25–H25B	0.9900
C26–C2_1	1.499(7)
C26–C2_3	1.50(3)
C27–C28	1.381(8)
C27–C32	1.401(7)
C28–C29	1.397(7)
C28–H28	0.9500
C29–C30	1.389(8)

C29–H29	0.9500
C30–C31	1.392(8)
C30–C33	1.494(8)
C31–C32	1.401(7)
C31–H31	0.9500
C32–H32	0.9500
C33–H33A	0.9800
C33–H33B	0.9800
C33–H33C	0.9800
C34–C39	1.383(7)
C34–C35	1.409(7)
C35–C36	1.384(7)
C35–H35	0.9500
C36–C37	1.400(7)
C36–H36	0.9500
C37–C38	1.378(8)
C37–C40	1.503(7)
C38–C39	1.399(8)
C38–H38	0.9500
C39–H39	0.9500
C40–H40A	0.9800
C40–H40B	0.9800
C40–H40C	0.9800
C1_1–C2_1	1.382(7)
C1_1–C8A_1	1.420(7)
C1_1–H1_1	0.9500
C2_1–C3_1	1.434(7)
C3_1–C4_1	1.361(7)
C3_1–H3_1	0.9500
C4_1–C4A_1	1.420(7)
C4_1–H4_1	0.9500
C4A_1–C5_1	1.416(7)
C4A_1–C8A_1	1.420(6)
C5_1–C6_1	1.370(7)
C5_1–H5_1	0.9500
C6_1–C7_1	1.408(7)
C6_1–H6_1	0.9500
C7_1–C8_1	1.388(7)
C7_1–H7_1	0.9500
C8_1–C8A_1	1.410(7)
C8_1–H8_1	0.9500
C1_2–C2_2	1.387(7)
C1_2–C8A_2	1.415(7)
C1_2–H1_2	0.9500
C2_2–C3_2	1.419(6)
C3_2–C4_2	1.381(7)
C3_2–H3_2	0.9500
C4_2–C4A_2	1.407(7)
C4_2–H4_2	0.9500
C4A_2–C8A_2	1.418(6)
C4A_2–C5_2	1.423(7)
C5_2–C6_2	1.358(7)
C5_2–H5_2	0.9500
C6_2–C7_2	1.419(7)
C6_2–H6_2	0.9500
C7_2–C8_2	1.380(7)
C7_2–H7_2	0.9500
C8_2–C8A_2	1.417(7)

C8_2–H8_2	0.9500
C1_3–C2_3	1.399(18)
C1_3–C8A_3	1.406(17)
C1_3–H1_3	0.9500
C2_3–C3_3	1.405(18)
C3_3–C4_3	1.403(18)
C3_3–H3_3	0.9500
C4_3–C4A_3	1.406(17)
C4_3–H4_3	0.9500
C4A_3–C8A_3	1.410(17)
C4A_3–C5_3	1.413(17)
C5_3–C6_3	1.405(18)
C5_3–H5_3	0.9500
C6_3–C7_3	1.405(18)
C6_3–H6_3	0.9500
C7_3–C8_3	1.399(18)
C7_3–H7_3	0.9500
C8_3–C8A_3	1.402(17)
C8_3–H8_3	0.9500
C1_4–C2_4	1.403(18)
C1_4–C8A_4	1.411(17)
C1_4–H1_4	0.9500
C2_4–C3_4	1.400(18)
C3_4–C4_4	1.401(18)
C3_4–H3_4	0.9500
C4_4–C4A_4	1.411(17)
C4_4–H4_4	0.9500
C4A_4–C5_4	1.407(17)
C4A_4–C8A_4	1.408(17)
C5_4–C6_4	1.403(18)
C5_4–H5_4	0.9500
C6_4–C7_4	1.405(18)
C6_4–H6_4	0.9500
C7_4–C8_4	1.410(18)
C7_4–H7_4	0.9500
C8_4–C8A_4	1.410(17)
C8_4–H8_4	0.9500
**Atom–Atom–Atom**	**Angle [°]**
C1–S1–C4	87.5(2)
C21–S2–C24	88.5(2)
C5–C1–C2	87.4(4)
C5–C1–S1	104.5(4)
C2–C1–S1	101.9(3)
C5–C1–H1	119.1
C2–C1–H1	119.1
S1–C1–H1	119.1
C1–C2–C3	83.6(4)
C1–C2–H2A	114.7
C3–C2–H2A	114.7
C1–C2–H2B	114.7
C3–C2–H2B	114.7
H2A–C2–H2B	111.8
C6–C3–C5	117.3(4)
C6–C3–C2	116.2(4)
C5–C3–C2	85.7(4)
C6–C3–C4	120.4(4)
C5–C3–C4	105.2(4)

C2–C3–C4	106.4(4)
C14–C4–C7	109.9(4)
C14–C4–C3	111.8(4)
C7–C4–C3	117.5(4)
C14–C4–S1	113.3(3)
C7–C4–S1	105.2(3)
C3–C4–S1	98.6(3)
C1–C5–C3	84.2(4)
C1–C5–H5A	114.6
C3–C5–H5A	114.6
C1–C5–H5B	114.6
C3–C5–H5B	114.6
H5A–C5–H5B	111.7
O1–C6–C2_2	117.9(4)
O1–C6–C3	121.3(5)
C2_2–C6–C3	120.1(4)
O1–C6–C2_4	114.0(13)
C3–C6–C2_4	117.7(13)
C8–C7–C12	117.8(5)
C8–C7–C4	124.4(4)
C12–C7–C4	117.8(4)
C7–C8–C9	120.3(5)
C7–C8–H8	119.9
C9–C8–H8	119.9
C10–C9–C8	121.0(5)
C10–C9–H9	119.5
C8–C9–H9	119.5
C9–C10–C11	118.7(5)
C9–C10–C13	121.3(5)
C11–C10–C13	120.1(5)
C12–C11–C10	120.4(5)
C12–C11–H11	119.8
C10–C11–H11	119.8
C11–C12–C7	121.9(5)
C11–C12–H12	119.1
C7–C12–H12	119.1
C10–C13–H13A	109.5
C10–C13–H13B	109.5
H13A–C13–H13B	109.5
C10–C13–H13C	109.5
H13A–C13–H13C	109.5
H13B–C13–H13C	109.5
C19–C14–C15	117.8(5)
C19–C14–C4	118.4(4)
C15–C14–C4	123.5(4)
C16–C15–C14	121.0(5)
C16–C15–H15	119.5
C14–C15–H15	119.5
C17–C16–C15	121.2(5)
C17–C16–H16	119.4
C15–C16–H16	119.4
C16–C17–C18	118.1(5)
C16–C17–C20	121.1(5)
C18–C17–C20	120.8(5)
C19–C18–C17	120.6(5)
C19–C18–H18	119.7
C17–C18–H18	119.7
C18–C19–C14	121.3(5)
C18–C19–H19	119.4
C14–C19–H19	119.4
C17–C20–H20A	109.5
C17–C20–H20B	109.5
H20A–C20–H20B	109.5
C17–C20–H20C	109.5
H20A–C20–H20C	109.5
H20B–C20–H20C	109.5
C22–C21–C25	87.4(4)
C22–C21–S2	101.5(4)
C25–C21–S2	104.7(4)
C22–C21–H21	119.2
C25–C21–H21	119.2
S2–C21–H21	119.2
C21–C22–C23	83.5(4)
C21–C22–H22A	114.7
C23–C22–H22A	114.7
C21–C22–H22B	114.7
C23–C22–H22B	114.7
H22A–C22–H22B	111.8
C26–C23–C25	118.4(4)
C26–C23–C24	118.9(4)
C25–C23–C24	105.0(4)
C26–C23–C22	118.1(4)
C25–C23–C22	85.7(4)
C24–C23–C22	105.3(4)
C27–C24–C34	110.7(4)
C27–C24–C23	111.4(4)
C34–C24–C23	115.2(4)
C27–C24–S2	113.6(3)
C34–C24–S2	106.6(3)
C23–C24–S2	98.7(3)
C21–C25–C23	84.4(4)
C21–C25–H25A	114.6
C23–C25–H25A	114.6
C21–C25–H25B	114.6
C23–C25–H25B	114.6
H25A–C25–H25B	111.7
O2–C26–C2_1	118.7(5)
O2–C26–C2_3	105.9(13)
O2–C26–C23	119.9(5)
C2_1–C26–C23	121.3(4)
C2_3–C26–C23	124.0(14)
C28–C27–C32	117.9(5)
C28–C27–C24	124.0(5)
C32–C27–C24	118.1(5)
C27–C28–C29	120.7(5)
C27–C28–H28	119.6
C29–C28–H28	119.6
C30–C29–C28	122.2(5)
C30–C29–H29	118.9
C28–C29–H29	118.9
C29–C30–C31	117.0(5)
C29–C30–C33	121.6(6)
C31–C30–C33	121.3(6)
C30–C31–C32	121.3(5)
C30–C31–H31	119.4
C32–C31–H31	119.4

C27–C32–C31	120.8(5)
C27–C32–H32	119.6
C31–C32–H32	119.6
C30–C33–H33A	109.5
C30–C33–H33B	109.5
H33A–C33–H33B	109.5
C30–C33–H33C	109.5
H33A–C33–H33C	109.5
H33B–C33–H33C	109.5
C39–C34–C35	117.2(5)
C39–C34–C24	126.3(5)
C35–C34–C24	116.5(4)
C36–C35–C34	121.3(5)
C36–C35–H35	119.3
C34–C35–H35	119.3
C35–C36–C37	121.2(5)
C35–C36–H36	119.4
C37–C36–H36	119.4
C38–C37–C36	117.1(5)
C38–C37–C40	123.0(5)
C36–C37–C40	119.8(5)
C37–C38–C39	122.1(5)
C37–C38–H38	118.9
C39–C38–H38	118.9
C34–C39–C38	120.9(5)
C34–C39–H39	119.5
C38–C39–H39	119.5
C37–C40–H40A	109.5
C37–C40–H40B	109.5
H40A–C40–H40B	109.5
C37–C40–H40C	109.5
H40A–C40–H40C	109.5
H40B–C40–H40C	109.5
C2_1–C1_1–C8A_1	120.8(5)
C2_1–C1_1–H1_1	119.6
C8A_1–C1_1–H1_1	119.6
C1_1–C2_1–C3_1	119.3(5)
C1_1–C2_1–C26	124.7(5)
C3_1–C2_1–C26	116.0(5)
C4_1–C3_1–C2_1	120.8(5)
C4_1–C3_1–H3_1	119.6
C2_1–C3_1–H3_1	119.6
C3_1–C4_1–C4A_1	120.7(5)
C3_1–C4_1–H4_1	119.7
C4A_1–C4_1–H4_1	119.7
C5_1–C4A_1–C4_1	122.3(5)
C5_1–C4A_1–C8A_1	118.4(5)
C4_1–C4A_1–C8A_1	119.4(4)
C6_1–C5_1–C4A_1	121.2(5)
C6_1–C5_1–H5_1	119.4
C4A_1–C5_1–H5_1	119.4
C5_1–C6_1–C7_1	120.3(5)
C5_1–C6_1–H6_1	119.8
C7_1–C6_1–H6_1	119.8
C8_1–C7_1–C6_1	120.0(5)
C8_1–C7_1–H7_1	120.0

C6_1–C7_1–H7_1	120.0
C7_1–C8_1–C8A_1	120.2(5)
C7_1–C8_1–H8_1	119.9
C8A_1–C8_1–H8_1	119.9
C8_1–C8A_1–C4A_1	119.8(4)
C8_1–C8A_1–C1_1	121.1(5)
C4A_1–C8A_1–C1_1	119.1(5)
C2_2–C1_2–C8A_2	120.7(5)
C2_2–C1_2–H1_2	119.6
C8A_2–C1_2–H1_2	119.6
C1_2–C2_2–C3_2	119.0(5)
C1_2–C2_2–C6	123.7(4)
C3_2–C2_2–C6	117.3(4)
C4_2–C3_2–C2_2	121.3(5)
C4_2–C3_2–H3_2	119.4
C2_2–C3_2–H3_2	119.4
C3_2–C4_2–C4A_2	120.0(5)
C3_2–C4_2–H4_2	120.0
C4A_2–C4_2–H4_2	120.0
C4_2–C4A_2–C8A_2	119.6(4)
C4_2–C4A_2–C5_2	122.0(4)
C8A_2–C4A_2–C5_2	118.4(5)
C6_2–C5_2–C4A_2	121.4(5)
C6_2–C5_2–H5_2	119.3
C4A_2–C5_2–H5_2	119.3
C5_2–C6_2–C7_2	120.4(5)
C5_2–C6_2–H6_2	119.8
C7_2–C6_2–H6_2	119.8
C8_2–C7_2–C6_2	119.6(5)
C8_2–C7_2–H7_2	120.2
C6_2–C7_2–H7_2	120.2
C7_2–C8_2–C8A_2	120.8(5)
C7_2–C8_2–H8_2	119.6
C8A_2–C8_2–H8_2	119.6
C1_2–C8A_2–C8_2	121.3(5)
C1_2–C8A_2–C4A_2	119.4(4)
C8_2–C8A_2–C4A_2	119.4(4)
C2_3–C1_3–C8A_3	121(2)
C2_3–C1_3–H1_3	119.7
C8A_3–C1_3–H1_3	119.7
C1_3–C2_3–C3_3	120(2)
C1_3–C2_3–C26	116(2)
C3_3–C2_3–C26	124(2)
C4_3–C3_3–C2_3	120(2)
C4_3–C3_3–H3_3	120.2
C2_3–C3_3–H3_3	120.2
C3_3–C4_3–C4A_3	121(2)
C3_3–C4_3–H4_3	119.6
C4A_3–C4_3–H4_3	119.6
C4_3–C4A_3–C8A_3	119.5(18)
C4_3–C4A_3–C5_3	119(2)

C8A_3–C4A_3–C5_3	121.2(18)
C6_3–C5_3–C4A_3	118(2)
C6_3–C5_3–H5_3	120.8
C4A_3–C5_3–H5_3	120.8
C5_3–C6_3–C7_3	120(2)
C5_3–C6_3–H6_3	119.9
C7_3–C6_3–H6_3	119.9
C8_3–C7_3–C6_3	121(2)
C8_3–C7_3–H7_3	119.6
C6_3–C7_3–H7_3	119.6
C7_3–C8_3–C8A_3	120(2)
C7_3–C8_3–H8_3	120.2
C8A_3–C8_3–H8_3	120.2
C8_3–C8A_3–C1_3	121(2)
C8_3–C8A_3–C4A_3	119.4(18)
C1_3–C8A_3–C4A_3	119.6(18)
C2_4–C1_4–C8A_4	119(2)
C2_4–C1_4–H1_4	120.5
C8A_4–C1_4–H1_4	120.5
C3_4–C2_4–C1_4	121(2)
C3_4–C2_4–C6	114(2)
C1_4–C2_4–C6	125(2)
C2_4–C3_4–C4_4	120(2)
C2_4–C3_4–H3_4	120.2
C4_4–C3_4–H3_4	120.2
C3_4–C4_4–C4A_4	120(2)
C3_4–C4_4–H4_4	120.0
C4A_4–C4_4–H4_4	120.0
C5_4–C4A_4–C8A_4	118.7(18)
C5_4–C4A_4–C4_4	121(2)
C8A_4–C4A_4–C4_4	119.8(18)
C6_4–C5_4–C4A_4	120(2)
C6_4–C5_4–H5_4	120.2
C4A_4–C5_4–H5_4	120.2
C5_4–C6_4–C7_4	121(2)
C5_4–C6_4–H6_4	119.4
C7_4–C6_4–H6_4	119.4
C6_4–C7_4–C8_4	118(2)
C6_4–C7_4–H7_4	120.9
C8_4–C7_4–H7_4	120.9
C7_4–C8_4–C8A_4	119(2)
C7_4–C8_4–H8_4	120.4
C8A_4–C8_4–H8_4	120.4
C4A_4–C8A_4–C8_4	121.2(18)
C4A_4–C8A_4–C1_4	120.3(18)
C8_4–C8A_4–C1_4	118(2)

Table 15. Torsion angles for Werz_dk320HT_1

Atom–Atom–Atom–Atom	Torsion Angle [°]
C4–S1–C1–C5	42.2(3)
C4–S1–C1–C2	−48.2(4)
C5–C1–C2–C3	−32.2(4)
S1–C1–C2–C3	72.1(4)
C1–C2–C3–C6	150.2(5)
C1–C2–C3–C5	31.8(4)
C1–C2–C3–C4	−72.8(4)
C6–C3–C4–C14	57.6(6)
C5–C3–C4–C14	−167.2(4)
C2–C3–C4–C14	−77.2(5)
C6–C3–C4–C7	−70.8(6)
C5–C3–C4–C7	64.5(5)
C2–C3–C4–C7	154.4(4)
C6–C3–C4–S1	177.0(4)
C5–C3–C4–S1	−47.8(4)
C2–C3–C4–S1	42.2(4)
C1–S1–C4–C14	121.3(4)
C1–S1–C4–C7	−118.6(4)
C1–S1–C4–C3	3.1(3)
C2–C1–C5–C3	32.5(4)
S1–C1–C5–C3	−69.2(4)
C6–C3–C5–C1	−149.2(5)
C2–C3–C5–C1	−31.9(4)
C4–C3–C5–C1	73.9(4)
C5–C3–C6–O1	−44.2(7)
C2–C3–C6–O1	−143.4(5)
C4–C3–C6–O1	85.9(7)
C5–C3–C6–C2_2	125.9(5)
C2–C3–C6–C2_2	26.7(7)
C4–C3–C6–C2_2	−104.0(6)
C5–C3–C6–C2_4	167.0(15)
C2–C3–C6–C2_4	67.8(15)
C4–C3–C6–C2_4	−62.9(15)
C14–C4–C7–C8	−112.3(5)
C3–C4–C7–C8	17.0(7)
S1–C4–C7–C8	125.4(5)
C14–C4–C7–C12	67.2(5)
C3–C4–C7–C12	−163.5(4)
S1–C4–C7–C12	−55.1(5)
C12–C7–C8–C9	−0.4(8)
C4–C7–C8–C9	179.1(5)
C7–C8–C9–C10	1.6(9)
C8–C9–C10–C11	−1.0(8)
C8–C9–C10–C13	178.5(5)
C9–C10–C11–C12	−0.8(8)
C13–C10–C11–C12	179.7(5)
C10–C11–C12–C7	2.0(8)
C8–C7–C12–C11	−1.4(7)
C4–C7–C12–C11	179.0(4)
C7–C4–C14–C19	49.4(6)
C3–C4–C14–C19	−82.9(5)
S1–C4–C14–C19	166.8(4)

C7–C4–C14–C15	−136.5(5)
C3–C4–C14–C15	91.2(6)
S1–C4–C14–C15	−19.1(6)
C19–C14–C15–C16	−2.0(7)
C4–C14–C15–C16	−176.1(4)
C14–C15–C16–C17	0.6(8)
C15–C16–C17–C18	0.7(8)
C15–C16–C17–C20	−177.6(6)
C16–C17–C18–C19	−0.7(8)
C20–C17–C18–C19	177.7(6)
C17–C18–C19–C14	−0.7(8)
C15–C14–C19–C18	2.1(7)
C4–C14–C19–C18	176.5(5)
C24–S2–C21–C22	48.7(4)
C24–S2–C21–C25	−41.6(3)
C25–C21–C22–C23	32.0(4)
S2–C21–C22–C23	−72.4(4)
C21–C22–C23–C26	−151.9(5)
C21–C22–C23–C25	−31.9(4)
C21–C22–C23–C24	72.5(4)
C26–C23–C24–C27	−57.4(6)
C25–C23–C24–C27	167.3(4)
C22–C23–C24–C27	77.8(5)
C26–C23–C24–C34	69.8(6)
C25–C23–C24–C34	−65.4(5)
C22–C23–C24–C34	−155.0(4)
C26–C23–C24–S2	−177.1(4)
C25–C23–C24–S2	47.7(4)
C22–C23–C24–S2	−41.9(4)
C21–S2–C24–C27	−121.1(4)
C21–S2–C24–C34	116.7(4)
C21–S2–C24–C23	−3.1(3)
C22–C21–C25–C23	−32.7(4)
S2–C21–C25–C23	68.6(4)
C26–C23–C25–C21	151.5(5)
C24–C23–C25–C21	−72.9(4)
C22–C23–C25–C21	31.7(4)
C25–C23–C26–O2	21.2(8)
C24–C23–C26–O2	−108.2(6)
C22–C23–C26–O2	122.2(6)
C25–C23–C26–C2_1	−161.2(5)
C24–C23–C26–C2_1	69.4(6)
C22–C23–C26–C2_1	−60.1(7)
C25–C23–C26–C2_3	−119.2(17)
C24–C23–C26–C2_3	111.4(17)
C22–C23–C26–C2_3	−18.1(18)
C34–C24–C27–C28	141.9(5)
C23–C24–C27–C28	−88.4(6)
S2–C24–C27–C28	22.0(6)
C34–C24–C27–C32	−41.1(6)
C23–C24–C27–C32	88.5(5)
S2–C24–C27–C32	−161.1(4)
C32–C27–C28–C29	−3.0(8)
C24–C27–C28–C29	173.9(5)
C27–C28–C29–C30	0.4(8)
C28–C29–C30–C31	1.0(8)
C28–C29–C30–C33	−179.7(6)
C29–C30–C31–C32	0.1(8)

C33–C30–C31–C32	−179.1(6)
C28–C27–C32–C31	4.1(8)
C24–C27–C32–C31	−173.0(5)
C30–C31–C32–C27	−2.8(8)
C27–C24–C34–C39	110.2(6)
C23–C24–C34–C39	−17.4(7)
S2–C24–C34–C39	−125.8(5)
C27–C24–C34–C35	−68.8(5)
C23–C24–C34–C35	163.7(4)
S2–C24–C34–C35	55.3(5)
C39–C34–C35–C36	0.1(7)
C24–C34–C35–C36	179.1(4)
C34–C35–C36–C37	−0.9(8)
C35–C36–C37–C38	0.5(8)
C35–C36–C37–C40	178.8(5)
C36–C37–C38–C39	0.8(8)
C40–C37–C38–C39	−177.5(5)
C35–C34–C39–C38	1.2(8)
C24–C34–C39–C38	−177.8(5)
C37–C38–C39–C34	−1.7(9)
C8A_1–C1_1–C2_1–C3_1	0.0(6)
C8A_1–C1_1–C2_1–C26	178.4(5)
O2–C26–C2_1–C1_1	−150.4(5)
C23–C26–C2_1–C1_1	31.9(7)
O2–C26–C2_1–C3_1	28.0(7)
C23–C26–C2_1–C3_1	−149.7(5)
C1_1–C2_1–C3_1–C4_1	−0.4(6)
C26–C2_1–C3_1–C4_1	−179.0(5)
C2_1–C3_1–C4_1–C4A_1	1.1(8)
C3_1–C4_1–C4A_1–C5_1	178.8(5)
C3_1–C4_1–C4A_1–C8A_1	−1.3(8)
C4_1–C4A_1–C5_1–C6_1	179.1(6)
C8A_1–C4A_1–C5_1–C6_1	−0.8(8)
C4A_1–C5_1–C6_1–C7_1	2.0(9)
C5_1–C6_1–C7_1–C8_1	−2.2(9)
C6_1–C7_1–C8_1–C8A_1	1.3(9)
C7_1–C8_1–C8A_1–C4A_1	−0.2(8)
C7_1–C8_1–C8A_1–C1_1	178.9(5)
C5_1–C4A_1–C8A_1–C8_1	0.0(8)
C4_1–C4A_1–C8A_1–C8_1	−180.0(5)
C5_1–C4A_1–C8A_1–C1_1	−179.2(5)
C4_1–C4A_1–C8A_1–C1_1	0.9(7)
C2_1–C1_1–C8A_1–C8_1	−179.4(5)
C2_1–C1_1–C8A_1–C4A_1	−0.3(7)
C8A_2–C1_2–C2_2–C3_2	−0.3(6)
C8A_2–C1_2–C2_2–C6	178.9(5)
O1–C6–C2_2–C1_2	−151.8(5)
C3–C6–C2_2–C1_2	37.7(7)
O1–C6–C2_2–C3_2	27.4(7)
C3–C6–C2_2–C3_2	−143.1(5)

C1_2–C2_2–C3_2–C4_2	−1.5(6)
C6–C2_2–C3_2–C4_2	179.2(5)
C2_2–C3_2–C4_2–C4A_2	2.2(8)
C3_2–C4_2–C4A_2–C8A_2	−1.2(8)
C3_2–C4_2–C4A_2–C5_2	179.1(5)
C4_2–C4A_2–C5_2–C6_2	179.6(5)
C8A_2–C4A_2–C5_2–C6_2	−0.2(8)
C4A_2–C5_2–C6_2–C7_2	1.8(9)
C5_2–C6_2–C7_2–C8_2	−1.6(9)
C6_2–C7_2–C8_2–C8A_2	−0.2(8)
C2_2–C1_2–C8A_2–C8_2	−177.9(5)
C2_2–C1_2–C8A_2–C4A_2	1.3(7)
C7_2–C8_2–C8A_2–C1_2	−179.0(5)
C7_2–C8_2–C8A_2–C4A_2	1.8(8)
C4_2–C4A_2–C8A_2–C1_2	−0.6(7)
C5_2–C4A_2–C8A_2–C1_2	179.2(5)
C4_2–C4A_2–C8A_2–C8_2	178.7(5)
C5_2–C4A_2–C8A_2–C8_2	−1.6(7)
C8A_3–C1_3–C2_3–C3_3	0(2)
C8A_3–C1_3–C2_3–C26	−178(4)
O2–C26–C2_3–C1_3	173.7(12)
C23–C26–C2_3–C1_3	−41(2)
O2–C26–C2_3–C3_3	−4(3)
C23–C26–C2_3–C3_3	141(2)
C1_3–C2_3–C3_3–C4_3	0(3)
C26–C2_3–C3_3–C4_3	178(4)
C2_3–C3_3–C4_3–C4A_3	1(5)
C3_3–C4_3–C4A_3–C8A_3	0(6)
C3_3–C4_3–C4A_3–C5_3	178(4)
C4_3–C4A_3–C5_3–C6_3	177(4)
C8A_3–C4A_3–C5_3–C6_3	−5(6)
C4A_3–C5_3–C6_3–C7_3	6(6)
C5_3–C6_3–C7_3–C8_3	−3(7)
C6_3–C7_3–C8_3–C8A_3	0(7)
C7_3–C8_3–C8A_3–C1_3	−178(4)
C7_3–C8_3–C8A_3–C4A_3	1(7)
C2_3–C1_3–C8A_3–C8_3	−180(3)
C2_3–C1_3–C8A_3–C4A_3	1(5)
C4_3–C4A_3–C8A_3–C8_3	180(4)
C5_3–C4A_3–C8A_3–C8_3	2(7)
C4_3–C4A_3–C8A_3–C1_3	−1(6)
C5_3–C4A_3–C8A_3–C1_3	−179(4)
C8A_4–C1_4–C2_4–C3_4	−1(2)
C8A_4–C1_4–C2_4–C6	−171(4)
O1–C6–C2_4–C3_4	3(2)
C3–C6–C2_4–C3_4	153.6(16)
O1–C6–C2_4–C1_4	173.8(14)
C3–C6–C2_4–C1_4	−35(2)
C1_4–C2_4–C3_4–C4_4	1(3)
C6–C2_4–C3_4–C4_4	173(3)
C2_4–C3_4–C4_4–C4A_4	−2(5)
C3_4–C4_4–C4A_4–C5_4	−177(4)
C3_4–C4_4–C4A_4–C8A_4	2(7)
C8A_4–C4A_4–C5_4–C6_4	−1(7)
C4_4–C4A_4–C5_4–C6_4	178(4)
C4A_4–C5_4–C6_4–C7_4	9(7)
C5_4–C6_4–C7_4–C8_4	−17(7)
C6_4–C7_4–C8_4–C8A_4	15(7)
C5_4–C4A_4–C8A_4–C8_4	0(7)
C4_4–C4A_4–C8A_4–C8_4	−178(5)
C5_4–C4A_4–C8A_4–C1_4	178(4)
C4_4–C4A_4–C8A_4–C1_4	−1(7)
C7_4–C8_4–C8A_4–C4A_4	−8(7)
C7_4–C8_4–C8A_4–C1_4	175(4)
C2_4–C1_4–C8A_4–C4A_4	0(5)
C2_4–C1_4–C8A_4–C8_4	178(3)

Structure Tables

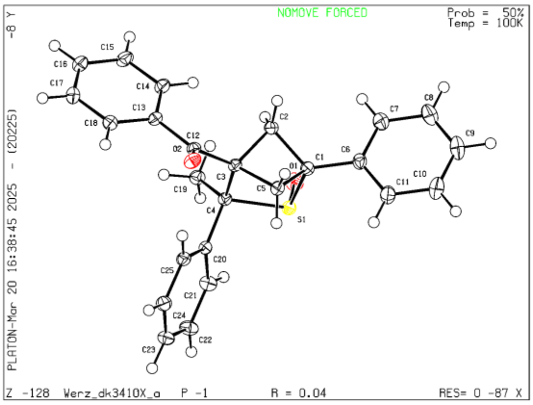

Crystals were obtained at room temperature by slow solvent evaporation from a solution of the compound dissolved in ethyl acetate. A colourless, block-shaped crystal was mounted on a MiTeGen micromount with perfluoroether oil. Data for Werz_dk341OX_a were collected from a shock-cooled single crystal at 100(2) K on a Bruker D8 VENTURE dual wavelength Mo/Cu three-circle diffractometer with a microfocus sealed X-ray tube using a mirror optics as monochromator and a Bruker PHOTON III detector. The diffractometer was equipped with an Oxford Cryostream 800 low temperature device and used MoK_α radiation (λ = 0.71073 Å). All data were integrated with SAINT V8.41 and a multi-scan absorption correction using SADABS 2016/2 was applied.[8,9] The structure was solved by direct methods with SHELXT 2018/2 and refined by full-matrix least-squares methods against F^2 using SHELXL-2019/2.[10,11] All non-hydrogen atoms were refined with anisotropic displacement parameters. All hydrogen atoms were refined isotropic on calculated positions using a riding model with their U_{iso} values constrained to 1.5 times the U_{eq} of their pivot atoms for terminal sp^3 carbon atoms and 1.2 times for all other carbon atoms. Crystallographic data for the structures reported in this paper have been deposited with the Cambridge Crystallographic Data Centre.[12] CCDC 2432823 contain the supplementary crystallographic data for this paper. These data can be obtained free of charge from The Cambridge Crystallographic Data Centre via www.ccdc.cam.ac.uk/structures. This report and the CIF file were generated using FinalCif.[13]

Table 16. Crystal data and structure refinement for Werz_dk341OX_a

CCDC number	2432823
Empirical formula	$C_{25}H_{22}O_2S$
Formula weight	386.48
Temperature [K]	100(2)
Crystal system	triclinic
Space group (number)	$P\overline{1}$ (2)
a [Å]	9.639(3)
b [Å]	9.825(3)
c [Å]	11.340(3)
α [°]	89.784(7)
β [°]	66.003(16)
γ [°]	85.593(8)
Volume [Å^3]	977.7(5)
Z	2
ρ_{calc} [gcm^{-3}]	1.313
μ [mm^{-1}]	0.184
F(000)	408
Crystal size [mm^3]	0.084×0.204×0.305
Crystal colour	colourless
Crystal shape	block
Radiation	MoK_α (λ=0.71073 Å)
2θ range [°]	3.93 to 61.24 (0.70 Å)
Index ranges	−13 ≤ h ≤ 13<br>−14 ≤ k ≤ 14<br>−16 ≤ l ≤ 16
Reflections collected	69669
Independent reflections	6026<br>R_{int} = 0.0608<br>R_{sigma} = 0.0266
Completeness to θ = 25.242°	100.0 %
Data / Restraints / Parameters	6026 / 0 / 254
Absorption correction T_{min}/T_{max} (method)	0.6716 / 0.7461 (multi-scan)
Goodness-of-fit on F^2	1.051
Final R indexes [I≥2σ(I)]	R_1 = 0.0415<br>wR_2 = 0.1061
Final R indexes [all data]	R_1 = 0.0521<br>wR_2 = 0.1127
Largest peak/hole [eÅ^{-3}]	0.44/−0.42

Table 17. Atomic coordinates and U_{eq} [Å^2] for Werz_dk341OX_a

Atom	*x*	*y*	*z*	U_{eq}
S1	0.22811(3)	0.10318(3)	0.43607(3)	0.01654(8)
O1	0.28295(12)	−0.02098(10)	0.48496(10)	0.0242(2)
O2	0.46693(10)	0.53567(9)	0.33087(9)	0.02003(19)
C1	0.19892(14)	0.24729(12)	0.54947(11)	0.0159(2)
C2	0.36135(14)	0.25843(13)	0.54080(11)	0.0164(2)
H2A	0.417739	0.169846	0.540780	0.020
H2B	0.368472	0.325602	0.602548	0.020
C3	0.39415(13)	0.31456(12)	0.40288(11)	0.0143(2)
C4	0.40376(13)	0.18706(12)	0.31795(11)	0.0148(2)
C5	0.22362(13)	0.36989(12)	0.45868(12)	0.0160(2)
H5A	0.201872	0.460561	0.503097	0.019
H5B	0.174766	0.365572	0.397188	0.019
C6	0.06142(14)	0.24042(13)	0.67309(12)	0.0182(2)
C7	0.07443(17)	0.18799(15)	0.78291(14)	0.0256(3)
H7	0.171170	0.153354	0.778683	0.031
C8	−0.05424(19)	0.18626(17)	0.89899(14)	0.0318(3)
H8	−0.044552	0.152381	0.974090	0.038
C9	−0.19609(18)	0.23382(16)	0.90500(15)	0.0310(3)
H9	−0.283584	0.232335	0.984096	0.037
C10	−0.21039(16)	0.28368(16)	0.79538(15)	0.0293(3)
H10	−0.307918	0.314964	0.799215	0.035
C11	−0.08195(15)	0.28788(15)	0.67985(13)	0.0238(3)
H11	−0.091942	0.323179	0.605335	0.029
C12	0.51066(13)	0.41981(12)	0.34819(11)	0.0155(2)
C13	0.67579(13)	0.38437(12)	0.31378(12)	0.0161(2)
C14	0.72922(14)	0.28680(13)	0.37844(13)	0.0192(2)
H14	0.659155	0.239076	0.447285	0.023
C15	0.88512(15)	0.25949(14)	0.34200(14)	0.0224(3)
H15	0.921436	0.192910	0.385912	0.027
C16	0.98757(15)	0.32913(15)	0.24182(13)	0.0237(3)
H16	1.093942	0.308768	0.216246	0.028
C17	0.93563(15)	0.42881(15)	0.17836(13)	0.0239(3)
H17	1.006218	0.477850	0.111084	0.029
C18	0.78042(15)	0.45607(14)	0.21389(12)	0.0198(2)
H18	0.744677	0.523612	0.170425	0.024
C19	0.54197(14)	0.08366(13)	0.28922(12)	0.0183(2)
H19A	0.632929	0.119840	0.224008	0.028
H19B	0.523917	−0.002533	0.256722	0.028
H19C	0.557351	0.067628	0.368575	0.028
C20	0.37941(13)	0.21299(12)	0.19515(11)	0.0152(2)
C21	0.34469(15)	0.10386(13)	0.13554(13)	0.0200(2)
H21	0.332113	0.017452	0.174779	0.024
C22	0.32838(16)	0.12002(14)	0.01996(13)	0.0227(3)
H22	0.304346	0.044904	−0.018754	0.027
C23	0.34693(15)	0.24506(14)	−0.03925(13)	0.0220(3)
H23	0.338464	0.255327	−0.119457	0.026
C24	0.37803(15)	0.35517(14)	0.01992(13)	0.0210(2)
H24	0.389113	0.441603	−0.019259	0.025
C25	0.39302(14)	0.33950(13)	0.13635(12)	0.0181(2)
H25	0.412811	0.415946	0.176460	0.022

U_{eq} is defined as 1/3 of the trace of the orthogonalized U_{ij} tensor.

Table 18. Anisotropic displacement parameters [Å^2] for Werz_dk341OX_a. The anisotropic displacement factor exponent takes the form: $-2\pi^2[\, h^2(a^*)^2U_{11} + k^2(b^*)^2U_{22} + \ldots + 2hka^*b^*U_{12}\,]$

Atom	U_{11}	U_{22}	U_{33}	U_{23}	U_{13}	U_{12}

S1	0.01783(14)	0.01554(14)	0.01716(14)	0.00196(10)	−0.00751(11)	−0.00478(10)
O1	0.0296(5)	0.0171(4)	0.0266(5)	0.0055(4)	−0.0117(4)	−0.0040(4)
O2	0.0200(4)	0.0156(4)	0.0276(5)	0.0024(3)	−0.0125(4)	−0.0031(3)
C1	0.0160(5)	0.0161(5)	0.0159(5)	0.0007(4)	−0.0066(4)	−0.0025(4)
C2	0.0167(5)	0.0179(5)	0.0159(5)	0.0017(4)	−0.0077(4)	−0.0032(4)
C3	0.0140(5)	0.0139(5)	0.0159(5)	0.0014(4)	−0.0069(4)	−0.0021(4)
C4	0.0147(5)	0.0144(5)	0.0160(5)	0.0010(4)	−0.0069(4)	−0.0019(4)
C5	0.0148(5)	0.0153(5)	0.0178(5)	0.0013(4)	−0.0064(4)	−0.0017(4)
C6	0.0180(5)	0.0172(5)	0.0176(5)	0.0004(4)	−0.0048(4)	−0.0044(4)
C7	0.0242(6)	0.0305(7)	0.0214(6)	0.0056(5)	−0.0081(5)	−0.0048(5)
C8	0.0343(8)	0.0361(8)	0.0202(6)	0.0077(6)	−0.0056(6)	−0.0061(6)
C9	0.0281(7)	0.0286(7)	0.0245(7)	0.0036(6)	0.0017(6)	−0.0052(6)
C10	0.0191(6)	0.0283(7)	0.0319(7)	0.0030(6)	−0.0018(5)	−0.0013(5)
C11	0.0192(6)	0.0262(7)	0.0228(6)	0.0033(5)	−0.0055(5)	−0.0018(5)
C12	0.0157(5)	0.0163(5)	0.0163(5)	0.0003(4)	−0.0081(4)	−0.0028(4)
C13	0.0149(5)	0.0163(5)	0.0183(5)	−0.0001(4)	−0.0079(4)	−0.0026(4)
C14	0.0171(5)	0.0197(6)	0.0230(6)	0.0044(5)	−0.0100(5)	−0.0044(4)
C15	0.0182(6)	0.0245(6)	0.0281(7)	0.0043(5)	−0.0130(5)	−0.0017(5)
C16	0.0160(5)	0.0310(7)	0.0244(6)	−0.0006(5)	−0.0087(5)	−0.0011(5)
C17	0.0182(6)	0.0317(7)	0.0198(6)	0.0044(5)	−0.0052(5)	−0.0060(5)
C18	0.0192(6)	0.0229(6)	0.0181(5)	0.0030(5)	−0.0082(5)	−0.0032(5)
C19	0.0182(5)	0.0183(5)	0.0193(5)	−0.0004(4)	−0.0089(4)	0.0016(4)
C20	0.0130(5)	0.0180(5)	0.0145(5)	0.0005(4)	−0.0055(4)	−0.0005(4)
C21	0.0249(6)	0.0164(5)	0.0207(6)	−0.0003(4)	−0.0115(5)	−0.0003(5)
C22	0.0283(7)	0.0214(6)	0.0217(6)	−0.0044(5)	−0.0140(5)	0.0009(5)
C23	0.0234(6)	0.0274(7)	0.0182(6)	0.0013(5)	−0.0116(5)	−0.0013(5)
C24	0.0215(6)	0.0237(6)	0.0204(6)	0.0071(5)	−0.0106(5)	−0.0071(5)
C25	0.0174(5)	0.0207(6)	0.0180(5)	0.0039(4)	−0.0084(4)	−0.0061(4)

Table 19. Bond lengths and angles for Werz_dk341OX_a

Atom–Atom	Length [Å]
S1–O1	1.4863(10)
S1–C1	1.8427(13)
S1–C4	1.9239(13)
O2–C12	1.2264(15)
C1–C6	1.4926(17)
C1–C2	1.5408(17)
C1–C5	1.5502(17)
C2–C3	1.5712(17)
C2–H2A	0.9900
C2–H2B	0.9900
C3–C12	1.5239(17)
C3–C5	1.5546(17)
C3–C4	1.5570(17)
C4–C20	1.5209(16)
C4–C19	1.5344(17)
C5–H5A	0.9900
C5–H5B	0.9900
C6–C7	1.3945(19)
C6–C11	1.3957(19)
C7–C8	1.395(2)
C7–H7	0.9500
C8–C9	1.385(2)
C8–H8	0.9500
C9–C10	1.389(2)
C9–H9	0.9500
C10–C11	1.3925(19)
C10–H10	0.9500
C11–H11	0.9500
C12–C13	1.4891(17)
C13–C14	1.3956(17)
C13–C18	1.4037(17)
C14–C15	1.3909(18)
C14–H14	0.9500
C15–C16	1.385(2)
C15–H15	0.9500
C16–C17	1.393(2)
C16–H16	0.9500
C17–C18	1.3861(18)
C17–H17	0.9500
C18–H18	0.9500
C19–H19A	0.9800
C19–H19B	0.9800
C19–H19C	0.9800
C20–C25	1.3980(17)
C20–C21	1.4012(17)
C21–C22	1.3893(18)
C21–H21	0.9500
C22–C23	1.3863(19)
C22–H22	0.9500
C23–C24	1.3890(19)
C23–H23	0.9500
C24–C25	1.3921(18)
C24–H24	0.9500
C25–H25	0.9500

Atom–Atom–Atom	Angle [°]
O1–S1–C1	108.50(6)
O1–S1–C4	107.85(6)
C1–S1–C4	87.25(6)
C6–C1–C2	124.21(10)
C6–C1–C5	123.19(10)
C2–C1–C5	87.92(9)
C6–C1–S1	113.37(9)
C2–C1–S1	101.91(8)
C5–C1–S1	101.25(8)
C1–C2–C3	84.51(9)
C1–C2–H2A	114.6
C3–C2–H2A	114.6
C1–C2–H2B	114.6
C3–C2–H2B	114.6
H2A–C2–H2B	111.7
C12–C3–C5	116.45(10)
C12–C3–C4	119.24(10)
C5–C3–C4	104.79(9)
C12–C3–C2	119.16(10)
C5–C3–C2	86.69(9)
C4–C3–C2	105.18(9)
C20–C4–C19	109.83(10)
C20–C4–C3	116.85(10)
C19–C4–C3	115.95(10)
C20–C4–S1	107.09(8)
C19–C4–S1	106.96(8)
C3–C4–S1	98.70(8)
C1–C5–C3	84.76(9)
C1–C5–H5A	114.5
C3–C5–H5A	114.5
C1–C5–H5B	114.5
C3–C5–H5B	114.5
H5A–C5–H5B	111.6
C7–C6–C11	119.31(12)
C7–C6–C1	120.72(12)
C11–C6–C1	119.96(12)
C6–C7–C8	120.17(14)
C6–C7–H7	119.9
C8–C7–H7	119.9
C9–C8–C7	120.15(14)
C9–C8–H8	119.9
C7–C8–H8	119.9
C8–C9–C10	119.99(13)
C8–C9–H9	120.0
C10–C9–H9	120.0
C9–C10–C11	120.08(14)
C9–C10–H10	120.0
C11–C10–H10	120.0
C10–C11–C6	120.27(13)
C10–C11–H11	119.9
C6–C11–H11	119.9
O2–C12–C13	119.59(11)
O2–C12–C3	118.91(11)
C13–C12–C3	121.50(10)
C14–C13–C18	119.46(11)
C14–C13–C12	122.92(11)
C18–C13–C12	117.60(11)
C15–C14–C13	119.95(12)
C15–C14–H14	120.0
C13–C14–H14	120.0
C16–C15–C14	120.16(12)
C16–C15–H15	119.9
C14–C15–H15	119.9
C15–C16–C17	120.44(12)
C15–C16–H16	119.8
C17–C16–H16	119.8
C18–C17–C16	119.65(12)
C18–C17–H17	120.2
C16–C17–H17	120.2
C17–C18–C13	120.32(12)
C17–C18–H18	119.8
C13–C18–H18	119.8
C4–C19–H19A	109.5
C4–C19–H19B	109.5
H19A–C19–H19B	109.5
C4–C19–H19C	109.5
H19A–C19–H19C	109.5
H19B–C19–H19C	109.5
C25–C20–C21	117.84(11)
C25–C20–C4	123.60(11)
C21–C20–C4	118.55(11)
C22–C21–C20	121.04(12)
C22–C21–H21	119.5
C20–C21–H21	119.5
C23–C22–C21	120.43(12)
C23–C22–H22	119.8
C21–C22–H22	119.8
C22–C23–C24	119.31(12)
C22–C23–H23	120.3
C24–C23–H23	120.3
C23–C24–C25	120.33(12)
C23–C24–H24	119.8
C25–C24–H24	119.8
C24–C25–C20	121.01(12)
C24–C25–H25	119.5
C20–C25–H25	119.5

Table 20. Torsion angles for Werz_dk341OX_a

Atom–Atom–Atom–Atom	Torsion Angle [°]
O1–S1–C1–C6	−73.28(10)
C4–S1–C1–C6	178.81(9)
O1–S1–C1–C2	62.54(9)
C4–S1–C1–C2	−45.37(8)
O1–S1–C1–C5	152.78(8)
C4–S1–C1–C5	44.86(8)
C6–C1–C2–C3	−159.31(12)
C5–C1–C2–C3	−29.69(8)

S1–C1–C2–C3	71.37(8)
C1–C2–C3–C12	148.23(11)
C1–C2–C3–C5	29.63(8)
C1–C2–C3–C4	−74.80(10)
C12–C3–C4–C20	−63.85(14)
C5–C3–C4–C20	68.67(12)
C2–C3–C4–C20	159.23(10)
C12–C3–C4–C19	68.17(14)
C5–C3–C4–C19	−159.31(10)
C2–C3–C4–C19	−68.75(12)
C12–C3–C4–S1	−178.09(9)
C5–C3–C4–S1	−45.57(9)
C2–C3–C4–S1	44.99(9)
C6–C1–C5–C3	160.46(11)
C2–C1–C5–C3	30.03(8)
S1–C1–C5–C3	−71.71(8)
C12–C3–C5–C1	−150.51(10)
C4–C3–C5–C1	75.42(10)
C2–C3–C5–C1	−29.42(8)
C2–C1–C6–C7	−26.86(19)
C5–C1–C6–C7	−139.95(13)
S1–C1–C6–C7	97.60(13)
C2–C1–C6–C11	152.60(12)
C5–C1–C6–C11	39.50(18)
S1–C1–C6–C11	−82.95(14)
C11–C6–C7–C8	−1.6(2)
C1–C6–C7–C8	177.89(13)
C6–C7–C8–C9	1.4(2)
C7–C8–C9–C10	−0.2(2)
C8–C9–C10–C11	−1.0(2)
C9–C10–C11–C6	0.8(2)
C7–C6–C11–C10	0.4(2)
C1–C6–C11–C10	−179.03(13)
C5–C3–C12–O2	−12.01(16)
C4–C3–C12–O2	115.24(13)
C2–C3–C12–O2	−113.76(13)
C5–C3–C12–C13	168.91(10)
C4–C3–C12–C13	−63.84(15)
C2–C3–C12–C13	67.15(15)
O2–C12–C13–C14	149.81(13)
C3–C12–C13–C14	−31.11(17)
O2–C12–C13–C18	−28.43(17)
C3–C12–C13–C18	150.65(12)
C18–C13–C14–C15	−1.24(19)
C12–C13–C14–C15	−179.45(12)
C13–C14–C15–C16	0.1(2)
C14–C15–C16–C17	1.2(2)
C15–C16–C17–C18	−1.5(2)
C16–C17–C18–C13	0.4(2)
C14–C13–C18–C17	0.98(19)
C12–C13–C18–C17	179.28(12)
C19–C4–C20–C25	−115.69(13)
C3–C4–C20–C25	19.07(16)
S1–C4–C20–C25	128.51(11)
C19–C4–C20–C21	62.85(14)
C3–C4–C20–C21	−162.39(11)
S1–C4–C20–C21	−52.95(13)
C25–C20–C21–C22	1.69(19)

C4–C20–C21–C22	−176.94(12)
C20–C21–C22–C23	0.3(2)
C21–C22–C23–C24	−1.7(2)
C22–C23–C24–C25	1.1(2)
C23–C24–C25–C20	0.9(2)
C21–C20–C25–C24	−2.29(18)
C4–C20–C25–C24	176.26(11)

References

1 Q. Fu, S. Cao, J. Wang, X. Lv, H. Wang, X. Zhao and Z. Jiang, Enantioselective 2π + 2σ Cycloadditions of Bicyclo1.1.0butanes with Vinylazaarenes through Asymmetric Photoredox Catalysis, *J. Am. Chem. Soc.*, 2024, **146**, 8372–8380.
2 J.-J. Wang, L. Tang, Y. Xiao, W.-B. Wu, G. Wang and J.-J. Feng, [Copy] Switching between the 2π+2σ and Hetero-4π+2σ Cycloaddition Reactivity of Bicyclobutanes with Lewis Acid Catalysts Enables the Synthesis of Spirocycles and Bridged Heterocycles, *Angew. Chem. Int. Ed.*, 2024, **63**, e202405222.
3 Y. Xiao, F. Wu, L. Tang, X. Zhang, M. Wei, G. Wang and J.-J. Feng, [Copy] Divergent Synthesis of Sulfur-Containing Bridged Cyclobutanes by Lewis Acid Catalyzed Formal Cycloadditions of Pyridinium 1,4-Zwitterionic Thiolates and Bicyclobutanes, *Angew. Chem. Int. Ed.*, 2024, **63**, e202408578.
4 F. Sachse and C. Schneider, Direct Access to 1,3‐Oxathiolan‐5‐ones through (3+2)‐Cycloaddition of Thioketones and Acetylenedicarboxylic Acid, *Eur. J. Org. Chem.*, 2023, **26**. DOI: 10.1002/ejoc.202300834.
5 A. U. Augustin, M. Sensse, P. G. Jones and D. B. Werz, Stereospecific Reactions of Donor-Acceptor Cyclopropanes with Thioketones: Access to Highly Substituted Tetrahydrothiophenes, *Angew. Chem. Int. Ed.*, 2017, **56**, 14293–14296.
6 Y. Liang, F. Paulus, C. G. Daniliuc and F. Glorius, Catalytic Formal 2π+2σ Cycloaddition of Aldehydes with Bicyclobutanes: Expedient Access to Polysubstituted 2-Oxabicyclo2.1.1hexanes, *Angew. Chem. Int. Ed.*, 2023, **62**, e202305043.
7 K. Dhake, K. J. Woelk, J. Becica, A. Un, S. E. Jenny and D. C. Leitch, Beyond Bioisosteres: Divergent Synthesis of Azabicyclohexanes and Cyclobutenyl Amines from Bicyclobutanes, *Angew. Chem. Int. Ed.*, 2022, **61**, e202204719.
8 Bruker, *SAINT*, Madison, Wisconsin, USA.
9 L. Krause, R. Herbst-Irmer, G. M. Sheldrick and D. Stalke, Comparison of silver and molybdenum microfocus X-ray sources for single-crystal structure determination, *J. Appl. Crystallogr.*, 2015, **48**, 3–10.
10 G. M. Sheldrick, SHELXT - integrated space-group and crystal-structure determination, *Acta crystallographica. Section A, Foundations and advances*, 2015, **71**, 3–8.
11 G. M. Sheldrick, Crystal structure refinement with SHELXL, *Acta crystallographica. Section C, Structural chemistry*, 2015, **71**, 3–8.
12 C. R. Groom, I. J. Bruno, M. P. Lightfoot and S. C. Ward, The Cambridge Structural Database, *Acta crystallographica Section B, Structural science, crystal engineering and materials*, 2016, **72**, 171–179.
13 D. Kratzert, FinalCif, https://dkratzert.de/finalcif.html.
